# Mechanisms and Specificity of Phenazine Biosynthesis Protein PhzF

**DOI:** 10.1038/s41598-017-06278-w

**Published:** 2017-07-24

**Authors:** Christina Diederich, Mario Leypold, Martin Culka, Hansjörg Weber, Rolf Breinbauer, G. Matthias Ullmann, Wulf Blankenfeldt

**Affiliations:** 1 Structure and Function of Proteins, Helmholtz Centre for Infection Research, Inhoffenstr. 7, 38124 Braunschweig, Germany; 2 Institute of Organic Chemistry, Graz University of Technology, Stremayrgasse 9, 8010 Graz, Austria; 3 Structural Biology/Bioinformatics, University of Bayreuth, Universitätsstr. 30, 95447 Bayreuth, Germany; 4 Institute for Biochemistry, Biotechnology and Bioinformatics, Technische Universität Braunschweig, Spielmannstr. 7, 38106 Braunschweig, Germany; 5 Department of Biochemistry, University of Bayreuth, Universitätsstr. 30, 95447 Bayreuth, Germany

## Abstract

Phenazines are bacterial virulence and survival factors with important roles in infectious disease. PhzF catalyzes a key reaction in their biosynthesis by isomerizing (2 *S*,3 *S*)-2,3-dihydro-3-hydroxy anthranilate (DHHA) in two steps, a [1,5]-hydrogen shift followed by tautomerization to an aminoketone. While the [1,5]-hydrogen shift requires the conserved glutamate E45, suggesting acid/base catalysis, it also shows hallmarks of a sigmatropic rearrangement, namely the suprafacial migration of a non-acidic proton. To discriminate these mechanistic alternatives, we employed enzyme kinetic measurements and computational methods. Quantum mechanics/molecular mechanics (QM/MM) calculations revealed that the activation barrier of a proton shuttle mechanism involving E45 is significantly lower than that of a sigmatropic [1,5]-hydrogen shift. QM/MM also predicted a large kinetic isotope effect, which was indeed observed with deuterated substrate. For the tautomerization, QM/MM calculations suggested involvement of E45 and an active site water molecule, explaining the observed stereochemistry. Because these findings imply that PhzF can act only on a limited substrate spectrum, we also investigated the turnover of DHHA derivatives, of which only *O*-methyl and *O*-ethyl DHHA were converted. Together, these data reveal how PhzF orchestrates a water-free with a water-dependent step. Its unique mechanism, specificity and essential role in phenazine biosynthesis may offer opportunities for inhibitor development.

## Introduction

Enzymes have evolved to convert nearly every class of molecule found in nature by catalyzing nearly every type of reaction mechanism known in chemistry. This versatility often makes it difficult to identify the mechanistic route that an enzyme follows even in cases where the structures of substrate, product and the enzyme itself are known. As a consequence, opportunities for the development of mechanism-based inhibitors in drug discovery or for the turnover of non-natural substrates in biotechnological applications are easily missed.

**Figure 1:**
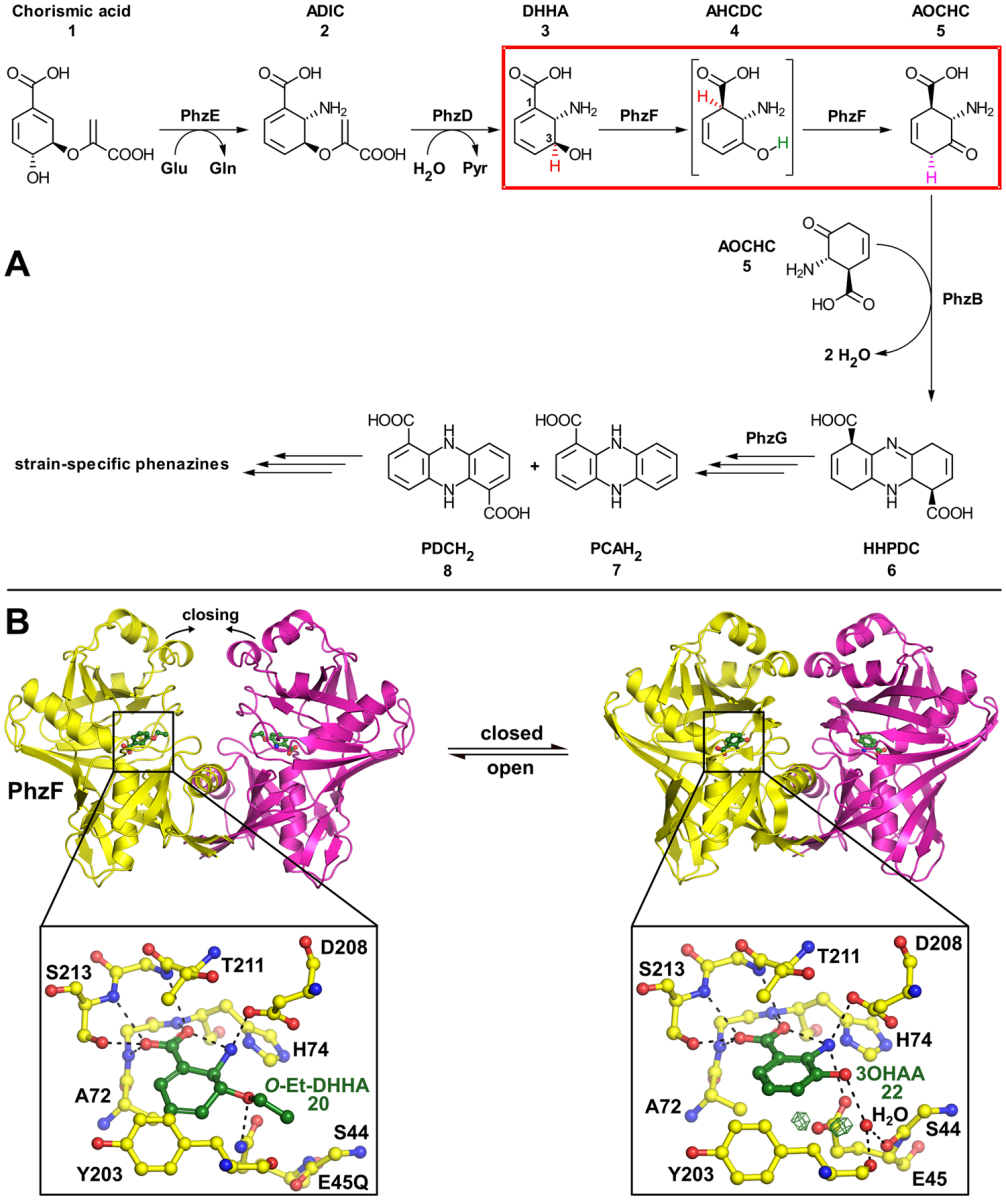
Phenazine biosynthesis and the role of PhzF. (**A**) Overview over the phenazine biosynthesis pathway. PhzE converts chorismic acid (**1**) to (5 *S*,6 *S*)-6-amino-5-(1-carboxyethenyloxy)-1,3-cyclohexadiene-1-carboxylic acid (ADIC, **2**), which is then hydrolyzed to (2 *S*,3 *S*)-2,3-dihydro-3-hydroxy anthranilic acid (DHHA, **3**) by PhzD. PhzF (red box) catalyzes isomerization to (1 *R*,6 *S*)-6-amino-5-oxo-2-cyclohexene-1-carboxylic acid (AOCHC, **5**) via (1 *R*,6 *S*)-6-amino-5-hydroxy-2,4-cyclohexadiene-1-carboxylic acid (AHCHC, **4**) by inducing a [1,5]-hydrogen shift (red H-atom) and a stereospecific tautomerization (green and magenta H-atoms). PhzB then condenses two AOCHC molecules (**5**) to hexahydrophenazine-1,6-dicarboxylic acid (HHPDC, **6**) before oxidation steps involving PhzG generate dihydro-phenazine-1-carboxylic acid (PCAH_2_, **7**) and dihydro-phenazine-1,6-dicarboxylic acid (PDCH_2_, **8**) as precursors for strain-specific phenazine derivatives. (**B**) Crystal structures of the PhzF homodimer in the open (PDB entry 5IWE) and closed (PDB entry 1U1W) conformation. The magnified insert on the left shows *O*-ethyl-DHHA (**20**) bound to the active center of the open form, whereas the insert on the right shows the substrate analogue 3OHAA (**22**) bound to the closed form. The green mesh displays |F_O_-F_C_| difference electron density at 3 σ and indicates that E45 may be protonated in this complex. This figure was prepared with PyMOL^53^.

Phenazine biosynthesis protein PhzF (E.C. 5.3.3.17) is an example of an enzyme that catalyzes an unusual reaction for which substantially different mechanistic routes can be proposed. The phenazines are a class of over 150 bacterial secondary metabolites with redox properties that enable them to act as broad specificity antibiotics as well as virulence and survival factors e.g. in infections caused by the opportunistic pathogen *Pseudomonas aeruginosa* ^1, 2^. Their biosynthesis involves the condensation of two chorismate-derived precursors into the symmetrical phenazine scaffold (Fig. 1A). In this pathway, PhzF was found to act as an isomerase of (2 *S*,3 *S*)-2,3-dihydro-3-hydroxyanthranilic acid (DHHA, **3**)^3, 4^. The reaction is believed to require two chemical steps, namely a hydrogen shift from C3 to C1 of the substrate and a subsequent tautomerization of the enol intermediate. Two molecules of the ensuing highly reactive ketone (1 *R*,6 *S*)-6-amino-5-oxo-2-cyclohexene-1-carboxylic acid (AOCHC, **5**) then undergo condensation to hexahydro-phenazine-1,6-dicarboxylic acid (HHPDC, **6**), followed by oxidations to dihydro-phenazine-1,6-dicarboxylic acid (PDCH_2_, **8**) and/or dihydro-phenazine-1-carboxylic acid (PCAH_2_, **7**) as precursors for strain-specific derivatives^5, 6^.

More detailed investigation of the reaction catalyzed by PhzF revealed several interesting features of the first isomerization step, the hydrogen shift from C3 to C1 to form enol intermediate (1 *R*,6 *S*)-6-amino-5-hydroxy-2,4-cyclohexadiene-1-carboxylic acid (AHCDC, **4**). First, when the PhzF-catalyzed isomerization of DHHA (**3**) is carried out in D_2_O, no incorporation of deuterium at C1 of AOCHC (**5**) is observed^4^. This indicates that the isomerization is the shift of a proton rather than a more common acid/base-catalyzed reaction in which one proton is abstracted and another is retransferred to the substrate, which would ultimately lead to incorporation of a proton from the solvent. Second, structural studies of the enzymes acting downstream of PhzF, namely PhzB and PhzG, revealed *R*-configuration of the newly created stereogenic center at C1 of AOCHC (**5**)^7, 8^, showing that the proton shift proceeds in a suprafacial fashion with respect to the cyclohexadienyl ring of DHHA (**3**). Third, the migrating C3-proton of DHHA (**3**) is expected to be rather non-acidic, suggesting that it cannot easily be moved by amino acid residues of a cofactor-free enzyme like PhzF. Together, these findings suggest that PhzF may not use the essential glutamic acid E45 as a proton shuttle as originally proposed by us^4^, but rather catalyzes a sigmatropic [1,5]-hydrogen shift, i.e. a concerted proton movement that is controlled by frontier orbital symmetry.

Sigmatropic mechanisms are relatively rare in the biochemical world. Examples of enzymes catalyzing such reactions include chorismate mutase, which induces a [3,3] Claisen rearrangement in chorismic acid^9^, isochorismate pyruvate lyase catalyzing a [1,5]-prototropic fragmentation^10^, precorrin-8x methyl mutase CobH believed to catalyze a [1,5]-sigmatropic methyl shift^11^ and enzymes that are discussed to perform Diels-Alder [4 + 2]-cycloadditions^12, 13^. On the other hand, although familiar to the synthetic chemist, enzyme-catalyzed sigmatropic [1,5]-hydrogen shifts at dienes are not known, which would make PhzF the first example of an enzyme catalyzing such a reaction.

Here, we have set out to analyze different potential catalytic mechanisms of both isomerization steps in PhzF, using a combination of synthetic, bioanalytical and computational methods. In addition, we have assessed the substrate spectrum of PhzF to explore if its unusual activity can be harnessed for interesting biochemical transformations. Our analyses show that a sigmatropic [1,5]-hydrogen shift in DHHA (**3**) would have to surpass a significantly higher activation barrier than an acid/base mechanism employing E45 as a proton-shuttling residue. Together with a tightly bound water molecule, E45 is also involved in the stereospecific tautomerization of AHCDC (**4**) to AOCHC (**5**), revealing how PhzF coordinates a water-free with a water-dependent catalytic step and explaining why only a small number of O-alkylated derivatives of DHHA (**3**) are accepted as substrates.

## Results

### Turnover of C3-deuterated DHHA (d-3) confirms that PhzF catalyzes a [1,5]-hydrogen shift

**Figure 2:**
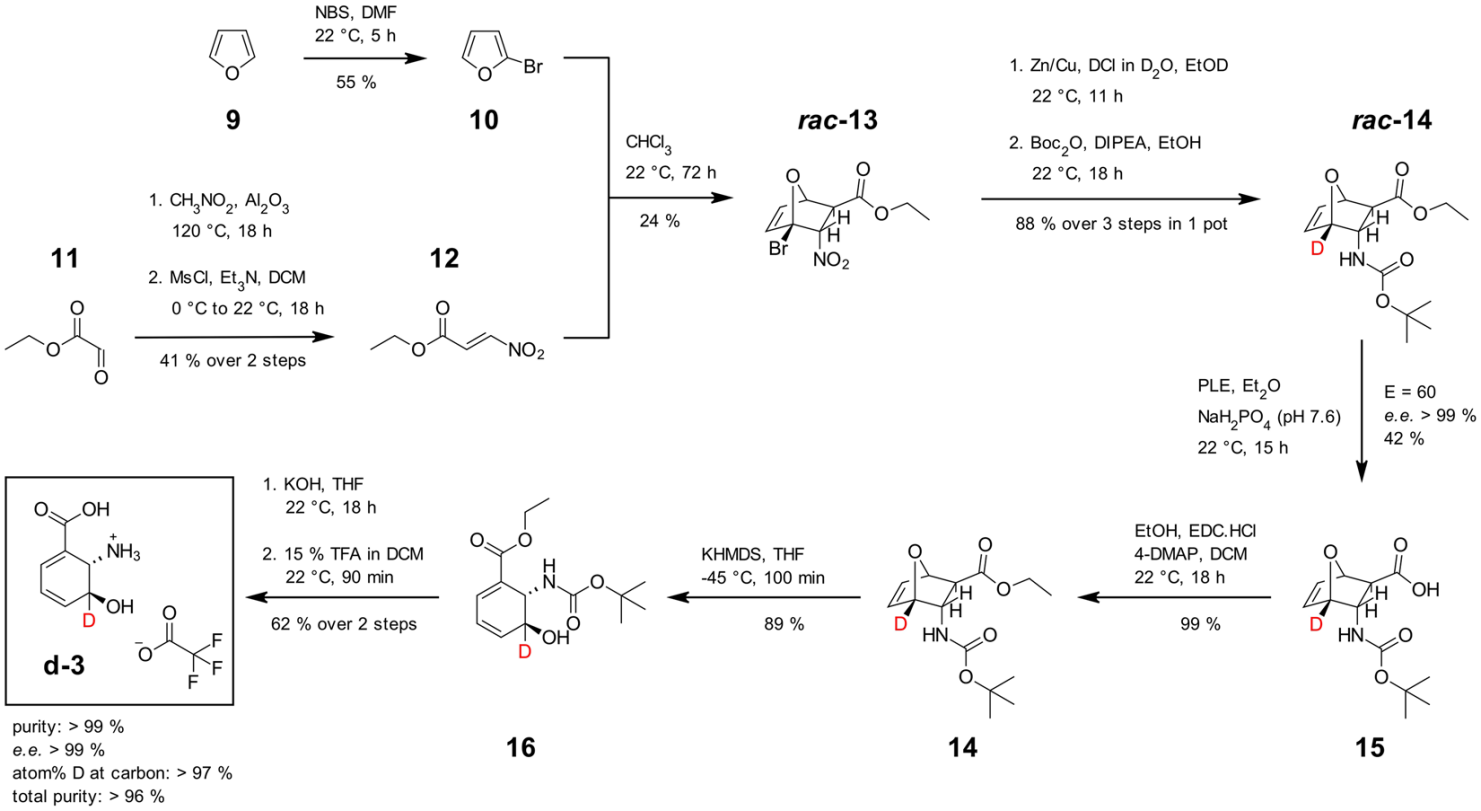
Synthesis of C3-deuterated DHHA. Diels-Alder reaction of bromofuran (**10**) and (*E*)-nitroacryl ethylester (**12**) furnishes the racemic bicyclic intermediate ***rac*** **-13**, which is then deuterated by reductive debromination in the presence of DCl. The desired enantiomer **14** is obtained by kinetic resolution with pig liver esterase (PLE). Treatment with potassium hexamethyldisilazide (KHDMS) and deprotection then yields the desired C3-deuterated DHHA (**d-3**). BOC: tert-butyloxycarbonyl; DCM: dichloromethane; DIPEA: *N,N*-diisopropylethylamine; 4-DMAP: 4-dimethylaminopyridine; DMF: *N,N*-dimethylformamide; EDC: 1-ethyl-3-(3-dimethylaminopropyl)-carbodiimide; MsCl: methanesulfonylchloride; NBS: *N*-bromo succinimide; TFA: trifluoroacetic acid; THF: tetrahydrofuran.

**Figure 3:**
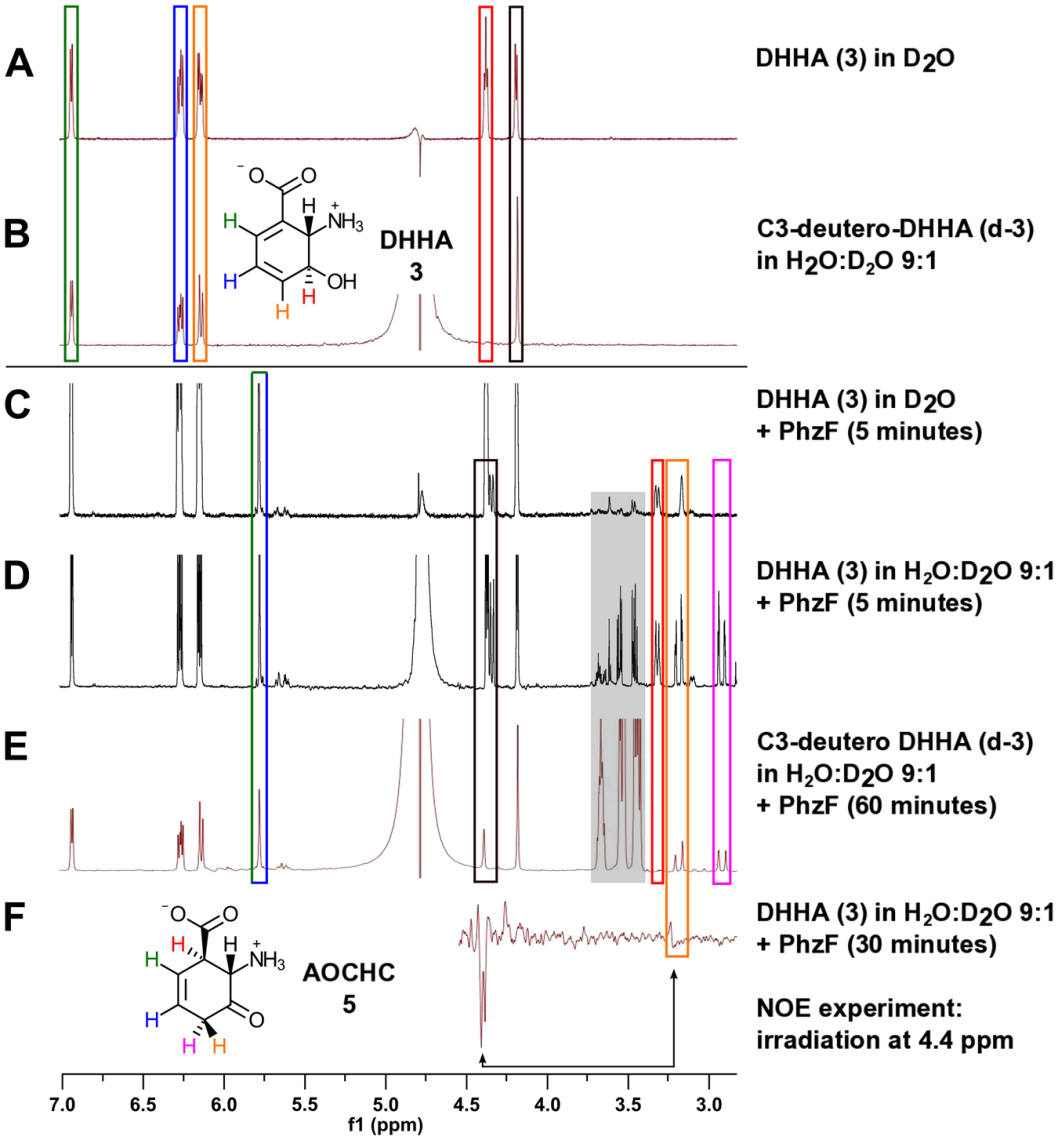
^1^H-NMR spectroscopic data. Synthetic DHHA (**3, A**) and C3-deutero-DHHA (**d-3**, **B**) have been incubated with PhzF for the indicated times (**C–E**). Resonance assignments for DHHA (**3**) and AOCHC (**5**) are indicated by colored boxes. The grey rectangle marks enzyme buffer components. Panel F shows an NOE experiment to assign proton resonances at C4 of AOCHC (**5**). The spectra demonstrate that PhzF recycles the proton at C3 of DHHA (**3**) to C1 of AOCHC (**5**; red rectangles) and catalyzes a stereospecific tautomerization with incorporation of a solvent-derived proton at C4 of AOCHC (**5**; orange and magenta rectangles).

In order to provide additional evidence for a [1,5]-hydrogen shift in the isomerization of DHHA (**3**) by PhzF and for the investigation of kinetic isotope effects (KIEs), we developed a synthetic route towards enantiomerically pure (2 *S*,3 *S*)-DHHA (**3**) that allows for deuteration at C3, adapting a synthetic approach by Steel and coworkers^14–17^. This route (Fig. 2) employs a Diels-Alder reaction of 2-bromofuran (**10**) with (*E*)-nitroacrylic esters (**12**), deuterodehalogenation mediated by a Zn/Cu-couple, and kinetic resolution of enantiomers with pig liver esterase (PLE), requiring 10 steps in the longest linear sequence (12 steps with re-esterification and repeated kinetic resolution with PLE) and producing the desired compounds in >99% ee with an overall yield of 2% (Fig. 3A,B).

We then followed the turnover of C3-deuterated DHHA (**d-3**) by ^1^H-NMR spectroscopy, expecting the absence of a signal for the proton at C1 of the product. Indeed, the signal for the C1-proton was missing, corroborating that the abstracted and re-donated proton are identical, i.e. that the reaction comprises a [1,5]-shift of a proton within the substrate (Fig. 3E).

### QM/MM calculations reveal the nature of the isomerization of DHHA (3) to AHCDC (4)

While the experiment with C3-deuterated DHHA (**d-3**) confirms that the catalytic cycle of PhzF contains a [1,5]-hydrogen shift, it does not allow distinguishing between a sigmatropic and an acid/base-catalytic mechanism in this step. Therefore, quantum mechanics/molecular mechanics (QM/MM) calculations were performed to analyze the energy profiles and hence discriminate different hypothetical reaction paths of the first isomerization catalyzed by PhzF.

To initiate this analysis, it was first necessary to investigate the protonation of titratable amino acids and of the substrate bound to the active site. On ligand binding, the homodimeric PhzF changes its conformation to a closed state with only one water molecule remaining in the active site (Fig. 1B). For this reason, the crystal structure of PhzF in complex with DHHA (**3**; PDB entry 1U1X)^4^ was used to assess protonation states by Monte Carlo titrations within the continuum electrostatic model. According to calculations at pH 7.5, DHHA (**3**) binds in the zwitterionic form with deprotonated carboxylate and protonated amino group. This is in agreement with titration experiments of free DHHA (**3**), which gave a pK_a_ value of 3.3 ± 0.4 for the carboxylate and of 8.6 ± 0.4 for the amino group, respectively, showing that the zwitterionic form is also the preferred species in solution (Supplemental Fig. S1). D208 is deprotonated and H74 is in the neutral Nε-protonated state. Surprisingly, the indispensable E45 is predicted to be protonated with a calculated deprotonation energy of 1.7 kcal/mol, corresponding to a pK_a_ of 9 (Fig. S2). The deprotonation energy increases to even 3.7 kcal/mol for the complex with 3-hydroxy anthranilic acid (3OHAA, **22**), which prompted us to reexamine our previously determined crystal structure of PhzF in complex with this compound (PDB entry 1U1W)^4^. Indeed, unexplained difference electron density at two positions within O-H-bond distance of the carboxylate group seems to indicate protonation of E45 in the complex with this inhibitor (Fig. 1B). However, since these crystals have been obtained at pH 4.6 and the resolution of this complex (1.35 Å) may not be high enough to identify hydrogen atoms unequivocally, this observation may not serve as a proof for protonation of E45 in the complex with DHHA (**3**) under physiological conditions.

**Figure 4:**
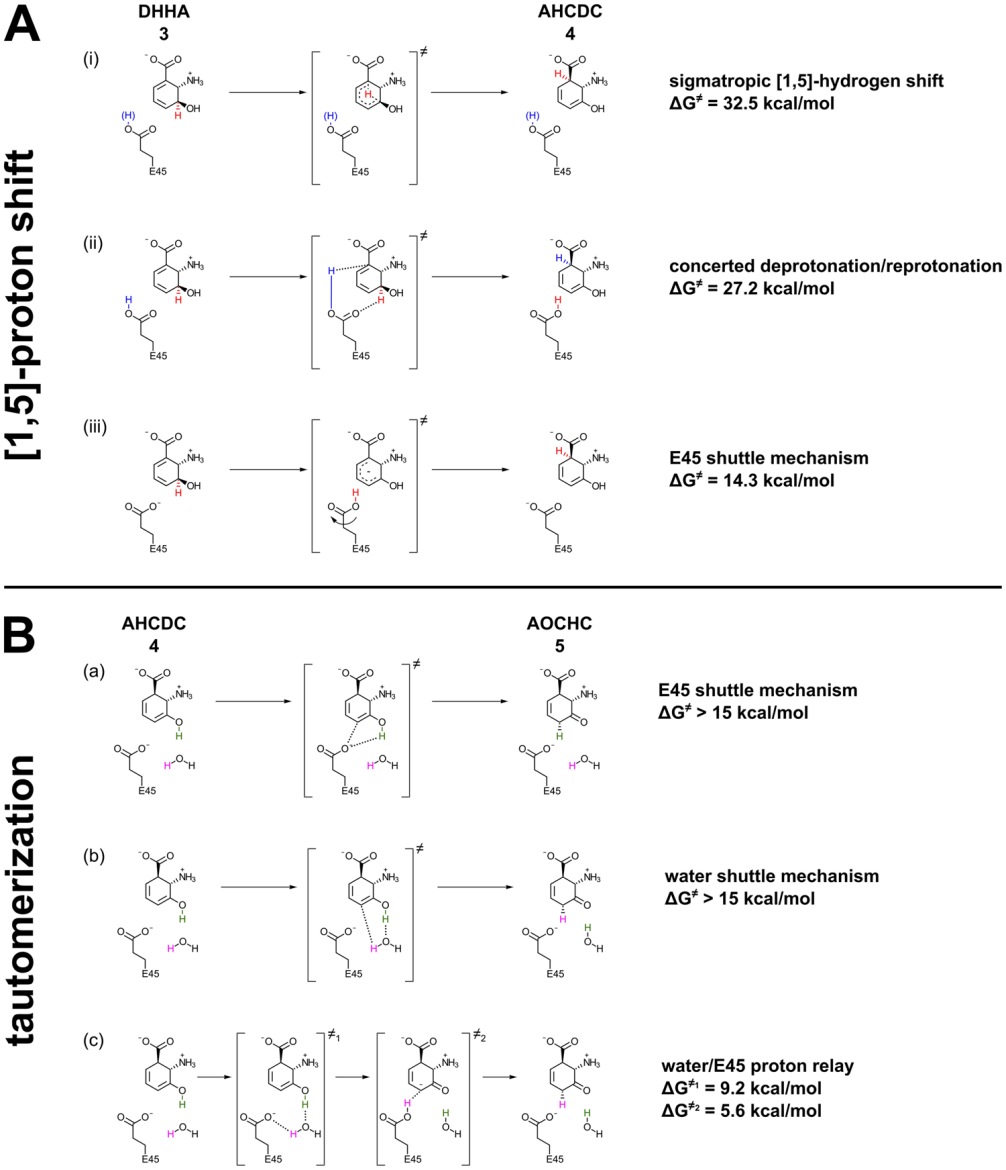
Investigated mechanisms of the isomerization reactions catalyzed by PhzF. (**A**) Hypothetical mechanisms of the [1,5]-proton shift from DHHA (**3**) to AHCDC (**4**). (**B**) Hypothetical mechanisms for the tautomerization of AHCDC (**4**) to AOCHC (**5**). Energy values for the activation energy barriers of the chemical steps shown in the figure (ΔG^≠^) have been obtained by QM/MM methods. Together, these values suggest that PhzF initiates isomerization of DHHA (**3**) by employing E45 to shuttle a proton from C3 to C1 (**A**, iii), followed by a proton-relay mechanism that stereospecifically tautomerizes AHCDC (**4**) to AOCHC (**5**; **B**, c).

Next, we used QM/MM methods to further investigate the following three reaction paths for the isomerization of DHHA (**3**) to AHCDC (**4**; Fig. 4A):

(i) a direct concerted transfer of the proton from C3 to C1 of the substrate (sigmatropic [1,5]-hydrogen shift) starting with protonated or deprotonated E45,
(ii) a concerted transfer mechanism in which the initially protonated E45 transfers its proton to C1 while it simultaneously abstracts a proton from C3, and
(iii) a stepwise acid/base mechanism in which deprotonated E45 shuttles the proton from C3 to C1, following our previous mechanistic proposal^4^.

Based on the *in silico* titrations described above, we started the investigation with the following active site protonation states: zwitterionic DHHA (**3**), deprotonated D208, neutral ε-isomer of H74 and protonated E45. For the direct mechanism (i) a very high barrier of 32.5 kcal/mol was found using the BP86 functional, which may be even higher, since the BP86 functional is known to underestimate transition state energies^18^. This high transition state energy indicates that the sigmatropic [1,5]-hydrogen shift with initially protonated E45 can be ruled out.

Next, the concerted mechanism (ii) was tested, even if the H/D-NMR exchange experiments mentioned above make this mechanism unlikely. We found a cationic transition state with both C1 and C3 protonated, which subsequently gets deprotonated at C3 by E45. This transition state has an energy of 27.2 kcal/mol above the substrate-bound state, again too high to be involved in a realistic reaction path.

Failing to find an energetically feasible mechanism with protonated E45 as a starting point, we next investigated reaction paths in which E45 is deprotonated at the onset of catalysis, i.e. a stepwise acid/base-mechanism (iii) and again a direct proton transfer via sigmatropic [1,5]-hydrogen shift (i). Given that deprotonation of E45 is predicted to require 1.7 kcal/mol, the energy of all transition paths has to be raised by this amount. For the sigmatropic [1,5]-hydrogen shift, a similar barrier as in the case of protonated E45 was calculated, making this reaction as unlikely as when starting with neutral E45. Interestingly, however, the refinement of this path results in a mechanism that follows the stepwise acid/base reaction (iii). For this third alternative, we found a barrier of 13–14 kcal/mol for the proton abstraction from C3 of DHHA (**3**) using the BP86 functional, revealing that this is the most plausible of the three mechanisms. We therefore refined this trajectory further by conjugate peak refinement (CPR) using the B3LYP functional, which gives more realistic barriers but at higher computational cost. In these calculations, the energy barrier for the deprotonation of DHHA (**3**) at C3 increased to 17.8 kcal/mol and analysis of vibrational frequencies on all stationary points of the pathway showed either no imaginary frequencies, indicating stable energy minima, or single imaginary frequency, indicating first order saddle points. The energies were then corrected by zero-point energy contribution calculated from the non-imaginary vibration frequencies. The resulting energy barrier for deprotonation of C3 decreased to 14.3 kcal/mol, which results in an overall barrier of 16.0 kcal/mol after correcting for the fact that E45 needs to be in the deprotonated state to be able to act as a base in this step. This value is similar to the barrier calculated in a recent *in silico* study of PhzF by Liu and colleagues^19^, but these authors did not consider a pericyclic [1,5]-hydrogen shift as an alternative to the E45 shuttle mechanism previously proposed by us^4^ and rather tried to distinguish it from an acid/base mechanism involving both D208 and E45 as suggested by Parsons *et al*.^3^. However, this mechanism would not lead to the observed recycling of the C3-proton of DHHA (**3**) to C1 of the product AOCHC (**5**) and can thus be ruled out.

Following proton abstraction at C3, the now protonated E45 needs to turn towards C1 before reprotonation at this position can occur. The system remains at a high energy level during this process before AHCDC (**4**) is formed, which is accompanied by an energy loss of 20.3 kcal/mol.

In summary, these QM/MM calculations show that the first step in PhzF-mediated turnover does not employ a pericyclic mechanism but uses a stepwise acid/base catalysis with E45 acting as a proton shuttle instead.

### Keto-enol tautomerization of AHCDC (4) to AOCHC (5) involves E45 and a water molecule

Chemical intuition would predict that the tautomerization of enol AHCDC (**4**; Fig. 1A) is spontaneous. However, we found a considerable energy barrier of 14.2 kcal/mol for the uncatalyzed tautomerization in water, using a QM model comprising the substrate and two explicit water molecules surrounded by a continuum solvent model. In addition, NMR-experiments in D_2_O revealed exclusive and stereospecific incorporation of deuterium at C4 (Fig. 3C)^4^. Together, this suggests that the formation of AOCHC (**5**) from AHCDC (**4**) is catalyzed by PhzF. We therefore investigated a possible involvement of the enzyme in this tautomerization step.

The tautomerization reaction requires deprotonation of the 3-hydroxy group and protonation at C4 of AHCDC (**4**). Inspection of the crystal structures of PhzF in complex with DHHA (**3**) or 3OHAA (**22**) shows that only two groups can participate in these proton transfers, namely the side chain of E45 and the only tightly bound water molecule mentioned above (Fig. 1B). The energy barrier for turning E45 back to its starting position after protonating C1 is 3.3 kcal/mol, after which the system reaches an energy minimum with −11.5 kcal/mol relative to the initial enzyme/substrate complex. Monte Carlo titration reveals that the pK_a_ of E45 is 7.5 at this stage, indicating that it could initiate ketone formation by deprotonating the 3-hydroxy group of AHCDC (**4**). On the other hand, the tightly bound water molecule is involved in a strong hydrogen bond with this 3-hydroxy group (2.6 Å) and at the same time engages in hydrogen bonds with the backbone carbonyl of S44 (2.9 Å) and Y203 (2.8 Å), such that it is perfectly positioned to perform the required proton abstraction.

To distinguish if E45 or the water molecule acts as the base that initiates the tautomerization reaction and to gain insight into catalysis of this step, the energy profiles of three reaction paths have been analyzed (Fig. 4B):

- E45 deprotonates the 3-hydroxy group and protonates C4 of the anionic intermediate, i.e. E45 again acts as a proton shuttle;
- the tightly bound water molecule shuttles the proton instead;
- the water molecule deprotonates the 3-hydroxy group and transfers a proton to E45, which then protonates C4 (water/E45 proton relay).

Although CPR found feasible reaction pathways for mechanisms (a) and (b), their activation barriers were higher than 15 kcal/mol, which is comparable to the estimate for the uncatalyzed reaction and also similar to the transition state energy for the initial isomerization step from DHHA (**3**) to AHCDC (**4**). The highest activation barrier in mechanism (c), on the other hand, was 9.2 kcal/mol, making this mechanism the most probable of the three investigated ketonization pathways. Here, computations suggest that E45 turns towards the water molecule, which repositions slightly and establishes a new hydrogen bonding pattern in which it no longer interacts with the carbonyl of Y203. The water molecule then deprotonates the 3-hydroxy group and protonates the E45 in a concerted manner before E45 turns back to protonate C4.

All three ketonization mechanisms predict stereospecific protonation from the pro-*R* site of C4 in AHCDC (**4**), i.e. on the same face as in the first isomerization reaction. For corroboration, we have conducted NMR experiments with chemical shift predictions and Nuclear Overhauser Enhancement (NOE) measurements. Hybrid DFT computations were carried out to predict the chemical shifts of the pro-*R* and pro-*S* protons at C4 of AOCHC (**5**). These calculations yielded values of 2.61 and 3.19 ppm, respectively, in good agreement with spectral data (2.92 and 3.19 ppm, Fig. 3D,E). When DHHA (**3**) was turned over in D_2_O, the signal at 2.92 ppm predicted to belong to the pro-*R* proton vanished (Fig. 3C)^4^. Further, irradiation of the proton at C2 of AOCHC (**5**; 4.4 ppm) caused a small but discernable NOE at 3.2 ppm, the predicted chemical shift of the pro-*S* proton at C4 on the same face of the six-membered ring of AOCHC (**5**; Fig. 3F). Together, these data argue for a PhzF-catalyzed tautomerization with mechanism (c) as the most likely reaction path.

**Figure 5:**
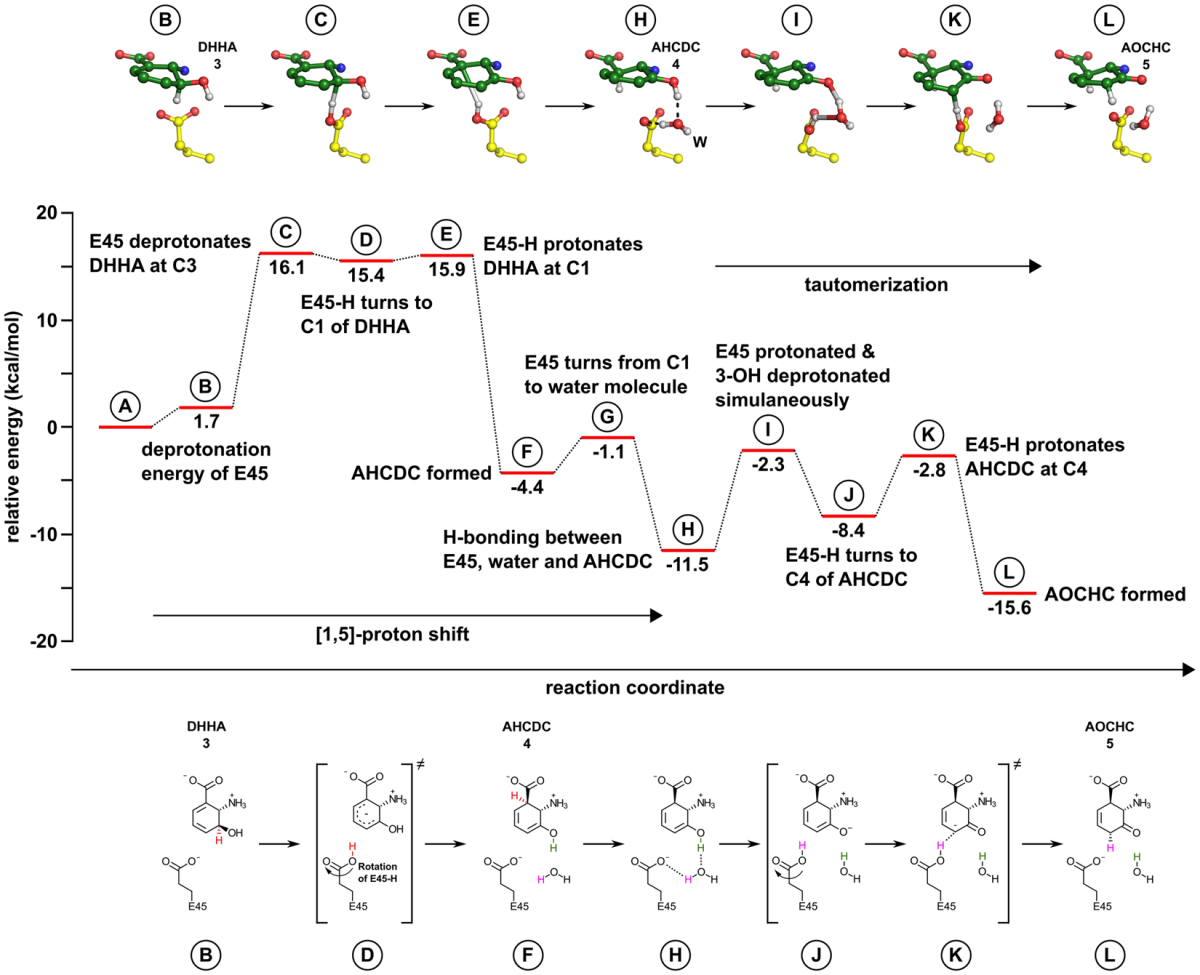
Energy profile of the complete catalytic cycle of PhzF. Calculated structures of intermediates are shown at the top, the most important chemical steps are shown at the bottom of the figure. Letters in circles correspond to respective states of the energy profile.

The complete energy profile of the catalytic cycle of PhzF together with structures of the most important intermediates is shown in Fig. 5. An animated version of the catalytic cycle can be found in the online supplementary information (Supplemental Movie S1).

### C3-deuteration of DHHA (3) leads to large primary kinetic isotope effects

The theoretical energy profile of the complete catalytic cycle (Fig. 5) implies that the deprotonation at C3 of DHHA (**3**) is the rate limiting step in PhzF catalysis, which should lead to a large primary KIE with C3-deuterated DHHA (**d-3**). Indeed, QM/MM zero point energy calculations with the C3-deuterated substrate indicate that only the C3 deprotonation step is significantly affected by deuteration, increasing the energy of the transition state by 0.9 kcal/mol. This increase corresponds to a calculated intrinsic kinetic isotope effect of 4.8.

In order to determine the primary KIE of C3-deuterium substitution experimentally, we developed a photometric assay that makes use of the fact that the substrate DHHA (**3**) absorbs UV light at 275 nm (ε_275_ = 6500 M^−1^ cm^−1^) whereas the product AOCHC (**5**) is photometrically silent at this wavelength (Fig. S3).

**Figure 6:**
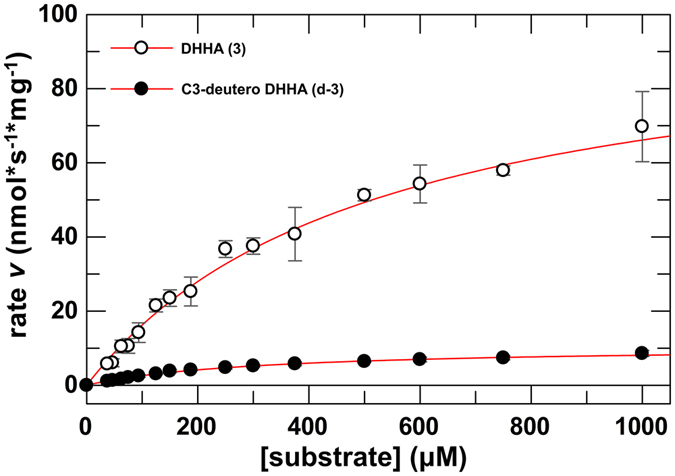
Determination of enzyme kinetic parameters of PhzF. The reaction velocity vs. substrate concentration for DHHA (**3**) or C3-deuterated DHHA (**d-3**) in H_2_O at pH 7.5 is shown. Enzyme kinetic parameters for DHHA (**3**) were determined as v_max_ = 100.34 ± 5.80 nmol s^−1^ mg^−1^ (k_cat_ = 3.23 ± 0.19 s^−1^) and K_M_ = 517 ± 57 µM, or as v_max_ = 10.60 ± 0.27 nmol s^−1^ mg^−1^ (k_cat_ = 0.34 ± 0.01 s^−1^) and K_M_ = 311 ± 14 µM for C3-deuterated DHHA (**d-3**), respectively.

At pH 7.5, enzyme kinetic parameters of v_max_ = 100.34 ± 5.80 nmol s^−1^ mg^−1^ (k_cat_ = 3.23 ± 0.19 s^−1^) and K_M_ = 517 ± 57 µM were found for DHHA (**3**; Fig. 6; Supplemental Table S1). The deuterated substrate **d-3** showed much slower turnover of v_max_ = 10.60 ± 0.27 nmol s^−1^ mg^−1^ (k_cat_ = 0.34 ± 0.01 s^−1^) and K_M_ = 311 ± 14 µM, corresponding to apparent KIE values of 9.5 ± 0.6 on v_max_ and 5.7 ± 0.8 on v_max_/K_M_.

### E45 cannot be replaced by aspartic acid

Our calculations hint at an essential double role of E45 as an acid/base catalyst in both steps of the enzyme-catalytic cycle of PhzF. While an E45A and an E45Q mutant were found to be inactive in previous work^4^, we tested if the glutamate could be replaced by aspartic acid. The E45D mutant displayed identical purification and crystallization behavior as the wildtype enzyme, but it was inactive in the photometric activity assay described above (data not shown). QM/MM calculations show that the energy barrier of a sigmatropic [1,5]-hydrogen shift was not significantly affected whereas the initial deprotonation step of the shuttle mechanism was disfavored by more than 15 kcal/mol by the E45D mutation (Fig. S4). This explains the absolute conservation of E45 in all PhzF orthologs known to date.

### PhzF is highly substrate-specific

**Figure 7:**
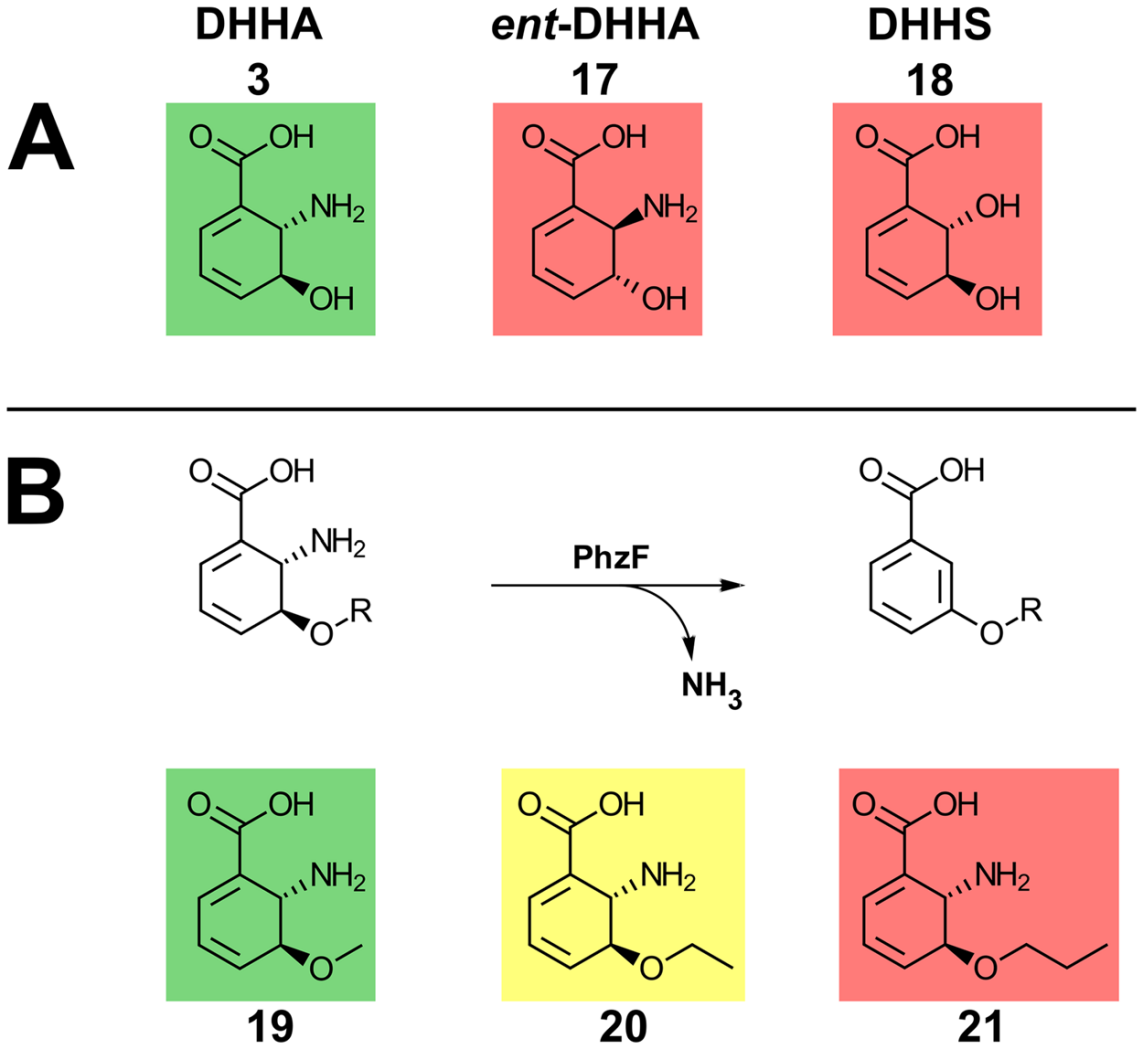
Substrate preferences of PhzF. (**A**) Turnover of isosters of DHHA, (**B**) conversion of *O*-alkylated DHHA-derivatives. Colors indicate velocities with which the respective compound is isomerized (green: fast; yellow: slow; red: no turnover).

The reaction catalyzed by PhzF is rather unique and it is therefore interesting to assess if the enzyme can be used to convert substrates other than DHHA (**3**). Towards this end, we have investigated the turnover of various analogues, including *ent*-DHHA (**17**), the 2-hydroxy derivative (2 *S*,3 *S*)-2,3-dihydro-3-dihydroxy salicylic acid (DHHS, **18**) and synthetic *O*-alkylated variants of DHHA (**19**–**21**; Fig. 7).

As expected from the stereochemistry of the mechanisms laid out above, the (2 *R*,3 *R*)-enantiomer of DHHA (*ent*-DHHA; **17**) was not turned over by PhzF. DHHS (**18**) was also not isomerized, which probably is a consequence of the effect on the pK_a_ values of E45 and of the proton at C3 (see Discussion).

*O*-Alkylated derivatives were synthesized in a similar procedure as DHHA (**3**). In contrast to DHHS (**18**), the *O*-methylated (**19**) and *O*-ethylated (**20**) compounds were turned over by PhzF, but because these molecules cannot undergo tautomerization, they eliminate ammonia to give the respective aromatic products (Figs 7 and S5), which may happen directly after deprotonation at C3 or after the [1,5]-proton shift. However, the elimination likely is uncatalyzed and would in principle also be possible in AHCDC (**4**), which lends additional support to the proposed tautomerization mechanism derived above. Further, the finding that racemic *O*-ethylated DHHA (**20**, v_max_ = 3.61 ± 0.65 nmol s^−1^ mg^−1^ (k_cat_ = 0.12 ± 0.02 s^−1^) and K_M_ = 1842 ± 355 µM) was consumed much more slowly than racemic *O*-methylated DHHA (**19**; v_max_ = 41.20 ± 2.49 nmol s^−1^ mg^−1^ (k_cat_ = 1.33 ± 0.08 s^−1^) and K_M_ = 622 ± 56 µM) and *O*-*n*-propylated DHHA (**21**) was not turned over at all (Table S1) indicates that steric factors limit the substrate spectrum of PhzF. Indeed, positioning the *O*-methylated derivative into the active center of PhzF in the ligand-bound closed conformation (PDB entries 1U1W or 1U1X) already leads to clashes and when the inactive E45Q-mutant of PhzF was crystallized in the presence of racemic *O*-ethylated-DHHA (**20**), PhzF was found in the open form otherwise observed in the absence of specific ligands and have the inert (2 *R*,3 *R*)-enantiomer of *O*-ethylated DHHA (**20**) bound. The structure, refined at 1.71 Å resolution (R_work_ = 15.0%; R_free_ = 17.4%; Table S2), reveals that the ethyl group occupies the position otherwise taken by the water molecule that our analysis predicts to be involved in the tautomerization reaction (Figs 1B and S6).

## Discussion

Phenazine biosynthesis protein PhzF catalyzes two subsequent isomerization reactions, namely a [1,5]-proton shift from C3 to C1 of DHHA (**3**) to form the enol intermediate AHCDC (**4**), which is then tautomerized to AOCHC (**5**). Despite showing several hallmarks of a pericyclic reaction mechanism, QM/MM calculations make it unlikely that the initial isomerization to AHCDC (**4**) follows a pericyclic reaction path, at least with zwitterionic DHHA (**3**), which we identified as the prevailing ionization state of DHHA (**3**) in solution and in the enzyme/substrate complex. Yet, sigmatropic reactions are known to be strongly accelerated by charged functional groups in some cases^20^. We therefore also tested if introducing a negative charge into the substrate by deprotonating the 3-OH-group of DHHA (**3**) first (Fig. S7) would make a sigmatropic [1,5]-hydrogen shift possible. QM/MM calculations indeed reveal that the energy barrier decreases to 22 kcal/mol (vs. 35 kcal/mol for zwitterionic DHHA; **3**). However, this is still significantly higher than that of the proton shuttle mechanism involving E45 (16 kcal/mol). In addition, we could not find an energetically feasible path for the initial deprotonation of the 3-OH group of DHHA (**3**), and this charge-induced mechanism can also not explain why PhzF converts *O*-alkylated substrates **19** and **20** (Table S1). Therefore, a charge-induced sigmatropic rearrangement, though chemically attractive, can be ruled out for PhzF.

It is interesting to speculate why the sigmatropic [1,5]-hydrogen shift invokes such high activation barriers. Obviously, the strain induced in DHHA (**3**) to adopt a pericyclic transition state for concerted hydrogen transfer cannot be compensated by interactions with the enzyme. This may also be the reason why enzyme-catalyzed sigmatropic [1,5]-hydrogen shifts have not yet been discovered: while it is technically easy to activate the respective substrates *in vitro* by heating, an enzyme would probably have to establish several attractive interactions in the strained transition state exclusively, which is difficult to envision for relatively small molecules like cyclohexadienes. This sets the [1,5]-sigmatropic hydrogen shift apart from other sigmatropic reactions such as the Claisen rearrangement or the Diels-Alder reaction, where catalysis mainly involves correct positioning of the reacting groups. In line with this, we found that the calculated activation barrier of a sigmatropic [1,5]-hydrogen shift in DHHA (**3**) in water is almost identical to that within the active center of PhzF (35.7 vs. 35 kcal/mol for zwitterionic DHHA (**3**) and 21 vs. 22 kcal/mol after additional deprotonation of the 3-OH group), indicating that PhzF has no effect on the transition state of this mechanism.

Instead of a sigmatropic hydrogen shift, our calculations suggest that PhzF enables room-temperature isomerization of DHHA (**3**) to AHCDC (**4**) via acid/base catalysis mediated through the conserved glutamate residue E45, which acts as a shuttle that transfers a proton from C3 to C1 of the substrate. This also results in a suprafacial [1,5]-proton shift but not via a single transition state as in a sigmatropic reaction. To initiate substrate turnover, PhzF decreases the acidity of E45 in the course of substrate binding such that the proton transfer within DHHA (**3**) becomes feasible. The pK_a_-gap between DHHA (**3**) and E45 can be estimated by referring to the allylic proton of cyclopentadiene, which is known to have a pK_a_ of 16. Using the B3LYP functional with a def2-TZVP basis set and modeling the solvent water with a continuum model, we found the pK_a_ of the C3-proton in DHHA (**3**) to be approx. 2.5 units higher. Binding to the active site of PhzF increases the pK_a_ further, leading to a pK_a_ of 21.3. At the same time, the calculated pK_a_ of E45 is 9, yielding a gap of 12.3 pK_a_-units. This corresponds to 17 kcal/mol for the proton transfer, which is in good agreement with the energy barrier derived with the more sophisticated QM/MM-methods outlined above. Deprotonation at C3 is probably also facilitated by the dense network of hydrogen bonds and by dipolar contributions from the N-termini of two α-helices pointing towards the carboxylate of DHHA (**3**; Fig. 1B), enabling additional delocalization of the ensuing negative charge through this group.

The shuttle mechanism is sensitive to electronic alterations in the substrate, explaining why electron density maps of PhzF in complex with the inhibitor 3OHAA (**22**) provide indications for a protonated E45 side chain (Fig. 1B) and why DHHS (**18**), the 2-hydroxy isomer of DHHA (**3**), is not a substrate of PhzF. Here, the substitution increases the pK_a_ of E45 to 21 and that of the proton at C3 to 37.3. Therefore, not only does the deprotonation of both groups become more difficult but also the energy barrier for the proton transfer increases.

Monte Carlo titrations gave a pK_a_ value of 9 for E45 in the enzyme/substrate complex, suggesting that this residue needs to be deprotonated before catalysis can take place. We accounted for this finding by increasing the energy of all transition states by 1.7 kcal/mol, but a mechanism for an initial deprotonation of E45 is not obvious from the crystal structure. Indeed, the catalytic cycle of PhzF may not require such a deprotonation step: as mentioned above, the enzyme undergoes a structural transition from an open to a closed conformation on substrate binding (Fig. 1B) and Monte Carlo titrations for the open, non-liganded form calculate the pK_a_ of E45 as 5.5. Therefore, E45 likely is deprotonated when it binds DHHA (**3**) and adoption of the closed conformation then increases its pK_a_, while the structural transition at the same time restricts contact with water or other proton-donating groups, thereby avoiding protonation of E45 and enabling catalysis.

Despite the decreased pK_a_ gap between E45 and the C3 proton of the substrate, the activation barrier of the first isomerization reaction remains high, implying slow reaction kinetics and a large kinetic isotope effect, which were both confirmed by enzyme kinetic experiments. The apparent KIE on v_max_ was 9.5 in H_2_O. Comparing these apparent values to the calculated intrinsic KIE of just the deprotonation step is difficult due to the fact that the apparent KIEs contain contributions from the complete kinetic mechanism. However, given that the zero-point energy difference between hydrogen and deuterium limits the intrinsic primary KIE of a C-H-bond-breaking reaction to 7 and apparent KIEs are expected to be smaller than the intrinsic KIE of the affected step in complex enzyme catalytic cycle^21^, the magnitude of the values found here suggests that tunneling may play a role in the deprotonation step.

For the tautomerization in PhzF catalysis, we propose a reaction mechanism involving a tightly bound water and E45. This mechanism agrees with the stereospecific incorporation of a solvent-derived proton into the product and reveals that PhzF catalyzes two reactions.

The active site of PhzF in the closed, ligand-bound conformation is perfectly adapted to bind DHHA (**3**) and a single water molecule only. This is a prerequisite for the remarkable catalytic activity of PhzF, which combines an isomerization reaction that needs to proceed in the absence of water with a tautomerization that requires a water molecule to become feasible. It also explains why only short *O*-alkylated derivatives of DHHA (**19** and **20**) were isomerized by PhzF, which seems to impede using the enzyme in a broader range of biotransformations but may, together with PhzF’s indispensability for phenazine virulence factor biosynthesis, make it an attractive target for the development of pathogenicity blockers with high specificity.

## Methods

### Synthesis of DHHA (3), C3-deuterated DHHA (d-3) and O-alkylated DHHA derivatives (19 – 21)

DHHA-derivatives (**3**, **d-3**, **19**–**21**) were synthesized by modification of a route established by Steel and coworkers^14–17^. Briefly, this approach involves a Diels-Alder cycloaddition of furan (**9**) to esters of nitoacrylic acid^22–24^ (**12**) and ring-opening with KHMDS as key steps. Deuteration was achieved by starting with 2-bromofuran^25^ (**10**) and reductive dehalogenation with Zn/Cu and DCl in D_2_O. Diastereomers were separated by flash chromatography and enantiomerically pure compounds were generated by repetitive kinetic resolution with PLE (Fig. 2 and Supplemental information).

### NMR measurements

NMR measurements were performed at 15 °C in a Varian/Agilent Unity Inova 500-MHz NMR spectrometer (Varian, Inc., Palo Alto, California) with 10 mM DHHA (**3**) in 50 mM sodium phosphate in 9:1 H_2_O:D_2_O pH 7.5 or in D_2_O at pD 7.5. Turnover was initiated by addition of PhzF to a final concentration of 5 or 1.4 µM and ^1^H-NMR spectra were recorded after 5 minutes, applying transmitter presaturation of the residual H_2_O line. NOEs were measured after irradiation of the proton at C2 of AOCHC (**5**). Chemical shifts were predicted by hybrid DFT computations on a computing cluster with blade architecture using the Gaussian09 software package^26^ at the PCM(H_2_O) B3LYP/IGLO-II//PCM(H_2_O) B3LYP/6–31 + G* level of theory^27–30^.

### pK_a_ determination of DHHA (3)

75 ml of a 10 mM solution of racemic synthetic DHHA (**3**) were titrated with 100 mM NaOH while the pH of the solution was monitored. Titrations of glycine and malonic acid where used for validation of the method by comparing to published values (Fig. S1).

### Computational methods

The crystal structure of PhzF in complex with DHHA (**3**; PDB entry 1U1X)^4^ was used for all *in silico* calculations. The model was prepared by computing atomic charges and by adding and optimizing missing hydrogen atoms using ORCA^31^ and CHARMM^32^. For continuum electrostatic calculations, all except two critically important crystallographic water molecules (water molecule 407 in chain A and 417 in chain B) were replaced by a dielectric continuum with dielectric constants of 4 for the protein interior and 80 for the outside. For QM/MM computation, the titration state of side chains was chosen based on the previous electrostatic calculations. In addition to crystal water molecules, a 6 Å water layer was added and the position of all water molecules was minimized.

Protonation probabilities of titratable side chains and of DHHA (**3**) were determined with a Poisson-Boltzmann continuum model combined with a Metropolis Monte Carlo algorithm in the pH range from 0 to 14 using MEAD^33^ and GMCT^34^.

QM/MM calculations were performed with pDynamo^35^ combined with ORCA^31^, using the CHARMM27^36^ force field for the MM part and DFT methods BP86^37, 38^ and B3LYP^29^ with 6–31 G* and 6–31 G** basis sets^30^ for the QM part, similar to previous studies^39^. The QM model consisted of residues in the active center of chain A, namely DHHA (**3**; zwitterionic state) and the sidechains of E45, H74, D208, S213 and water molecule 407, while the rest of the protein was treated with MM methods and the external parts were restrained as described previously^39^. A first estimate for the reaction coordinate was obtained by potential energy surface scan (PES), followed by minimization. Promising pathways were then optimized by modified nudged elastic band (NEB)^40^ and conjugate peak refinement (CPR)^41^ with our own python implementation PyCPR^42^, using the BP86 functional as the QM method. Energetically feasible reaction paths were further refined using CPR and the B3LYP functional, which was also used for vibration frequency calculations. Energies were corrected by zero point energy contribution.

QM calculations of the uncatalyzed tautomerization in water were done with ORCA using the COSMO^43^ continuum solvation model and two explicit water molecules. B3LYP with 6–31 G** basis set was employed. Transition states were located using PES scan and eigenvector-following algorithm and the energies were corrected by zero point energy contribution.

pK_a_-values for C3 of DHHA (**3**) and DHHS (**18**) were predicted from the difference in energy of the protonated and deprotonated species and corrected by comparison to cyclopentadiene. Geometry optimizations and energy calculations were performed by ORCA using the COSMO continuum solvation model and the B3LYP functional with def2-TZVP basis set^44^.

### Protein Expression and Purification

PhzF from *Pseudomonas fluorescens* 2–79 was expressed and purified as described previously^45^. In brief, the protein (UniProt-entry Q51792 (PHZF_PSEFL)) was overexpressed as an N-terminally His_6_-tagged fusion in *E. coli* and purified by immobilized Ni^2+^-affinity chromatography followed by a size-exclusion step in assay (50 mM sodium phosphate pH 7.5) or crystallization buffer (20 mM TRIS/HCl pH 7.5, 150 mM NaCl) supplemented with 10% (v/v) glycerol. Purified protein was concentrated in the respective buffer, snap-frozen in liquid nitrogen and stored at −80 °C until further usage. Site-directed mutagenesis was achieved with the QuikChange method (Stratagene).

### Photometric assay

Enzyme kinetic parameters were determined in an Infinite® 200 microplate reader (Tecan Group AG, Männedorf, Switzerland) with UV-Star® 96-Well microplates (Greiner Bio-One International GmbH, Kremsmünster, Austria) at 25 °C. After determination of a suitable pH value (Fig. S3A), 40 nM PhzF dimer were added to obtain 100 µl assay solution consisting of 50 mM sodium phosphate pH 7.5, 1% (v/v) DMSO and up to 1 mM DHHA (**3**). Substrate depletion was followed at 275 nm for 20 minutes after reaching a linear phase (Fig. S3B), using an experimentally determined extinction coefficient of 6500 M^−1^ cm^−1^ for DHHA (**3**). The enzyme concentration was increased to 200 and 1000 nM to obtain measurable rates for C3-deuterated DHHA (**d-3**) and for racemic *O*-Et-DHHA (**20**), respectively. All experiments were performed in triplicate and enzyme kinetic parameters were derived by fitting to a Michaelis-Menten model in GraFit5 (Erithacus Software Ltd., Horley, UK).

### Crystallography

Crystallization conditions for PhzF in complex with *O*-ethylated DHHA were identified using the JCSG Core I–IV and The Cryos suites (Qiagen N.V., Venlo, Netherlands) in a sitting drop vapor diffusion setup at room temperature using the E45Q-mutant at 10 mg/ml pre-incubated with 10 mM *rac*-*O*-Et-DHHA (**20**). Drops consisted of 200 nl protein solution and 200 nl precipitant. Crystals appeared after 12 hours from a reservoir consisting of 0.085 M sodium acetate pH 4.6, 0.17 M ammonium acetate, 25.5% (w/v) PEG 4000 and 15% (v/v) glycerol. Diffraction data were collected at beamline MX BL14.1 at the BESSY-II synchrotron (Helmholtz Centre Berlin, Germany) and reduced with XDS^46^ followed by AIMLESS^47^ of the CCP4 package^48^. Refinement and model building were achieved by alternating rounds of manual adjustment in COOT^49^ and maximum likelihood refinement in phenix.refine^50^ of the PHENIX software suite^51^.

Data collection and refinement statistics are reported in Table S2. Diffraction data and coordinates of PhzF in complex with *O*-Et-DHHA (**20**) have been deposited in the Protein Data Bank^52^ with entry code 5IWE.

## Electronic supplementary material

Supplementary Information

Supplementary Movie 1

## References

[1] Okegbe C, Sakhtah H, Sekedat MD, Price-Whelan A, Dietrich LEP (2012). Redox eustress: roles for redox-active metabolites in bacterial signaling and behavior. Antioxid. Redox Signal..

[2] Guttenberger N, Blankenfeldt W, Breinbauer R (2017). Recent developments in the isolation, biological function, biosynthesis, and synthesis of phenazine natural products. Bioorg. Med. Chem..

[3] Parsons JF (2004). Structure and function of the phenazine biosynthesis protein PhzF from Pseudomonas fluorescens 2–79. Biochemistry.

[4] Blankenfeldt W (2004). Structure and function of the phenazine biosynthetic protein PhzF from Pseudomonas fluorescens. Proc. Natl. Acad. Sci. USA..

[5] Mentel M (2009). Of two make one: the biosynthesis of phenazines. Chembiochem Eur. J. Chem. Biol..

[6] Blankenfeldt W, Parsons JF (2014). The structural biology of phenazine biosynthesis. Curr. Opin. Struct. Biol..

[7] Ahuja EG (2008). PhzA/B catalyzes the formation of the tricycle in phenazine biosynthesis. J. Am. Chem. Soc..

[8] Xu N (2013). Trapped intermediates in crystals of the FMN-dependent oxidase PhzG provide insight into the final steps of phenazine biosynthesis. Acta Crystallogr. D Biol. Crystallogr..

[9] Burschowsky D (2014). Electrostatic transition state stabilization rather than reactant destabilization provides the chemical basis for efficient chorismate mutase catalysis. Proc. Natl. Acad. Sci. USA..

[10] DeClue MS, Baldridge KK, Künzler DE, Kast P, Hilvert D (2005). Isochorismate pyruvate lyase: a pericyclic reaction mechanism?. J. Am. Chem. Soc..

[11] Li, Y. *et al*. Biosynthesis of vitamin B12: mechanistic studies on the transfer of a methyl group from C-11 to C-12 and incorporation of 18O. *J. Chem. Soc. Chem. Commun*. 2507–2508 (1994).

[12] Ose T (2003). Insight into a natural Diels-Alder reaction from the structure of macrophomate synthase. Nature.

[13] Byrne MJ (2016). The Catalytic Mechanism of a Natural Diels-Alderase Revealed in Molecular Detail. J. Am. Chem. Soc..

[14] Bunnage ME, Ganesh T, Masesane IB, Orton D, Steel PG (2003). Asymmetric synthesis of the putative structure of (−)-oryzoxymycin. Org. Lett..

[15] Masesane IB, Steel PG (2004). Stereoselective routes to 3-hydroxy and 3,4-dihydroxy derivatives of 2-aminocyclohexanecarboxylic acid. Tetrahedron Lett..

[16] Masesane IB, Batsanov AS, Howard JAK, Mondal R, Steel PG (2006). The oxanorbornene approach to 3-hydroxy, 3,4-dihydroxy and 3,4,5-trihydroxy derivatives of 2-aminocyclohexanecarboxylic acid. Beilstein J. Org. Chem..

[17] Bwire RN, Majinda RR, Masesane IB, Steel PG (2009). From nature, through chemical synthesis, toward use in agriculture: Oryzoxymycin case study. Pure Appl. Chem..

[18] Lewars, E. G. *Computational Chemistry*. (Springer Netherlands, 2011).

[19] Liu F (2015). Elucidation of Enzymatic Mechanism of Phenazine Biosynthetic Protein PhzF Using QM/MM and MD Simulations. PloS One.

[20] Evans DA, Golob AM (1975). [3,3]Sigmatropic rearrangements of 1,5-diene alkoxides. Powerful accelerating effects of the alkoxide substituent. J. Am. Chem. Soc..

[21] Northrop DB (1975). Steady-state analysis of kinetic isotope effects in enzymic reactions. Biochemistry.

[22] Ballini R, Fiorini D, Palmieri A (2004). Nitroalkanes and ethyl glyoxalate as common precursors for the preparation of both β-keto esters and α,β-unsaturated esters. Tetrahedron Lett..

[23] Addo JK, Teesdale-Spittle P, Hoberg JO (2005). Synthesis of 3-Nitropropanol Homologues. Synthesis.

[24] Mukherjee S, Corey EJ (2010). [4 + 2] Cycloaddition Reactions Catalyzed by a Chiral Oxazaborolidinium Cation. Reaction Rates and Diastereo-, Regio-, and Enantioselectivity Depend on Whether Both Bonds Are Formed Simultaneously. Org. Lett..

[25] Raheem M-A, Nagireddy JR, Durham R, Tam W (2010). Efficient procedure for the preparation of 2-bromofuran and its application in the synthesis of 2-arylfurans. Synth. Commun..

[26] Frisch, M. J. *et al*. Gaussian09 Revision E.01.

[27] Tomasi J, Mennucci B, Cammi R (2005). Quantum mechanical continuum solvation models. Chem. Rev..

[28] Kutzelnigg, W., Fleischer, U. & Schindler, M. In *Deuterium and Shift Calculation* 165–262 (Springer Berlin Heidelberg, 1990).

[29] Becke AD (1993). Density‐functional thermochemistry. III. The role of exact exchange. J. Chem. Phys..

[30] Hehre WJ, Ditchfield R, Pople JA (1972). Self—Consistent Molecular Orbital Methods. XII. Further Extensions of Gaussian—Type Basis Sets for Use in Molecular Orbital Studies of Organic Molecules. J. Chem. Phys..

[31] Neese F (2012). The ORCA program system. Wiley Interdiscip. Rev. Comput. Mol. Sci..

[32] Brooks BR (2009). CHARMM: the biomolecular simulation program. J. Comput. Chem..

[33] Bashford D, Gerwert K (1992). Electrostatic calculations of the pKa values of ionizable groups in bacteriorhodopsin. J. Mol. Biol..

[34] Ullmann RT, Ullmann GM (2012). GMCT: a Monte Carlo simulation package for macromolecular receptors. J. Comput. Chem..

[35] Field MJ (2008). The pDynamo Program for Molecular Simulations using Hybrid Quantum Chemical and Molecular Mechanical Potentials. J. Chem. Theory Comput..

[36] MacKerell AD (1998). All-atom empirical potential for molecular modeling and dynamics studies of proteins. J. Phys. Chem. B.

[37] Becke AD (1988). Density-functional exchange-energy approximation with correct asymptotic behavior. Phys. Rev. A.

[38] Perdew JP (1986). Density-functional approximation for the correlation energy of the inhomogeneous electron gas. Phys. Rev. B Condens. Matter.

[39] Feliks M, Martins BM, Ullmann GM (2013). Catalytic mechanism of the glycyl radical enzyme 4-hydroxyphenylacetate decarboxylase from continuum electrostatic and QC/MM calculations. J. Am. Chem. Soc..

[40] Aleksandrov A, Field M (2012). A hybrid elastic band string algorithm for studies of enzymatic reactions. Phys. Chem. Chem. Phys. PCCP.

[41] Fischer S, Karplus M (1992). Conjugate peak refinement: an algorithm for finding reaction paths and accurate transition states in systems with many degrees of freedom. Chem. Phys. Lett..

[42] Gisdon FJ, Culka M, Ullmann GM (2016). PyCPR - a python-based implementation of the Conjugate Peak Refinement (CPR) algorithm for finding transition state structures. J. Mol. Model..

[43] Sinnecker S, Rajendran A, Klamt A, Diedenhofen M, Neese F (2006). Calculation of Solvent Shifts on Electronic g-Tensors with the Conductor-Like Screening Model (COSMO) and Its Self-Consistent Generalization to Real Solvents (Direct COSMO-RS). J. Phys. Chem. A.

[44] Weigend F, Ahlrichs R (2005). Balanced basis sets of split valence, triple zeta valence and quadruple zeta valence quality for H to Rn: Design and assessment of accuracy. Phys. Chem. Chem. Phys. PCCP.

[45] Mavrodi DV, Bleimling N, Thomashow LS, Blankenfeldt W (2004). The purification, crystallization and preliminary structural characterization of PhzF, a key enzyme in the phenazine-biosynthesis pathway from Pseudomonas fluorescens 2–79. Acta Crystallogr. D Biol. Crystallogr..

[46] Kabsch, W. XDS. *Acta Crystallogr. D Biol. Crystallogr*. **66**, 125–132 (2010). 10.1107/S0907444909047337 PMC2815665 20124692

[47] Evans PR, Murshudov GN (2013). How good are my data and what is the resolution?. Acta Crystallogr. D Biol. Crystallogr..

[48] Winn MD (2011). Overview of the CCP4 suite and current developments. Acta Crystallogr. D Biol. Crystallogr..

[49] Emsley P, Lohkamp B, Scott WG, Cowtan K (2010). Features and development of Coot. Acta Crystallogr. D Biol. Crystallogr..

[50] Afonine PV (2012). Towards automated crystallographic structure refinement with phenix.refine. Acta Crystallogr. D Biol. Crystallogr..

[51] Adams PD (2010). PHENIX: a comprehensive Python-based system for macromolecular structure solution. Acta Crystallogr. D Biol. Crystallogr..

[52] Berman HM (2000). The Protein Data Bank. Nucleic Acids Res..

[53] Schrödinger, L. The PyMOL Molecular Graphics System (2010).

