# Supplementary material for: Mechanisms and Specificity of Phenazine Biosynthesis Protein PhzF
Source: Sci Rep. 2017 Jul 24;7:6272. doi: 10.1038/s41598-017-06278-w (PMC5524880; doi:10.1038/s41598-017-06278-w)
Supplement: Supplementary file 1 — Supplementary Information [file 41598_2017_6278_MOESM1_ESM.pdf]

Supplemental Information

Mechanisms and Specificity of Phenazine Biosynthesis Protein PhzF

Christina Diederich[1,5,6], Mario Leypold[2,6], Martin Culka[3,6], Hansjörg Weber[2], Rolf Breinbauer[2,*], G. Matthias Ullmann[3,*] & Wulf Blankenfeldt[1,4,5,*]

[1]Structure and Function of Proteins, Helmholtz Centre for Infection Research, Inhoffenstr. 7, 38124 Braunschweig, Germany

[2]Institute of Organic Chemistry, Graz University of Technology, Stremayrgasse 9, 8010 Graz, Austria

[3]Structural Biology/Bioinformatics, University of Bayreuth, Universitätsstr. 30, 95447 Bayreuth, Germany

[4]Institute for Biochemistry, Biotechnology and Bioinformatics, Technische Universität Braunschweig, Spielmannstr. 7, 38106 Braunschweig, Germany

[5]previous address: Department of Biochemistry, University of Bayreuth, Universitätsstr. 30, 95447 Bayreuth, Germany

[6]These authors contributed equally.

Table of contents

	pages
Supplemental Figures	S2 – S5
Supplemental Tables	S6 – S7
Full Experimental Methods	
Computational methods	S8 – S9
Protein biochemical and crystallographic methods	S10
Synthetic methods	S11 - S46
Spectroscopic data of synthetic compounds	S47 - S79
Supplemental References	S80 - S81

Supplemental Figures

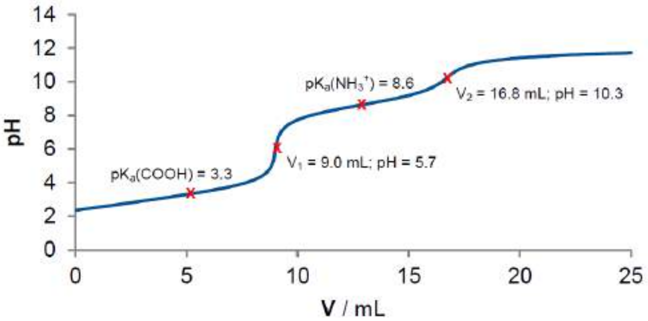

Figure S1. pK_a determination of DHHA. 75 ml of 10 mM racemic synthetic DHHA were titrated with 100 mM NaOH and the pH was monitored. The method was validated by titrating L-glycine and malonic acid and comparing to pK_a values reported in the literature. Differences to these values were used as standard errors for the pK_a values of DHHA.

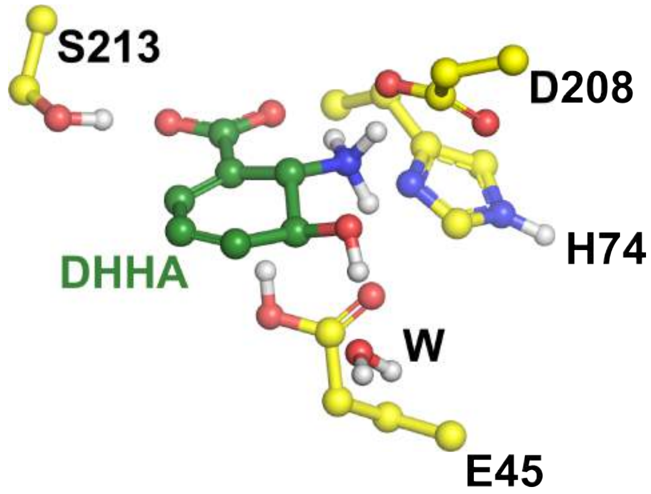

Figure S2. Protonation of residues in the active site of PhzF in complex with DHHA at pH 7.5. Protonations have been predicted by Monte Carlo titration within the continuum electrostatic model. The residues shown here also represent the QM region of QM/MM calculations in this work. W: the only tightly bound water molecule in the active site of PhzF in the closed, ligand-bound conformation (W407 in PDB entry 1U1X).

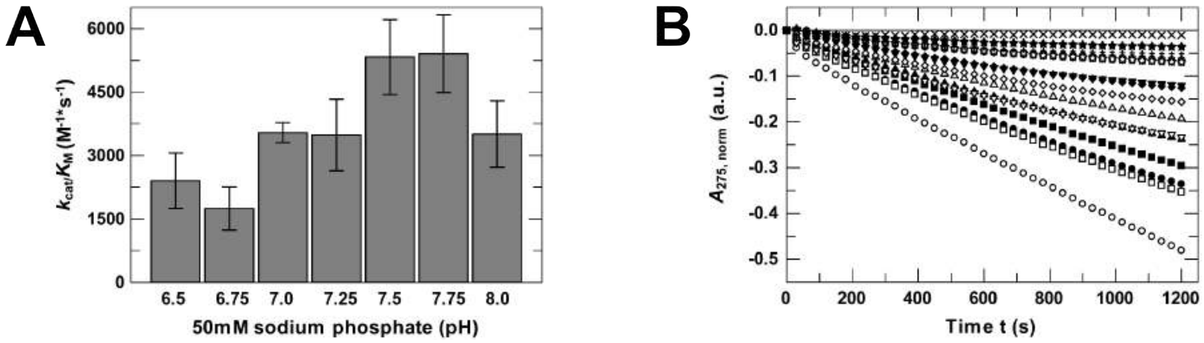

Figure S3. Development of a photometric assay for PhzF based on depletion of DHHA. (A) Determination of an optimal pH, based on k_{cat}/K_M. (B) Time traces obtained at different concentrations of DHHA (○: 1 mM DHHA; x: 0 mM DHHA; OD_{275} was measured at pH 7.5).

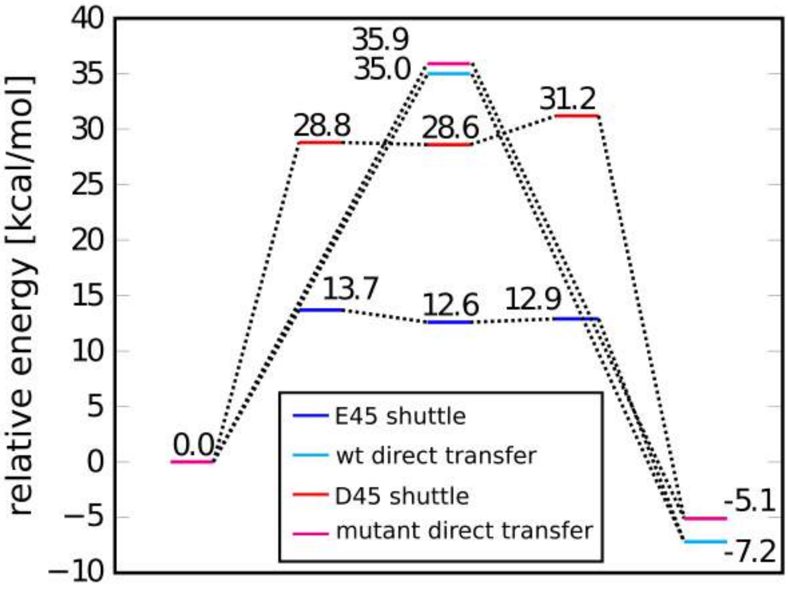

Figure S4. Calculated energy profiles for the sigmatropic [1,5]-hydrogen shift and the proton shuttle mechanism (see Fig. 4, A, i & iii) in the isomerization of DHHA to AHCDC by wildtype PhzF and by its E45D mutant. Energy values have been calculated with the computationally less expensive BP86: 6-31G* method and therefore deviate from wildtype values given in the main text. An in-silico E45D mutant was used as a structural template for the calculations presented in this figure.
The high activation barriers for the E45D mutant explain its inactivity.

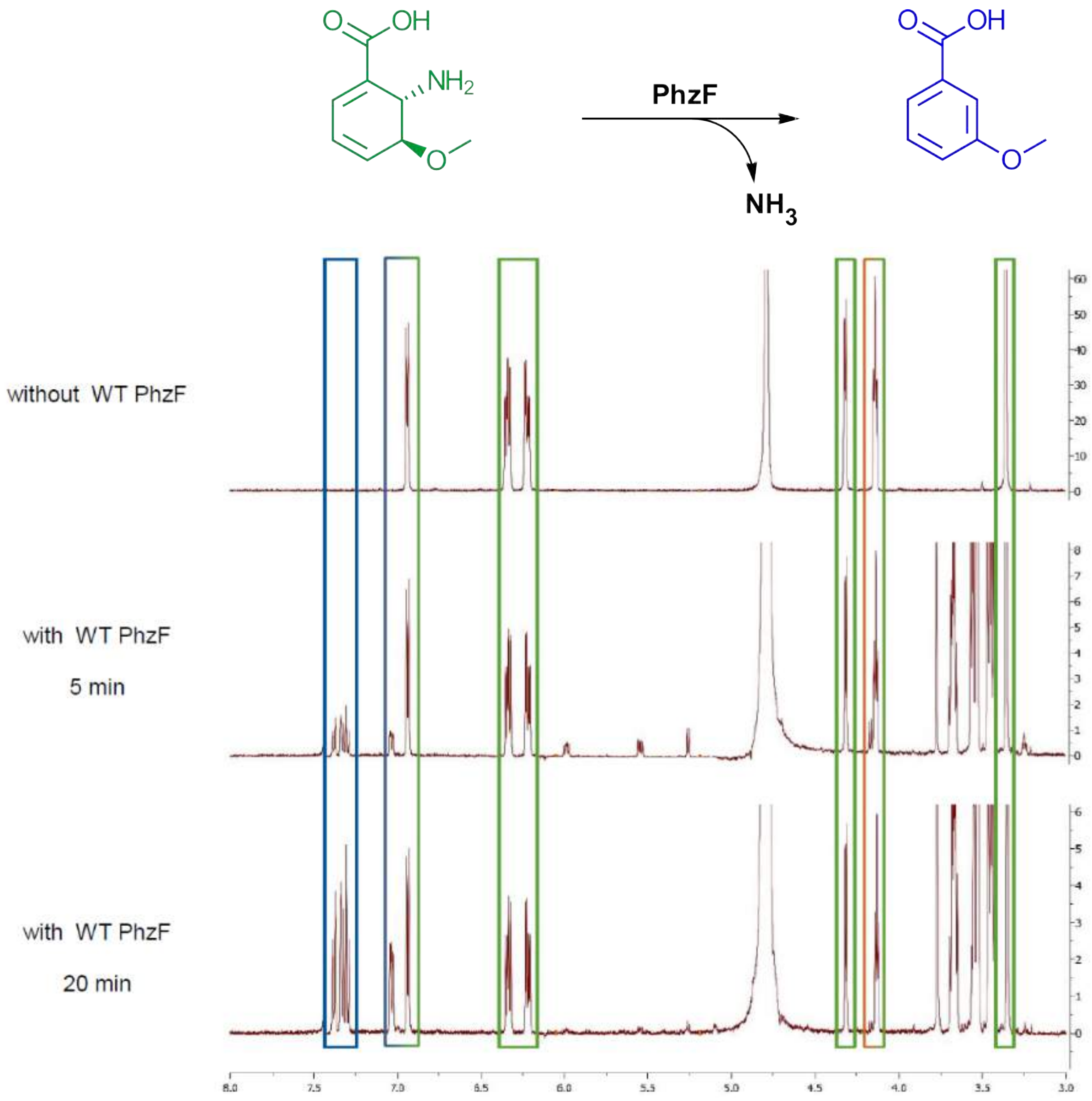

Figure S5. ^{1}H-NMR spectra of PhzF-mediated turnover of *rac-O*-Me-DHHA. Spectra were measured at the indicated times after addition of the enzyme.

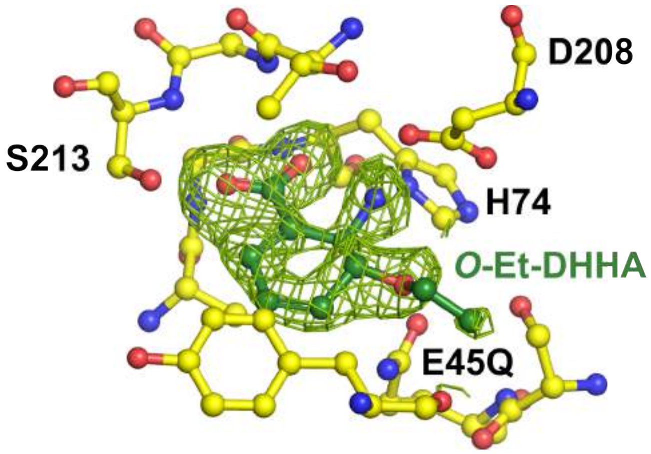

Figure S6. $|F_O-F_C|$ OMIT difference electron density of *O*-Et-DHHA (PDB entry 5IWE). The density is displayed at 2 σ.

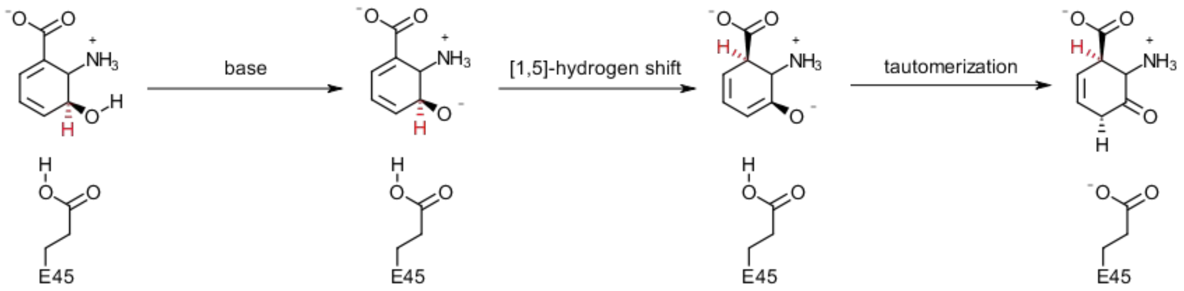

Figure S7. Hypothetical mechanism of a charge-induced sigmatropic [1,5]-hydrogen shift in PhzF. The activation barrier for the [1,5]-hydrogen shift was found at 22 kcal/mol in QM/MM calculations and no feasible mechanism for the initial deprotonation of the 3-hydroxy group could be identified.

Supplemental Tables

Table S1. Enzyme kinetic parameters.

substrate	v_{max} (nmol s^{-1} mg^{-1})		k_{cat} (s^{-1})		K_M (μM)		k_{cat}/K_M ($M^{-1}s^{-1}$)	
DHHA	100.34	± 5.80	3.23	± 0.19	517	± 57	6251	± 773
d-DHHA	10.60	± 0.27	0.34	± 0.01	311	± 14	1099	± 58
rac-O-Me-DHHA	41.20	± 2.49	1.33	± 0.08	622	± 56	2134	± 231
rac-O-Et-DHHA	3.61	± 0.65	0.12	± 0.02	1842	± 355	63	± 17

Table S2. Data collection & refinement statistics for PhzF in complex with *O*-ethyl-DHHA .

	Complex with *O*-ethyl DHHA
**Data collection**[1]	
Beamline[†]	MX BL14.1, BESSY II
Wavelength (Å)	0.91841
Resolution range (Å)	77.87 – 1.71 (1.74 – 1. 71)
Space group	$P3_221$
unit cell dimensions	
A, *b*, *c* (Å)	56.03, 56.03, 155.74
α, β, γ (°)	90, 90, 120
Mosaicity[‡] (°)	0.07
Total No. of measured reflections	164740 (7396)
Unique reflections	31593 (1662)
Multiplicity	5.2 (4.5)
Mean $I/\sigma(I)$	8.9 (1.9)
$CC_{1/2}$ *	0.995 (0.573)
Completeness (%)	99.9 (99.9)
R_{meas}[§] (%)	13.8 (86.2)
R_{pim}[¶] (%)	6.0 (40.8)
**Refinement**	
Resolution range (Å)	46.33 – 1.71 (1.76 – 1.71)
R_{cryst} (%)	14.9 (23.8)
R_{free} (%)	17.3 (27.2)
No. of non H-atoms	
Protein	2170
Ligand / Ion	47
Water	242
Average *B* factors (Å²)	
Protein	16
Ligand / Ion	35
Water	34
R.m.s deviations	
Bond lengths (Å)	0.007
Bond angles (°)	0.945
Ramachandran plot (%)	
Favoured regions	98.6
Outliers	0
MolProbity score #	1.21
PDB entry	5IWE

[1] The data set was collected from a single crystal.
[†] BESSY II: Berlin Electron Storage Ring Society for Synchrotron Radiation, Helmholtz Centre Berlin, Berlin, Germany.
[‡] Mosaicity values reported by XDS[1].
* $CC_{1/2}$ is the correlation coefficient between two random half data sets[2].
[§] $R_{meas} = \sum_{hkl}\{N(hkl)/[N(hkl)-1]\}^{1/2}\sum_i|I_i(hkl) - \langle I(hkl)\rangle| \,/\, \sum_{hkl}\sum_i I_i\,(hkl)$, where *N(hkl)* is the number of observations of the reflection with index *hkl* and $I_i(hkl)$ is the intensity of its *i*th observation[3].
[¶] $R_{p.i.m.} = \sum_{hkl}\{1/[N(hkl)-1]\}^{1/2}\sum_i|I_i(hkl) - \langle I(hkl)\rangle|/\sum_{hkl}\sum_i I_i(hkl)$[4].
[#] As reported by MolProbity (http://molprobity.biochem.duke.edu/)[5].

Full Experimental Methods

Computational methods

Structure preparation

Coordinates of the X-ray structure of the PhzF dimer in complex with 2,3-dihydro-3-hydroxy-anthranilic acid (DHHA) bound in the active site of both subunits (PDB code 1U1X)[6] were used as a basis for all theoretical studies. Electrostatic calculations were done on the whole homodimer, while only subunit A was used for the subsequent hybrid quantum mechanics/ molecular mechanics (QM/MM) studies.

The structure preparation was done within the CHARMM program[7], using parameters from the CHARMM27 force field[8]. The atomic charges for DHHA and its variants were taken from a CHELPG fit of the quantum-mechanical electrostatic potential derived from gas phase calculation in ORCA (B3LYP with TZVP basis set)[9] and the parameters were assigned in analogy to existing compounds in the force field. The HBUILD routine was used for adding the missing hydrogen atoms and their positions were subsequently optimized while keeping the heavy atoms fixed. For the continuum electrostatic calculations, the protonation of all titratable side chains was set to the standard values at pH 7. Afterwards, crystal water molecules were removed to be replaced by a dielectric continuum (see below). Critical active site crystal waters 407 (chain A) and 417 (chain B) were treated explicitly including lone pairs and rotamers apart from the substrate-free calculation. For QM/MM calculations, titration states of amino acid side chains were chosen based on the previous electrostatic calculations. In addition to the crystal water molecules, a 6 Å layer was added around the protein. The position of the water molecules was minimized keeping the protein atoms fixed.

Protonation probability determination

The protonation probabilities of the titratable amino acid side chains and of DHHA were determined within the Poisson-Boltzmann continuum electrostatic model combined with Monte Carlo titration. The electrostatic calculations and Monte Carlo titrations were carried out using MEAD[10] and GMCT[11], respectively. The following parameters were used for the Poisson-Boltzmann model: the dielectric constant of the protein interior was set to 4, the solvent was modeled with dielectricity of 80 and an ionic strength of 0.15M. The protonation probability for each titratable residue was calculated by the Metropolis Monte Carlo algorithm as a function of pH in a range of 0 to 14 in 0.25 pH steps. For every pH step, 500 equilibration and 10000 production scans were carried out at T = 300 K.

For AHCDC and AOCHC as well as DHHS, only the zwitterionic forms were considered.

Prediction of pK_a differences

All QM calculations were done using ORCA[9] with the B3LYP[12] DFT functional and the def2-TZVP[13] basis set in the environment of the COSMO[14] continuum solvent emulation. For all compounds (DHHA, DHHS, cyclopentandiene), both normal and carbon-deprotonated structures were subjected to geometry optimization. The energy difference between protonated and deprotonated cyclopentadiene was substracted from corresponding energy differences for DHHA and DHHS. The result was converted to pK_a units (assuming a temperature of 298 K) and added to the tabulated value for cyclopentandiene (pK_a = 16), giving pK_a values for C3-deprotonation of DHHA and DHHS in water. In order to predict the impact of the protein environment on the C3 pK_a value of DHHA and DHHS, the calculated solution pK_as were used as model pK_a values for Monte Carlo titrations in the Poisson-Boltzmann continuum solvent. In addition, partial atomic charges obtained from a CHELPG fit in ORCA for both protonated and deprotonated species were utilized. The pK_a predictions inside the protein were performed as described above.

QM/MM mechanism search

All QM/MM calculations were carried out using the pDynamo library[15] with the quantum chemical part calculated in ORCA[9]. The CHARMM27 force field was used for the MM part,

while DFT methods BP86 for broader investigation[16,17] and B3LYP for final path refinement[12] with 6-31G* basis set and 6-31G** basis sets[18] were used for the QM part. QM/MM calculations were performed using electrostatic embedding and link atoms as implemented in the pDynamo library were introduced to saturate the valence at the QM/MM boundary.
The QM-treated active site model comprised of the substrate DHHA (zwitterionic form) and side chains of neighboring polar aminoacids, namely E45 (both protonated and deprotonated version), H74, D208, S213 and a crystal water molecule 407. The rest of the protein and the surface water layer were present in the MM region. Harmonic restraints were applied on MM atoms beyond 8 Å from any QM atom, i.e. the QM region was surrounded by 8 Å of flexible MM layer, between 8 – 16 Å the force constants of the restraints linearly increased from 0 to 12 kcal/mol and beyond 16 Å the restraints were set to maximal force constant 12 kcal/mol. The initial reaction state was derived from the CHARMM-hydrogenated crystal coordinates by geometry optimization. The potential energy surface scans followed by geometry optimizations were then used for fundamental investigation of possible reaction pathways. In a typical scan, a hydrogen atom was moved in discrete 0.2 Å steps breaking a bond to its donor and subsequently forming a new bond with its acceptor. After each step, the position of the hydrogen in question was fixed, while the rest of the QM/MM model was optimized. After moving the hydrogen to a typical bond distance from the acceptor, the geometry was minimized without restraints applied on the hydrogen in question. An RMS gradient of less than 0.01 kcal/(mol * Å) was used as a minimization convergence criterion. The promising scan-derived pathways were refined by a modified Nudge Elastic Band (NEB) method[19] and the Conjugate Peak Refinement (CPR) method[20]. Alternatively, CPR was also used to find the reaction pathway between two stable states (eg. adduct and intermediate state) *de novo*. The procedure described above was carried out using the BP86 DFT functional with as the QM method. Since BP86 is known to underestimate the energies while producing reasonable geometries, energetically feasible pathways were further optimized using CPR with the computationally more demanding hybrid B3LYP as QM method. Vibration frequency calculations were performed on the B3LYP level as well. To make the frequency calculations computationally feasible, the MM region was kept completely fixed. Vibration frequencies were used to verify the minima and transition states of the final reaction coordinate and furthermore for the calculation of zero point energy correction. The kinetic isotope effect of the initial reaction step was calculated from the difference in vibrational frequencies between normal and C3-deuterated variant of DHHA and the first transition state[21].

Protein biochemical and crystallographic methods

Protein expression and purification

PhzF from *Pseudomonas fluorescens* 2-79 was expressed and purified as described previously[22]. In brief, the protein (UniProt-entry: Q51792 (PHZF_PSEFL)) was cloned into a pET15b expression vector (Novagen) and overexpressed in *E. coli* Rosetta (DE3) pLysS (Novagen) in LB-(Luria Bertani) medium after induction with 0.5 mM Isopropyl β-D-1-thiogalactopyranoside (IPTG) at an OD_{600} of 0.6 at 20 °C over night. Purification of the N-terminal hexahistidine-tagged protein followed a standard two-step procedure starting from immobilized Ni^{2+}-affinity chromatography followed by a size-exclusion step in assay (50 mM sodium phosphate pH 7.5) or crystallization buffer (20 mM TRIS/HCl pH7.5, 150 mM NaCl) supplemented with 10% (v/v) glycerol. Purified protein was concentrated in the respective buffer, snap-frozen in liquid nitrogen and stored at -80 °C until further usage. Site-directed mutagenesis was performed with the QuikChange protocol (Stratagene).

Enzyme assay

Enzyme kinetic parameters were determined in an Infinite® 200 microplate reader (Tecan Group Ltd.) with UV-Star® 96-Well microplates (Greiner Bio-One) at 25°C. After determination of a suitable pH value (Figure S3A), the assay was performed in 50 mM sodium phosphate pH 7.5, 1% (v/v) DMSO using up to 1 mM DHHA in a final volume of 100 µL and the reaction initiated by the addition of 40 nM PhzF dimer. Substrate depletion was followed at 275 nm for 20 minutes after reaching a linear phase, using an experimentally determined extinction coefficient of 6500 M^{-1} cm^{-1} for DHHA. Typical UV-time traces are shown in Figure S3B. The enzyme concentration was increased to 200 and 1000 nM to obtain measurable rates for *2S,3S*-3-deutero-DHHA and racemic *O*-Et-DHHA, respectively. All experiments were performed in triplicate and rate constants were derived by fitting to a Michaelis-Menten model in GraFit5 (Erithacus Software Ltd.).
Enzyme kinetic parameters are listed in Table S1.

Crystallography

New crystallization conditions for PhzF in complex with *O*-ethyl DHHA were identified using the commercial JCSG+ and The Cryos suites (Qiagen) in a sitting drop vapor diffusion setup at room temperature with 10 mg/ml of the E45Q-mutant pre-incubated with 10 mM *rac*-*O*-Et-DHHA. Drops consisted of 200 nl protein solution and 200 nl precipitant. Initial crystals were obtained after 12 hours from a reservoir comprised of 0.085 M sodium acetate pH 4.6, 0.17 M ammonium acetate, 25.5% (w/v) PEG 4000 and 15% (v/v) glycerol. Diamond-shaped crystals with maximum dimensions of 0.3 * 0.3 * 0.25 µm grew within a week, were then mounted on a nylon loop and flash frozen in liquid nitrogen. Diffraction data were collected at 100 K at beamline MX BL14.1, BESSY-II at Helmholtz-Zentrum Berlin (Berlin, Germany). Data reduction was carried out with XDS[1] followed by AIMLESS[23] of the CCP4 package[24]. Refinement and model building were achieved by alternating rounds of manual adjustment in COOT[25] and maximum likelihood refinement in phenix.refine[26] of the PHENIX software suite[27]. Ligand restraints were generated using the program phenix.elbow[28] of the latter software suite. With respect to the apparent resolution of 1.7 Å structural flexibility was modelled using TLS (Translation/Libration/Screw) refinement[29]. 8 TLS groups were identified by the program phenix.find_tls_groups, implemented in phenix.refine. In the last step of refinement, non-protein residues (water and ligands) were attributed to their nearest TLS group using a script developed in our group (Reichelt & Blankenfeldt, unpublished).
Final structure validation was done with MolProbity[5]. Diffraction data and coordinates have been deposited in the Protein Data Bank[30] (PDB entry 5IWE for the complex with O-Et-DHHA). Full data collection and refinement statistics are shown in Table S2.

Synthetic methods

Reagents and analytical methods for the synthesis of DHHA and DHHA derivatives
Reagents and solvents were purchased from Sigma-Aldrich, Alfa, Aesar, ABCR, Fisher Scientific, Acros Organics, Roth or VWR and dried and/or degassed as required.

Pig liver esterase (PLE) was purchased from Fluka as a technical precipitate in a half saturated $(NH_4)_2SO_4$ solution. 100 mM NaH_2PO_4/Na_2HPO_4 buffer at pH 7.6 was added to provide a constant pH.

Analytical thin layer chromatography (TLC) was carried out on Merck TLC silica gel aluminium sheets (silica gel 60, F254, 20 x 20 cm). Flash column chromatography was performed on silica gel 60 from Acros Organics (particle size 35 - 70 µm).

Enantiomeric excess was determined at 210 nm after liquid chromatography on an Agilent 1100 Series HPLC with a chiral Daicel Chemical Industries Chiralpak® AD-H column (4.6 x 250 mm, 5.0 µm) run isocratically with n-heptane:EtOH = 85:15 +/- 0.01 % TFA (v/v/v) at 1 ml/min, 15 °C, 65 bar. Some compounds (**1a**, **5a**, **34a** and **41a**) were derivatized with Marfey's reagent ((*S*)-2-((5-fluoro-2,4-dinitrophenyl)amino)propanamide) to reduce their polarity prior to HPLC analysis.

^{1}H-, ^{13}C and ^{19}F-NMR spectra were recorded on a Bruker AVANCE III 300 or a Varian/Agilent Unity Inova 500 spectrometer. Chemical shifts were referenced to tetramethylsilane as internal standard or to the residual proton and carbon signal of deuterated solvents, which were purchased from euriso-top®. $CDCl_3$ was neutralized by filtering through activated aluminium oxide, basic type 5016A, 58 Å, particle size: 150 mesh, Brockmann Grade I from Acros Organics.

HRMS spectra were recorded on a Waters Micromass GCT Premier system either directly or after gas-chromatographic ionization on a Hewlett Packard BC 7890A system, using electron impact (EI) for ionization. Molecule ions were analyzed by a time-of-flight mass analyzer in the positive mode.

Melting points were determined on a Mel-Temp melting point apparatus from Electrothermal, specific optical rotations were measured at 589 nm on a Perkin Elmer Polarimeter 341.

Synthesis of DHHA (9)

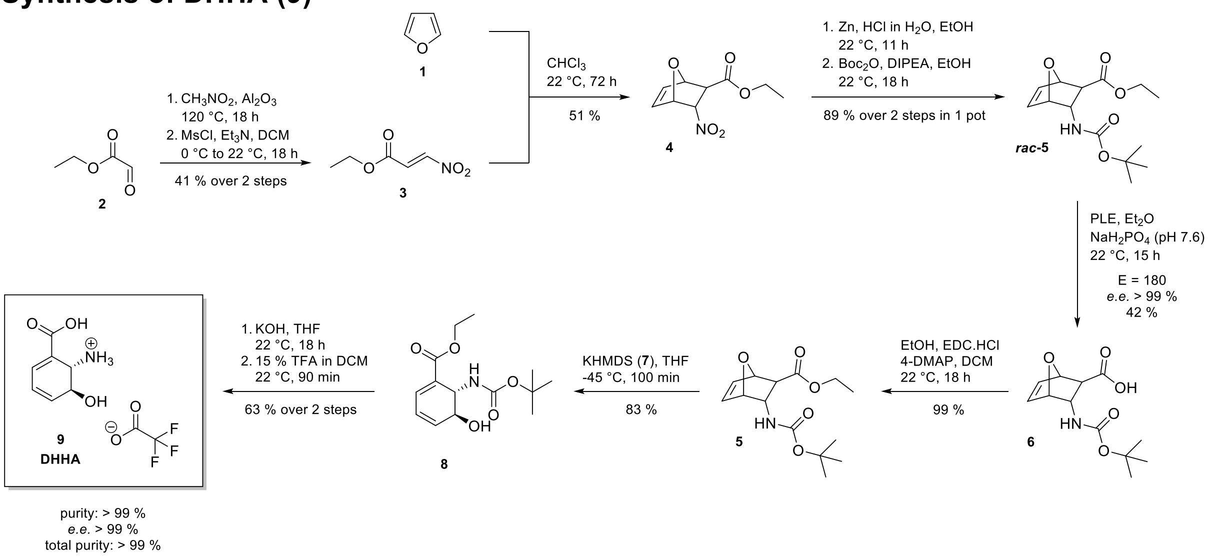

Enantioselective synthesis of DHHA in form of a TFA salt **9** in analogy to the published synthesis of Steel[31].

Ethyl 2-hydroxy-3-nitropropanoate (10)[32]

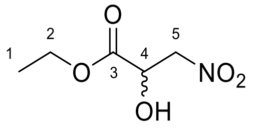

10

An oven-dried 100 mL two-necked round-bottom flask equipped with a Teflon-coated magnetic stirring bar, reflux condenser and a $CaCl_2$ drying tube was charged with a colorless solution of 15.0 g (73.5 mmol, 1.0 eq) ethyl glyoxalate (~50 % (w/w) in toluene) in 35 mL nitromethane. 15.0 g Al_2O_3 (neutral, activated, Brockmann Grade I) were added and the resulting yellowish suspension was heated under reflux in an oil bath at 120 °C for 18 h. Afterwards the resulting orange suspension was cooled down to RT, filtered through a pad of Celite® (diameter: 8.0 cm, height: 4.0 cm) and the filter cake was washed with EtOAc (4 x 30 mL). The solvent of the orange solution was evaporated on a rotary evaporator and the brownish, oily crude material was purified via flash column chromatography (520 g SiO_2, 22.0 x 8.0 cm, eluent: cyclohexane:EtOAc = 2:1 (v/v), R_f = 0.26). Finally, the light orange, needle shaped crystals were dried under high vacuum.

Yield: 7.78 g (48.2 mmol, 66 %); light orange, needle shaped crystals.

$C_5H_9NO_5$ [163.13 g/mol].

R_f = 0.26 (cyclohexane:EtOAc = 2:1 (v/v)).

m_p = 41-42 °C.

^{1}H-NMR (300.36 MHz, $CDCl_3$): δ = 4.77 (d, $^3J_{HH}$ = 4.2 Hz, 2H, H-5), 4.63 (q, $^3J_{HH}$ = 4.3 Hz, 1H, H-4), 4.40-4.29 (m, 2H, H-2), 3.40 (d, $^3J_{HH}$ = 4.7 Hz, 1H, OH), 1.33 (t, $^3J_{HH}$ = 7.2 Hz, 3H, H-1).

^{13}C-NMR (75.53 MHz, $CDCl_3$): δ = 170.8 (C_q, C-3), 76.9 (C-5), 67.7 (C-4), 63.3 (C-2), 14.2 (C-1).

Ethyl (*E*)-3-nitroacrylate (3)

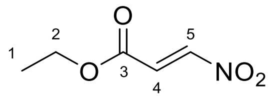

3

An oven-dried, evacuated and argon purged 500 mL three-necked round-bottom flask equipped with a Teflon-coated magnetic stirring bar and gas inlet adapter was charged with a yellowish solution of 8.00 g (49.0 mmol, 1.0 eq) ethyl 2-hydroxy-3-nitropropanoate (**10**) in 100 mL anhydrous DCM and cooled in an acetone/dry ice bath to -20 °C. Consecutively, 11.4 mL (147 mmol, 3.0 eq) methanesulfonyl chloride (MsCl) and 20.9 mL (147 mmol, 3.0 eq) Et_3N were added via a syringe and septum over a period of 15 min each and the brownish suspension was vigorously stirred at -20 °C for additional 3 h. Afterwards the suspension was poured into 450 mL ice-cooled H_2O, warmed to ambient temperature and stirred for 15 min. The phases were separated and the yellow aqueous phase was extracted with DCM (3 x 100 mL). The combined brownish organic layers were washed with H_2O (3 x 150 mL) and brine (1 x 150 mL), dried over $MgSO_4$, filtered and the solvent was removed under reduced pressure on a rotary evaporator. Finally the brownish, liquid crude material was purified by fractional distillation (38-39 °C, 0.52 mbar) and the residual MsCl was carefully removed by drying the intensively yellow colored liquid at 1.0 mbar at RT for 15 h.

Yield: 4.34 g (29.9 mmol, 61 %); yellow liquid.

$C_5H_7NO_4$ [145.11 g/mol].

R_f = 0.26 (cyclohexane:EtOAc = 12:1 (v/v)).

b_p = 38-39 °C (0.52 mbar).

^{1}H-NMR (300.36 MHz, $CDCl_3$): δ = 7.67 (d, $^3J_{HH}$ = 13.5 Hz, 1H, H-5), 7.08 (d, $^3J_{HH}$ = 13.5 Hz, 1H, H-4), 4.32 (q, $^3J_{HH}$ = 7.1 Hz, 2H, H-2), 1.34 (t, $^3J_{HH}$ = 7.1 Hz, 3H, H-1).

^{13}C-NMR (75.53 MHz, $CDCl_3$): δ = 162.8 (C_q, C-3), 149.1 (C-5), 127.8 (C-4), 62.6 (C-2), 14.2 (C-1).

Ethyl (1*R*,2*S*,3*S*,4*S*)-3-nitro-7-oxabicyclo[2.2.1]hept-5-ene-2-carboxylate (4)[33]

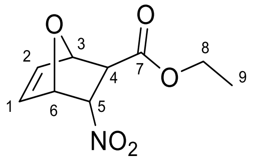

4

An oven-dried 50 mL round-bottom flask equipped with a Teflon-coated magnetic stirring was charged with a yellow solution of 3.63 g (25.0 mmol, 1.0 eq) ethyl (*E*)-3-nitroacrylate (**3**) in 13 mL anhydrous $CHCl_3$. 3.64 mL (50.0 mmol, 2.0 eq) furan (**1**) were added in one portion. The yellow solution was vigorously stirred at RT in the closed flask for 28 h. Afterwards the solvent and all non-reacted volatile starting materials were removed on a rotary evaporator (10 mbar, 35 °C). Finally the brownish crude material was purified via flash column chromatography (520 g SiO_2, 21.0 x 8.0 cm, eluent: cyclohexane:EtOAc = 3:1 (v/v), R_f = 0.23) and the resulting yellowish, crystalline solid was dried under high vacuum.
Yield: 2.70 g (12.7 mmol, 51 %); yellowish, crystalline solid.

$C_9H_{11}NO_5$ [213.19 g/mol].

R_f = 0.42 (cyclohexane:EtOAc = 3:1 (v/v)).

m_p = 54-55 °C.

^{1}H-NMR (300.36 MHz, $CDCl_3$): δ = 6.72 (dd, $^3J_{HH}$ = 5.8 Hz, $^3J_{HH}$ = 1.8 Hz, 1H, H-2), 6.38 (dd, $^3J_{HH}$ = 5.8 Hz, $^3J_{HH}$ = 1.4 Hz, 1H, H-1), 5.53 (dd, $^3J_{HH}$ = 4.8 Hz, $^3J_{HH}$ = 3.0 Hz, 1H, H-5), 5.46 (d, $^3J_{HH}$ = 4.8 Hz, 1H, H-6), 5.33 (d, $^3J_{HH}$ = 0.7 Hz, 1H, H-3), 4.26 (q, $^3J_{HH}$ = 7.1 Hz, 2H, H-8), 3.21 (d, $^3J_{HH}$ = 2.9 Hz, 1H, H-4), 1.32 (t, $^3J_{HH}$ = 7.1 Hz, 3H, H-9).

^{13}C-NMR (75.53 MHz, $CDCl_3$): δ = 169.8 (C_q, C-7), 139.0 (C-2), 133.8 (C-1), 84.4 (C-5), 83.4 (C-3), 79.1 (C-6), 62.3 (C-8), 49.1 (C-4), 14.3 (C-9).

Ethyl (1*S*,2*S*,3*S*,4*R*)-3-nitro-7-oxabicyclo[2.2.1]hept-5-ene-2-carboxylate (11)

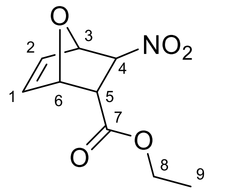

11

$C_9H_{11}NO_5$ [213.19 g/mol].

R_f = 0.33 (cyclohexane:EtOAc = 3:1 (v/v)).

m_p = 34-35 °C.

^{1}H-NMR (300.36 MHz, $CDCl_3$): δ = 6.54 (dd, $^3J_{HH}$ = 5.8 Hz, $^3J_{HH}$ = 1.4 Hz, 1H, H-1), 6.51 (dd, $^3J_{HH}$ = 5.8 Hz, $^3J_{HH}$ = 1.6 Hz, 1H, H-2), 5.50 (s, 1H, H-3), 5.32 (d, $^3J_{HH}$ = 4.8 Hz, 1H, H-6), 4.83 (d, $^3J_{HH}$ = 3.0 Hz, 1H, H-4), 4.16 (q, $^3J_{HH}$ = 7.1 Hz, 2H, H-8), 3.94 (dd, $^3J_{HH}$ = 4.8 Hz, $^3J_{HH}$ = 3.0 Hz, 1H, H-5), 1.26 (t, $^3J_{HH}$ = 7.1 Hz, 3H, H-9).

^{13}C-NMR (75.53 MHz, $CDCl_3$): δ = 168.9 (C_q, C-7), 138.5 (C-1), 134.4 (C-2), 86.8 (C-4), 84.2 (C-3), 79.3 (C-6), 61.9 (C-8), 49.8 (C-5), 14.2 (C-9).

Ethyl (1*R*,2*S*,3*S*,4*S*)-3-((*tert*-butoxycarbonyl)amino)-7-oxabicyclo[2.2.1]-hept-5-ene-2-carboxylate (*rac*-5)[33]

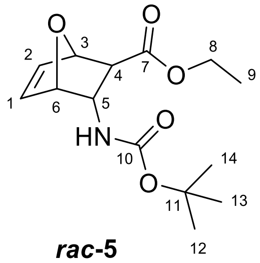

***rac*-5**

An oven-dried 500 mL round-bottom flask equipped with a Teflon-coated magnetic stirring bar was charged with a colorless solution of 4.26 g (20.0 mmol, 1.0 eq) bicyclic Diels-Alder compound **4** in 200 mL EtOH, which was afterwards cooled in an ice-water bath to 0 °C. Consecutively, 27 mL conc. HCl (~36 % (w/w) in H_2O) and 26.2 g (400 mmol, 20.0 eq) activated Zn (Zn washed with 1 M HCl, H_2O as well as MeOH and afterwards dried under high vacuum) were added in small portions to the colorless solution. Immediately after the addition of the Zn an intense H_2 gas formation was observed. After 30 min of vigorously stirring at 0 °C, the ice-water bath was removed and the grey suspension was additionally stirred at RT for 8 h. It was filtered through a pad of Celite® (diameter: 6.0 cm, height: 5.0 cm) and the filter cake was carefully washed with EtOH (2 x 50 mL). The filtrate was collected in an oven-dried 1000 mL round-bottom flask equipped with a Teflon-coated magnetic stirring bar. 55.9 mL (320 mmol, 16.0 eq) DIPEA and 7.76 g (36.0 mmol, 1.8 eq) Boc_2O were added successively and the resulting colorless suspension was vigorously stirred at RT for 22 h. Subsequently it was carefully concentrated under high vacuum, the colorless solid residue was diluted with 800 mL EtOAc and washed with H_2O (1 x 800 mL). The cloudy, colorless aqueous phase was reextracted with EtOAc (2 x 800 mL), the combined yellowish organic layers washed with saturated $NaHCO_3$ (1 x 800 mL), dried over $MgSO_4$, filtered and the solvent was removed under reduced pressure on a rotary evaporator. Finally the colorless solid crude material was purified via flash column chromatography (250 g SiO_2, 19.0 x 6.0 cm, eluent: cyclohexane:EtOAc = 3:1 (v/v), R_f = 0.24) and the resulting colorless solid dried under high vacuum.

Yield: 4.99 g (17.6 mmol, 88 %); colorless solid.

$C_{14}H_{21}NO_5$ [283.32 g/mol].

R_f = 0.33 (cyclohexane:EtOAc = 2:1 (v/v)).

m_p = 97-98 °C.

^{1}H-NMR (300.36 MHz, $CDCl_3$): δ = 6.60 (dd, $^3J_{HH}$ = 5.8 Hz, $^3J_{HH}$ = 1.7 Hz, 1H, H-2), 6.46 (dd, $^3J_{HH}$ = 5.8 Hz, $^3J_{HH}$ = 1.5 Hz, 1H, H-1), 5.12 (s, 1H, H-3), 5.06 (bs, 1H, H-6), 4.54 (bs, 1H, H-5), 4.31 (d, $^3J_{HH}$ = 7.8 Hz, 1H, NH), 4.21 (q, $^3J_{HH}$ = 7.1 Hz, 2H, H-8), 2.05 (d, $^3J_{HH}$ = 3.5 Hz, 1H, H-4), 1.43 (s, 9H, H-12, H-13, H-14), 1.28 (t, $^3J_{HH}$ = 7.1 Hz, 3H, H-9).

^{13}C-NMR (75.53 MHz, $CDCl_3$): δ = 171.9 (C_q, C-7), 155.1 (C_q, C-10), 138.0 (C-2), 134.6 (C-1), 82.2 (C-3), 80.1 (C_q, C-11), 79.1 (C-6), 61.4 (C-8), 53.5 (C-5), 52.5 (C-4), 28.5 (C-12, C-13, C-14), 14.3 (C-9).

(1*R*,2*S*,3*S*,4*S*)-3-((*tert*-Butoxycarbonyl)amino)-7-oxabicyclo[2.2.1]hept-5-ene-2-carboxylic acid (6)[31]

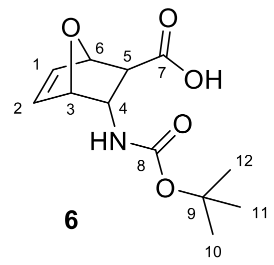

6

Five 250 mL round-bottom flasks equipped with a Teflon-coated magnetic stirring bar were charged with a solution of 984 mg (3.47 mmol, 1.0 eq) bicyclic ester ***rac*-5** in 40 mL Et_2O. After the addition of 70 mL NaH_2PO_4/Na_2HPO_4 buffer (pH 7.6, c = 100 mM) and 3.6 mL PLE-precipitate (in half saturated $(NH_4)_2SO_4$, unknown activity), the yellowish two-phasic mixture was stirred with 200 rpm in the closed flask at RT for 22 h until the non-hydrolyzed enantiomer of compound ***ent*-5** reached an *e.e.* value between 94-96 % (E = 180).
The yellowish reaction mixture was phase separated and the aqueous phase was washed with Et_2O (2 x 600 mL). In order to obtain a better phase separation the mixture was centrifuged. The yellowish aqueous layer was acidified with 50 mL of saturated $KHSO_4$ to pH 1-2 and the product was extracted with DCM (4 x 500 mL). The combined yellowish organic layers were dried over $MgSO_4$, filtered and the solvent was removed under reduced pressure on a rotary evaporator. For purification the brownish crude material was triturated in 15 mL cyclohexane:EtOAc = 2:1 (v/v), collected by filtration, washed with cold cyclohexane:EtOAc = 2:1 (v/v) (2 x 2.0 mL) and the resulting colorless powder was dried under high vacuum.

Yield: 1.96 g (7.68 mmol, 44 %); colorless powder.

chiral HPLC: t_R = 11.31 min (major enantiomer) and 13.15 min (minor enantiomer); *e.e.* = 96 %.
In order to obtain more precise *e.e.* values, this procedure was repeated with 2.10 g (7.41 mmol, 1.0 eq) of the enantiomerically enriched bicyclic ester **5** (*e.e.* = 96 %) as starting material. After reaching an *e.e.* = 38 % of non-hydrolyzed ester ***ent*-5**, the work-up as well as the purification was performed as described above.

Yield: 1.57 g (6.15 mmol, 83 %); colorless powder.

$C_{12}H_{17}NO_5$ [255.26 g/mol].

R_f = 0.24 (EtOAc:MeOH:AcOH = 1000:4:1 (v/v/v)).

m_p = 133-134 °C.

$[\alpha]_D^{31\text{-}32\ °C}$ = -189.0 ° (c = 1.00 in DMSO).

chiral HPLC (Method_ACID): t_R = 11.31 min (major enantiomer) and 13.15 min (minor enantiomer); *e.e.* > 99 %.

^{1}H-NMR (300.36 MHz, DMSO-d$_6$): δ = 12.45 (s, 1H, COOH), 6.84 (d, $^3J_{HH}$ = 4.1 Hz, 1H, NH), 6.56 (d, $^3J_{HH}$ = 5.1 Hz, 1H, H-1), 6.36 (d, $^3J_{HH}$ = 4.6 Hz, 1H, H-2), 4.99 (s, 1H, H-6), 4.92 (d, $^3J_{HH}$ = 3.5 Hz, 1H, H-3), 4.07 (bs, 1H, H-4), 2.24 (s, 1H, H-5), 1.38 (s, 9H, H-10, H-11, H-12).

^{13}C-NMR (75.53 MHz, DMSO-d$_6$): δ = 173.4 (C_q, C-7), 155.4 (C_q, C-8), 137.0 (C-1), 134.1 (C-2), 81.6 (C-6), 78.5 (C-3), 78.0 (C_q, C-9), 52.6 (C-4), 49.6 (C-5), 28.2 (C-10, C-11, C-12).

Ethyl (1*R*,2*S*,3*S*,4*S*)-3-((*tert*-butoxycarbonyl)amino)-7-oxabicyclo[2.2.1]-hept-5-ene-2-carboxylate (5)

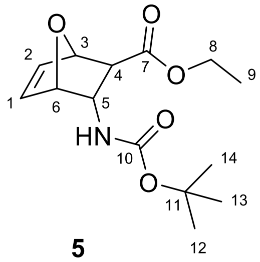

5

An oven-dried, evacuated and argon purged 250 mL Schlenk flask with a Teflon-coated magnetic stirring bar was charged with colorless solution of 1.93 g (7.56 mmol, 1.0 eq) bicyclic carboxylic acid **6** (*e.e.* = 96 %) in 70 mL anhydrous DCM. 661 µL (11.3 mmol, 1.5 eq) anhydrous EtOH as well as 92.4 mg (756 µmol, 10 mol%) 4-DMAP were added in an argon counter flow, respectively. Afterwards the colorless solution was cooled in an ice-water bath to 0 °C and 2.17 g (11.3 mmol, 1.5 eq) EDC.HCl were added in one portion. Immediately after the addition of the coupling reagent the ice-water bath was removed, the Schlenk flask equipped with a bubbler and the colorless solution was stirred at RT for 16 h under argon atmosphere. Afterwards the colorless solution was diluted with 50 mL DCM, washed with H_2O (3 x 60 mL) and the product was reextracted from the colorless aqueous phase with DCM (2 x 30 mL). The combined colorless organic layers were dried over $MgSO_4$, filtered and concentrated on a rotary evaporator. Finally the yellowish crude material was purified via flash column chromatography (80 g SiO_2, 25.0 x 3.0 cm, eluent: cyclohexane:EtOAc = 3:1 (v/v), R_f = 0.24) and the resulting colorless, crystalline solid was dried under high vacuum.

Yield: 2.11 g (7.45 mmol, 99 %); colorless, crystalline solid.

chiral HPLC: t_R = 11.15 min (minor enantiomer) and 13.18 min (major enantiomer); *e.e.* = 96 %.

This procedure was repeated with 1.55 g (6.07 mmol, 1.0 eq) of the enantiomerically enriched bicyclic carboxylic acid **6** (*e.e.* > 99 %) as starting material.

Yield: 1.71 g (6.04 mmol, 99 %); colorless, crystalline solid.

$C_{14}H_{21}NO_5$ [283.32 g/mol].

R_f = 0.43 (cyclohexane:EtOAc = 3:2 (v/v)).

m_p = 104-105 °C.

$[\alpha]_D^{31\text{-}32\,°C}$ = -142.0 ° (c = 1.00 in $CHCl_3$).

chiral HPLC: t_R = 11.15 min (minor enantiomer) and 13.18 min (major enantiomer); *e.e.* > 99 %.

^{1}H-NMR (300.36 MHz, $CDCl_3$): δ = 6.60 (dd, $^3J_{HH}$ = 5.8 Hz, $^3J_{HH}$ = 1.7 Hz, 1H, H-2), 6.46 (dd, $^3J_{HH}$ = 5.8 Hz, $^3J_{HH}$ = 1.5 Hz, 1H, H-1), 5.12 (s, 1H, H-3), 5.06 (bs, 1H, H-6), 4.54 (bs, 1H, H-5), 4.31 (d, $^3J_{HH}$ = 7.8 Hz, 1H, NH), 4.21 (q, $^3J_{HH}$ = 7.1 Hz, 2H, H-8), 2.05 (d, $^3J_{HH}$ = 3.5 Hz, 1H, H-4), 1.43 (s, 9H, H-12, H-13, H-14), 1.28 (t, $^3J_{HH}$ = 7.1 Hz, 3H, H-9).

^{13}C-NMR (75.53 MHz, $CDCl_3$): δ = 171.9 (C_q, C-7), 155.1 (C_q, C-10), 138.0 (C-2), 134.6 (C-1), 82.2 (C-3), 80.1 (C_q, C-11), 79.1 (C-6), 61.4 (C-8), 53.5 (C-5), 52.5 (C-4), 28.5 (C-12, C-13, C-14), 14.3 (C-9).

Potassium hexamethyldisilazide (KHMDS) (7)[34]

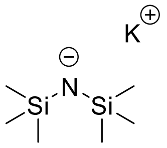

7

An oven-dried, evacuated and argon purged 100 mL three-necked round-bottom flask equipped with a Teflon-coated magnetic stirring bar, gas inlet adapter, reflux condenser and bubbler was charged with 2.36 g (20.6 mmol, 1.1 eq) KH suspension (35 % (w/w) in mineral oil). It was dispersed in 10 mL anhydrous *n*-hexane, the supernatant was removed and the remaining solid was washed with *n*-hexane (3 x 5 mL) as described above. Afterwards the greyish solid was carefully dried under high vacuum, diluted with 20 mL anhydrous THF and 3.8 mL (18.4 mmol, 1.0 eq) 1,1,1,3,3,3-hexamethyldisilazane were added in one portion. The greyish suspension was ultrasonicated in an oil bath for 4 h under argon atmosphere. After a short induction time, heavily gas bubbling was observed and a yellowish suspension was formed. Subsequently, the yellowish suspension was allowed to stand at RT under argon atmosphere for 15 h and the supernatant was transferred into an oven-dried, evacuated and argon purged 80 mL Schlenk flask by cannuling. It can be stored in a freezer at -24 °C under argon atmosphere for several weeks without significant degradation, but has to be titrated before use (see below).
<u>Concentration:</u> 0.76 M (after titration with 1.0 M 2-butanol (in toluene) and 2-(6-butyl-1,6-dihydropyridin-2-yl)pyridine (**12**) in THF as indicator solution).

2-(6-Butyl-1,6-dihydropyridin-2-yl)pyridine (12)[35]

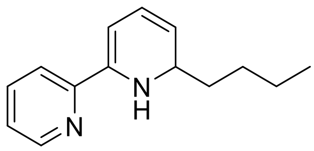

12

An oven-dried, evacuated and argon purged 15 mL Schlenk flask equipped with a Teflon-coated magnetic stirring bar was charged with colorless solution of 5.0 mg (32 µmol, 1.0 eq) 2,2'-bipyridine in 3.0 mL anhydrous THF. Afterwards 63 µL (128 µmol, 4.0 eq) *nBuLi* (2.04 M in *n*-hexane) were added over a period of 5 min via septum and syringe under argon atmosphere at RT, which resulted in the formation of a deeply red colored solution. Finally, 1.0 M 2-butanol (in toluene) was dropwise added under vigorous stirring until a yellowish solution was consistent, which was immediately used after this preparation.

Titration of KHMDS in THF[35]

An oven-dried, evacuated an argon purged 8 mL Schlenk flask was charged with 200 µL of the yellow 2-(6-butyl-1,6-dihydropyridin-2-yl)pyridine (**12**) solution. It was diluted with 1.0 mL of anhydrous THF and one droplet of KHMDS solution was added to eliminate the background noise, whereas a red solution was formed. Subsequently one droplet of a 1.0 M 2-butanol solution (in toluene) was added and afterwards exactly 2.0 mL of the KHMDS solution. The red solution was titrated via septum and syringe with the 1.0 M 2-butanol solution (in toluene) under vigorously stirring until a grey to yellowish suspension was formed indicating the equivalence point.

Ethyl (5*S*,6*S*)-6-((*tert*-butoxycarbonyl)amino)-5-hydroxycyclohexa-1,3-diene-1-carboxylate (8)[31]

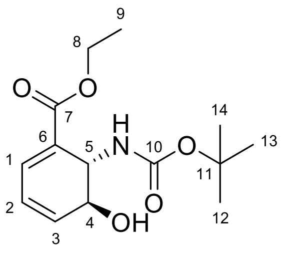

8

An oven-dried, evacuated and argon purged 30 mL Schlenk flask with a Teflon-coated magnetic stirring bar was charged with a colorless solution of 1.13 g (4.00 mmol, 1.0 eq) bicyclic ester **5** (*e.e.* > 99 %) in 17 mL anhydrous THF and afterwards it was cooled in an acetone/dry ice bath to -45 °C under argon atmosphere. In parallel 13 mL (12.0 mmol, 3.0 eq) of a 0.923 M KHMDS solution (in anhydrous THF) were transferred into a second oven-dried, evacuated and argon purged 250 mL Schlenk flask and diluted with 20 mL anhydrous THF. This yellowish suspension was also cooled in an acetone/dry ice bath to -45 °C under inert atmosphere. In an argon counter flow the colorless solution of starting material was added to the yellowish, cloudy KHMDS solution in one portion, the Schlenk flask with the former solution of ester **5** was rinsed with 10 mL anhydrous THF and the resulting yellow solution was vigorously stirred at -45 °C in an acetone/dry ice bath for 100 min. Afterwards the resulting orange, cloudy solution was poured into 100 mL saturated NH_4Cl and extracted with EtOAc (3 x 60 mL). The combined yellowish, organic layers were dried over $MgSO_4$, filtered and concentrated on a rotary evaporator. Finally the orange, oily crude material was purified via flash column chromatography (130 g SiO_2, 14.0 x 5.0 cm, eluent: cyclohexane:EtOAc = 1:1 (v/v), R_f = 0.25) and the resulting yellowish, highly viscous liquid was dried under high vacuum.

Yield: 1.02 g (3.60 mmol, 90 %); yellowish, highly viscous liquid.

$C_{14}H_{21}NO_5$ [283.32 g/mol].

R_f = 0.39 (cyclohexane:EtOAc = 2:3 (v/v)).

$[\alpha]_D^{31\text{-}32\ °C}$ = +300.1 ° (c = 1.50 in $CHCl_3$); *e.e.* > 99 %.

^{1}H-NMR (300.36 MHz, $CDCl_3$): δ = 7.16 (d, $^3J_{HH}$ = 3.6 Hz, 1H, H-1), 6.30-6.22 (m, 2H, H-2, H-3), 4.76 (d, $^3J_{HH}$ = 7.2 Hz, 1H, H-5), 4.47 (bs, 1H, NH), 4.32 (bs, 1H, H-4), 4.28-4.13 (m, 2H, H-8), 3.03 (bs, 1H, OH), 1.42 (s, 9H, H-12, H-13, H-14), 1.28 (t, $^3J_{HH}$ = 7.1 Hz, 3H, H-9).

^{13}C-NMR (75.53 MHz, $CDCl_3$): δ = 166.0 (C_q, C-7), 155.6 (C_q, C-10), 133.6 (C-1), 132.8 (C-3), 127.5 (C_q, C-6), 124.6 (C-2), 80.2 (C_q, C-11), 67.9 (C-4), 61.0 (C-8), 50.3 (C-5), 28.5 (C-12, C-13, C-14), 14.3 (C-9).

(5*S*,6*S*)-6-((*tert*-Butoxycarbonyl)amino)-5-hydroxycyclohexa-1,3-diene-1-carboxylic acid (13)[31]

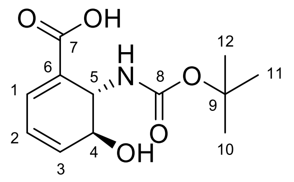

13

A 10 mL round-bottom flask with a Teflon-coated magnetic stirring bar was charged with a colorless solution of 567 mg (2.00 mmol, 1.0 eq) ester **8** (*e.e.* > 99 %) in 11 mL THF. Afterwards 1.0 mL (10.0 mmol, 5.0 eq) 10 M KOH in H_2O were added in one portion. The resulting light orange solution was vigorously stirred at RT for 19 h. It was concentrated on a rotary evaporator and the brownish, oily residue was dissolved in 50 mL H_2O. The brownish aqueous phase was washed with EtOAc (2 x 50 mL), the product from the organic phase reextracted with H_2O (2 x 10 mL) and the combined brownish aqueous layers subsequently acidified with 5.0 mL saturated $KHSO_4$ to pH 1-2. The product was extracted with EtOAc (4 x 100 mL), the combined yellow organic phases were dried over $MgSO_4$, filtered and the solvent was removed on a rotary evaporator. Finally the crude material was purified via flash column chromatography (50 g SiO_2, 10.0 x 3.5 cm, eluent: EtOAc:AcOH = 1000:1 (v/v), R_f = 0.18) and the resulting colorless powder was dried under high vacuum.

Yield: 383 mg (1.50 mmol, 79 %); colorless solid.

$C_{12}H_{17}NO_5$ [255.27 g/mol].

R_f = 0.31 (EtOAc:MeOH:AcOH = 1000:100:1 (v/v/v)).

m_p = 168-169 °C (decomposition).

$[\alpha]_D^{31\text{-}32\ °C}$ = +466.4 ° (c = 1.00 in DMSO); *e.e.* > 99 %.

^{1}H-NMR (300.36 MHz, DMSO-d_6): δ = 12.28 (s, 1H, COOH), 7.00-6.98 (m, 1H, H-1), 6.58 (d, $^3J_{HH}$ = 7.4 Hz, 1H, NH), 6.20-6.13 (m, 2H, H-2, H-3), 5.07 (d, $^3J_{HH}$ = 5.6 Hz, 1H, OH), 4.43 (d, $^3J_{HH}$ = 7.2 Hz, 1H, H-5), 3.91 (bs, 1H, H-4), 1.36 (s, 9H, H-10, H-11, H-12).

^{13}C-NMR (75.53 MHz, DMSO-d_6): δ = 167.4 (C_q, C-7), 155.1 (C_q, C-8), 133.3 (C-1), 133.1 (C-3), 126.9 (C_q, C-6), 123.9 (C-2), 77.7 (C_q, C-9), 66.2 (C-4), 49.0 (C-5), 28.2 (C-10, C-11, C-12).

(1*S*,6*S*)-2-Carboxy-6-hydroxycyclohexa-2,4-diene-1-ammonium 2,2,2-trifluoroacetate (9) (DHHA)

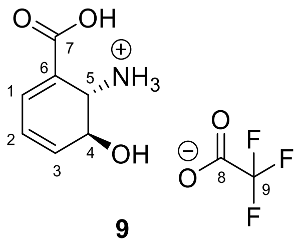

9
DHHA

An oven-dried 25 mL round-bottom flask equipped with a Teflon-coated magnetic stirring bar was charged with a colorless suspension of 383 mg (1.50 mmol, 1.0 eq) carboxylic acid **13** (*e.e.* > 99 %) in 5.0 mL DCM and 750 µL TFA were added (15 % TFA in DCM (v/v)), respectively. A yellowish solution was immediately formed after the addition of TFA, it turned brownish by the time and the desired product precipitated in the form of a brownish solid. The suspension was stirred at RT for 90 min. Subsequently it was cooled in an ice-water bath to 0 °C, the precipitate was collected by filtration and washed with a small amount of cooled DCM (2 x 700 µL). Finally the brownish, powdery crude material was purified by trituration in 1.6 mL DCM:MeCN = 2:1 (v/v). It was collected by filtration, washed with cold DCM:MeCN = 2:1 (v/v) (2 x 300 µL) and the resulting colorless powder was dried under high vacuum.

Yield: 323 mg (1.20 mmol, 80 %); colorless powder.

$C_9H_{10}F_3NO_5$ [269.17 g/mol].

m_p = 161-162 °C (decomposition).

$[\alpha]_D^{32\text{-}33\ °C}$ = +364.4 ° (c = 0.50 in DMSO); *e.e.* > 99 %.

^{1}H-NMR (300.36 MHz, D_2O): δ = 7.45 (d, $^3J_{HH}$ = 5.3 Hz, 1H, H-1), 6.51-6.40 (m, 2H, H-2, H-3), 4.47-4.45 (m, 1H, H-4), 4.38 (d, $^3J_{HH}$ = 3.0 Hz, 1H, H-5).

^{1}H-NMR (300.36 MHz, DMSO-d_6): δ = 13.08 (bs, 1H, COOH), 8.07 (bs, 3H, NH_3^+), 7.25-7.22 (m, 1H, H-1), 6.37-6.29 (m, 2H, H-2, H-3), 5.64 (bs, 1H, OH), 4.16 (s, 1H, H-4), 4.04 (s, 1H, H-5).

^{13}C-NMR (125.69 MHz, D_2O): δ = 168.4 (C_q, C-7), 162.9 (C_q, q, $^2J_{CF}$ = 35.5 Hz, C-8), 137.6 (C-1), 131.8 (C-3), 125.1 (C_q, C-6), 122.0 (C-2), 116.2 (C_q, q, $^1J_{CF}$ = 292 Hz, C-9), 64.0 (C-4), 48.7 (C-5).
^{19}F-NMR (470.35 MHz, D_2O): δ = -75.7 (decoupled, CF_3).

(5*S*,6*S*)-6-((5-(((*S*)-1-Amino-1-oxopropan-2-yl)amino)-2,4-dinitrophenyl)-amino)-5-hydroxycyclohexa-1,3-diene-1-carboxylic acid (14)

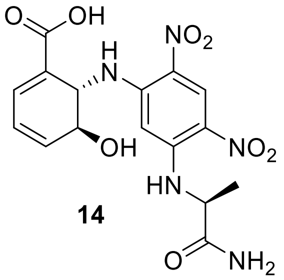

Ammonium salt **9** was derivatized with Marfey's reagent in order to determine the *e.e.* value of this compound.

HPLC-MS: t_R = 1.51 min (minor diastereomer) and 1.80 min (major diastereomer); *d.e.* > 99 %, consequently *e.e.* > 99 % of compound **9**.

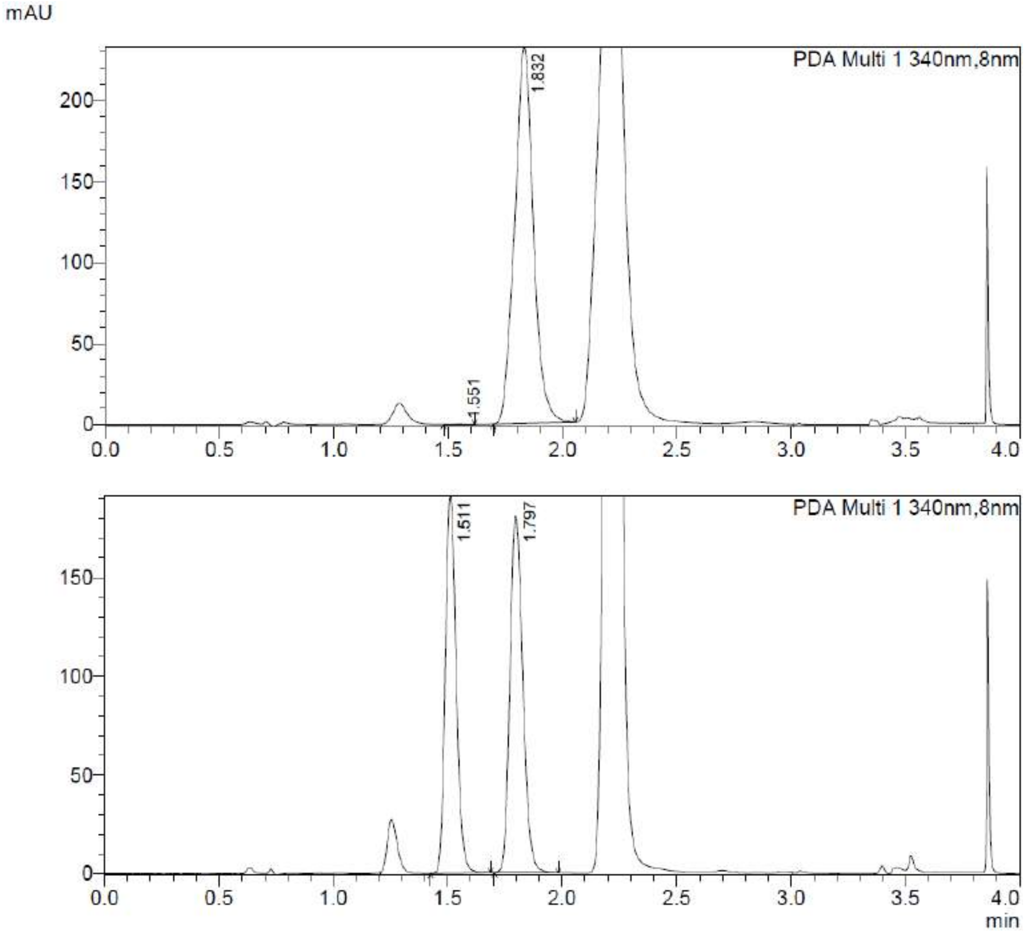

HPLC-MS chromatograms of **14** and ***rac*-14** for the determination of the diastereomeric excess (*d.e.*).

Synthesis of d-DHHA (20)

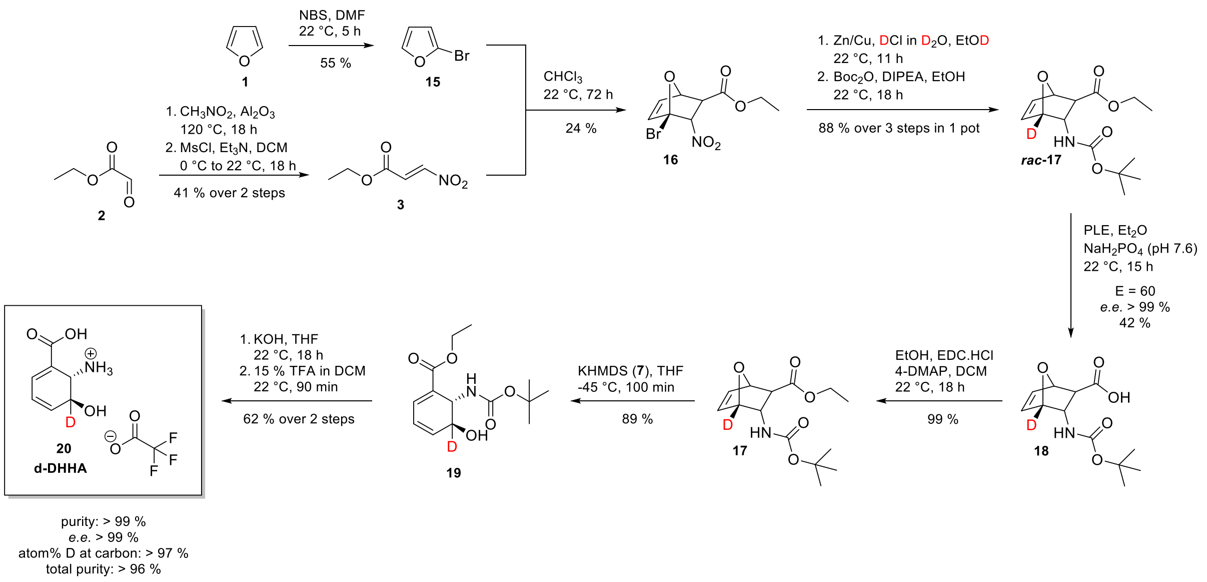

Synthesis of d-DHHA in form of a TFA salt **20** in analogy to the published synthesis of DHHA (**9**) by Steel.[31]

2-Bromofuran (15)[36]

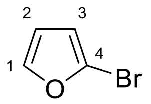

15

An oven-dried, evacuated and argon purged 250 mL three-necked round-bottom flask equipped with a Teflon-coated magnetic stirring bar, gas inlet adapter, 100 mL dropping funnel, internal thermometer and bubbler was charged with a solution of 15.3 g (225 mmol, 2.0 eq) furan in 40 mL anhydrous DMF. Afterwards a deeply orange solution consisting of 20.0 g (112 mmol, 1.0 eq) *N*-bromosuccinimide (NBS) in 60 mL anhydrous DMF was added via the dropping funnel at RT over a period of 50 min. The temperature did not exceed 35 °C, and the brownish reaction mixture was vigorously stirred at RT for additional 5 h. Afterwards the brown solution was concentrated on a rotary evaporator (10 mbar, 35 °C) to remove the excess of unreacted furan. The remaining solution was purified by water steam distillation. Therefore the storage vessel filled with H_2O was heated in an oil bath to 140 °C, the brown solution, however, in a second oil bath to 105 °C. Due to contamination with furan, the first few drops of condensate were discarded. The collected colorless condensate was washed with H_2O (1 x 30 mL) to remove residual DMF and finally the colorless, clear product was stored in an inert 25 mL Schlenk flask over dried K_2CO_3 under argon atmosphere in a freezer at -20 °C for several weeks.

Yield: 9.03 g (61.4 mmol, 55 %); clear, colorless liquid.

C_4H_3BrO [146.97 g/mol].

b_p = 65-67 °C (350 mbar).

^{1}H-NMR (300.36 MHz, $CDCl_3$): δ = 7.43-7.42 (m, 1H, H-1), 6.38-6.36 (m, 1H, H-2), 6.31-6.30 (m, 1H, H-3).

^{13}C-NMR (75.53 MHz, $CDCl_3$): δ = 144.5 (C-1), 122.1 (C_q, C-4), 112.6 (C-2), 111.3 (C-3).

Ethyl (1*R*,2*S*,3*S*,4*R*)-4-bromo-3-nitro-7-oxabicyclo[2.2.1]hept-5-ene-2-carboxylate (16)

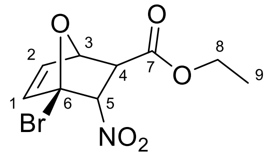

16

An oven-dried 50 mL round-bottom flask equipped with a Teflon-coated magnetic stirring bar was charged with a colorless solution of 2.47 g (17.0 mmol, 1.0 eq) ethyl (*E*)-3-nitroacrylate (**3**) in 8 mL anhydrous $CHCl_3$. Afterwards 3.75 g (25.5 mmol, 1.5 eq) 2-bromofuran (**15**) were added in one portion. The yellowish solution was stirred in the closed flask at RT for 72 h. The solvent and all non-reacted volatile starting materials were removed on a rotary evaporator (10 mbar, 35 °C). Finally, the oily, orange crude material was purified via flash column chromatography (440 g SiO_2, 21.5 x 7.5 cm, eluent: cyclohexane:EtOAc = 12:1 (v/v), R_f = 0.17) from all other diastereomers and the resulting colorless solid dried under high vacuum.

Yield: 1.18 g (4.0 mmol, 24 %); colorless solid.

$C_9H_{10}NO_5Br$ [292.08 g/mol].

R_f = 0.60 (cyclohexane:EtOAc = 2:1 (v/v)).

m_p = 40-41 °C.

^{1}H-NMR (300.36 MHz, $CDCl_3$): δ = 6.69 (dd, $^3J_{HH}$ = 5.6 Hz, $^3J_{HH}$ = 2.1 Hz, 1H, H-2), 6.45 (d, $^3J_{HH}$ = 5.6 Hz, 1H, H-1), 5.51 (d, $^3J_{HH}$ = 3.2 Hz, 1H, H-5), 5.31 (d, $^3J_{HH}$ = 1.7 Hz, 1H, H-3), 4.29 (q, $^3J_{HH}$ = 7.1 Hz, 2H, H-8), 3.31 (d, $^3J_{HH}$ = 3.2 Hz, 1H, H-4), 1.33 (t, $^3J_{HH}$ = 7.1 Hz, 3H, H-9).

^{13}C-NMR (75.53 MHz, $CDCl_3$): δ = 168.4 (C_q, C-7), 138.5 (C-2), 138.2 (C-1), 90.3 (C-5), 88.0 (C_q, C-6), 82.1 (C-3), 62.7 (C-8), 52.7 (C-4), 14.2 (C-9).

HRMS: calculated for $C_9H_{10}NO_5Br^+$: 290.9742; found: 290.9729.

Ethyl (1*S*,2*R*,3*S*,4*R*)-1-bromo-3-nitro-7-oxabicyclo[2.2.1]hept-5-ene-2-carboxylate (21)

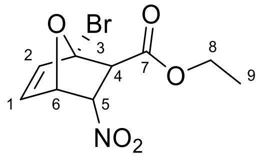

21

$C_9H_{10}NO_5Br$ [292.08 g/mol].

R_f = 0.60 (cyclohexane:EtOAc = 2:1 (v/v)).

^{1}H-NMR (300.36 MHz, $CDCl_3$): δ = 6.70 (d, $^3J_{HH}$ = 5.6 Hz, 1H, H-2), 6.45 (dd, $^3J_{HH}$ = 5.6 Hz, $^3J_{HH}$ = 1.7 Hz, 1H, H-1), 5.59 (dd, $^3J_{HH}$ = 4.9 Hz, $^3J_{HH}$ = 3.4 Hz, 1H, H-5), 5.46 (dd, $^3J_{HH}$ = 5.1 Hz, $^3J_{HH}$ = 1.6 Hz, 1H, H-6), 4.43-4.25 (m, 2H, H-8), 3.52 (d, $^3J_{HH}$ = 3.3 Hz, 1H, H-4), 1.38 (t, $^3J_{HH}$ = 7.2 Hz, 3H, H-9).

^{13}C-NMR (75.53 MHz, $CDCl_3$): δ = 168.2 (C_q, C-7), 143.2 (C-2), 135.1 (C-1), 91.1 (C_q, C-3), 85.8 (C-5), 77.9 (C-6), 62.6 (C-8), 54.6 (C-4), 14.4 (C-9).

Ethyl (1*S*,2*S*,3*S*,4*S*)-4-bromo-3-nitro-7-oxabicyclo[2.2.1]hept-5-ene-2-carboxylate (22)

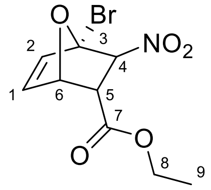

22

$C_9H_{10}NO_5Br$ [292.08 g/mol].

R_f = 0.49 (cyclohexane:EtOAc = 2:1 (v/v)).

^{1}H-NMR (300.36 MHz, $CDCl_3$): δ = 6.63 (dd, $^3J_{HH}$ = 5.6 Hz, $^3J_{HH}$ = 1.7 Hz, 1H, H-1), 6.48 (d, $^3J_{HH}$ = 5.6 Hz, 1H, H-2), 5.35 (dd, $^3J_{HH}$ = 4.9 Hz, $^3J_{HH}$ = 1.5 Hz, 1H, H-6), 5.08 (d, $^3J_{HH}$ = 3.4 Hz, 1H, H-4), 4.17 (q, $^3J_{HH}$ = 7.1 Hz, 2H, H-8), 4.06 (dd, $^3J_{HH}$ = 4.8 Hz, $^3J_{HH}$ = 3.5 Hz, 1H, H-5), 1.26 (t, $^3J_{HH}$ = 7.1 Hz, 3H, H-9).

^{13}C-NMR (75.53 MHz, $CDCl_3$): δ = 167.7 (C_q, C-7), 140.0 (C-1), 138.7 (C-2), 90.5 (C_q, C-3), 90.5 (C-4), 78.5 (C-6), 62.3 (C-8), 52.5 (C-5), 14.2 (C-9).

Ethyl (1*R*,2*R*,3*S*,4*R*)-1-bromo-3-nitro-7-oxabicyclo[2.2.1]hept-5-ene-2-carboxylate (23)

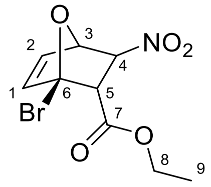

23

$C_9H_{10}NO_5Br$ [292.08 g/mol].

R_f = 0.49 (cyclohexane:EtOAc = 2:1 (v/v)).

^{1}H-NMR (300.36 MHz, $CDCl_3$): δ = 6.60 (d, $^3J_{HH}$ = 5.6 Hz, 1H, H-1), 6.50 (dd, $^3J_{HH}$ = 5.6 Hz, $^3J_{HH}$ = 2.0 Hz, 1H, H-2), 5.49 (d, $^3J_{HH}$ = 2.1 Hz, 1H, H-3), 4.85 (d, $^3J_{HH}$ = 3.1 Hz, 1H, H-4), 4.32-4.16 (m, 2H, H-8), 4.08 (d, $^3J_{HH}$ = 3.1 Hz, 1H, H-5), 1.32 (t, $^3J_{HH}$ = 7.1 Hz, 3H, H-9).

^{13}C-NMR (75.53 MHz, $CDCl_3$): δ = 167.7 (C_q, C-7), 142.6 (C-1), 133.9 (C-2), 88.3 (C-4), 87.7 (C_q, C-6), 82.8 (C-3), 62.5 (C-8), 57.6 (C-5), 14.2 (C-9).

Ethyl (1*R*,2*S*,3*S*,4*S*)-3-((*tert*-butoxycarbonyl)amino)-7-oxabicyclo[2.2.1]-hept-5-ene-2-carboxylate-4-*d* (*rac*-17)

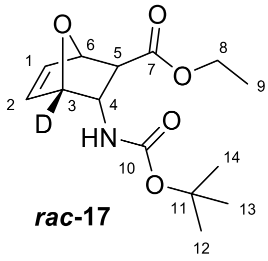

***rac*-17**

An oven-dried 250 mL three-necked round-bottom flask equipped with a Teflon-coated magnetic stirring bar, gas inlet adapter and bubbler was charged with a colorless solution of 3.65 g (12.5 mmol, 1.0 eq) bicyclic Diels-Alder compound **16** in 110 mL EtOD and afterwards cooled in an ice-water bath to 0 °C. Consecutively, 25.8 mL conc. DCl (~38 % (w/w) in D_2O, 99.5 atom% D) and 24.5 g (375 mmol, 30.0 eq) Zn/Cu couple (max. 3 % Cu) were added in small portions to the colorless solution in an argon counter flow. Immediately after the addition of the Zn/Cu couple intense D_2 gas formation was observed. After 30 min of vigorously stirring at 0 °C, the ice-water bath was removed and the grey suspension was additionally stirred at RT for 10 h. It was filtered through a pad of anhydrous Celite® (diameter: 3.0 cm, height: 4.0 cm) and the filter cake was washed with EtOD (2 x 20 mL). The filtrate was collected in an oven-dried 500 mL round-bottom flask equipped with a Teflon-coated magnetic stirring bar. 52.4 mL (300 mmol, 24.0 eq) DIPEA and 4.91 g (22.5 mmol, 1.8 eq) Boc_2O were added successively and the resulting colorless suspension was vigorously stirred at RT for 16 h. Subsequently it was carefully concentrated under high vacuum, the colorless solid residue was diluted with 600 mL EtOAc, washed with H_2O (1 x 600 mL) and the cloudy, colorless aqueous phase was reextracted with EtOAc (2 x 600 mL). The combined yellowish organic layers were washed with saturated $NaHCO_3$ (1 x 600 mL), dried over $MgSO_4$, filtered and the solvent was removed under reduced pressure on a rotary evaporator. Finally the colorless solid crude material was purified via flash column chromatography (350 g SiO_2, 17.0 x 8.0 cm, eluent: cyclohexane:EtOAc = 3:1 (v/v), R_f = 0.24) and the resulting colorless solid dried under high vacuum.

Yield: 3.12 g (11.0 mmol, 88 %); colorless solid.

$C_{14}H_{20}DNO_5$ [284.33 g/mol].

R_f = 0.33 (cyclohexane:EtOAc = 2:1 (v/v).

m_p = 88-89 °C.

^{1}H-NMR (300.36 MHz, $CDCl_3$): δ = 6.60 (dd, $^3J_{HH}$ = 5.8 Hz, $^3J_{HH}$ = 1.4 Hz, 1H, H-1), 6.46 (d, $^3J_{HH}$ = 5.8 Hz, 1H, H-2), 5.12 (bs, 1H, H-6), 4.53 (bs, 1H, H-4), 4.29 (d, $^3J_{HH}$ = 7.8 Hz, 1H, NH), 4.21 (q, $^3J_{HH}$ = 7.1 Hz, 2H, H-8), 2.05 (d, $^3J_{HH}$ = 3.4 Hz, 1H, H-5), 1.43 (s, 9H, H-12, H-13, H-14), 1.28 (t, $^3J_{HH}$ = 7.1 Hz, 3H, H-9).

^{13}C-NMR (75.53 MHz, $CDCl_3$): δ = 171.9 (C_q, C-7), 155.2 (C_q, C-10), 137.9 (C-1), 134.5 (C-2), 82.2 (C-6), 80.1 (C_q, C-11), 78.8 ($^1J_{CD}$ = 25.5 Hz, C-3), 61.4 (C-8), 52.5 (C-4), 28.4 (C-12, C-13, C-14), 14.3 (C-9).

Ethyl (1*R*,2*S*,3*S*,4*S*)-3-((*tert*-butoxycarbonyl)amino)-7-oxabicyclo[2.2.1]-hept-5-ene-2-carboxylic-4-*d* acid (18)

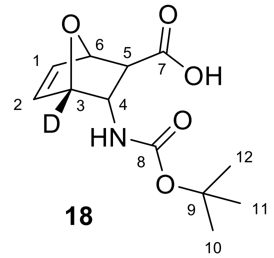

18

This preparation (total amount of deuterated bicyclic ester ***rac*-17**: 2.70 g (9.50 mmol, 1.0 eq)) was divided into three smaller preparations with 900 mg (3.17 mmol, 1.0 eq) of compound ***rac*-17** each. Each 250 mL round-bottom flask equipped with a Teflon-coated magnetic stirring bar was charged with a colorless solution of 900 mg (3.17 mmol, 1.0 eq) deuterated bicyclic ester ***rac*-17** in 36 mL Et_2O. After the addition of 64 mL NaH_2PO_4/Na_2HPO_4 buffer (pH 7.6, c = 100 mM) and 3.6 mL PLE-precipitate (in half saturated $(NH_4)_2SO_4$, unknown activity) to each preparation, the yellowish two-phasic mixtures were stirred with 150 rpm in closed systems at RT for 22 h until the non-hydrolyzed enantiomer of compound ***ent*-17** reached an *e.e.* value between 73-75 % (E = 60).
The combined yellowish reaction mixtures were phase separated and the aqueous phase was washed with Et_2O (2 x 300 mL). In order to obtain a better phase separation the mixture was centrifuged. The yellowish aqueous layer was acidified with 30 mL of saturated $KHSO_4$ to pH 1-2 and the product was extracted with DCM (4 x 300 mL). The combined yellowish organic layers were dried over $MgSO_4$, filtered and the solvent was removed under reduced pressure on a rotary evaporator. For purification the brownish crude material was triturated in 12 mL cyclohexane:EtOAc = 2:1 (v/v), collected by filtration, washed with cold cyclohexane:EtOAc = 2:1 (v/v) (2 x 2.0 mL) and the resulting colorless powder was dried under high vacuum.

Yield: 1.03 g (4.00 mmol, 42 %); colorless powder.

chiral HPLC: t_R = 11.31 min (major enantiomer) & 13.15 min (minor enantiomer); *e.e.* = 93 %.
In order to gain perfect *e.e.* values, this procedure was repeated with 650 mg (2.29 mmol, 1.0 eq) of the enantiomerically enriched deuterated bicyclic ester **17** (*e.e.* = 93 %) as starting material. After reaching an *e.e.* = 67 % of non-hydrolyzed ester ***ent*-17**, the work-up as well as the purification was performed as described above.

Yield: 407 mg (1.58 mmol, 69 %); colorless powder.

$C_{12}H_{16}DNO_5$ [256.27 g/mol].

R_f = 0.24 (EtOAc:MeOH:AcOH = 1000:4:1 (v/v/v)).

m_p = 123-124 °C.

$[\alpha]_D^{31\text{-}32\ °C}$ = -189.3 ° (c = 0.50 in DMSO).
chiral HPLC: t_R = 11.31 min (major enantiomer) & 13.15 min (minor enantiomer); *e.e.* > 99 %.

^{1}H-NMR (300.36 MHz, DMSO-d$_6$): δ = 12.44 (s, 1H, COOH), 6.83 (bs, 1H, NH), 6.56 (d, $^3J_{HH}$ = 4.2 Hz, 1H, H-1), 6.36 (d, $^3J_{HH}$ = 4.7 Hz, 1H, H-2), 4.99 (s, 1H, H-6), 4.07 (bs, 1H, H-4), 2.24 (s, 1H, H-5), 1.38 (s, 9H, H-10, H-11, H-12).

^{13}C-NMR (75.53 MHz, DMSO-d$_6$): δ = 173.3 (C$_q$, C-7), 155.3 (C$_q$, C-8), 137.0 (C-1), 134.1 (C-2), 81.6 (C-6), 78.2 ($^1J_{CD}$ = 25.2 Hz, C-3), 78.0 (C$_q$, C-9), 52.6 (C-4), 49.6 (C-5), 28.1 (C-10, C-11, C-12).
HRMS (DI-EI): calculated for $[C_{12}H_{16}DNO_5 - C_4H_3DO]^+$: 187.0845; found: 187.0852.

Ethyl (1*R*,2*S*,3*S*,4*S*)-3-((*tert*-butoxycarbonyl)amino)-7-oxabicyclo[2.2.1]-hept-5-ene-2-carboxylate-4-*d* (17)

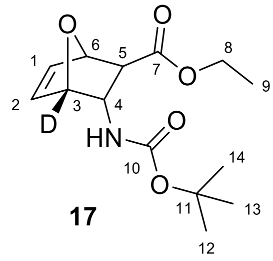

17

An oven-dried, evacuated and argon purged 100 mL Schlenk flask with a Teflon-coated magnetic stirring bar was charged with a colorless solution of 980 mg (3.82 mmol, 1.0 eq) deuterium labeled bicyclic carboxylic acid **18** (*e.e.* = 93 %) in 40 mL anhydrous DCM. 335 µL (5.74 mmol, 1.5 eq) anhydrous EtOH as well as 46.7 mg (382 µmol, 10 mol%) 4-DMAP were added in an argon counter flow, respectively. Afterwards the colorless solution was cooled in an ice-water bath to 0 °C and 1.10 g (5.74 mmol, 1.5 eq) EDC.HCl were added in one portion. Immediately after the addition of coupling reagent the ice-water bath was removed, the Schlenk flask equipped with a bubbler and the colorless solution was stirred at RT for 16 h under argon atmosphere. Afterwards the colorless solution was diluted with 40 mL DCM and washed with H_2O (3 x 40 mL). The colorless organic phase was dried over $MgSO_4$, filtered and concentrated on a rotary evaporator. Finally the yellowish solid was purified via flash column chromatography (55 g SiO_2, 13.0 x 3.0 cm, eluent: cyclohexane:EtOAc = 3:1 (v/v), R_f = 0.24) and the resulting colorless, crystalline solid was dried under high vacuum.

Yield: 1.07 g (3.76 mmol, 99 %); colorless, crystalline solid.

chiral HPLC: t_R = 11.15 min (minor enantiomer) and 13.18 min (major enantiomer); *e.e.* = 93 %.
This procedure was repeated with 360 mg (1.40 mmol, 1.0 eq) of the enantiomerically pure deuterated bicyclic carboxylic acid **18** (*e.e.* > 99 %) as starting material.

Yield: 394 mg (1.39 mmol, 99 %); colorless, crystalline solid.

$C_{14}H_{20}DNO_5$ [284.33 g/mol].

R_f = 0.33 (cyclohexane:EtOAc = 2:1 (v/v)).

m_p = 101-102 °C.

$[\alpha]_D^{33\text{-}34\ °C}$ = -142.7 ° (c = 1.00 in $CHCl_3$).

chiral HPLC: t_R = 11.15 min (minor enantiomer) and 13.18 min (major enantiomer); *e.e.* > 99 %.

^{1}H-NMR (300.36 MHz, $CDCl_3$): δ = 6.60 (dd, $^3J_{HH}$ = 5.8 Hz, $^3J_{HH}$ = 1.4 Hz, 1H, H-1), 6.46 (d, $^3J_{HH}$ = 5.8 Hz, 1H, H-2), 5.12 (bs, 1H, H-6), 4.53 (bs, 1H, H-4), 4.29 (d, $^3J_{HH}$ = 7.8 Hz, 1H, NH), 4.21 (q, $^3J_{HH}$ = 7.1 Hz, 2H, H-8), 2.05 (d, $^3J_{HH}$ = 3.4 Hz, 1H, H-5), 1.43 (s, 9H, H-12, H-13, H-14), 1.28 (t, $^3J_{HH}$ = 7.1 Hz, 3H, H-9).

^{13}C-NMR (75.53 MHz, $CDCl_3$): δ = 171.9 (C_q, C-7), 155.2 (C_q, C-10), 137.9 (C-1), 134.5 (C-2), 82.2 (C-6), 80.1 (C_q, C-11), 78.8 ($^1J_{CD}$ = 25.5 Hz, C-3), 61.4 (C-8), 52.5 (C-4), 28.4 (C-12, C-13, C-14), 14.3 (C-9).
HRMS (DI-EI): calculated for $[C_{14}H_{20}DNO_5 - C_4H_3DO]^+$: 215.1158; found: 215.1170.

Ethyl (5*S*,6*S*)-6-((*tert*-butoxycarbonyl)amino)-5-hydroxycyclohexa-1,3-diene-1-carboxylate-5-*d* (19)

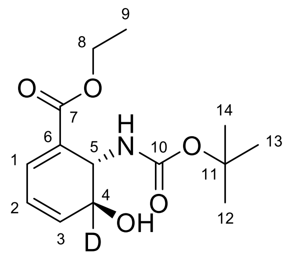

19

An oven-dried, evacuated and argon purged 15 mL Schlenk flask with a Teflon-coated magnetic stirring bar was charged with a colorless solution of 420 mg (1.48 mmol, 1.0 eq) deuterium labeled bicyclic ester **17** (*e.e.* > 99 %) in 6.5 mL anhydrous THF and afterwards cooled in an acetone/dry ice bath to -45 °C under argon atmosphere. In parallel 5.4 mL (4.43 mmol, 3.0 eq) of a 0.829 M KHMDS solution (in anhydrous THF) were transferred into a second oven-dried, evacuated and argon purged 80 mL Schlenk flask and diluted with 7.6 mL anhydrous THF. This yellowish, cloudy solution was also cooled in an acetone/dry ice bath to -45 °C under inert atmosphere. In an argon counter flow the colorless solution of starting material was added to the yellowish KHMDS solution in one portion, the Schlenk flask with the former solution of ester **17** was rinsed with 2.0 mL anhydrous THF and the resulting yellow suspension was vigorously stirred at -45 °C in the acetone/dry ice bath for 100 min. Afterwards the resulting orange, cloudy solution was poured into 40 mL saturated NH_4Cl and extracted with EtOAc (3 x 30 mL). The combined yellowish, organic layers were dried over $MgSO_4$, filtered and concentrated on a rotary evaporator. Finally the yellow, oily crude material was purified via flash column chromatography (55 g SiO_2, 12.0 x 4.0 cm, eluent: cyclohexane:EtOAc = 5:4 (v/v), R_f = 0.22) and the resulting yellowish, highly viscous liquid was dried under high vacuum.

Yield: 375 mg (1.32 mmol, 89 %); yellowish, highly viscous liquid.

$C_{14}H_{20}DNO_5$ [284.33 g/mol].

R_f = 0.31 (cyclohexane:EtOAc = 2:3 (v/v)).

$[\alpha]_D^{33\text{-}34\ °C}$ = +298.3 ° (c = 1.50 in $CHCl_3$); *e.e.* > 99 %.

^{1}H-NMR (300.36 MHz, $CDCl_3$): δ = 7.17-7.15 (m, 1H, H-1), 6.29-6.22 (m, 2H, H-2, H-3), 4.75 (d, $^3J_{HH}$ = 8.0 Hz, 1H, H-5), 4.46 (bs, 1H, NH), 4.31-4.13 (m, 2H, H-8), 3.09 (bs, 1H, OH), 1.42 (s, 9H, H-12, H-13, H-14), 1.28 (t, $^3J_{HH}$ = 7.1 Hz, 3H, H-9).

^{13}C-NMR (75.53 MHz, $CDCl_3$): δ = 166.0 (C_q, C-7), 155.6 (C_q, C-10), 133.6 (C-1), 132.7 (C-3), 127.5 (C_q, C-6), 124.7 (C-2), 80.2 (C_q, C-11), 67.4 ($^1J_{CD}$ = 22.5 Hz, C-4), 61.0 (C-8), 50.2 (C-5), 28.5 (C-12, C-13, C-14), 14.3 (C-9).

HRMS: calculated for $C_{14}H_{20}DNO_5^+$: 284.1483; found: 284.1489.

(5*S*,6*S*)-6-((*tert*-Butoxycarbonyl)amino)-5-hydroxycyclohexa-1,3-diene-1-carboxylic-5-*d* acid (24)

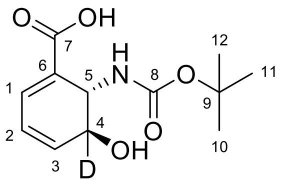

24

A 10 mL round-bottom flask with a Teflon-coated magnetic stirring bar was charged with a solution of 370 mg (1.30 mmol, 1.0 eq) deuterium labeled ester **19** (*e.e.* > 99 %) in 6.0 mL THF and 650 µL (6.50 mmol, 5.0 eq) 10 M KOH in H_2O were added in one portion. The resulting light orange solution was vigorously stirred at RT for 15 h. Afterwards it was concentrated on a rotary evaporator and the brownish, oily residue was dissolved in 30 mL H_2O. The brownish aqueous phase was washed with EtOAc (2 x 30 mL) and subsequently acidified with 2.0 mL saturated $KHSO_4$ to pH 1-2. The product was extracted with EtOAc (4 x 50 mL), the combined yellow organic phases were dried over $MgSO_4$, filtered and the solvent was removed on a rotary evaporator. Finally the crude material was purified via flash column chromatography (35 g SiO_2, 25.0 x 2.0 cm, eluent: EtOAc:AcOH = 1000:1 (v/v), R_f = 0.18) and the resulting colorless powder was dried under high vacuum.

Yield: 264 mg (1.03 mmol, 79 %); colorless powder.

$C_{12}H_{16}DNO_5$ [256.27 g/mol].

R_f = 0.28 (EtOAc:MeOH:AcOH = 1000:30:1 (v/v/v)).

m_p = 168-169 °C (decomposition).

$[\alpha]_D^{32\text{-}33\,°C}$ = +420.8 ° (c = 1.00 in DMSO); *e.e.* > 99 %.

^{1}H-NMR (300.36 MHz, DMSO-d_6): δ = 12.26 (s, 1H, COOH), 7.00-6.98 (m, 1H, H-1), 6.56 (d, $^3J_{HH}$ = 6.9 Hz, 1H, NH), 6.20-6.16 (m, 2H, H-2, H-3), 5.03 (s, 1H, OH), 4.43 (d, $^3J_{HH}$ = 7.2 Hz, 1H, H-5), 1.37 (s, 9H, H-10, H-11, H-12).

^{13}C-NMR (75.53 MHz, DMSO-d_6): δ = 167.4 (C_q, C-7), 155.1 (C_q, C-8), 133.2 (C-1), 133.0 (C-3), 126.9 (C_q, C-6), 123.9 (C-2), 77.7 (C_q, C-9), 65.7 ($^1J_{CD}$ = 21.6 Hz, C-4), 49.0 (C-5), 28.2 (C-10, C-11, C-12).
HRMS (DI-EI): calculated for $C_{12}H_{16}DNO_5^+$: 256.1169; found: 256.1184.

(1*S*,6*S*)-2-Carboxy-6-hydroxycyclohexa-2,4-diene-6-*d*-1-ammonium 2,2,2-trifluoroacetate (20) (d-DHHA)

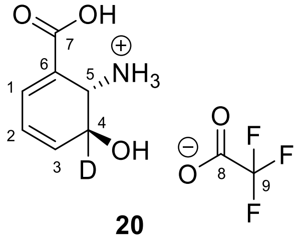

20
d-DHHA

An oven-dried 10 mL round-bottom flask equipped with a Teflon-coated magnetic stirring bar was charged with a colorless suspension of 235 mg (917 µmol, 1.0 eq) deuterium labeled carboxylic acid **24** (*e.e.* > 99 %) in 3.5 mL DCM. After the addition of 525 µL TFA (15 % TFA in DCM (v/v)), a yellowish solution was immediately formed, which turned brownish by the time. Afterwards the suspension was stirred at RT for 90 min, in which the desired product precipitated in the form of a brownish solid. Subsequently it was cooled in an ice-water bath to 0 °C, the precipitate was collected by filtration and washed with a small amount of cooled DCM (2 x 400 µL). Finally the brownish, powdery crude material was purified by trituration in 1.0 mL DCM:MeCN = 2:1 (v/v). It was collected by filtration, washed with cold DCM:MeCN = 2:1 (v/v) (2 x 200 µL) and the resulting colorless powder was dried under high vacuum.

Yield: 193 mg (714 µmol, 78 %); colorless powder.

$C_9H_9DF_3NO_5$ [270.18 g/mol].

m_p = 161-162 °C (decomposition).

$[\alpha]_D^{32\text{-}33\ °C}$ = +364.4 ° (c = 0.50 in DMSO); *e.e.* > 99 %.

^{1}H-NMR (300.36 MHz, D_2O): δ = 7.47 (d, $^3J_{HH}$ = 5.2 Hz, 1H, H-1), 6.53-6.43 (m, 2H, H-2, H-3), 4.40 (s, 1H, H-5).

^{1}H-NMR (300.36 MHz, DMSO-d_6): δ = 13.06 (bs, 1H, COOH), 8.05 (bs, 3H, NH_3^+), 7.24-7.23 (m, 1H, H-1), 6.37-6.30 (m, 2H, H-2, H-3), 5.60 (s, 1H, OH), 4.04 (s, 1H, H-5).

^{13}C-NMR (125.69 MHz, D_2O): δ = 168.5 (C_q, C-7), 162.9 (C_q, q, $^2J_{CF}$ = 35.4 Hz, C-8), 137.7 (C-1), 131.8 (C-3), 125.2 (C_q, C-6), 122.1 (C-2), 116.3 (C_q, q, $^1J_{CF}$ = 292 Hz, C-9), 63.7 ($^1J_{CD}$ = 22.4 Hz, C-4), 48.8 (C-5).

^{19}F-NMR (470.35 MHz, DMSO-d_6): δ = -73.5 (decoupled, CF_3).
HRMS (DI-EI): calculated for $C_7H_8DNO_3^+$: 156.0645; found: 156.0651.

(5*S*,6*S*)-6-((5-(((*S*)-1-Amino-1-oxopropan-2-yl)amino)-2,4-dinitrophenyl)-amino)-5-hydroxycyclohexa-1,3-diene-1-carboxylic-5-*d* acid (25)

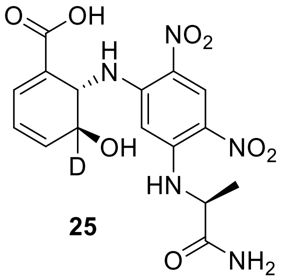

25

Ammonium salt **20** was derivatized with Marfey's reagent in order to determine the *e.e.* value of compound **20**.

HPLC-MS: t_R = 1.51 min (minor diastereomer) and 1.80 min (major diastereomer); *d.e.* > 99 %, consequently *e.e.* > 99 % of compound **20**.

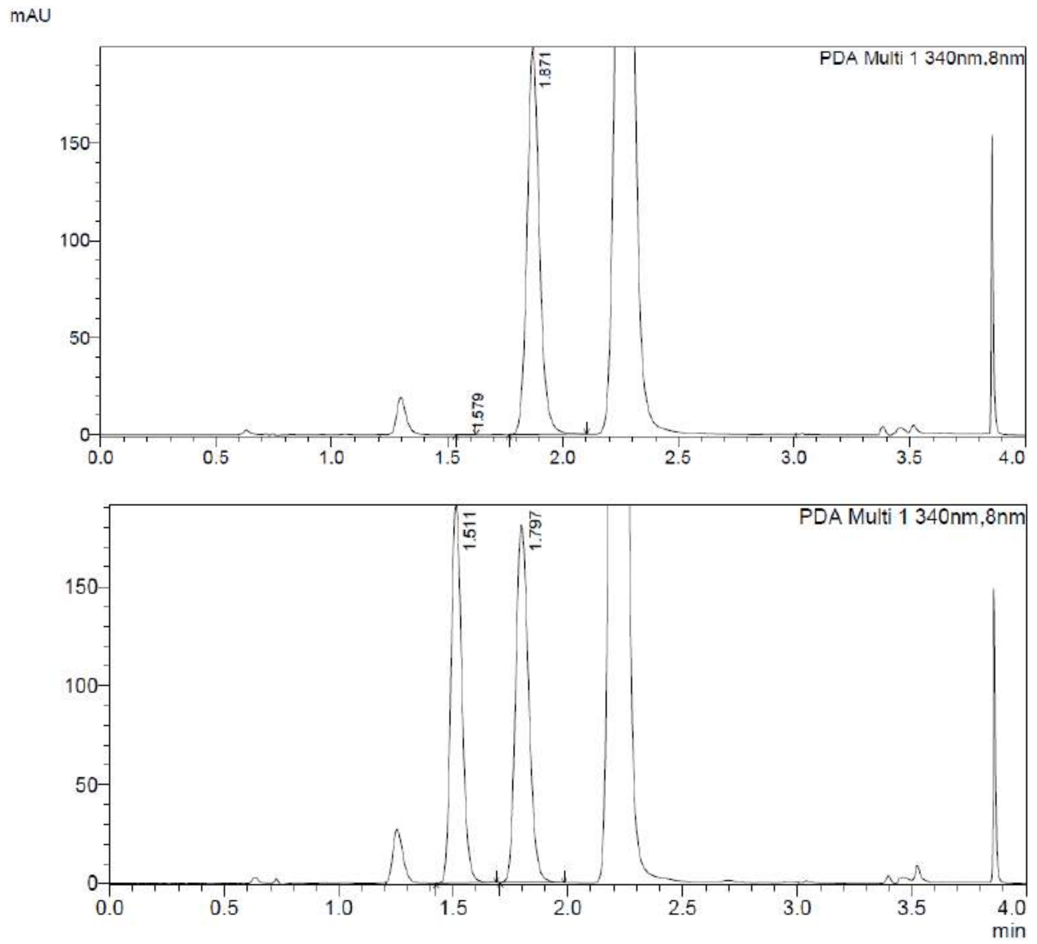

HPLC-MS chromatograms of **25** and ***rac*-25** for the determination of the diastereomeric excess (*d.e.*).

Synthesis of *O*-Alkylated DHHA Derivatives *O*-Me-DHHA (26), rac-*O*-Me-DHHA (*rac*-26), *rac*-*O*-Et-DHHA (*rac*-27) and *rac*-*O*-*n*Pr-DHHA (*rac*-28)

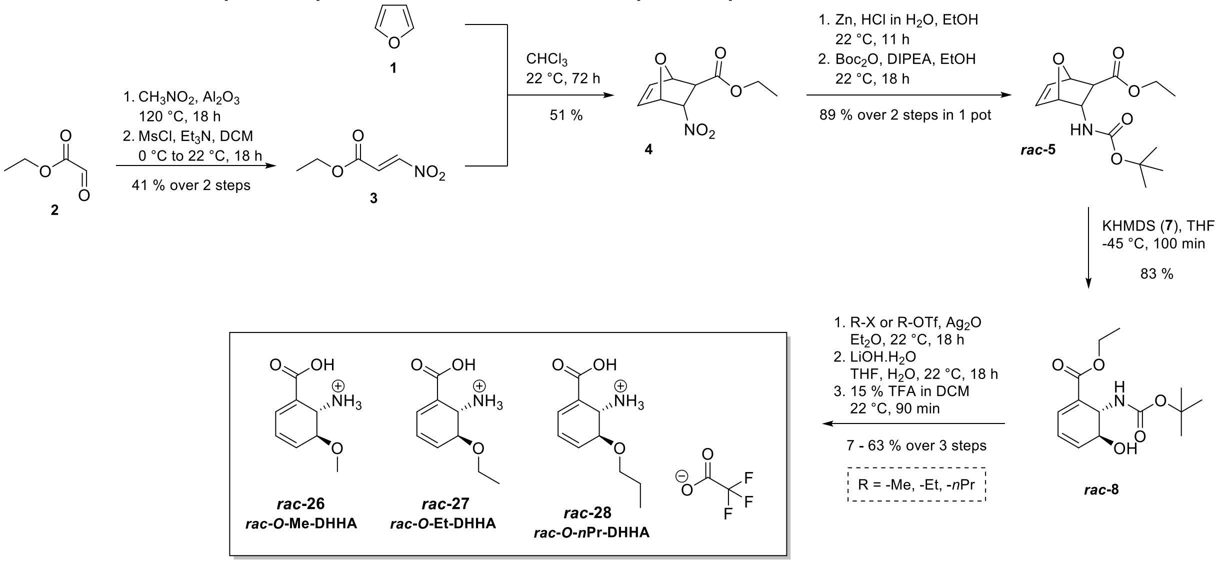

Synthesis of *O*-alkylated DHHA derivatives in form of TFA salts in analogy to the synthesis of DHHA (**9**) published by Steel.[31]

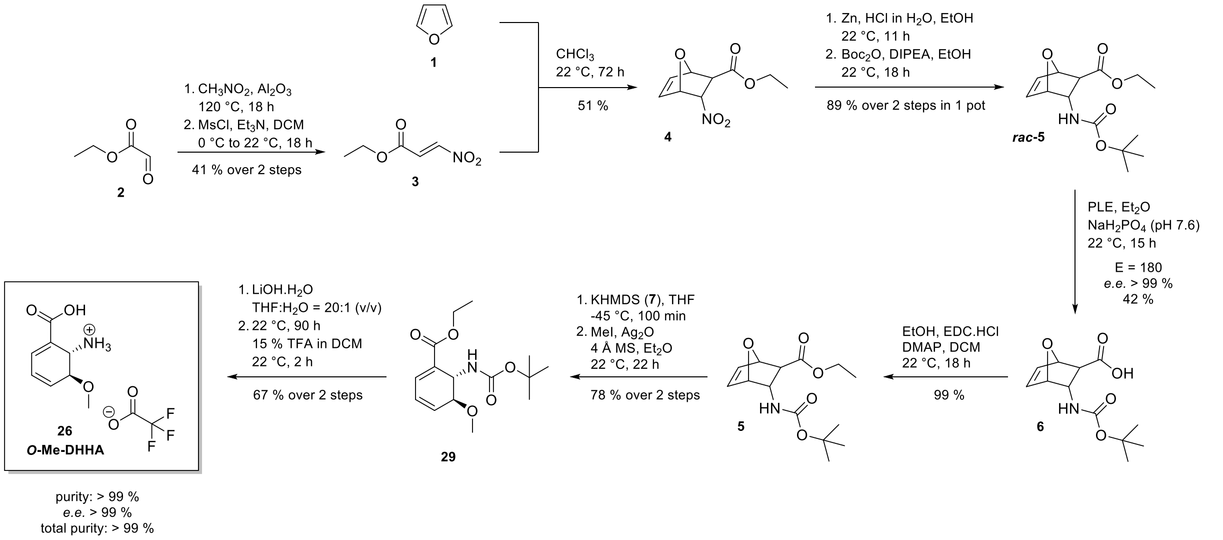

Synthesis of enantiomerically pure *O*-Me-DHHA (**26**) in analogy to the synthesis of DHHA (**9**) by Steel.[31]

Ethyl (5*S*,6*S*)-6-((*tert*-butoxycarbonyl)amino)-5-methoxycyclohexa-1,3-diene-1-carboxylate (29)

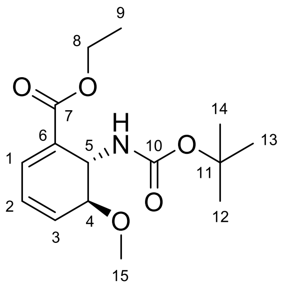

29

An oven-dried 25 mL round-bottom flask equipped with a Teflon-coated magnetic stirring bar was charged with a solution of 312 mg (1.10 mmol, 1.0 eq) ester **8** (*e.e.* > 99 % or racemate) in 13 mL anhydrous Et_2O. Afterwards 510 mg (2.20 mmol, 2.0 eq) Ag_2O, 2.0 g 4 Å MS and 411 µL (6.60 mmol, 6.0 eq) iodomethane (MeI) were added, respectively, and the black suspension was stirred in the closed flask at RT for 22 h. Subsequently it was filtered through a pad of Celite® (diameter: 3.0 cm, height: 4.0 cm), the filter cake was washed with EtOAc (3 x 50 mL) and the solvent was removed on a rotary evaporator. Finally, the yellow, viscous crude material was purified via flash column chromatography (33 g SiO_2, 21.0 x 2.0 cm, eluent: cyclohexane:EtOAc = 3:1 (v/v), R_f = 0.28) and the resulting colorless, highly viscous liquid was dried under high vacuum.

Yield: 307 mg (1.03 mmol, 94 %); colorless, highly viscous liquid.

$C_{15}H_{23}NO_5$ [297.34 g/mol].

R_f = 0.60 (cyclohexane:EtOAc = 1:1 (v/v)).

$[\alpha]_D^{31\text{-}32\,°C}$ = +312.8 ° (c = 1.00 in $CHCl_3$); *e.e.* > 99 %.

^{1}H-NMR (300.36 MHz, $CDCl_3$): δ = 7.15 (d, $^3J_{HH}$ = 5.0 Hz, 1H, H-1), 6.32-6.22 (m, 2H, H-2, H-3), 4.90 (d, $^3J_{HH}$ = 7.0 Hz, 1H, H-5), 4.31-4.13 (m, 3H, H-8, NH), 3.86 (bs, 1H, H-4), 3.48 (s, 3H, H-15), 1.43 (s, 9H, H-12, H-13, H-14), 1.28 (t, $^3J_{HH}$ = 7.1 Hz, 3H, H-9).

^{13}C-NMR (75.53 MHz, $CDCl_3$): δ = 165.8 (C_q, C-7), 155.1 (C_q, C-10), 133.4 (C-1), 130.5 (C-3), 128.1 (C_q, C-6), 125.6 (C-2), 79.9 (C_q, C-11), 75.9 (C-4), 60.9 (C-8), 56.8 (C-15), 45.6 (C-5), 28.5 (C-12, C-13, C-14), 14.3 (C-9).

HRMS: calculated for $C_{15}H_{23}NO_5^+$: 297.1576; found: 297.1567.

(5*S*,6*S*)-6-((*tert*-Butoxycarbonyl)amino)-5-methoxycyclohexa-1,3-diene-1-carboxylic acid (30)

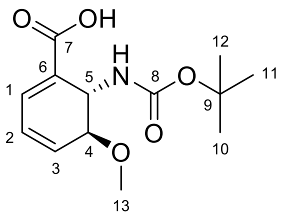

30

A 25 mL round-bottom flask equipped with a Teflon-coated magnetic stirring bar was charged with a colorless solution of 279 mg (940 µmol, 1.0 eq) methoxy ester **29** (ee > 99 % or racemate) in 8.0 mL THF. 400 µL H_2O as well as 158 mg (3.76 mmol, 4.0) $LiOH.H_2O$ were added, respectively and the resulting yellowish, milky solution was stirred in a closed system at RT for 90 h. Afterwards it was concentrated on a rotary evaporator and the oily residue was dissolved in 25 mL H_2O. The yellowish aqueous phase was washed with EtOAc (2 x 25 mL), the product from the organic phase reextracted with H_2O (2 x 5 mL) and the combined yellowish aqueous layers subsequently acidified with 2.0 mL saturated $KHSO_4$ to pH 1-2. The product was extracted with EtOAc (3 x 25 mL), the combined yellowish organic phases were dried over $MgSO_4$, filtered and the solvent was removed on a rotary evaporator. Finally, the crude material was purified via flash column chromatography (36 g SiO_2, 22.5 x 2.0 cm, eluent: cyclohexane:EtOAc:AcOH = 1000:5000:1 (v/v/v), R_f = 0.22) and the resulting colorless, viscous liquid was dried under high vacuum.

Yield: 248 mg (921 µmol, 98 %); colorless, viscous liquid.

$C_{13}H_{19}NO_5$ [269.29 g/mol].

R_f = 0.49 (EtOAc:AcOH = 1000:1 (v/v), UV and CAM).

$[\alpha]_D^{31\text{-}32\,°C}$ = +325.9 ° (c = 0.50 in DMSO); *e.e.* > 99 %.

^{1}H-NMR (300.36 MHz, DMSO-d$_6$): δ = 12.40 (bs, 1H, COOH), 7.00 (d, $^3J_{HH}$ = 5.1 Hz, 1H, H-1), 6.75 (d, $^3J_{HH}$ = 7.1 Hz, 1H, NH), 6.31-6.20 (m, 2H, H-2, H-3), 4.54 (d, $^3J_{HH}$ = 6.9 Hz, 1H, H-5), 3.69 (d, $^3J_{HH}$ = 3.8 Hz, 1H, H-4), 3.30 (s, 3H, H-13), 1.37 (s, 9H, H-10, H-11, H-12).

^{13}C-NMR (75.53 MHz, DMSO-d$_6$): δ = 167.1 (C_q, C-7), 155.0 (C_q, C-8), 133.1 (C-1), 129.7 (C-3), 127.4 (C_q, C-6), 125.8 (C-2), 77.9 (C_q, C-9), 75.2 (C-4), 55.5 (C-13), 45.6 (C-5), 28.2 (C-10, C-11, C-12).

HRMS: calculated for $C_{13}H_{19}NO_5^+$: 269.1263; found: 269.1271.

(1*S*,6*S*)-2-Carboxy-6-methoxycyclohexa-2,4-diene-1-ammonium 2,2,2-trifluoroacetate (26) (*O*-Me-DHHA)

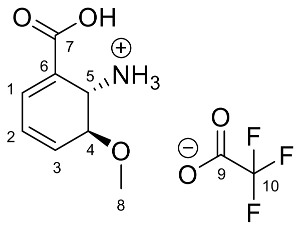

26
***O*-Me-DHHA**

An oven-dried 10 mL round-bottom flask equipped with a Teflon-coated magnetic stirring bar was charged with a colorless solution of 249 mg (925 µmol, 1.0 eq) methoxy carboxylic acid **30** (*e.e.* > 99 % or racemate) in 3.0 mL DCM. After the addition of 450 µL TFA (15 % TFA in DCM (v/v)), a brownish solution was immediately formed, which turned greenish over the time. It was stirred at RT for 120 min. Subsequently the solvent was removed on a rotary evaporator and the remaining brownish solid was triturated in 3.0 mL DCM. The resulting brownish suspension was cooled in an ice-water bath to 0 °C, the precipitate was collected by filtration and washed with a small amount of cooled DCM (2 x 500 µL). Finally the brownish, powdery crude material was purified by a second trituration step in 2.0 mL DCM:MeCN = 4:1 (v/v). It was collected by filtration, washed with cold DCM:MeCN = 4:1 (v/v) (2 x 400 µL) and the resulting colorless powder was dried under high vacuum.

Yield: 177 mg (625 µmol, 68 %); colorless powder.

$C_{10}H_{12}F_3NO_5$ [283.20 g/mol].

m_p = 144-145 °C.

$[\alpha]_D^{32-33\ °C}$ = +313.8 ° (c = 0.50 in DMSO); *e.e.* > 99 %.

^{1}H-NMR (300.36 MHz, D_2O): δ = 7.44 (d, $^3J_{HH}$ = 5.2 Hz, 1H, H-1), 6.57-6.47 (m, 2H, H-2, H-3), 4.51 (d, $^3J_{HH}$ = 2.8 Hz, 1H, H-5), 4.23-4.21 (m, 1H, H-4), 3.45 (s, 3H, H-8).

^{1}H-NMR (300.36 MHz, DMSO-d_6): δ = 13.03 (bs, 1H, COOH), 8.21 (bs, 3H, NH_3^+), 7.24 (dd, $^3J_{HH}$ = 4.2 Hz, $^3J_{HH}$ = 1.9 Hz, 1H, H-1), 6.48-6.40 (m, 2H, H-2, H-3), 4.18 (d, $^3J_{HH}$ = 1.7 Hz, 1H, H-5), 3.98 (s, 1H, H-4), 3.30 (s, 3H, H-8).

^{13}C-NMR (125.69 MHz, D_2O): δ = 168.2 (C_q, C-7), 162.9 (C_q, q, $^2J_{CF}$ = 35.6 Hz, C-9), 137.4 (C-1), 129.3 (C-3), 126.3 (C-2), 122.5 (C_q, C-6), 116.2 (C_q, q, $^1J_{CF}$ = 292 Hz, C-10), 72.7 (C-4), 56.1 (C-8), 45.7 (C-5).

^{19}F-NMR (470.35 MHz, D_2O): δ = -75.7 (decoupled, CF_3).

HRMS: calculated for $C_8H_{11}NO_3^+$: 169.0739; found: 169.0738.

(5*S*,6*S*)-6-((5-(((*S*)-1-Amino-1-oxopropan-2-yl)amino)-2,4-dinitrophenyl)-amino)-5-methoxycyclohexa-1,3-diene-1-carboxylic acid (31)

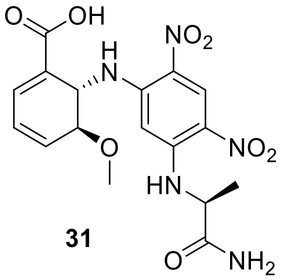

31

Ammonium salt **26** was derivatized with Marfey's reagent in order to determine the *e.e.* value of this compound.

HPLC-MS: t_R = 4.16 min (minor diastereomer) and 5.58 min (major diastereomer); *d.e.* > 99 %, consequently *e.e.* > 99 % of compound **26**.

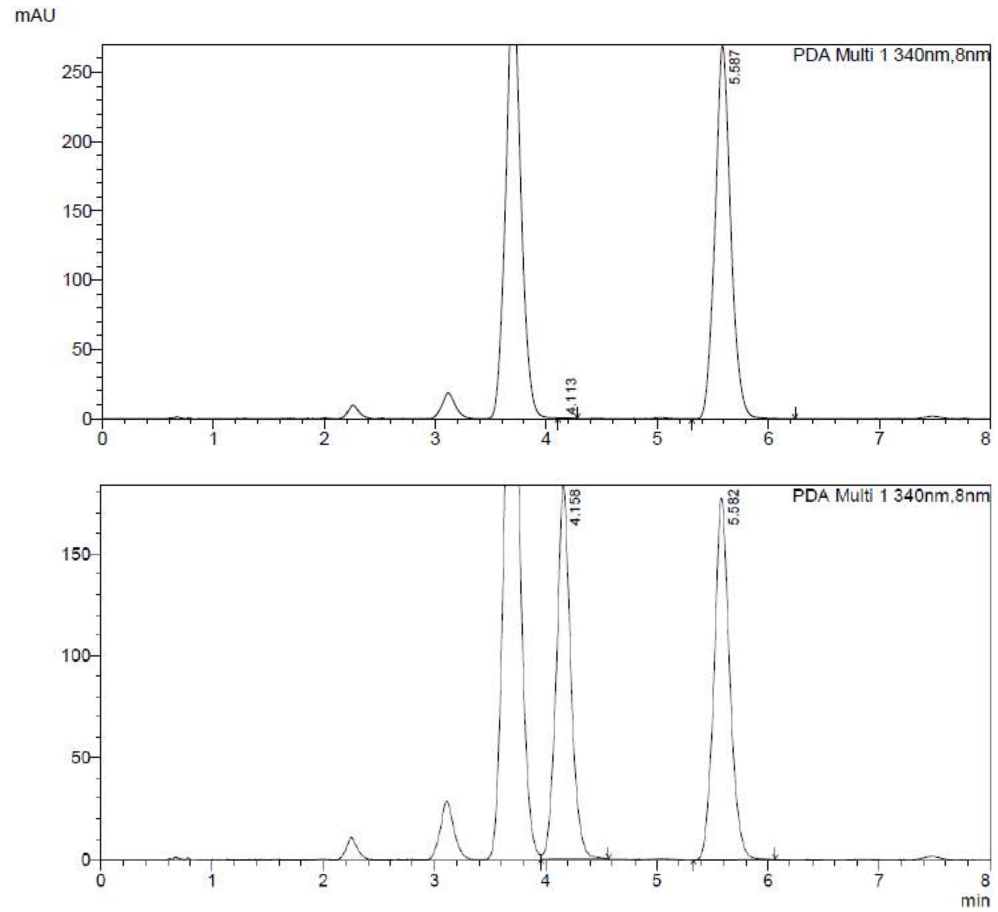

HPLC-MS chromatograms of **31** and ***rac*-31** for the determination of the diastereomeric excess (*d.e.*).

Ethyl (5*S*,6*S*)-6-((*tert*-Butoxycarbonyl)amino)-5-ethoxycyclohexa-1,3-diene-1-carboxylate (*rac*-32)

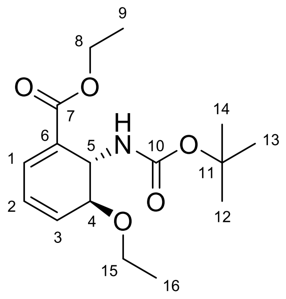

***rac*-32**

An oven-dried, evacuated and argon purged 25 mL two-necked round-bottom flask equipped with a Teflon-coated magnetic stirring bar and gas inlet adapter was charged with a solution of 283 mg (1.00 mmol, 1.0 eq) racemic ester ***rac*-8** in 12 mL anhydrous Et_2O. Afterwards 463 mg (2.00 mmol, 2.0 eq) Ag_2O, 2.0 g 3 Å MS and 778 µL (6.00 mmol, 6.0 eq) ethyl trifluoromethanesulfonate were added in an argon counter flow, respectively, and the black suspension was stirred in the closed flask under argon atmosphere at RT for 70 h. Subsequently it was filtered through a pad of Celite® (diameter: 3.0 cm, height: 4.0 cm), the filter cake was washed with EtOAc (4 x 50 mL) and the solvent was removed on a rotary evaporator. Finally, the yellow, viscous crude material was purified via flash column chromatography (50 g SiO_2, 30.0 x 3.0 cm, eluent: cyclohexane:EtOAc = 4:1 (v/v), R_f = 0.18) and the resulting yellowish, highly viscous liquid was dried under high vacuum.

Yield: 78 mg (250 µmol, 25 %); yellowish, highly viscous liquid.

$C_{16}H_{25}NO_5$ [311.38 g/mol].

R_f = 0.68 (cyclohexane:EtOAc = 1:1 (v/v)).

^{1}H-NMR (300.36 MHz, $CDCl_3$): δ = 7.17 (d, $^3J_{HH}$ = 5.1 Hz, 1H, H-1), 6.31-6.18 (m, 2H, H-2, H-3), 4.89 (d, $^3J_{HH}$ = 7.1 Hz, 1H, H-5), 4.32-4.13 (m, 3H, H-8, NH), 3.96 (d, $^3J_{HH}$ = 3.9 Hz, 1H, H-4), 3.88-3.84 (m, 1H, H-15), 3.69-3.59 (m, 1H, H-15), 1.44 (s, 9H, H-12, H-13, H-14), 1.29 (t, $^3J_{HH}$ = 7.1 Hz, 3H, H-9), 1.19 (t, $^3J_{HH}$ = 7.0 Hz, 3H, H-16).

^{13}C-NMR (75.53 MHz, $CDCl_3$): δ = 166.0 (C_q, C-7), 155.1 (C_q, C-10), 133.6 (C-1), 131.0 (C-3), 128.0 (C_q, C-6), 125.2 (C-2), 79.9 (C_q, C-11), 74.6 (C-4), 64.8 (C-15), 60.9 (C-8), 46.1 (C-5), 28.5 (C-12, C-13, C-14), 15.7 (C-16), 14.4 (C-9).

HRMS: calculated for $C_{16}H_{25}NO_5^+$: 311.1733; found: 311.1747.

(5*S*,6*S*)-6-((*tert*-Butoxycarbonyl)amino)-5-ethoxycyclohexa-1,3-diene-1-carboxylic acid (*rac*-33)

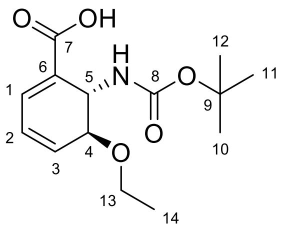

***rac*-33**

A 25 mL round-bottom flask equipped with a Teflon-coated magnetic stirring bar was charged with a colorless solution of 78 mg (250 µmol, 1.0 eq) ethoxy ester ***rac*-32** in 2.0 mL THF. 175 µL H_2O as well as 63 mg (1.50 mmol, 6.0 eq) LiOH.H_2O were added, respectively, and the resulting yellowish, milky solution was stirred in the closed flask at RT for 6 d. Afterwards it was concentrated on a rotary evaporator and the oily residue was dissolved in 10 mL H_2O. The yellowish aqueous phase was washed with EtOAc (2 x 10 mL), the product from the organic phase reextracted with H_2O (2 x 2.0 mL) and the combined yellowish aqueous layers subsequently acidified with 1.0 mL saturated $KHSO_4$ to pH 1-2. The product was extracted with EtOAc (3 x 20 mL), the combined yellowish organic phases were dried over $MgSO_4$, filtered and the solvent was removed on a rotary evaporator. Finally the crude material was purified via flash column chromatography (7.0 g SiO_2, 25.0 x 0.8 cm, eluent: cyclohexane:EtOAc:AcOH = 1000:5000:1 (v/v/v), R_f = 0.24) and the resulting colorless solid was dried under high vacuum.

Yield: 67 mg (238 µmol, 95 %); colorless solid.

$C_{14}H_{21}NO_5$ [283.32 g/mol].

R_f = 0.52 (EtOAc:AcOH = 1000:1 (v/v)).

m_p = 53-54 °C.

^{1}H-NMR (300.36 MHz, DMSO-d$_6$): δ = 12.37 (bs, 1H, COOH), 7.00 (d, $^3J_{HH}$ = 4.8 Hz, 1H, H-1), 6.74 (d, $^3J_{HH}$ = 7.2 Hz, 1H, NH), 6.20-6.19 (m, 2H, H-2, H-3), 4.53 (d, $^3J_{HH}$ = 7.1 Hz, 1H, H-5), 3.77 (d, $^3J_{HH}$ = 3.8 Hz, 1H, H-4), 3.70-3.59 (m, 1H, H-13), 3.57-3.46 (m, 1H, H-13), 1.37 (s, 9H, H-10, H-11, H-12), 1.07 (t, $^3J_{HH}$ = 7.0 Hz, 3H, H-14).

^{13}C-NMR (75.53 MHz, DMSO-d$_6$): δ = 167.2 (C_q, C-7), 155.1 (C_q, C-8), 133.2 (C-1), 130.3 (C-3), 127.3 (C_q, C-6), 125.4 (C-2), 77.9 (C_q, C-9), 73.8 (C-4), 63.1 (C-13), 46.1 (C-5), 28.2 (C-10, C-11, C-12), 15.5 (C-14).

HRMS: calculated for $C_{14}H_{21}NO_5^+$: 283.1420; found: 283.1442.

(1*S*,6*S*)-2-Carboxy-6-ethoxycyclohexa-2,4-diene-1-ammonium 2,2,2-trifluoroacetate (*rac*-27) (*O*-Et-DHHA)

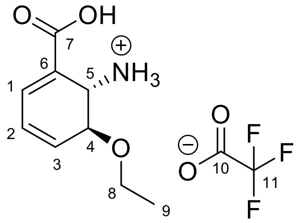

***rac*-27**
***O*-Et-DHHA**

An oven-dried 5 mL round-bottom flask equipped with a Teflon-coated magnetic stirring bar was charged with a colorless solution of 54 mg (190 µmol, 1.0 eq) ethoxy carboxylic acid ***rac*-33** in 500 µL DCM. After the addition of 75 µL TFA (15 % TFA in DCM (v/v)), a brownish solution was immediately formed, which was stirred at RT for 100 min. Subsequently the solvent was removed on a rotary evaporator and the remaining brownish solid was triturated in 500 µL DCM. The resulting brownish suspension was cooled in an ice-water bath to 0 °C, the precipitate was collected by filtration and washed with a small amount of cooled DCM (2 x 100 µL). Finally the brownish, powdery crude material was purified by a second trituration step in 200 µL DCM:MeCN = 6:1 (v/v). It was collected by filtration, washed with cold DCM:MeCN = 6:1 (v/v) (2 x 50 µL) and the resulting colorless powder was dried under high vacuum.

Yield: 18 mg (59 µmol, 31 %); colorless powder.

$C_{11}H_{14}F_3NO_5$ [297.23 g/mol].

m_p = 133-134 °C.

^{1}H-NMR (300.36 MHz, D_2O): δ = 7.44 (d, $^3J_{HH}$ = 5.3 Hz, 1H, H-1), 6.56-6.45 (dd, $^3J_{HH}$ = 9.6 Hz, $^3J_{HH}$ = 5.5 Hz, 1H, H-2), 6.47 (dd, $^3J_{HH}$ = 9.0 Hz, $^3J_{HH}$ = 4.8 Hz, 1H, H-3), 4.51 (d, $^3J_{HH}$ = 2.5 Hz, 1H, H-5), 4.29 (dd, $^3J_{HH}$ = 4.4 Hz, $^3J_{HH}$ = 2.8 Hz, 1H, H-4), 3.82-3.66 (m, 2H, H-8), 1.18 (t, $^3J_{HH}$ = 7.0 Hz, 3H, H-9).

^{13}C-NMR (125.69 MHz, D_2O): δ = 168.4 (C_q, C-7), 162.9 (C_q, q, $^2J_{CF}$ = 35.5 Hz, C-10), 137.3 (C-1), 129.6 (C-3), 126.0 (C-2), 116.2 (C_q, q, $^1J_{CF}$ = 292 Hz, C-11), 71.2 (C-4), 65.2 (C-8), 45.9 (C-5), 14.3 (C-9).

^{19}F-NMR (470.35 MHz, D_2O): δ = -75.7 (decoupled, CF_3).

HRMS: calculated for $C_9H_{13}NO_3^+$: 183.0895; found: 183.0886.

Propyl trifluoromethanesulfonate (34)

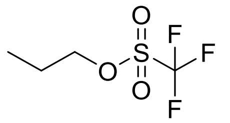

34

An oven-dried, evacuated and argon purged 50 mL Schlenk flask equipped with a Teflon-coated magnetic stirring bar was charged with a colorless solution of 1.33 mL (16.5 mmol, 1.10 eq) pyridine in 15 mL anhydrous DCM and afterwards cooled in an acetone/dry ice bath under argon atmosphere to -20 °C. To this cooled, colorless solution 2.65 mL (15.8 mmol, 1.05 eq) Tf_2O were added via a syringe and septum over a period of 10 min, which resulted in the formation of a colorless suspension. This suspension was additionally stirred at -20 °C in the acetone/dry ice bath under argon for 10 min. Afterwards 1.12 mL (15.0 mmol, 1.00 eq) *n*-PrOH were added dropwise via a syringe over a period of 10 min, the cooling bath was removed and the colorless suspension was stirred under argon at RT for 15 min. It was filtered through a Schlenk frit, the filter cake was washed with anhydrous DCM (2 x 10 mL) and the colorless filtrate was carefully concentrated in the vacuum of an oil pump to approximately 5 mL, which resulted in the precipitation of a colorless solid. Subsequently the colorless suspension was treated with 30 mL anhydrous *n*-pentane and again filtered through a second Schlenk frit in an oven-dried, evacuated and argon purged 80 mL Schlenk flask. The filter cake was washed with anhydrous *n*-pentane (2 x 5 mL) and the solvent of the colorless filtrate was carefully removed in the vacuum of an oil pump. Finally, the brownish, oily residue was dried at 5 mbar for 5 min. It was immediately used in the propylation step without further purification.

Yield: 2.88 g (15.0 mmol, 100 %); brownish, viscous liquid.

Ethyl (5*S*,6*S*)-6-((*tert*-butoxycarbonyl)amino)-5-propoxycyclohexa-1,3-diene-1-carboxylate (*rac*-35)

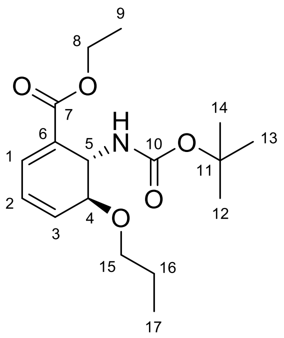

***rac*-35**

An oven-dried, evacuated and argon purged 50 mL two-necked round-bottom flask equipped with a Teflon-coated magnetic stirring bar and gas inlet adapter was charged with a colorless solution of 425 mg (1.50 mmol, 1.0 eq) racemic ester ***rac*-8** in 10 mL anhydrous Et_2O and to the colorless solution 695 mg (2.00 mmol, 2.0 eq) Ag_2O as well as 3.0 g 3 Å MS were added in an argon counter flow, respectively, which resulted in the formation of a black suspension. In parallel 2.88 g (15.0 mmol, 10.0 eq) of the freshly prepared propyl trifluoromethanesulfonate (**34**) were dissolved in 7.5 mL anhydrous Et_2O under argon in the same Schlenk flask, in which this compound was dried. This solution was added to the black suspension via a syringe and septum in one portion, the Schlenk flask was rinsed with anhydrous Et_2O (1 x 2.5 mL) and the black suspension was vigorously stirred in the closed flask at RT for 120 h. Afterwards it was filtered through a pad of Celite® (diameter: 3.0 cm, height: 4.0 cm), the filter cake was washed with EtOAc (4 x 50 mL) and the solvent was removed on a rotary evaporator. Finally, the orange, viscous crude material was purified via flash column chromatography (80 g SiO_2, 25.0 x 2.5 cm, eluent: cyclohexane:EtOAc = 9:2 (v/v), R_f = 0.24) and the resulting yellowish, highly viscous liquid was dried under high vacuum.

Yield: 56 mg (172 µmol, 11 %); yellowish, highly viscous liquid.

$C_{17}H_{27}NO_5$ [325.40 g/mol].

R_f = 0.70 (cyclohexane:EtOAc = 1:1 (v/v)).

^{1}H-NMR (300.36 MHz, $CDCl_3$): δ =7.16 (d, $^3J_{HH}$ = 4.9 Hz, 1H, H-1), 6.31-6.17 (m, 2H, H-2, H-3), 4.89 (d, $^3J_{HH}$ = 7.1 Hz, 1H, H-5), 4.33-4.13 (m, 3H, H-8, NH), 3.94 (d, $^3J_{HH}$ = 2.8 Hz, 1H, H-4), 3.77-3.66 (m, 1H, H-15a), 3.60-3.47 (m, 1H, H-15b), 1.57 (h, $^3J_{HH}$ = 7.2 Hz, 2H, H-16), 1.43 (s, 9H, H-12, H-13, H-14), 1.29 (t, $^3J_{HH}$ = 7.1 Hz, 3H, H-9), 0.89 (t, $^3J_{HH}$ = 7.4 Hz, 3H, H-17).

^{13}C-NMR (75.53 MHz, $CDCl_3$): δ = 166.0 (C_q, C-7), 155.2 (C_q, C-10), 133.6 (C-1), 131.0 (C-3), 128.0 (C_q, C-6), 125.2 (C-2), 79.8 (C_q, C-11), 74.6 (C-4), 71.0 (C-15), 60.9 (C-8), 46.0 (C-5), 28.5 (C-12, C-13, C-14), 23.4 (C-16), 14.3 (C-9), 10.6 (C-17).

HRMS: calculated for $C_{17}H_{27}NO_5^+$: 325.1889; found: 325.1900.

(5*S*,6*S*)-6-((*tert*-Butoxycarbonyl)amino)-5-propoxycyclohexa-1,3-diene-1-carboxylic acid (*rac*-36)

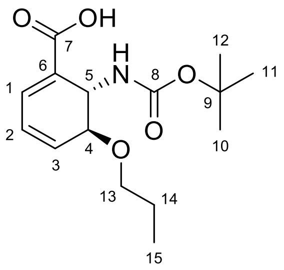

***rac*-36**

A 5 mL round-bottom flask equipped with a Teflon-coated magnetic stirring bar was charged with a yellowish solution of 49 mg (150 µmol, 1.0 eq) propoxy ester ***rac*-35** in 1.2 mL THF. After the addition of 70 µL H_2O as well as 38 mg (900 µmol, 6.0 eq) $LiOH.H_2O$, the resulting yellowish, milky solution was stirred in the closed flask at RT for 7 d. Afterwards it was concentrated on a rotary evaporator and the oily residue was dissolved in 5.0 mL H_2O. The yellowish aqueous phase was washed with EtOAc (2 x 5.0 mL), the product from the organic phase reextracted with H_2O (2 x 500 µL) and the combined yellowish aqueous layers subsequently acidified with 750 µL saturated $KHSO_4$ to pH 1-2. The product was extracted with EtOAc (3 x 5.0 mL), the combined yellowish organic phases were dried over $MgSO_4$, filtered and the solvent was removed on a rotary evaporator. Finally the crude material was purified via flash column chromatography (6.0 g SiO_2, 23.0 x 0.8 cm, eluent: cyclohexane:EtOAc:AcOH = 1000:5000:1 (v/v/v), R_f = 0.26) and the resulting yellowish, viscous liquid was dried under high vacuum.

Yield: 44 mg (148 µmol, 99 %); yellowish, viscous liquid.

$C_{15}H_{23}NO_5$ [297.35 g/mol].

R_f = 0.58 (EtOAc:AcOH = 1000:1 (v/v)).

^{1}H-NMR (300.36 MHz, DMSO-d$_6$): δ = 12.31 (bs, 1H, COOH), 7.00 (d, $^3J_{HH}$ = 4.9 Hz, 1H, H-1), 6.74 (d, $^3J_{HH}$ = 7.0 Hz, 1H, NH), 6.29-6.19 (m, 2H, H-2, H-3), 4.54 (d, $^3J_{HH}$ = 7.0 Hz, 1H, H-5), 3.76 (d, $^3J_{HH}$ = 3.5 Hz, 1H, H-4), 3.58-3.51 (m, 1H, H-13), 3.46-3.41 (m, 1H, H-13), 1.51-1.42 (m, 2H, H-14), 1.37 (s, 9H, H-10, H-11, H-12), 0.82 (t, $^3J_{HH}$ = 7.3 Hz, 3H, H-15).

^{13}C-NMR (75.53 MHz, DMSO-d$_6$): δ =167.2 (C$_q$, C-7), 155.1 (C$_q$, C-8), 133.2 (C-1), 130.3 (C-3), 127.3 (C$_q$, C-6), 125.4 (C-2), 77.9 (C$_q$, C-9), 73.9 (C-4), 69.4 (C-13), 46.0 (C-5), 28.2 (C-10, C-11, C-12), 22.8 (C-14), 10.5 (C-15).

HRMS: calculated for $C_{15}H_{23}NO_5^+$: 297.1576; found: 297.1576.

(1*S*,6*S*)-2-Carboxy-6-propoxycyclohexa-2,4-diene-1-ammonium 2,2,2-trifluoroacetate (*rac*-28) (*O*-*n*Pr-DHHA)

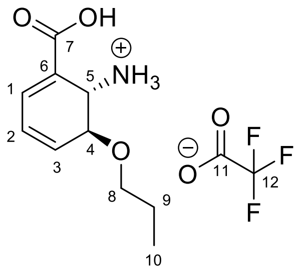

***rac*-28**
***O*-*n*Pr-DHHA**

An oven-dried 5 mL round-bottom flask equipped with a Teflon-coated magnetic stirring bar was charged with a yellowish solution of 30 mg (100 µmol, 1.0 eq) propoxy carboxylic acid ***rac*-36** in 300 µL DCM. After the addition of 45 µL TFA (15 % TFA in DCM (v/v)), a brownish solution was immediately formed, which was stirred at RT for 120 min. Subsequently the solvent was removed on a rotary evaporator and the remaining brownish solid was triturated in 500 µL DCM:*n*-pentane = 1:1 (v/v). The resulting brownish suspension was cooled in an ice-water bath to 0 °C, the precipitate was collected by filtration and washed with cold DCM:*n*-pentane = 1:1 (v/v) (2 x 100 µL). Finally the brownish powder was dried under high vacuum.

Yield: 17 mg (55 µmol, 55 %); brownish powder.

$C_{12}H_{16}F_3NO_5$ [311.26 g/mol].

m_p = 109-110 °C.

^{1}H-NMR (300.36 MHz, DMSO-d$_6$): δ = 12.95 (bs, 1H, COOH), 8.19 (bs, 3H, NH_3^+), 7.24 (dd, $^3J_{HH}$ = 4.2 Hz, $^4J_{HH}$ = 1.5 Hz, 1H, H-1), 6.46-6.38 (m, 2H, H-2, H-3), 4.17 (s, 1H, H-5), 4.04 (bs, 1H, H-4), 3.52-3.42 (m, 2H, H-8), 1.47 (h, $^3J_{HH}$ = 7.0 Hz, 2H, H-9), 0.82 (t, $^3J_{HH}$ = 7.4 Hz, 3H, H-10).

^{13}C-NMR (75.53 MHz, DMSO-d$_6$): δ = 166.5 (C$_q$, C-7), 157.9 (C$_q$, q, $^2J_{CF}$ = 30.7 Hz, C-11), 135.2 (C-1), 130.2 (C-3), 125.3 (C-2), 117.3 (C$_q$, q, $^1J_{CF}$ = 301 Hz, C-12), 70.7 (C-4), 69.6 (C-8), 45.7 (C-5), 22.5 (C-9), 10.3 (C-10).

^{19}F-NMR (470.35 MHz, DMSO-d$_6$): δ = -73.5 (decoupled, CF$_3$).

HRMS: calculated for $C_{10}H_{15}NO_3^+$: 197.1052; found: 197.1060.

NMR-spectra of synthetic compounds

^{1}H- and ^{13}C-NMR-spectrum of ethyl 2-hydroxy-3-nitropropanoate (10)

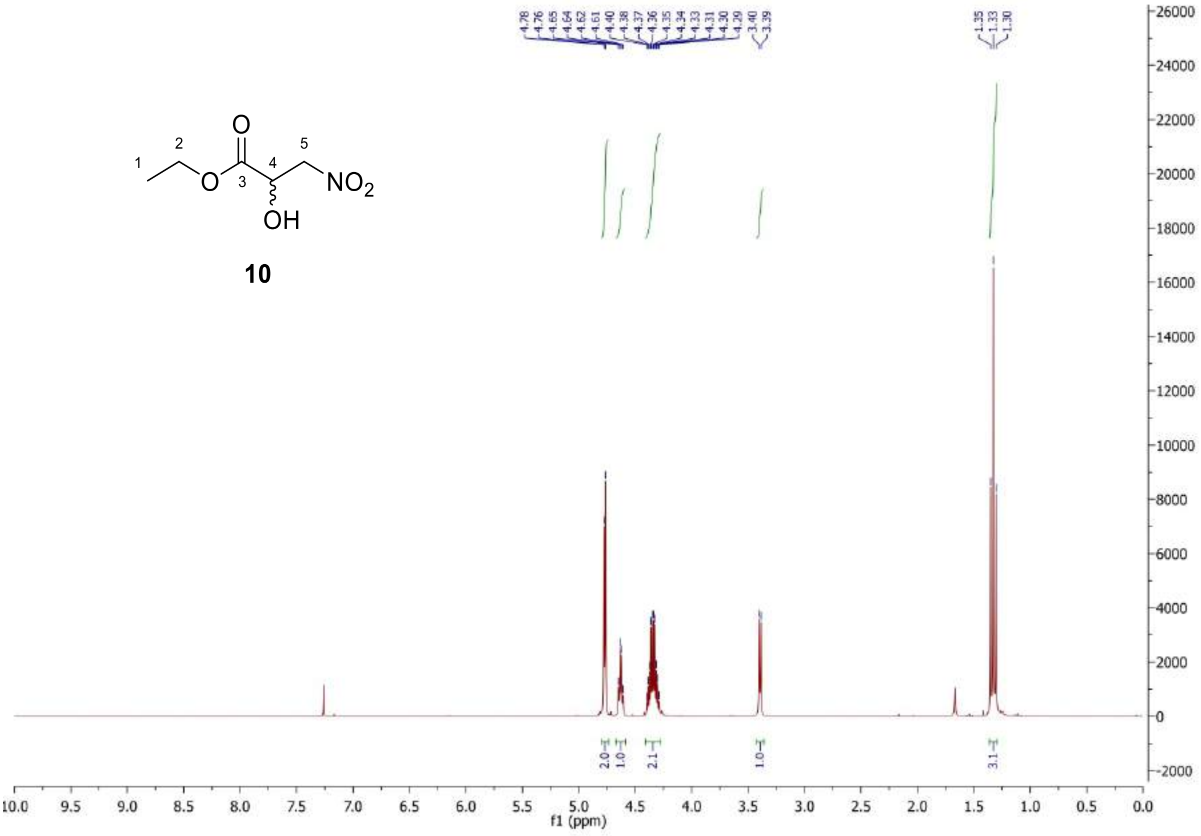

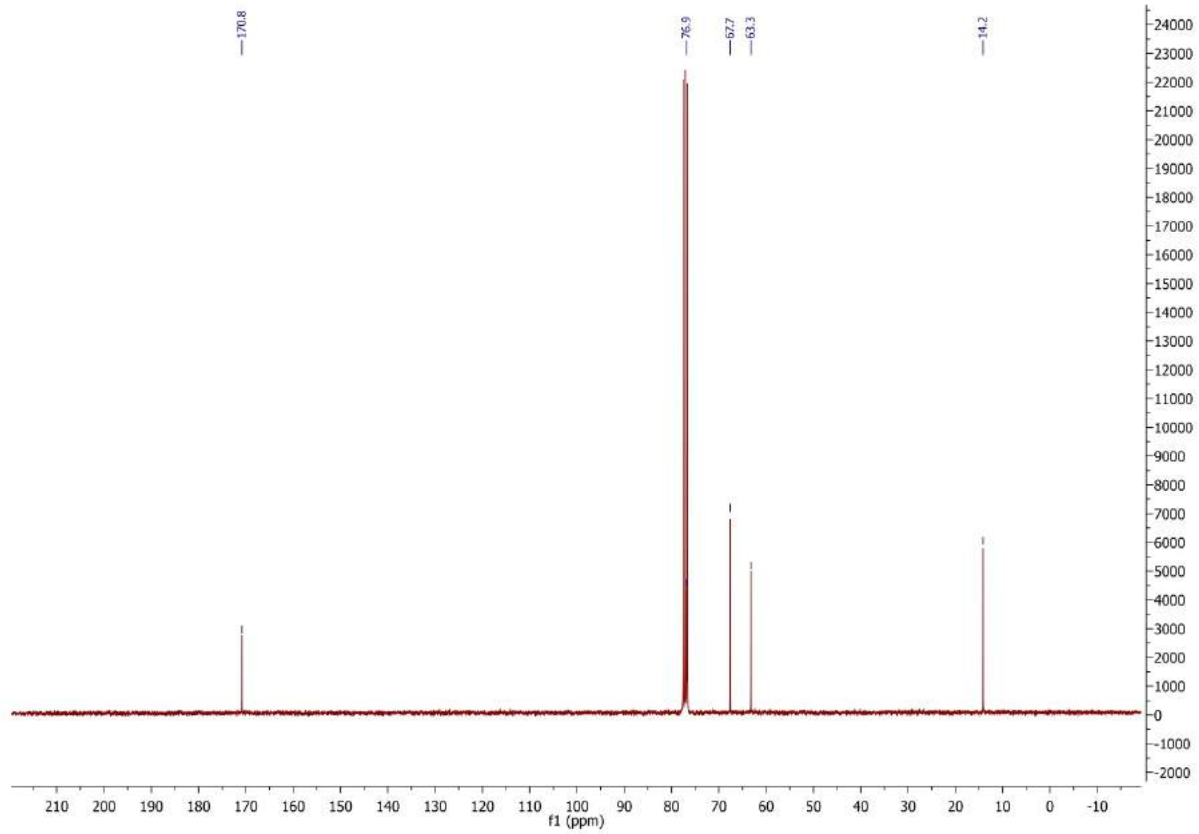

^{1}H- and ^{13}C-NMR-spectrum of ethyl (*E*)-3-nitroacrylate (3)

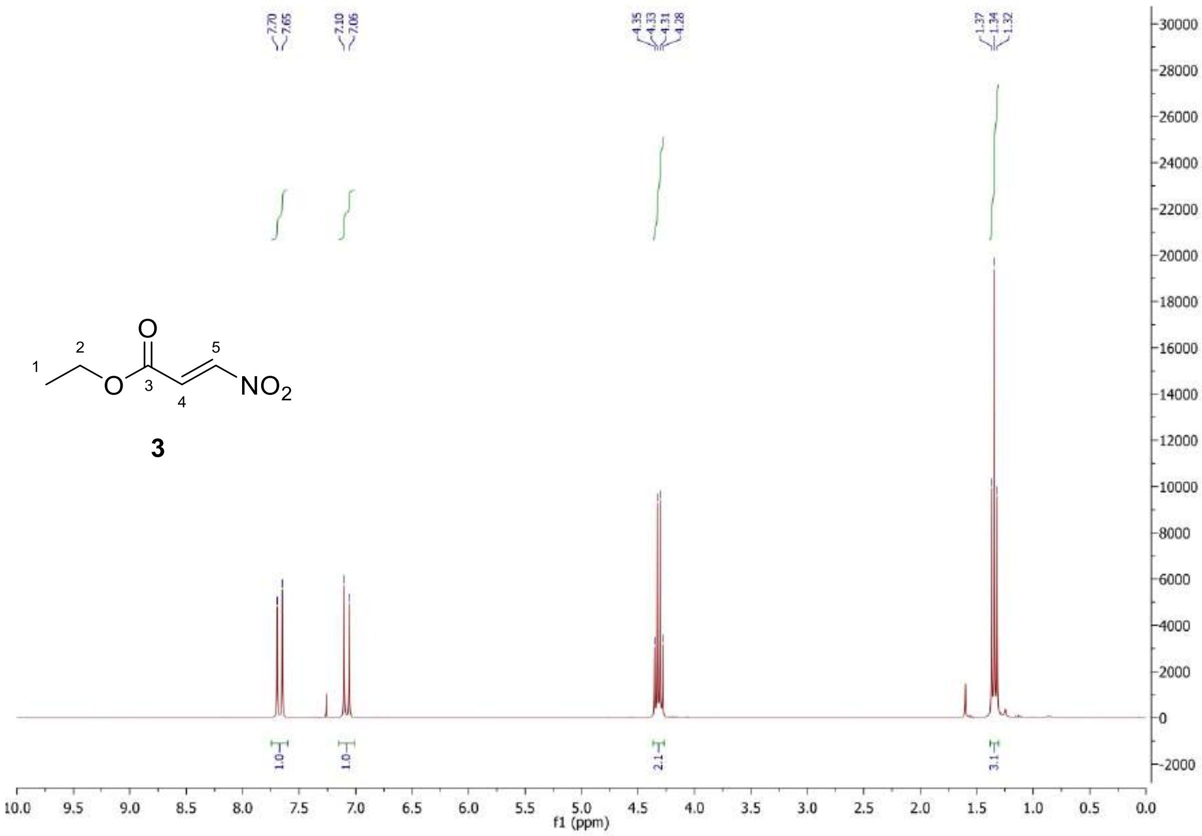

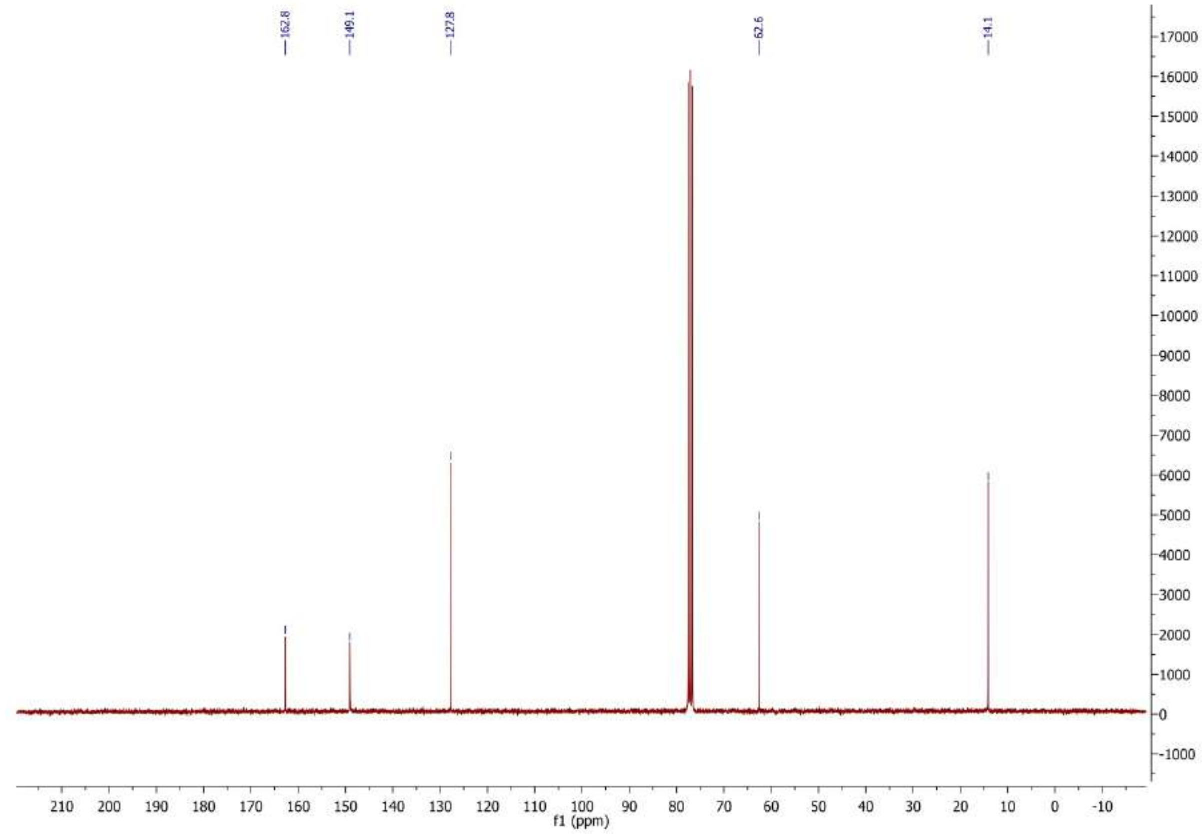

^{1}H- and APT-NMR-spectrum of ethyl (1*R*,2*S*,3*S*,4*S*)-3-nitro-7-oxabicyclo[2.2.1]hept-5-ene-2-carboxylate (4)

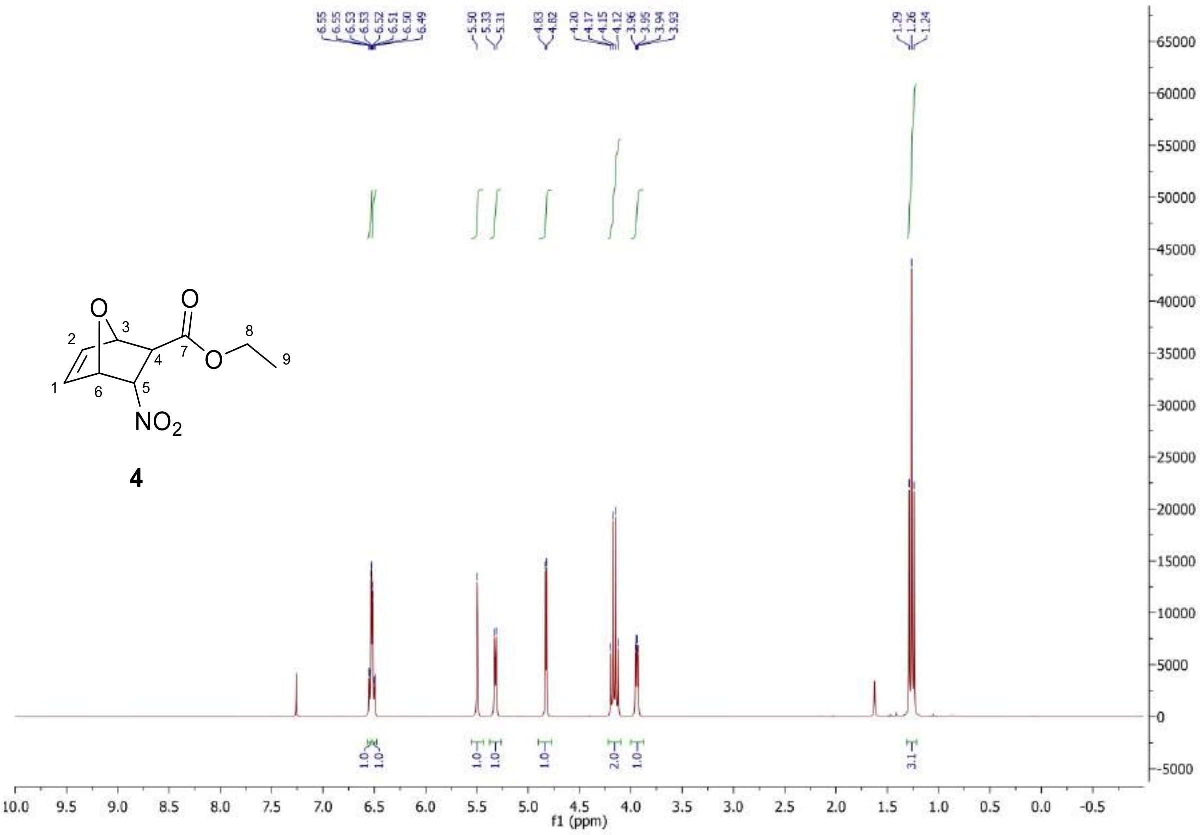

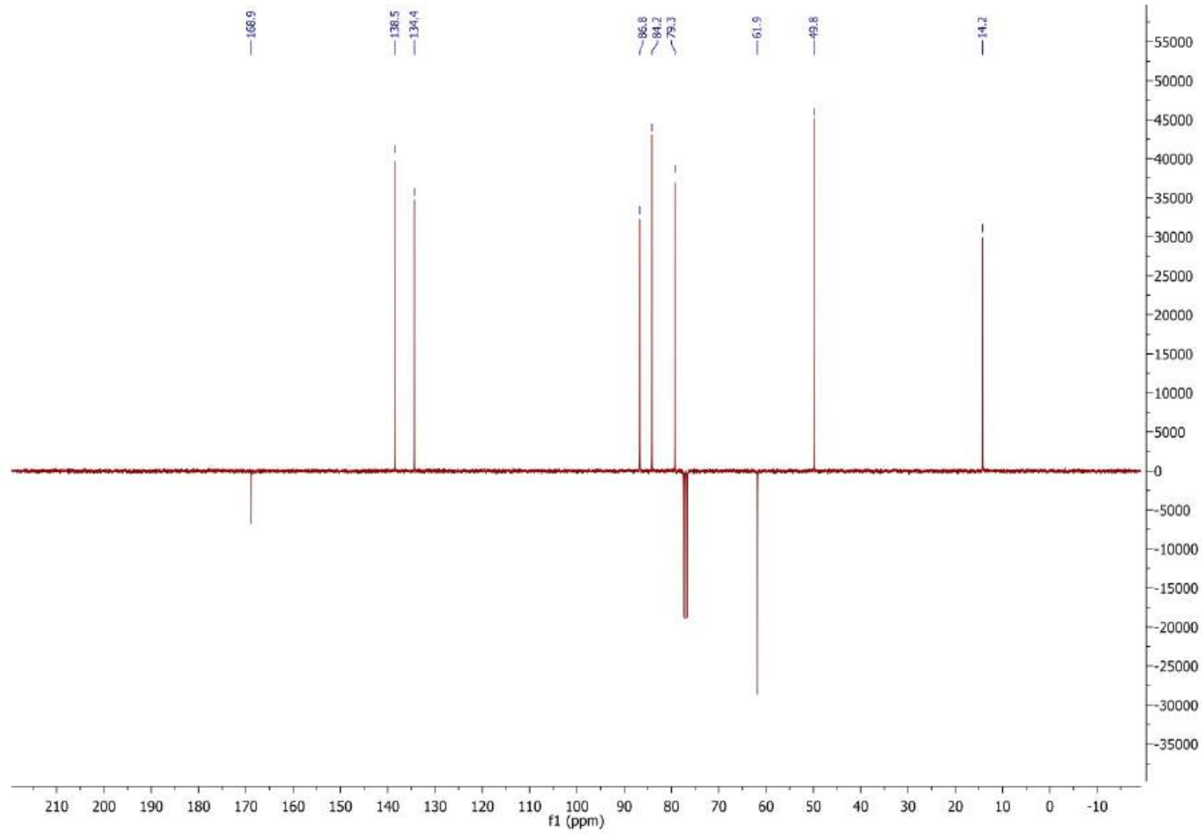

^{1}H- and APT-NMR-spectrum of ethyl (1*S*,2*S*,3*S*,4*R*)-3-nitro-7-oxabicyclo[2.2.1]hept-5-ene-2-carboxylate (11)

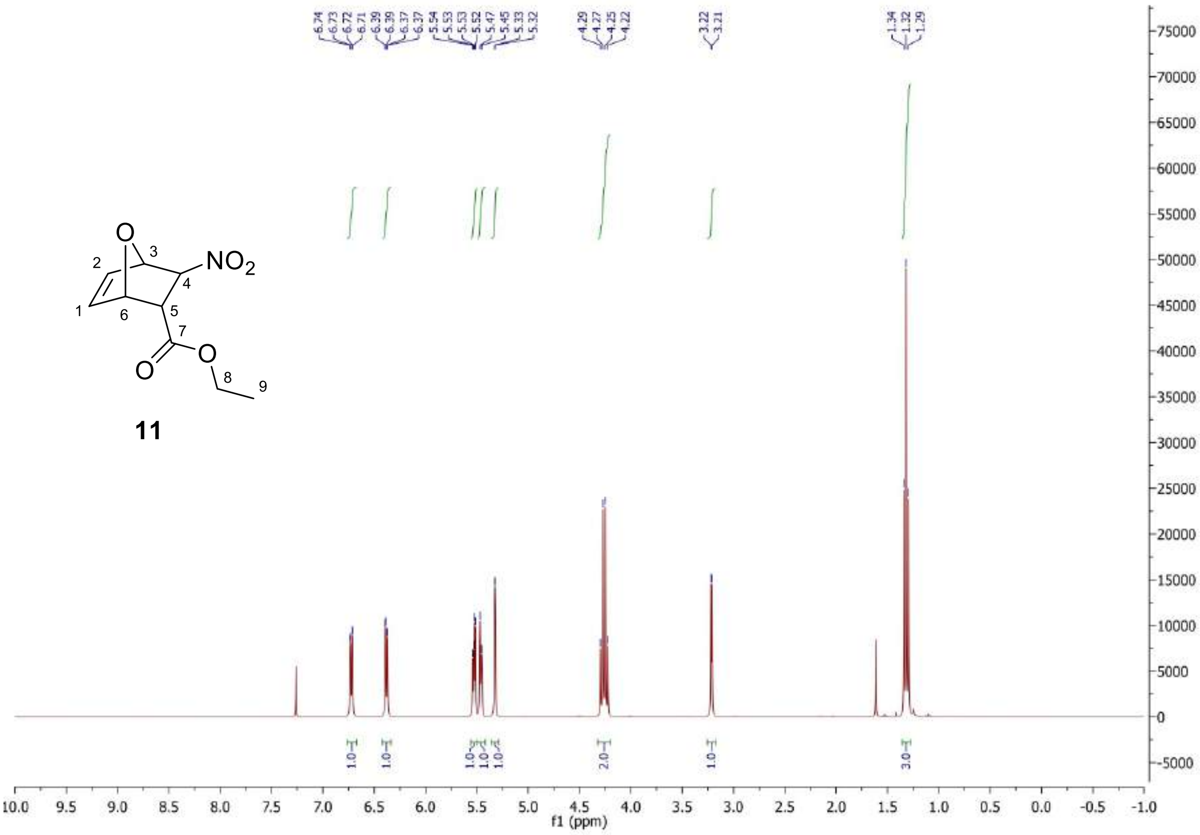

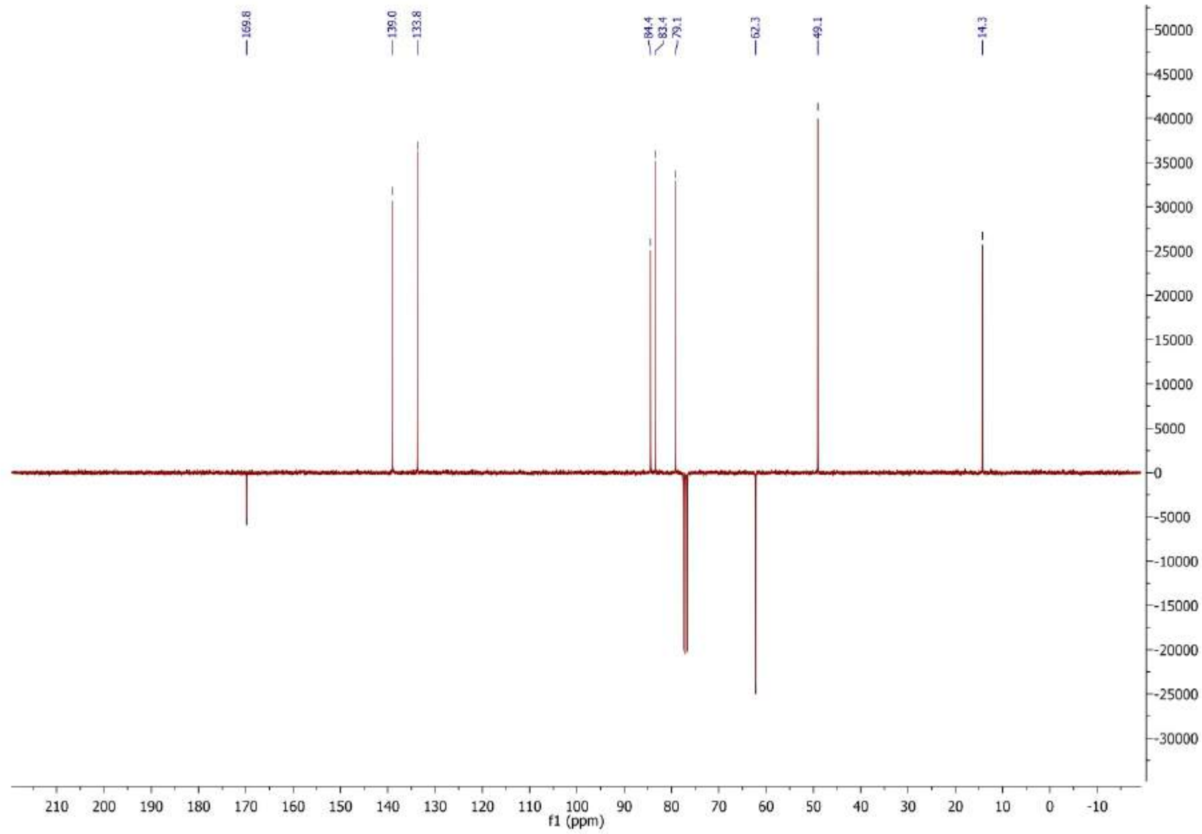

^{1}H- and ^{13}C-NMR-spectrum of ethyl (1*R*,2*S*,3*S*,4*S*)-3-((*tert*-butoxycarbonyl)amino)-7-oxabicyclo[2.2.1]-hept-5-ene-2-carboxylate (5)

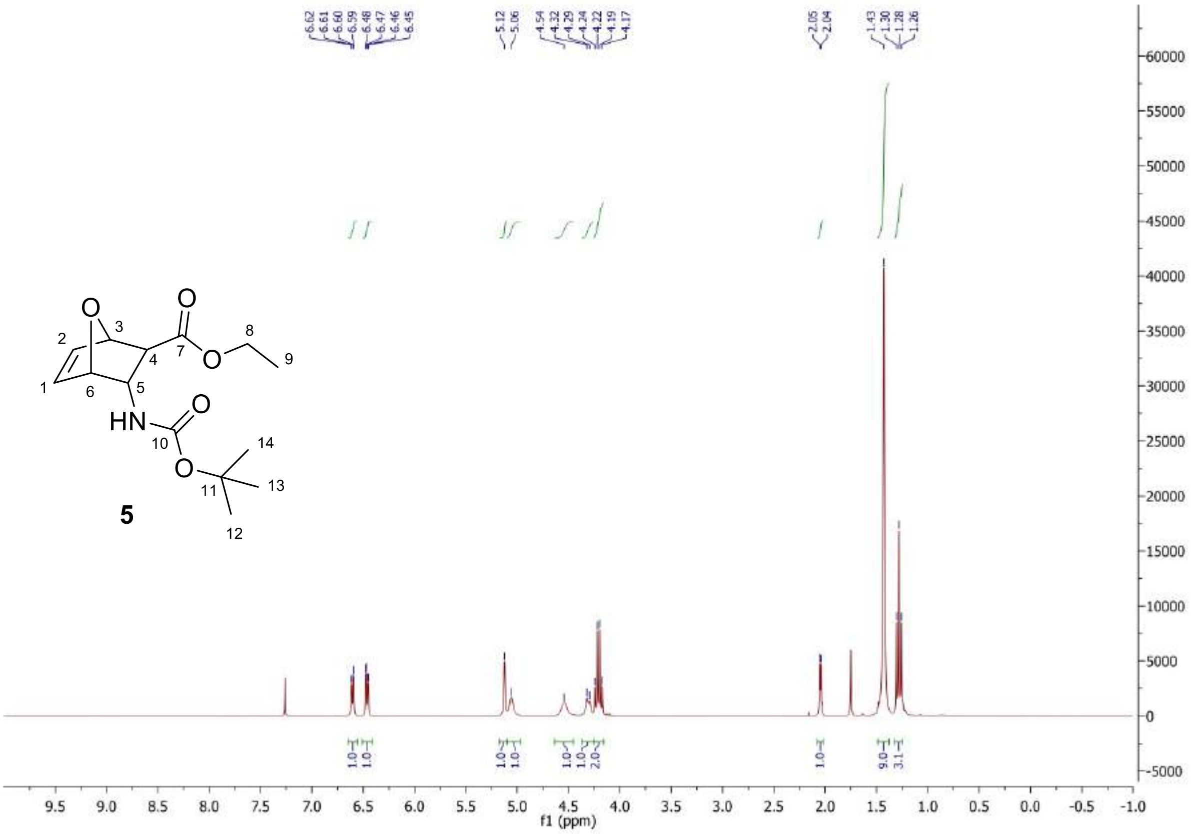

5

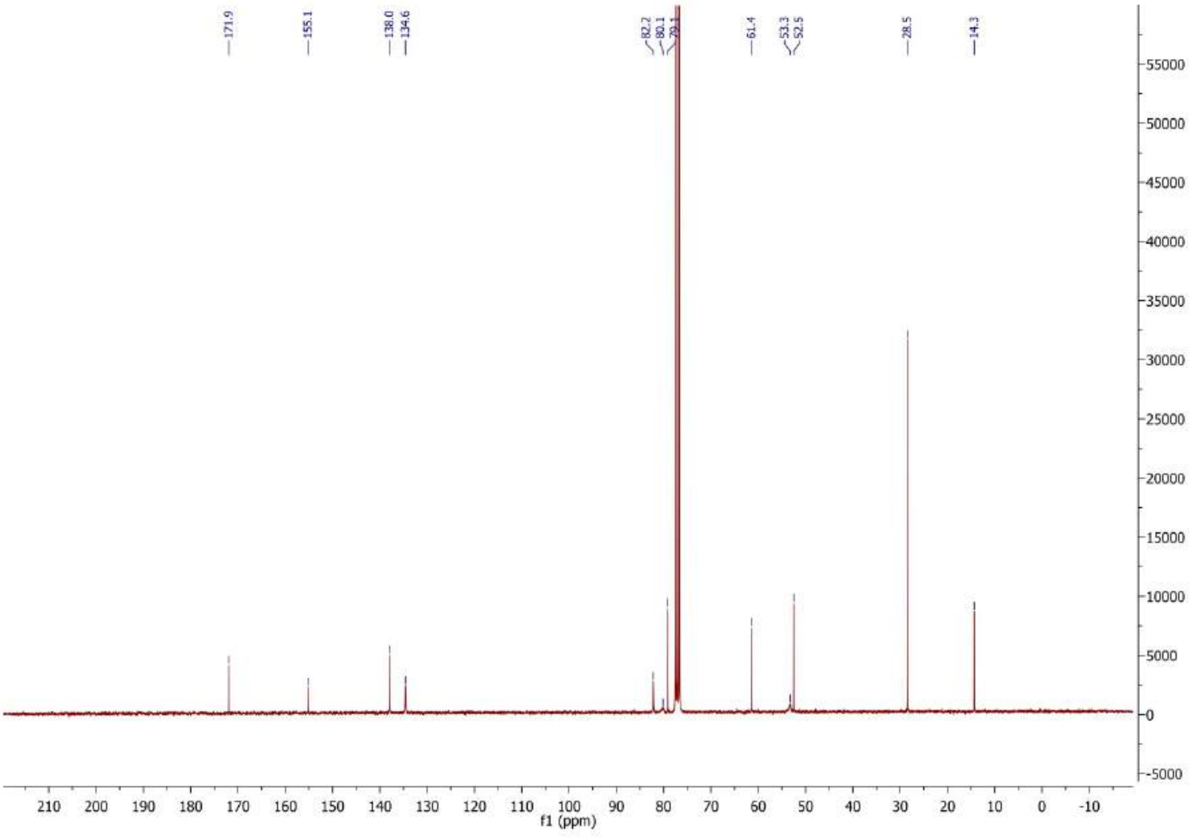

^{1}H- and ^{13}C-NMR-spectrum of (1*R*,2*S*,3*S*,4*S*)-3-((*tert*-butoxycarbonyl)amino)-7-oxabicyclo[2.2.1]hept-5-ene-2-carboxylic acid (6)

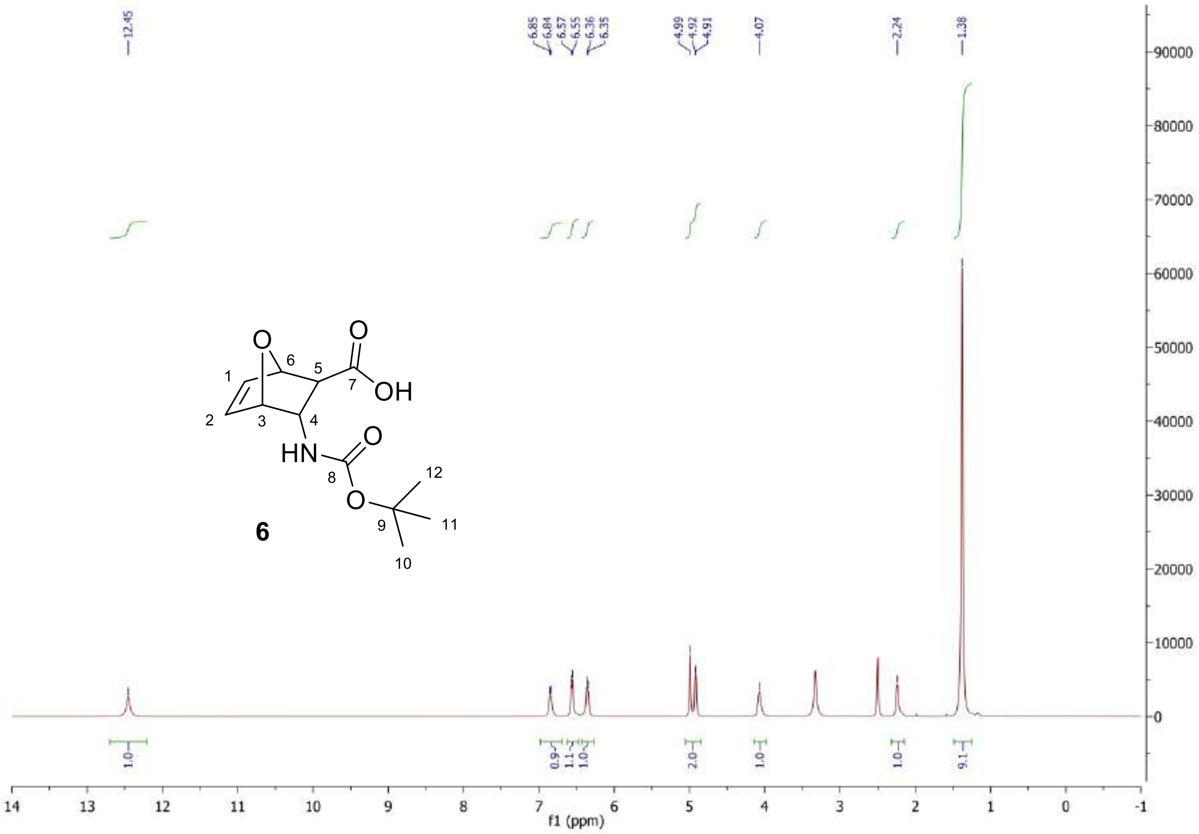

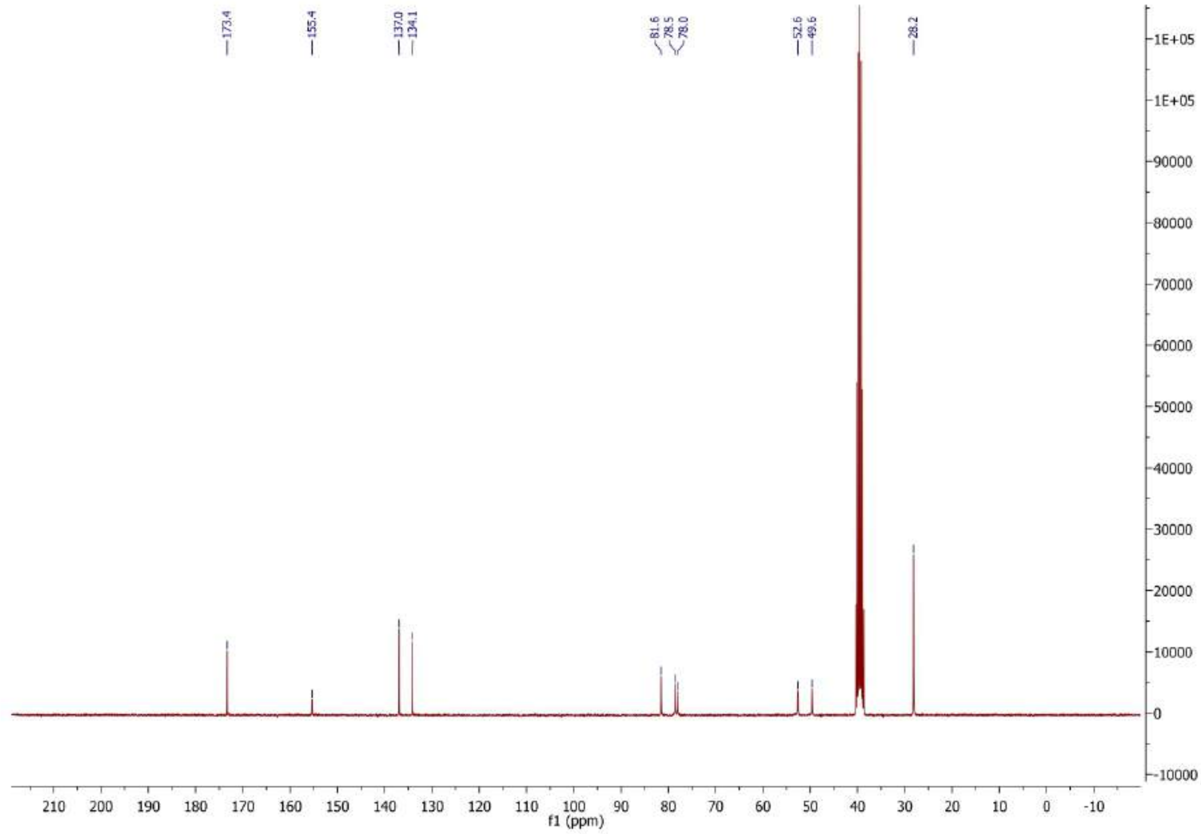

^{1}H- and ^{13}C-NMR-spectrum of ethyl (5*S*,6*S*)-6-((*tert*-butoxycarbonyl)amino)-5-hydroxycyclohexa-1,3-diene-1-carboxylate (8)

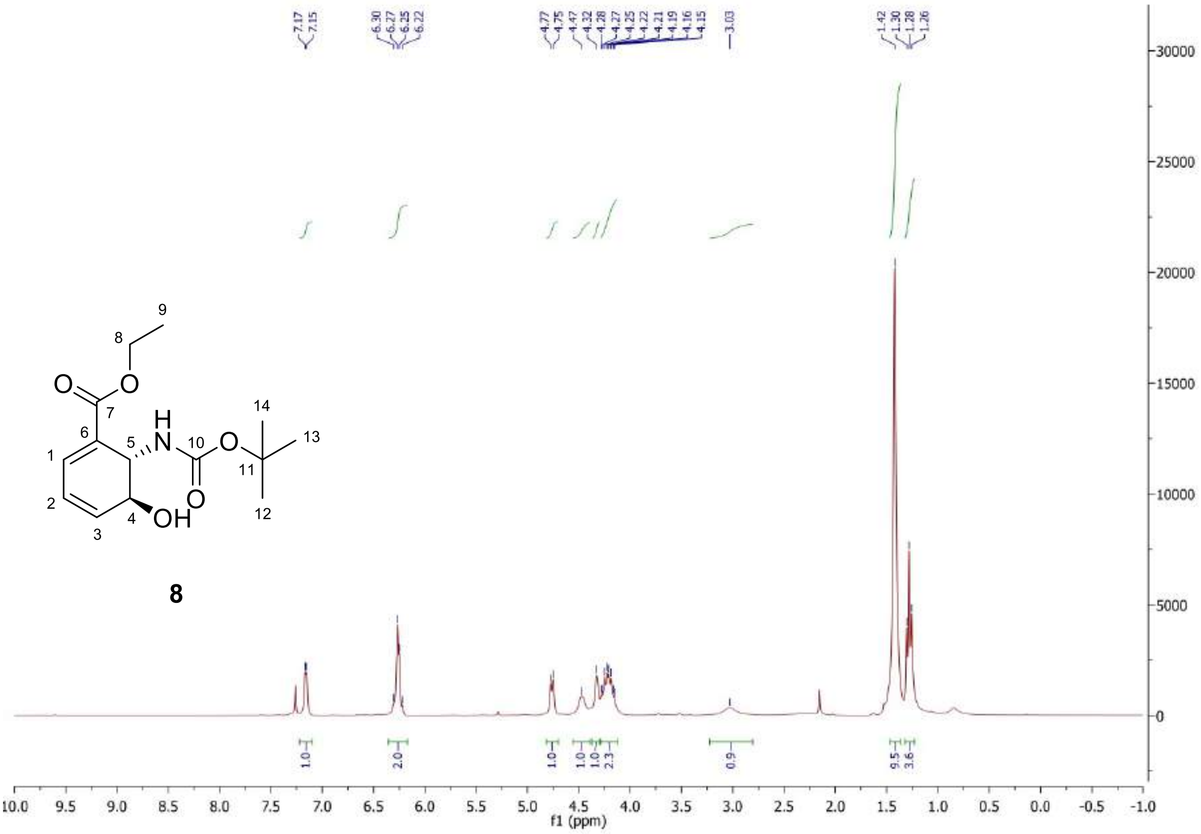

8

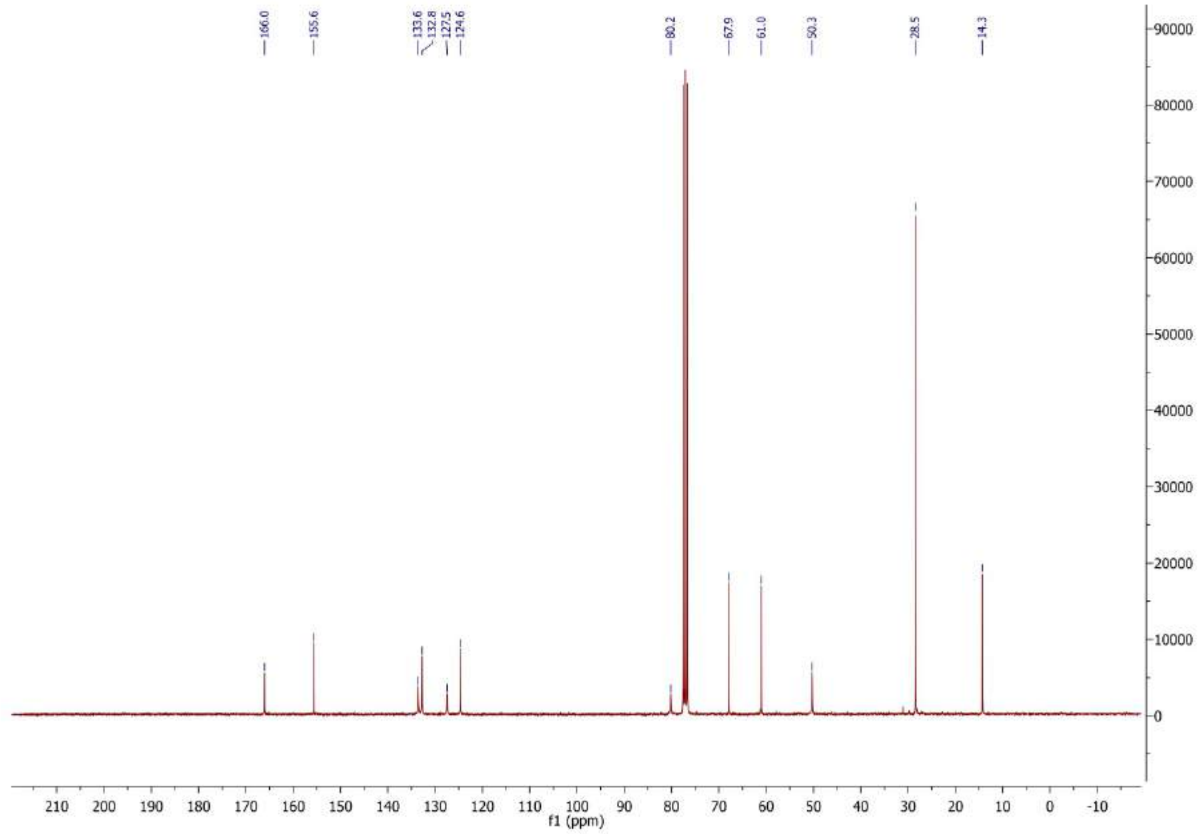

^{1}H- and ^{13}C-NMR-spectrum of (5*S*,6*S*)-6-((*tert*-butoxycarbonyl)amino)-5-hydroxycyclohexa-1,3-diene-1-carboxylic acid (13)

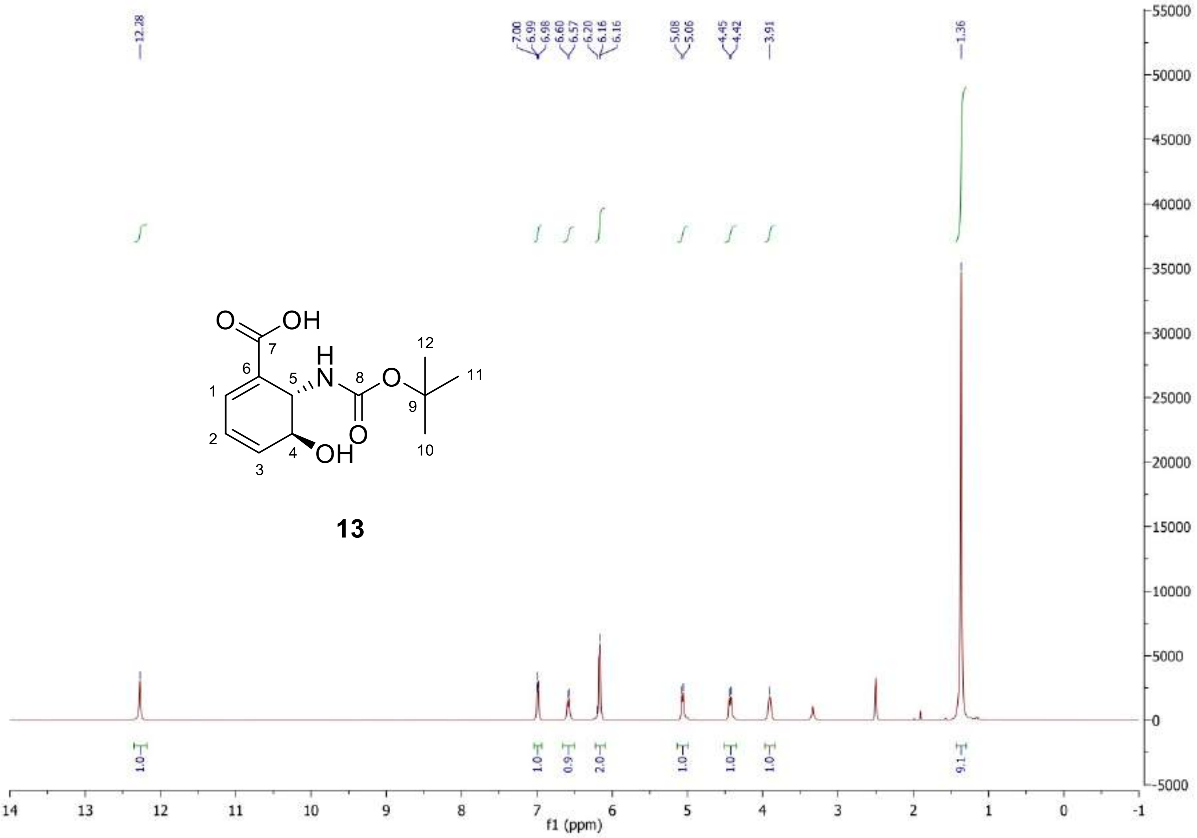

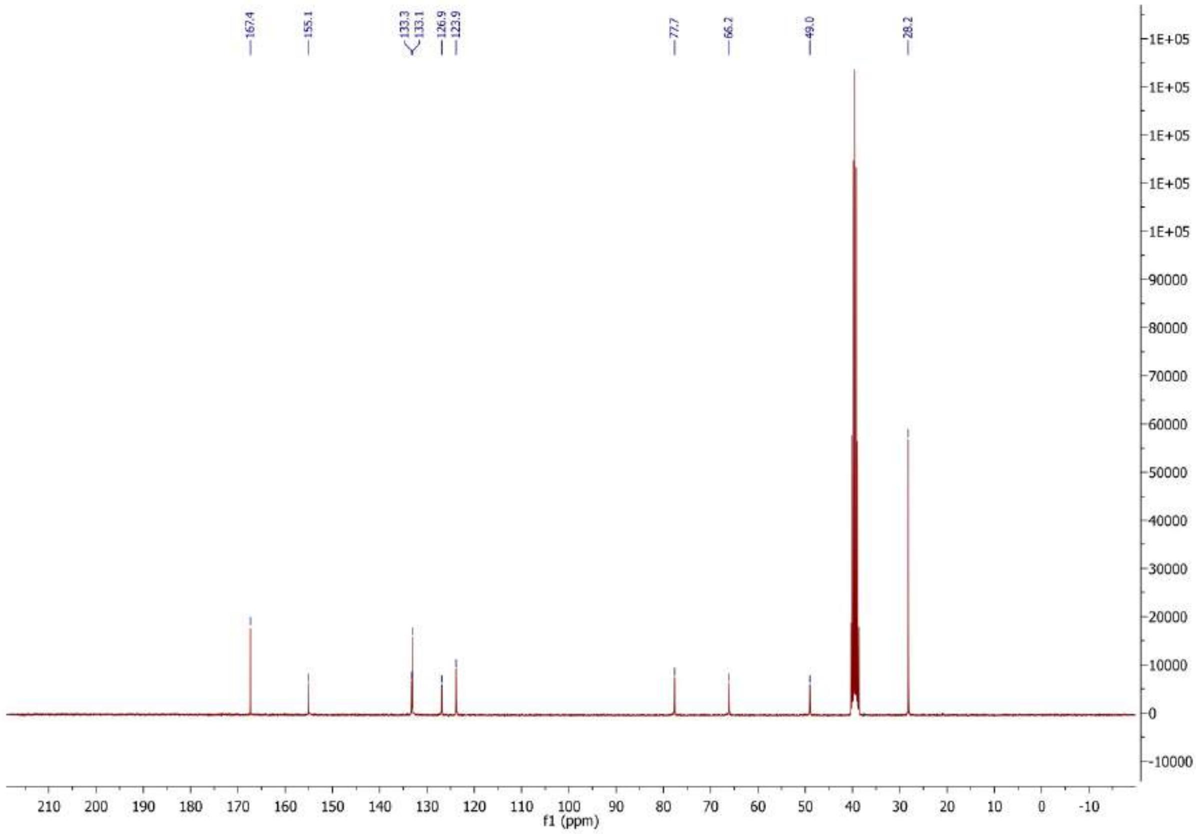

^{1}H-, ^{13}C- and ^{19}F-NMR-spectrum of (1*S*,6*S*)-2-carboxy-6-hydroxycyclohexa-2,4-diene-1-ammonium 2,2,2-trifluoroacetate (9) (DHHA)

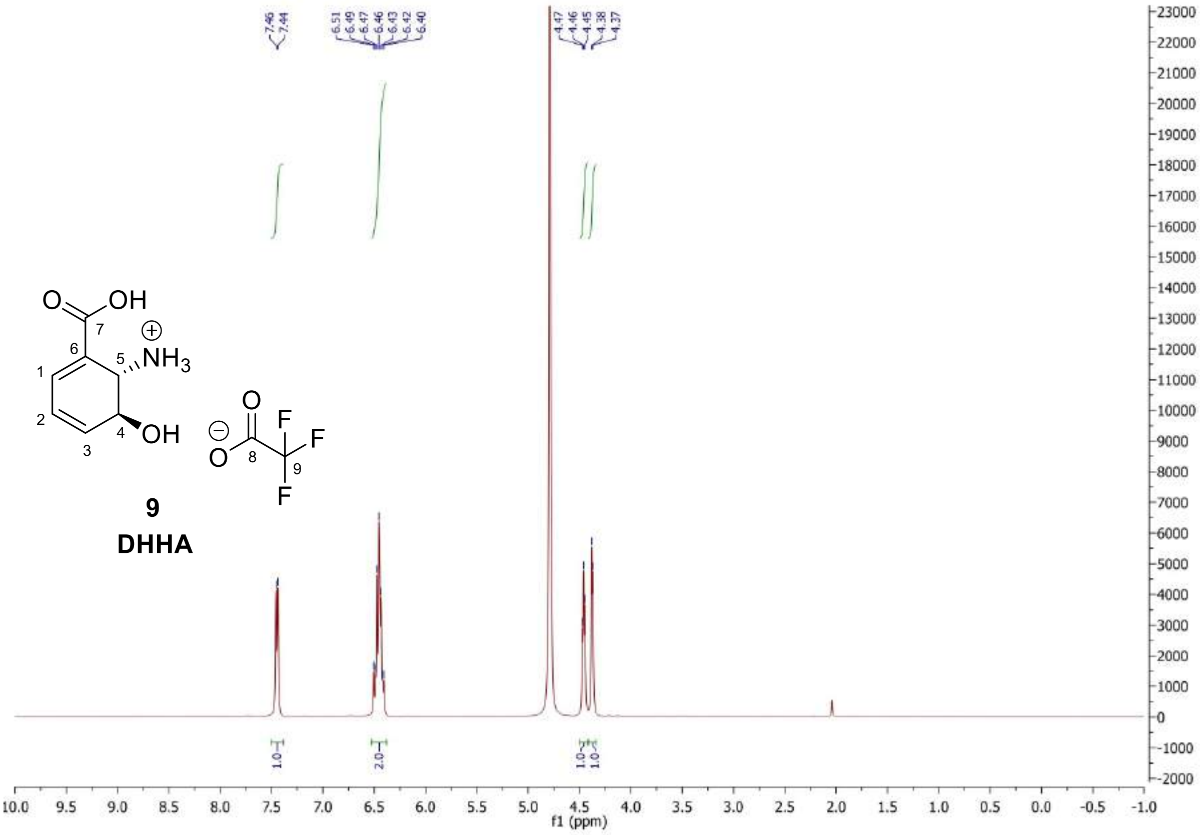

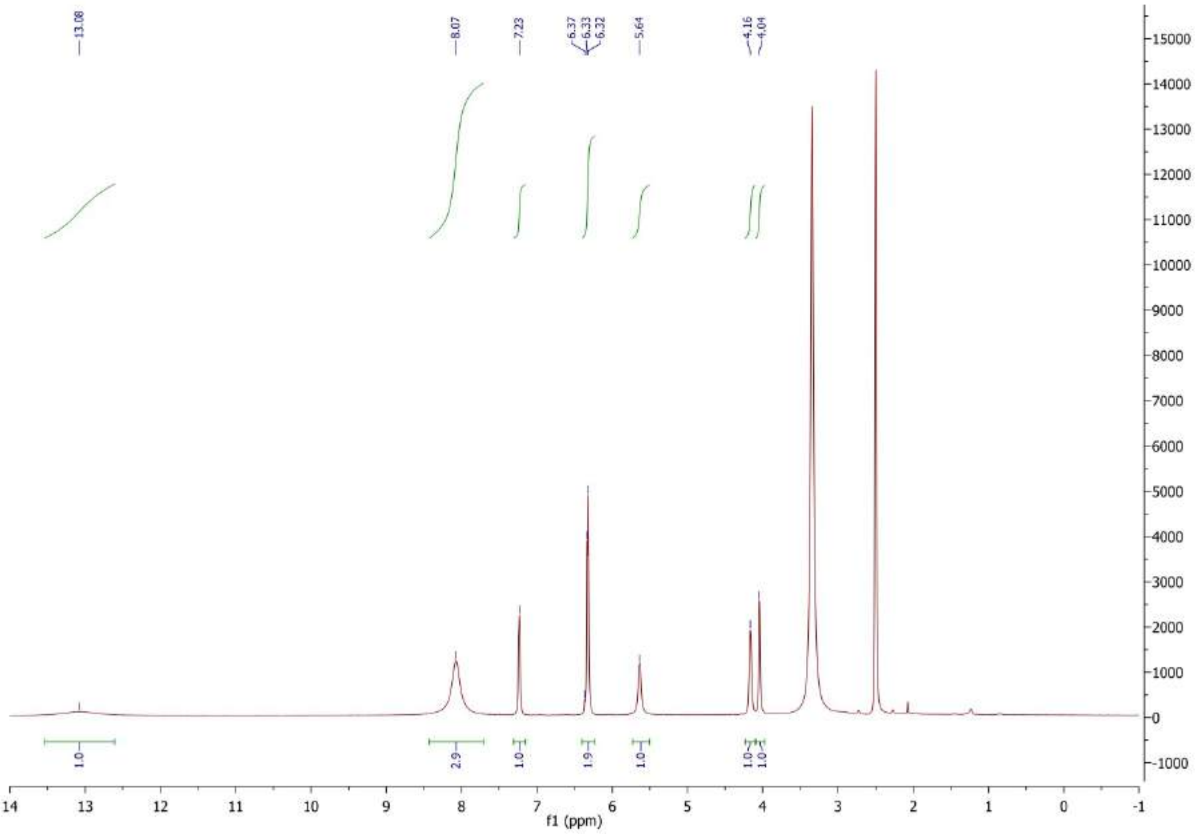

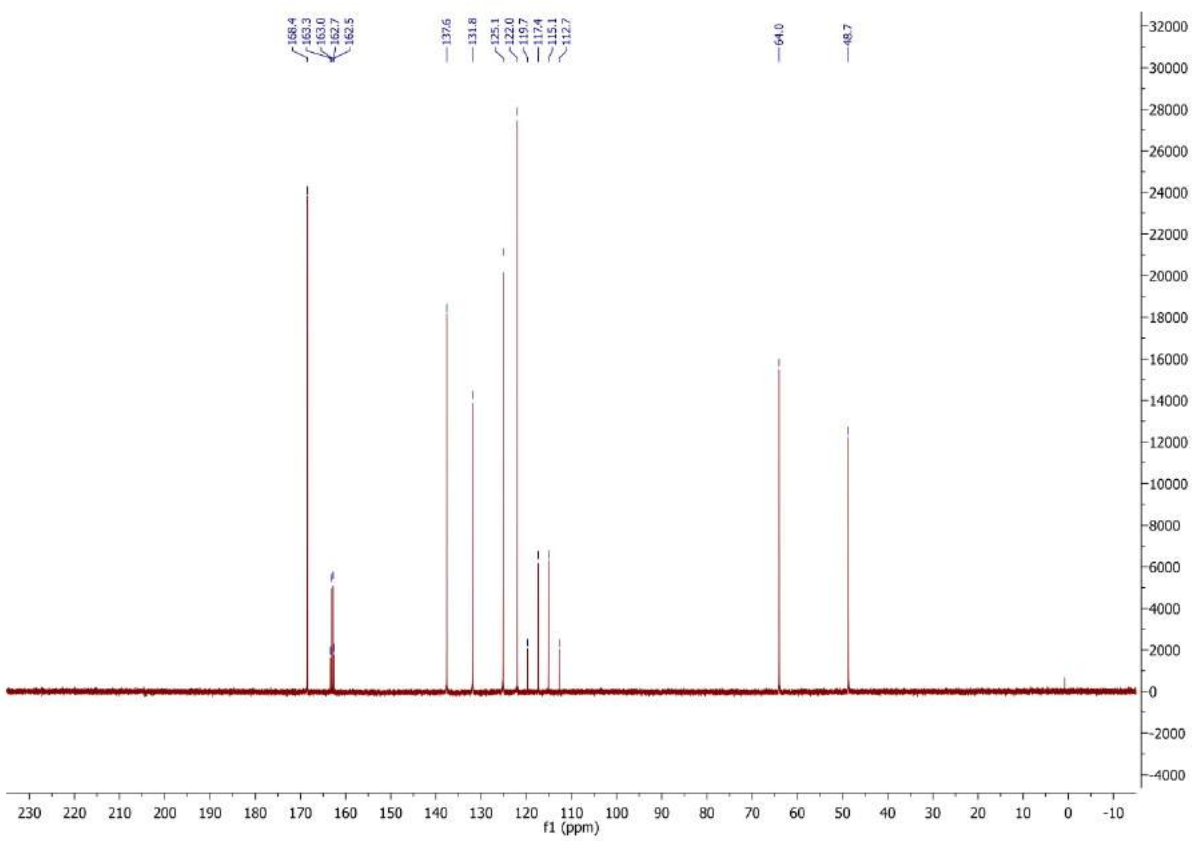
168.4
163.3
163.0
162.7
162.5
137.6
131.8
125.1
122.0
119.7
117.4
115.1
112.7
64.0
48.7
f1 (ppm)

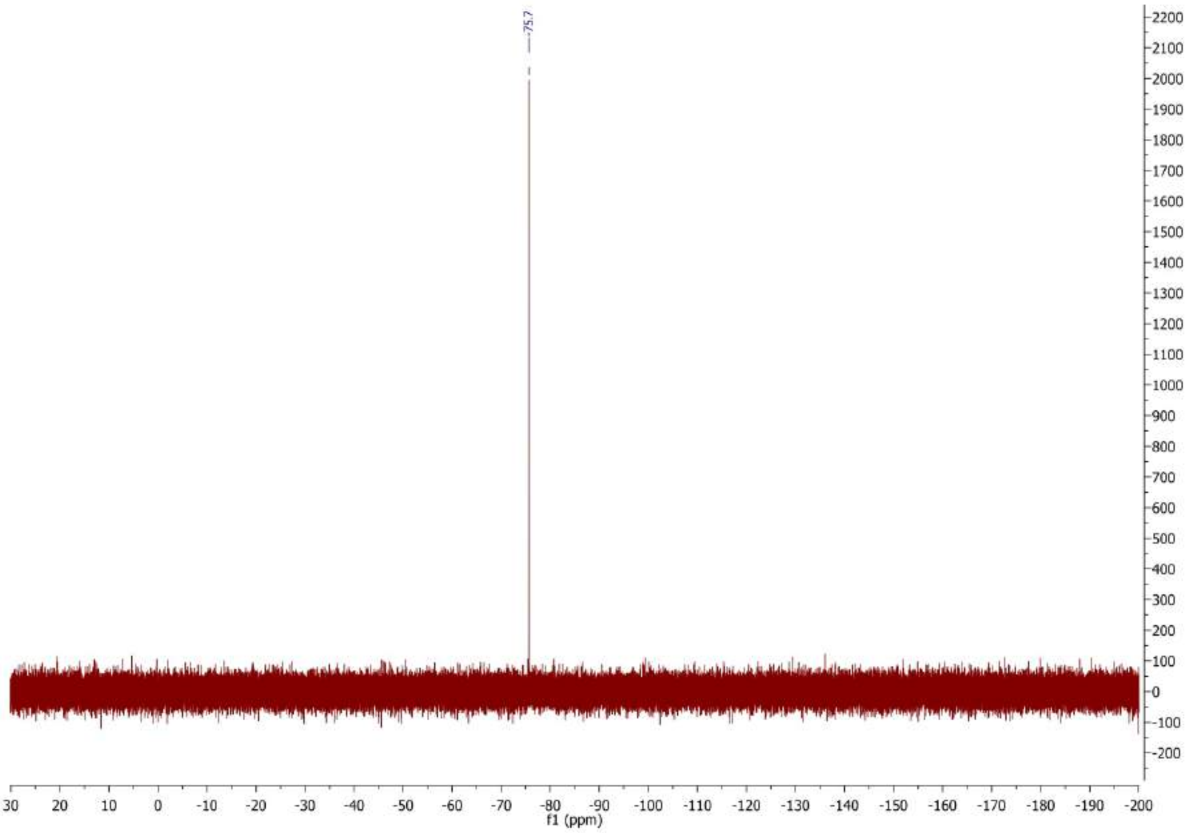
-75.7
f1 (ppm)

^{1}H- and ^{13}C-NMR-spectrum of 2-bromofuran (15)

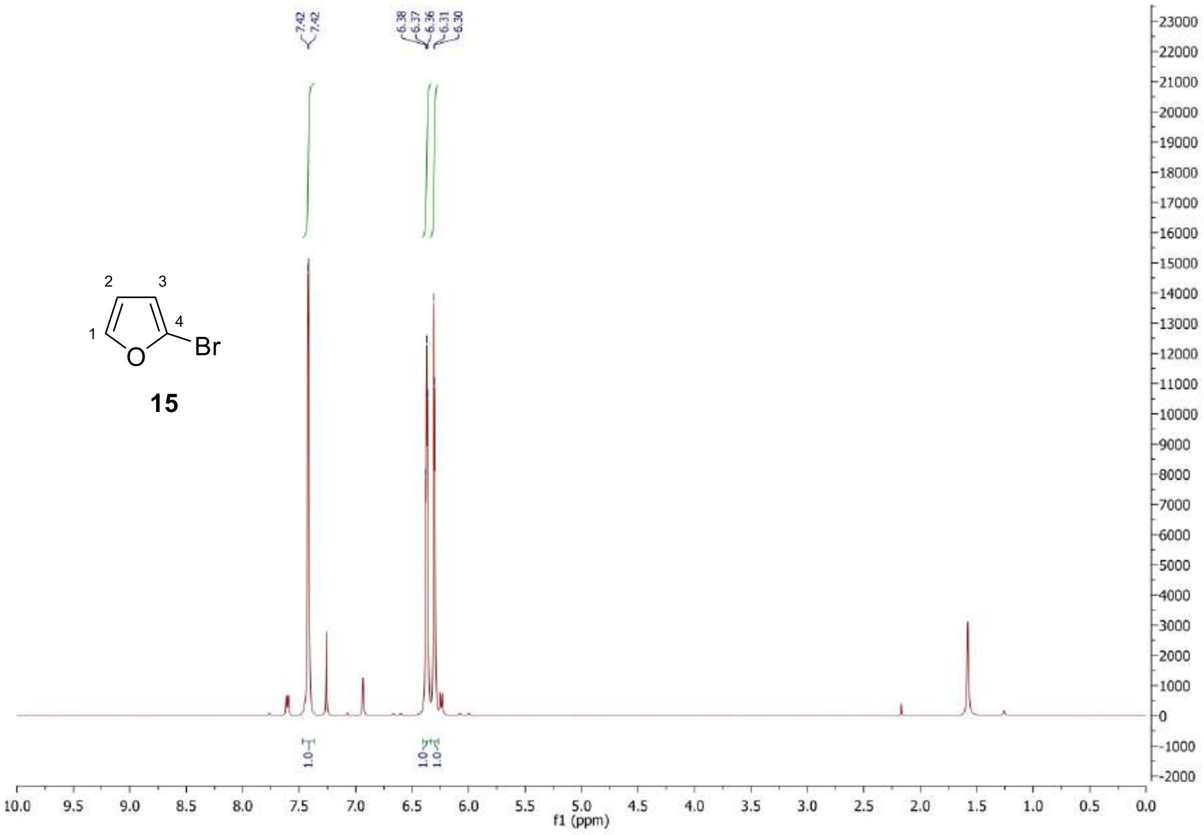

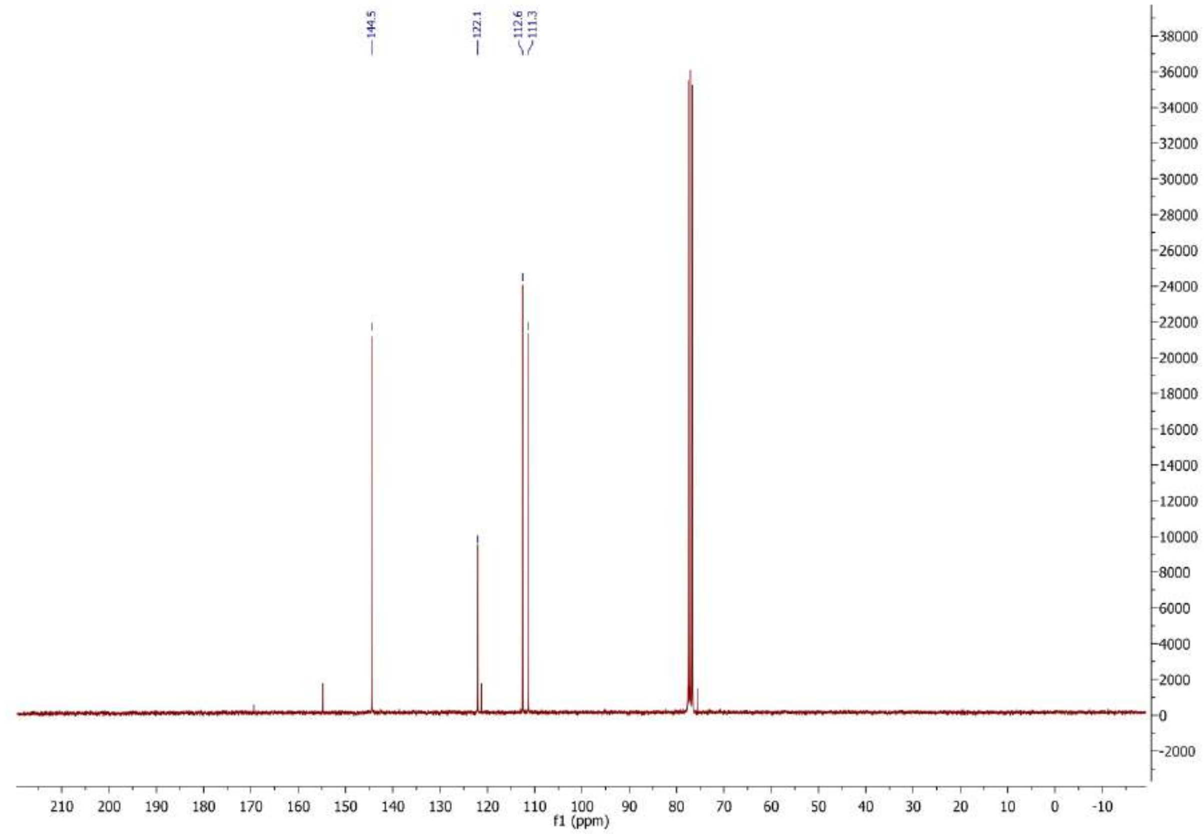

^{1}H- and ^{13}C-NMR-spectrum of ethyl (1*R*,2*S*,3*S*,4*R*)-4-bromo-3-nitro-7-oxabicyclo[2.2.1]hept-5-ene-2-carboxylate (16)

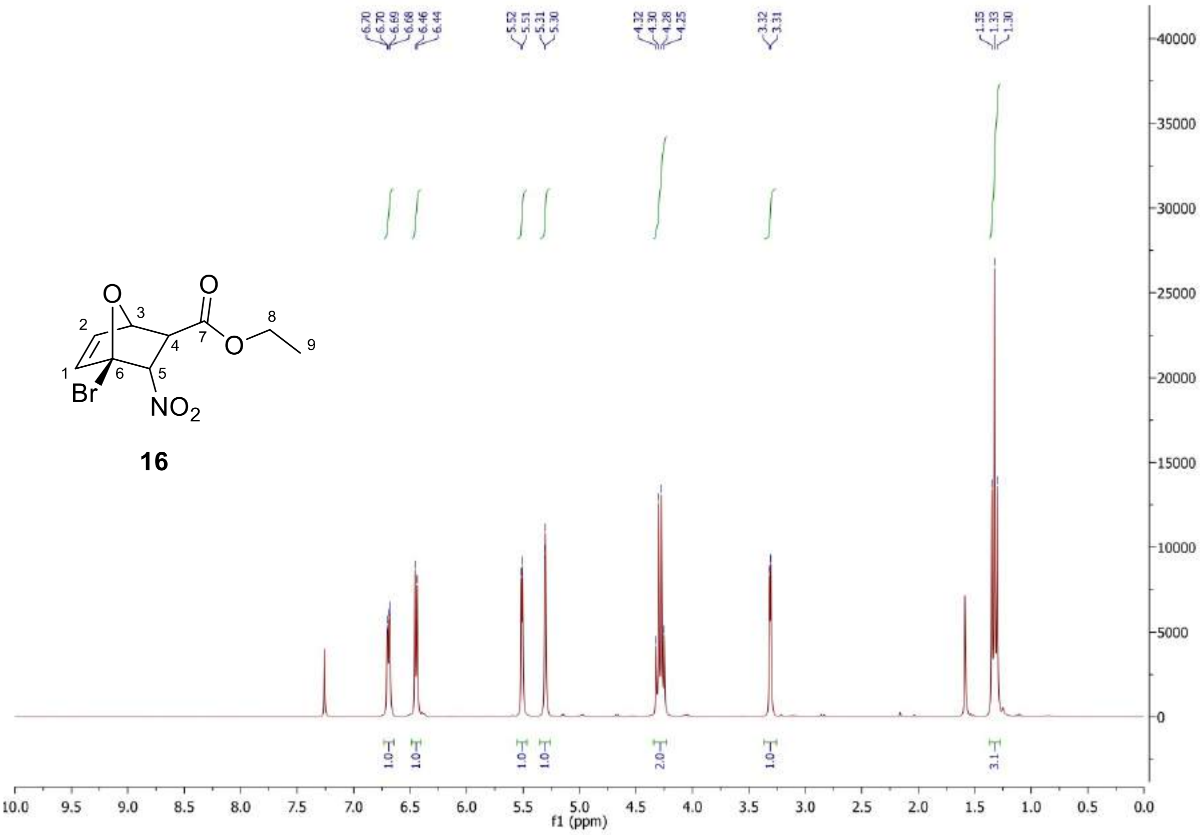

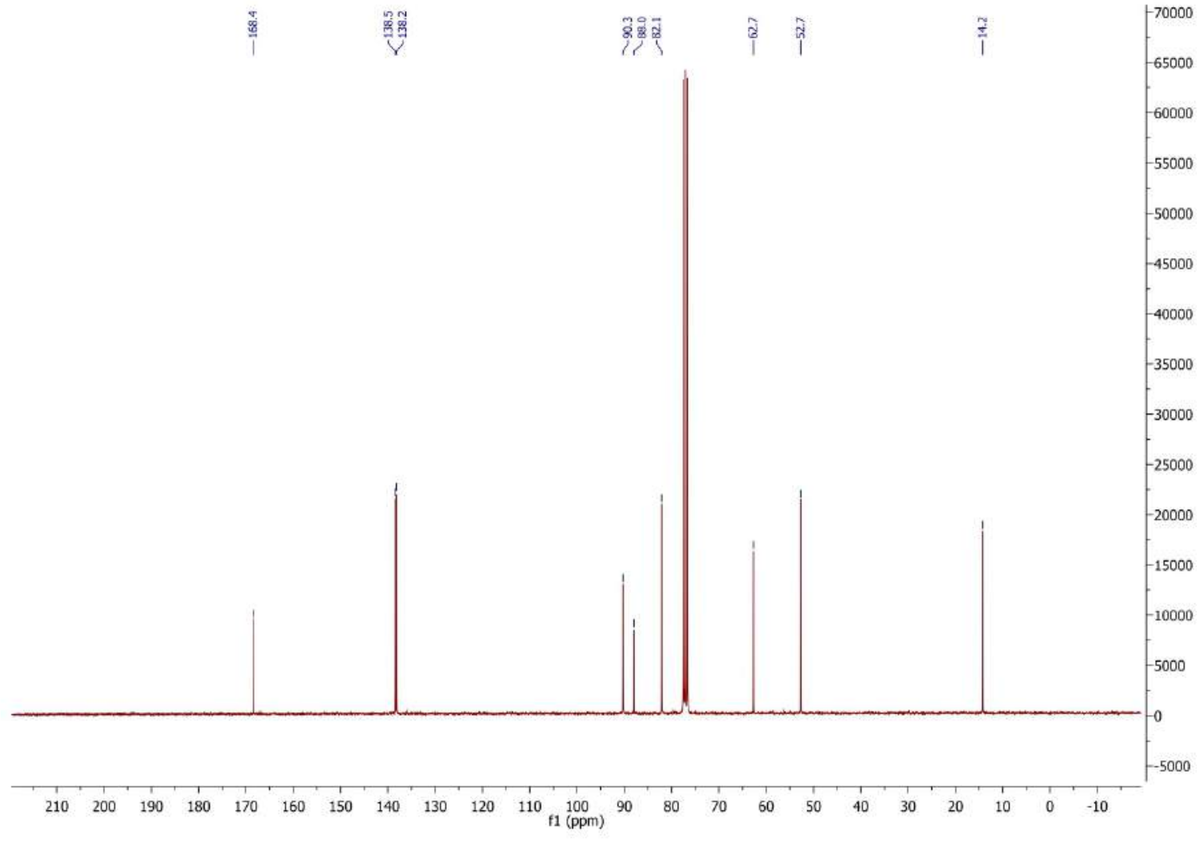

^{1}H- and ^{13}C-NMR-spectrum of ethyl (1*S*,2*R*,3*S*,4*R*)-1-bromo-3-nitro-7-oxabicyclo[2.2.1]hept-5-ene-2-carboxylate (21)

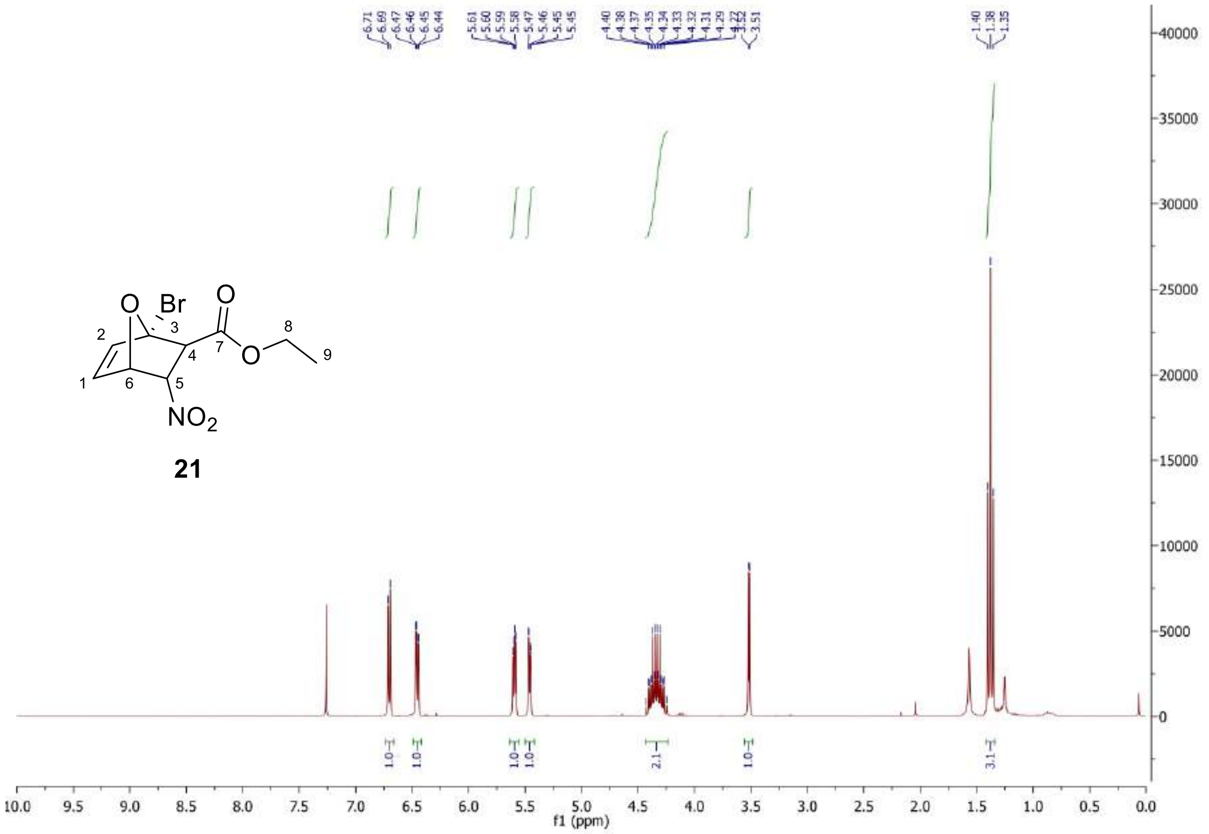

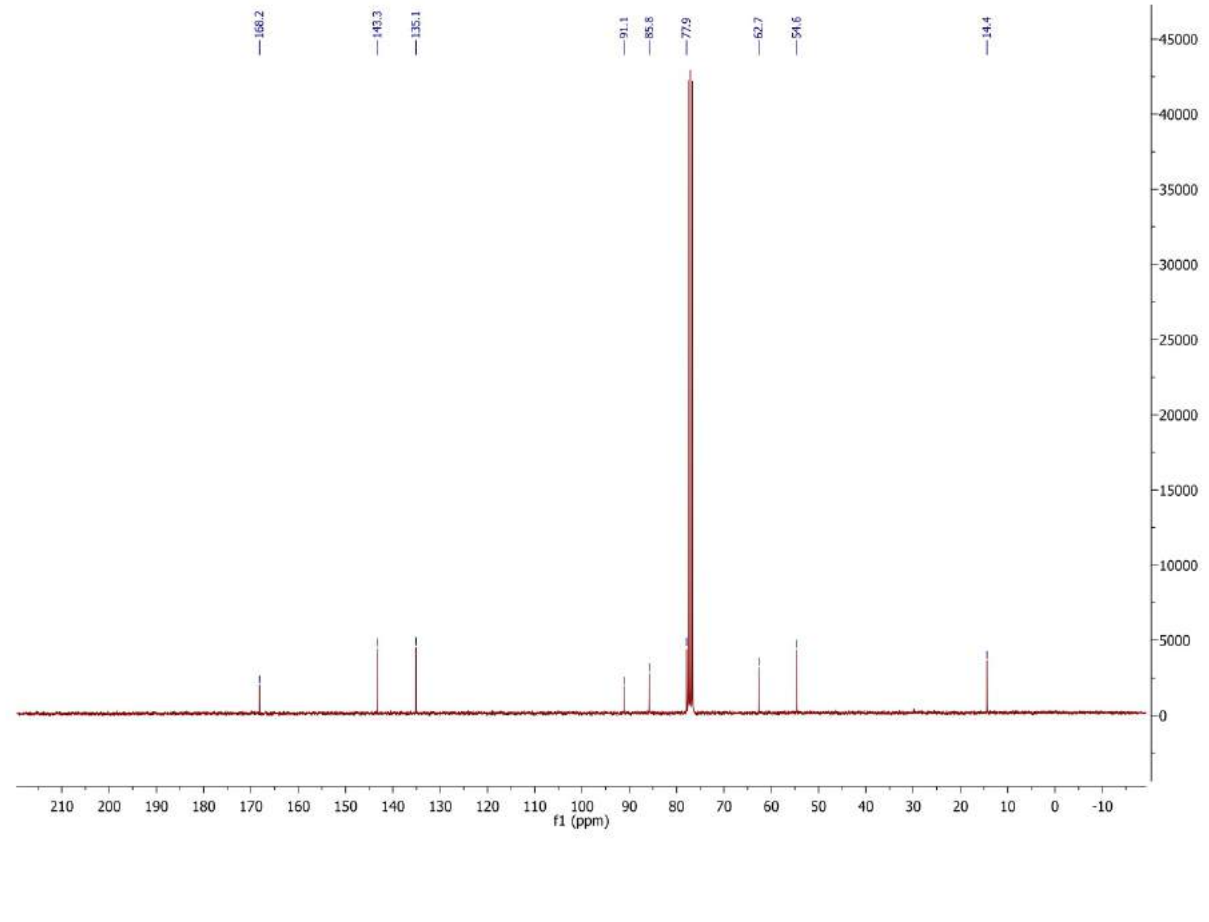

^{1}H- and APT-NMR-spectrum of ethyl (1*S*,2*S*,3*S*,4*S*)-4-bromo-3-nitro-7-oxabicyclo[2.2.1]hept-5-ene-2-carboxylate (22)

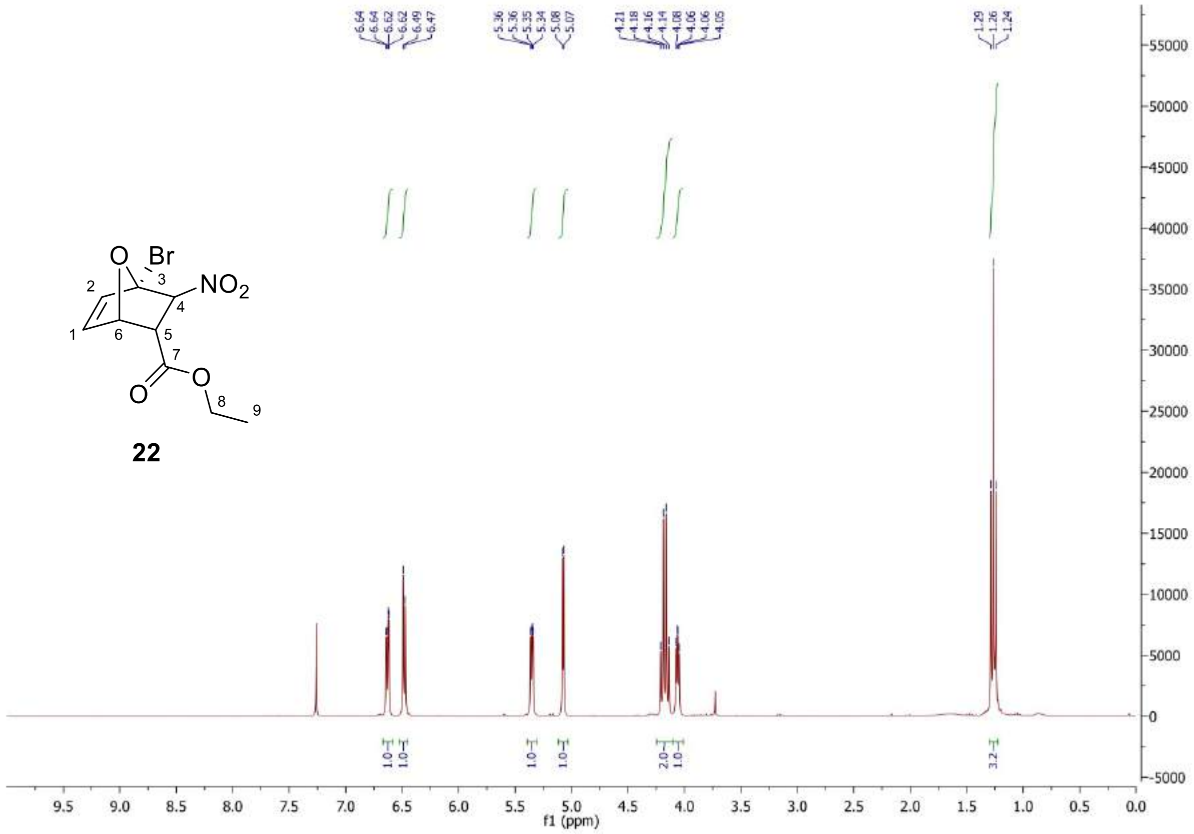

22

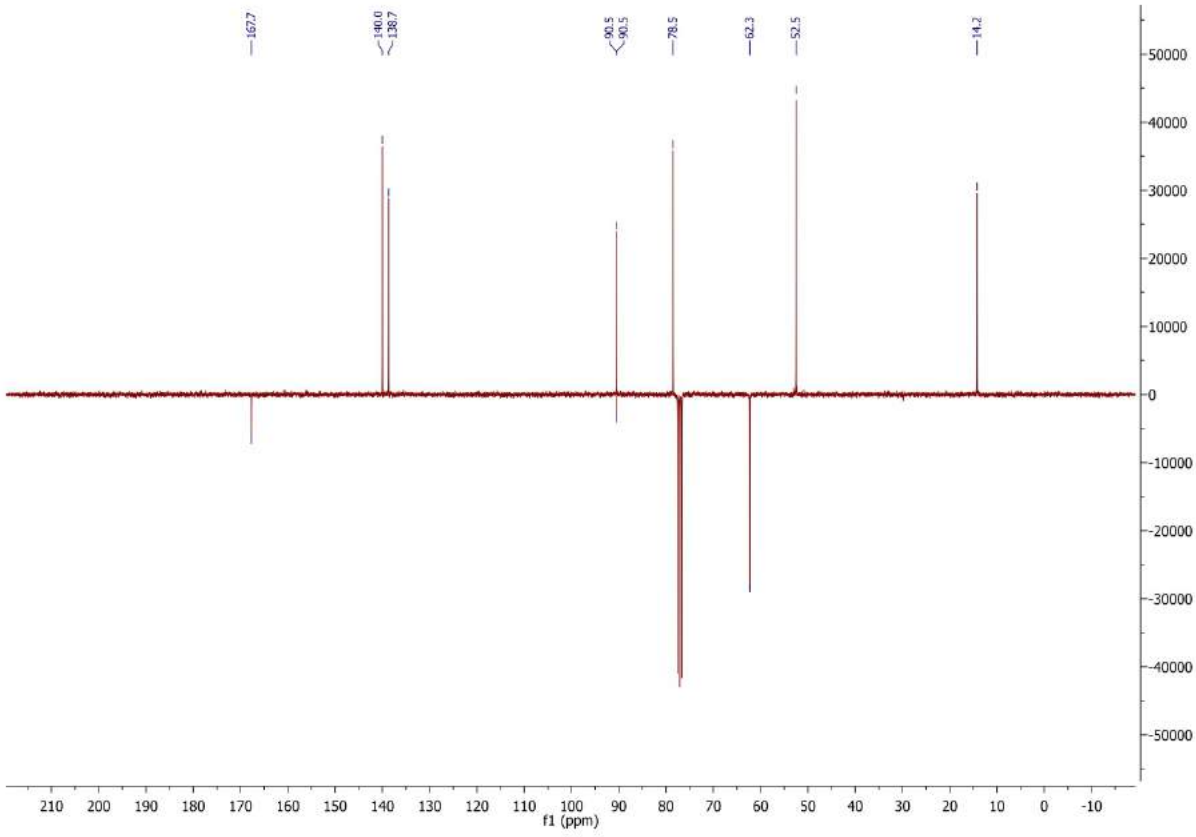

^{1}H- and APT-NMR-spectrum of ethyl (1*R*,2*R*,3*S*,4*R*)-1-bromo-3-nitro-7-oxabicyclo[2.2.1]hept-5-ene-2-carboxylate (23)

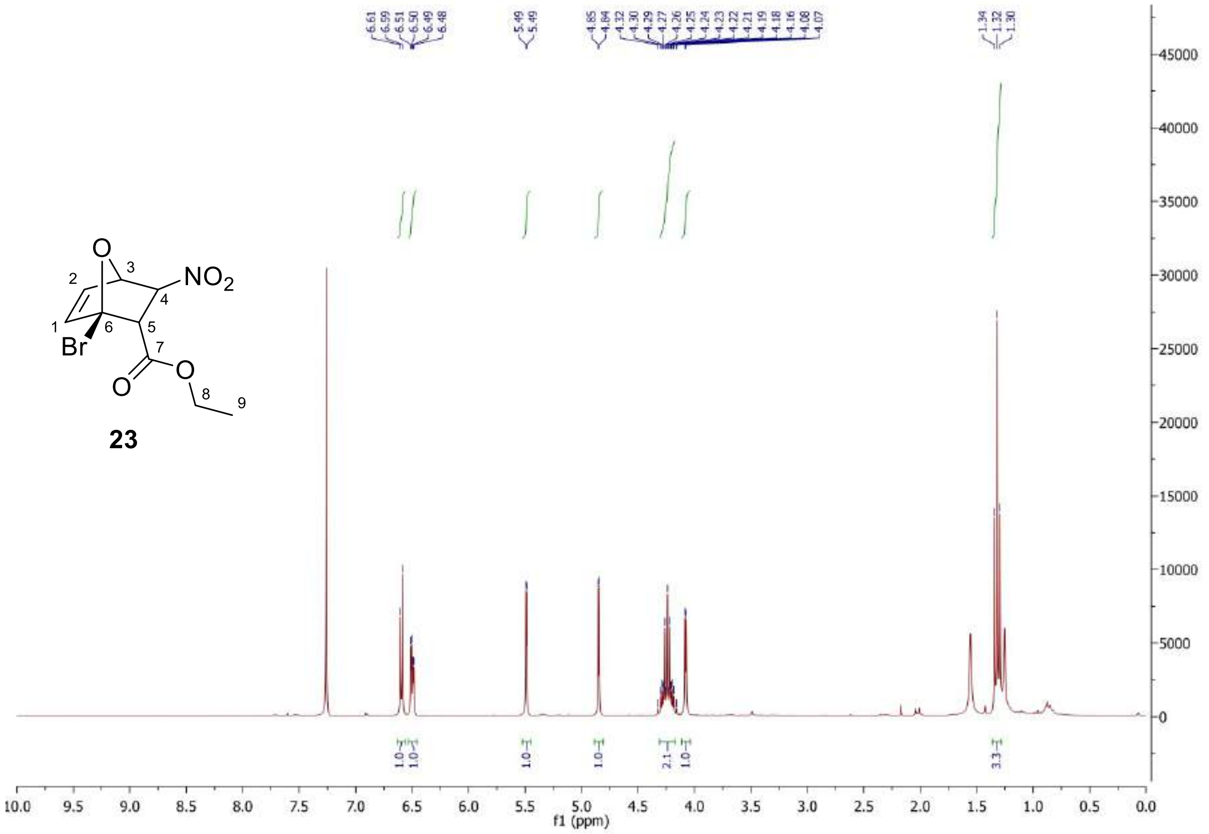

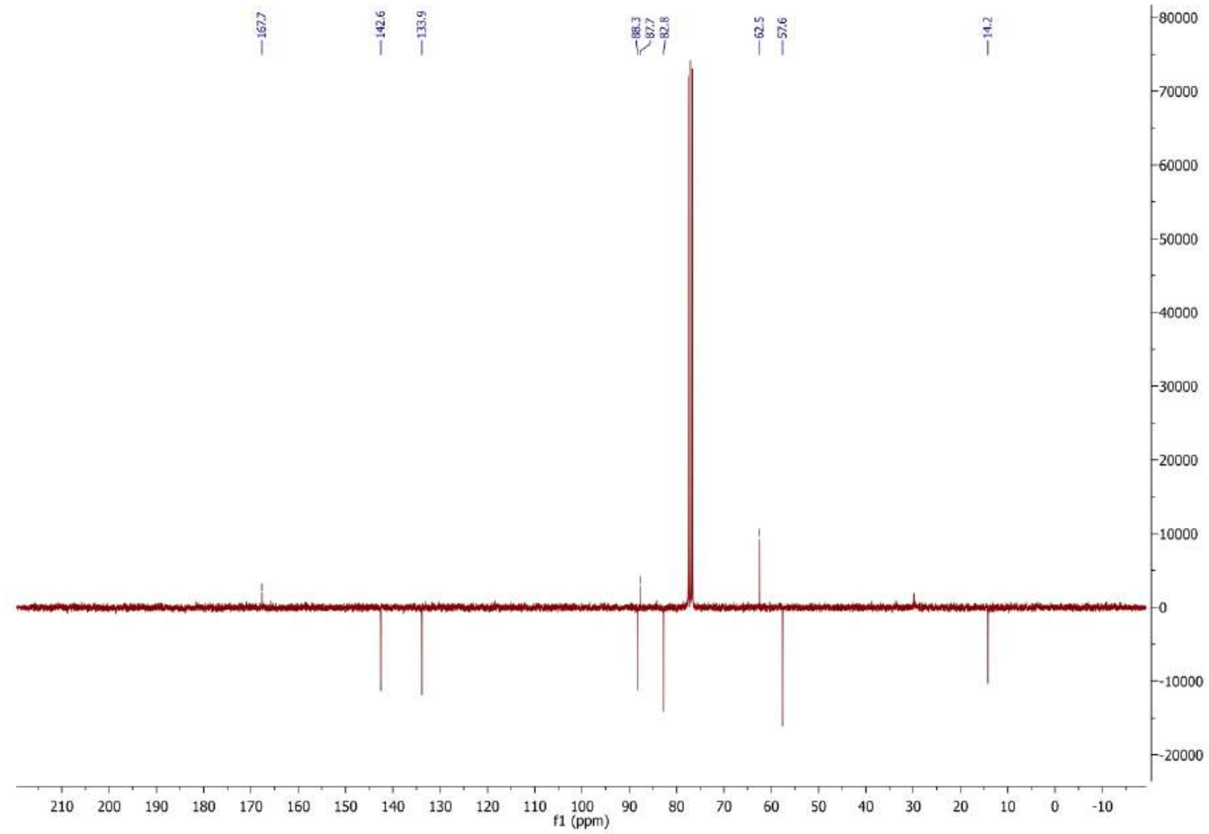

^{1}H- and ^{13}C-NMR-spectrum of ethyl (1*R*,2*S*,3*S*,4*S*)-3-((*tert*-butoxycarbonyl)amino)-7-oxabicyclo[2.2.1]-hept-5-ene-2-carboxylate-4-*d* (17)

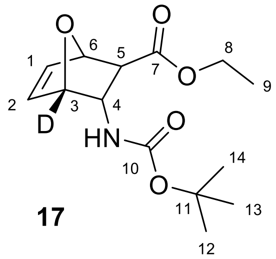

17

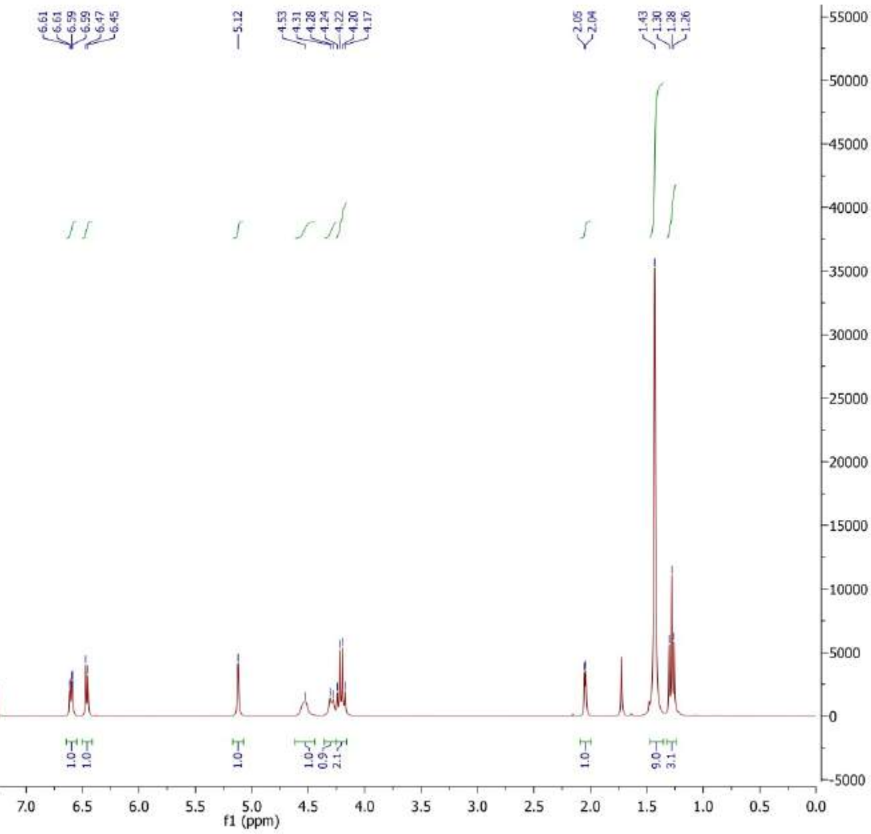

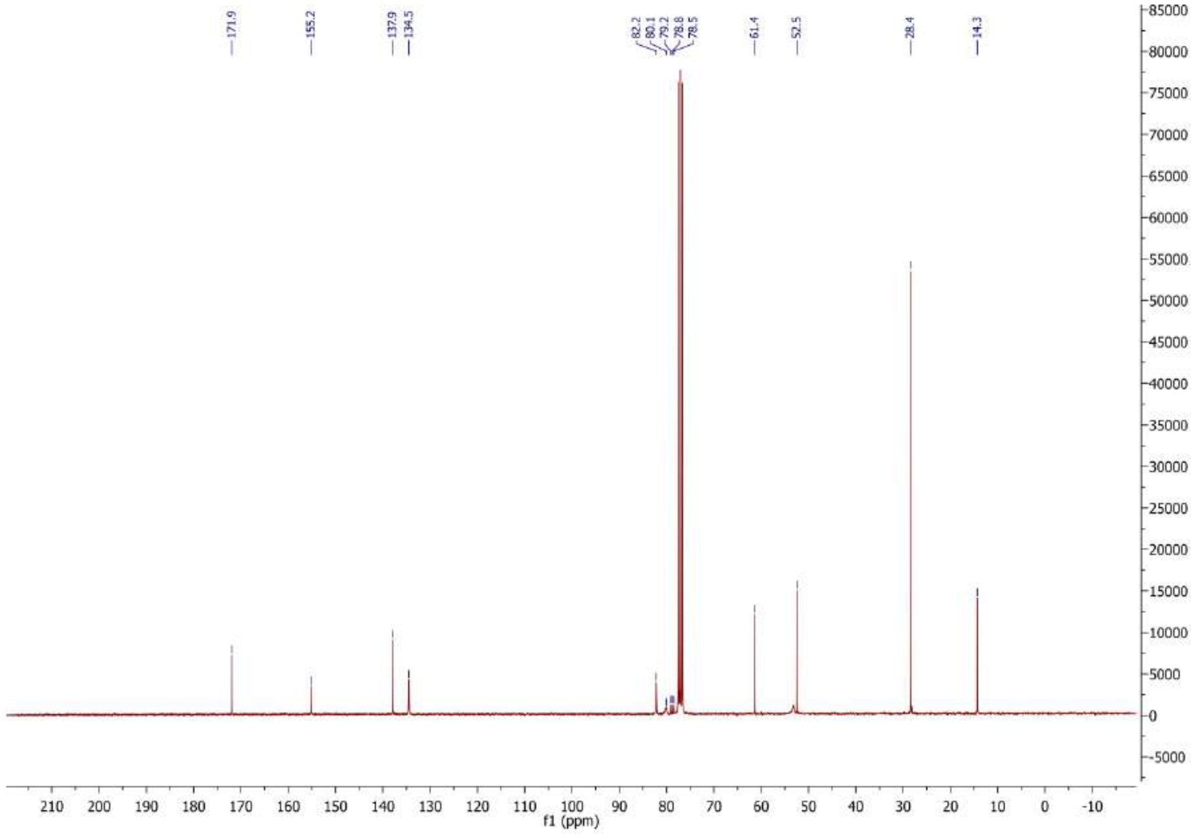

^{1}H- and ^{13}C-NMR-spectrum of (1*R*,2*S*,3*S*,4*S*)-3-((*tert*-butoxycarbonyl)amino)-7-oxabicyclo[2.2.1]-hept-5-ene-2-carboxylic-4-*d* acid (18)

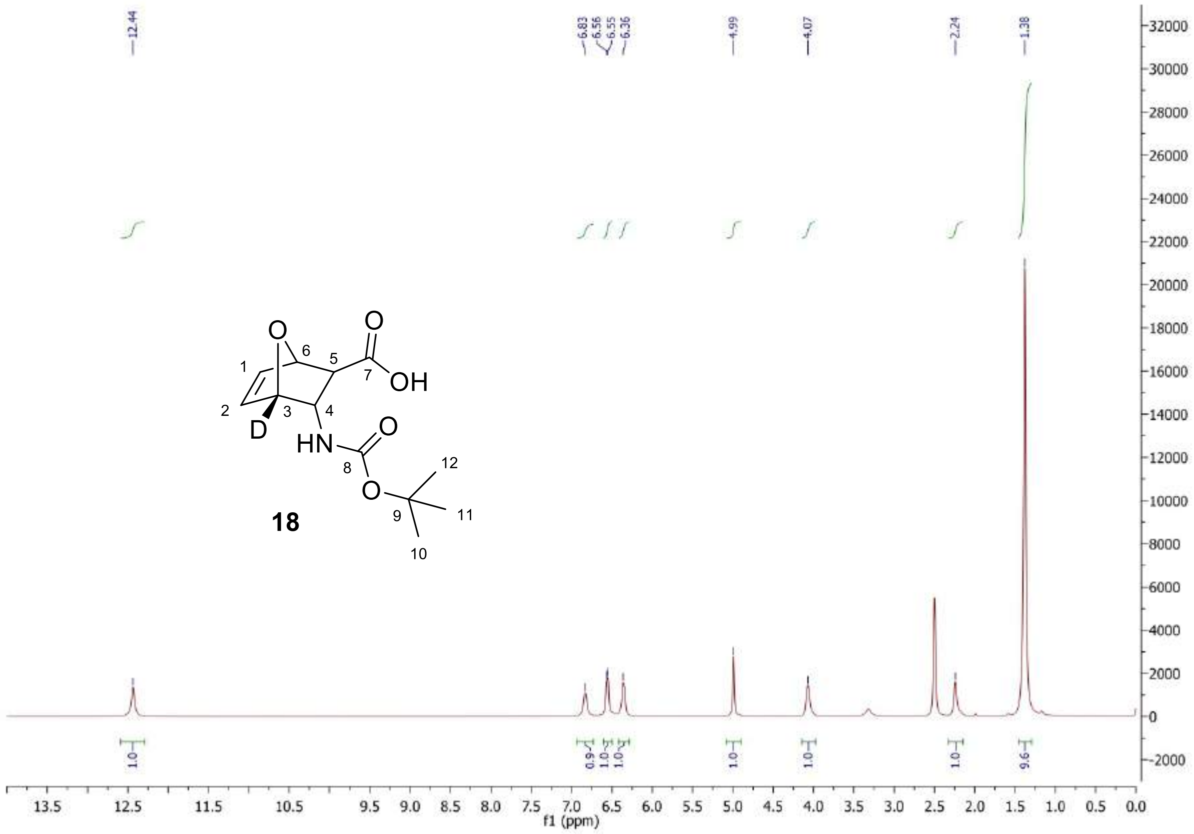

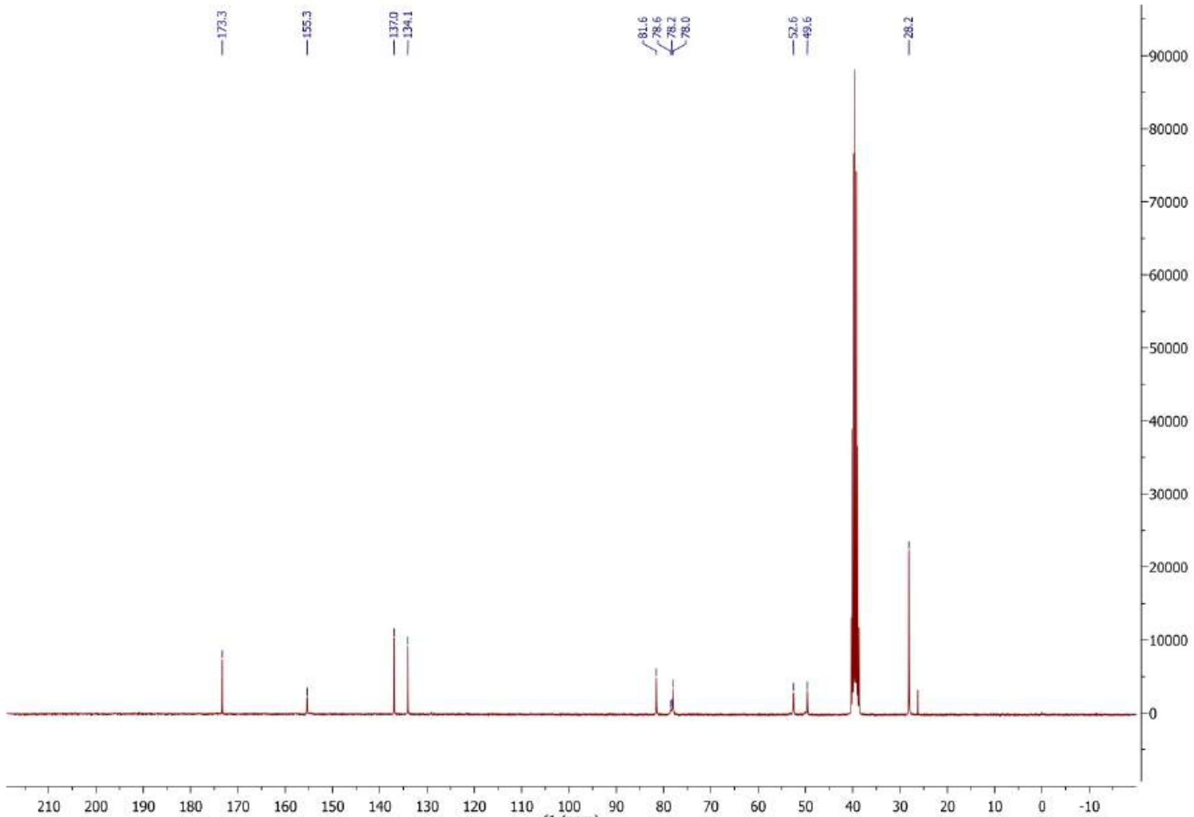

^{1}H- and ^{13}C-NMR-spectrum of ethyl (5*S*,6*S*)-6-((*tert*-butoxycarbonyl)amino)-5-hydroxycyclohexa-1,3-diene-1-carboxylate-5-*d* (19)

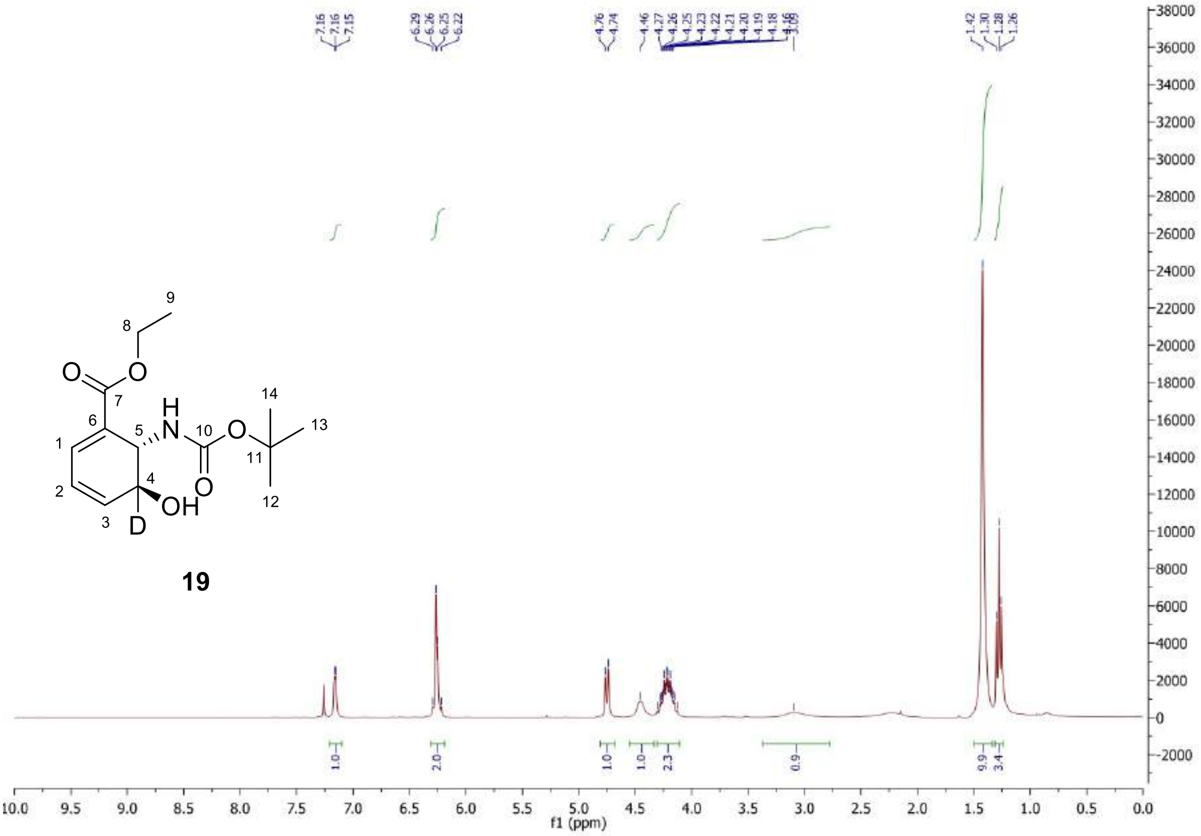

19

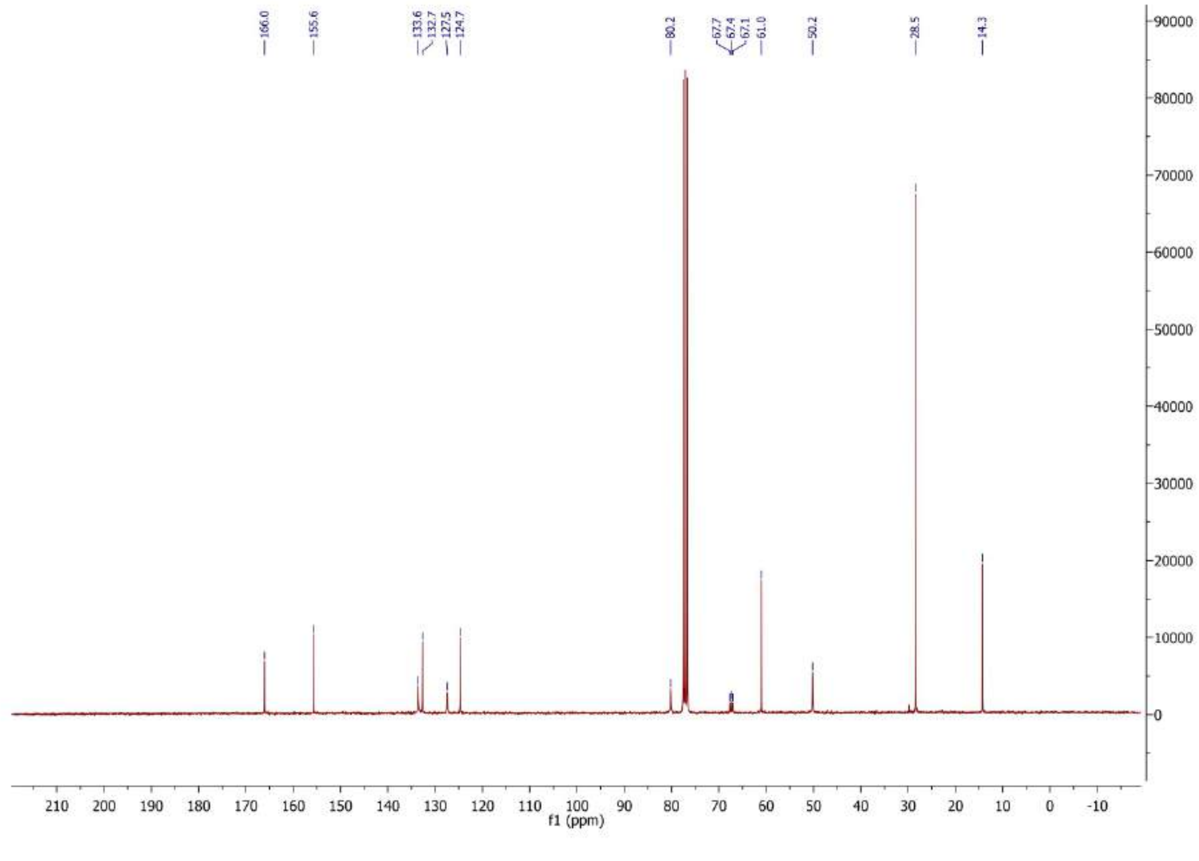

^{1}H- and ^{13}C-NMR-spectrum of (5*S*,6*S*)-6-((*tert*-butoxycarbonyl)amino)-5-hydroxycyclohexa-1,3-diene-1-carboxylic-5-*d* acid (24)

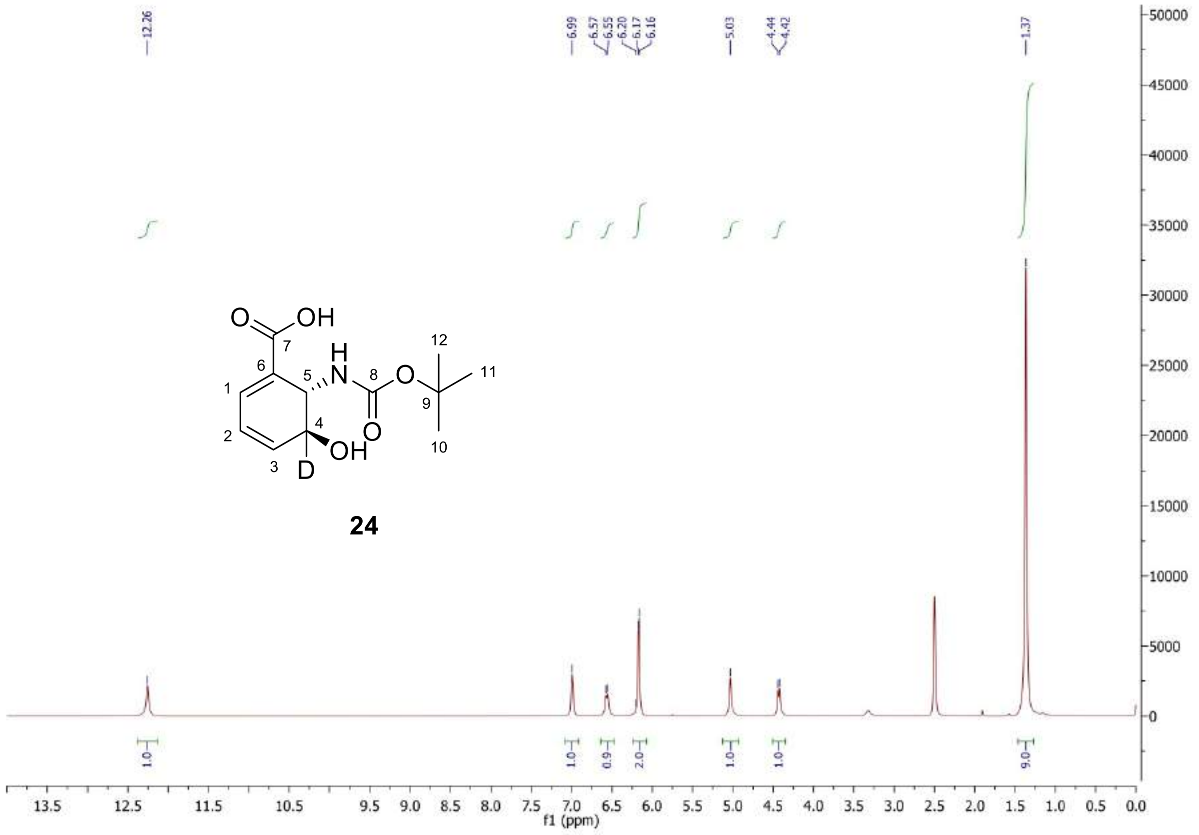

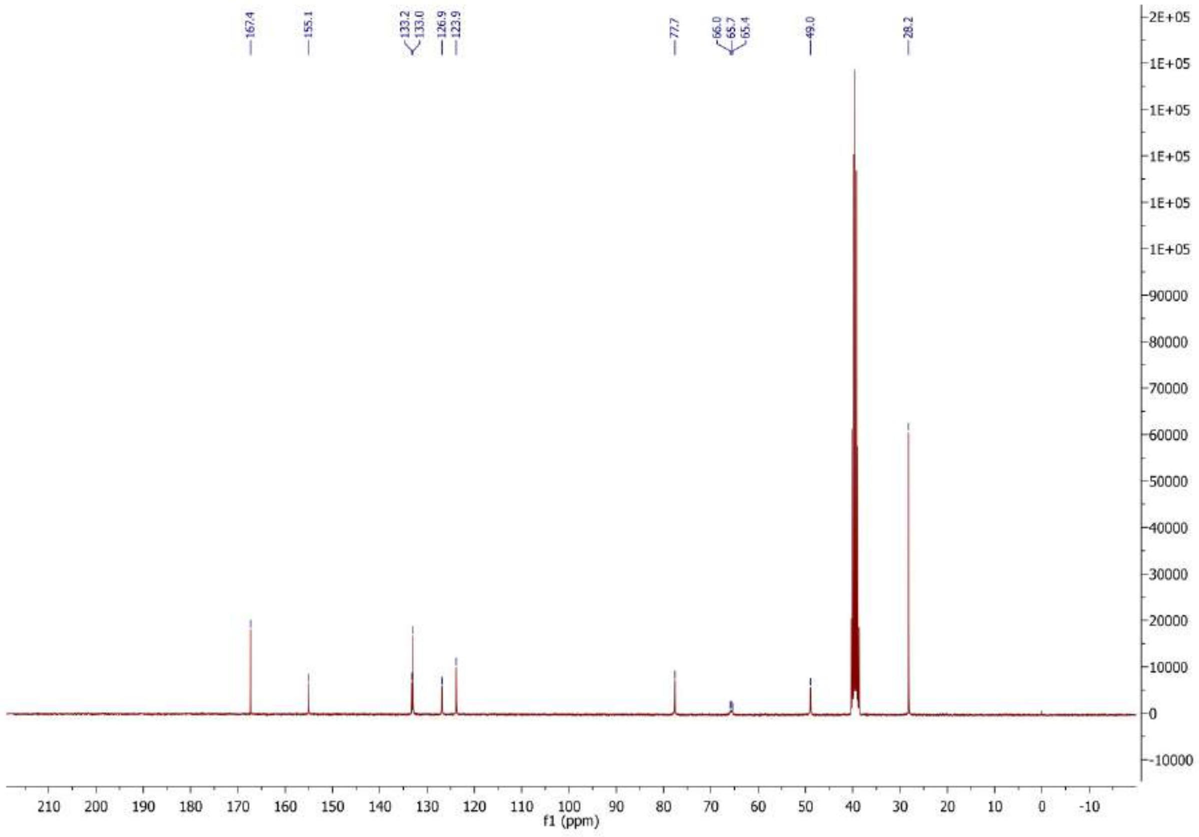

^{1}H-, ^{13}C- and ^{19}F-NMR-spectrum of (1*S*,6*S*)-2-carboxy-6-hydroxycyclohexa-2,4-diene-6-*d*-1-ammonium 2,2,2-trifluoroacetate (20) (d-DHHA)

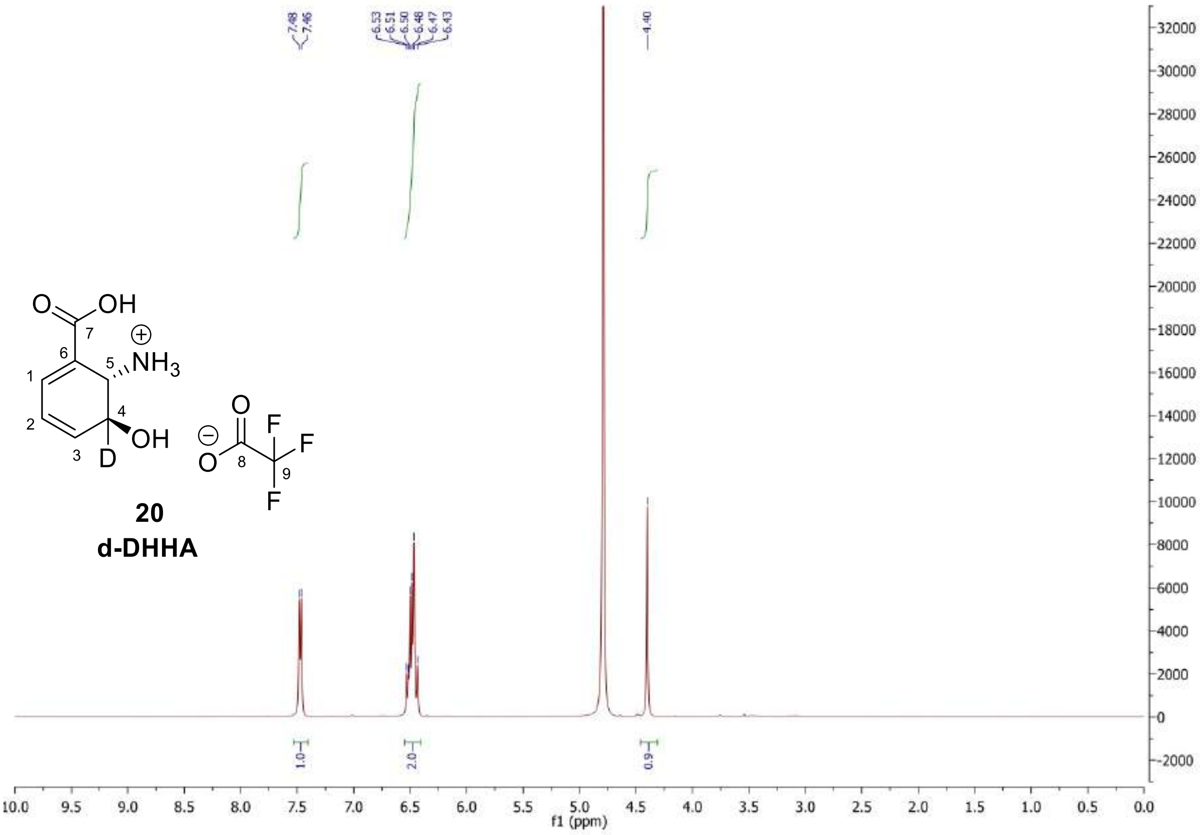

20
d-DHHA

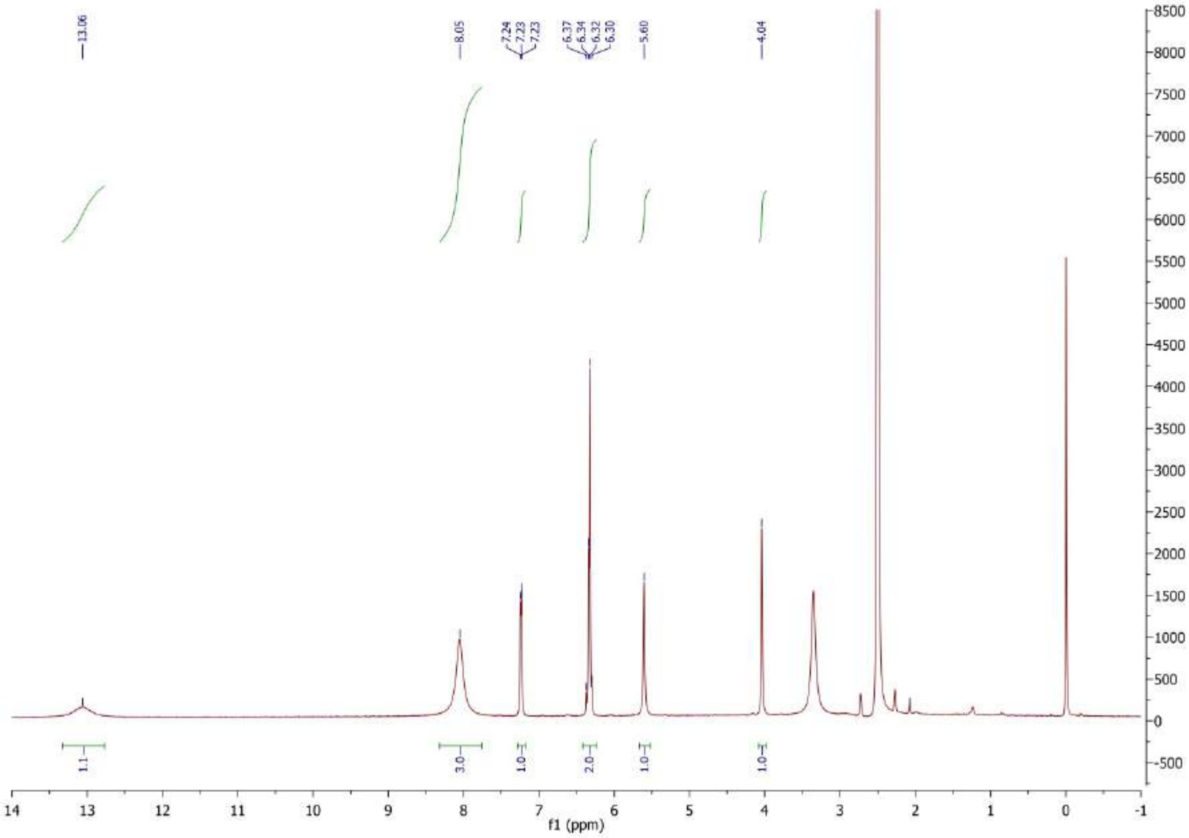

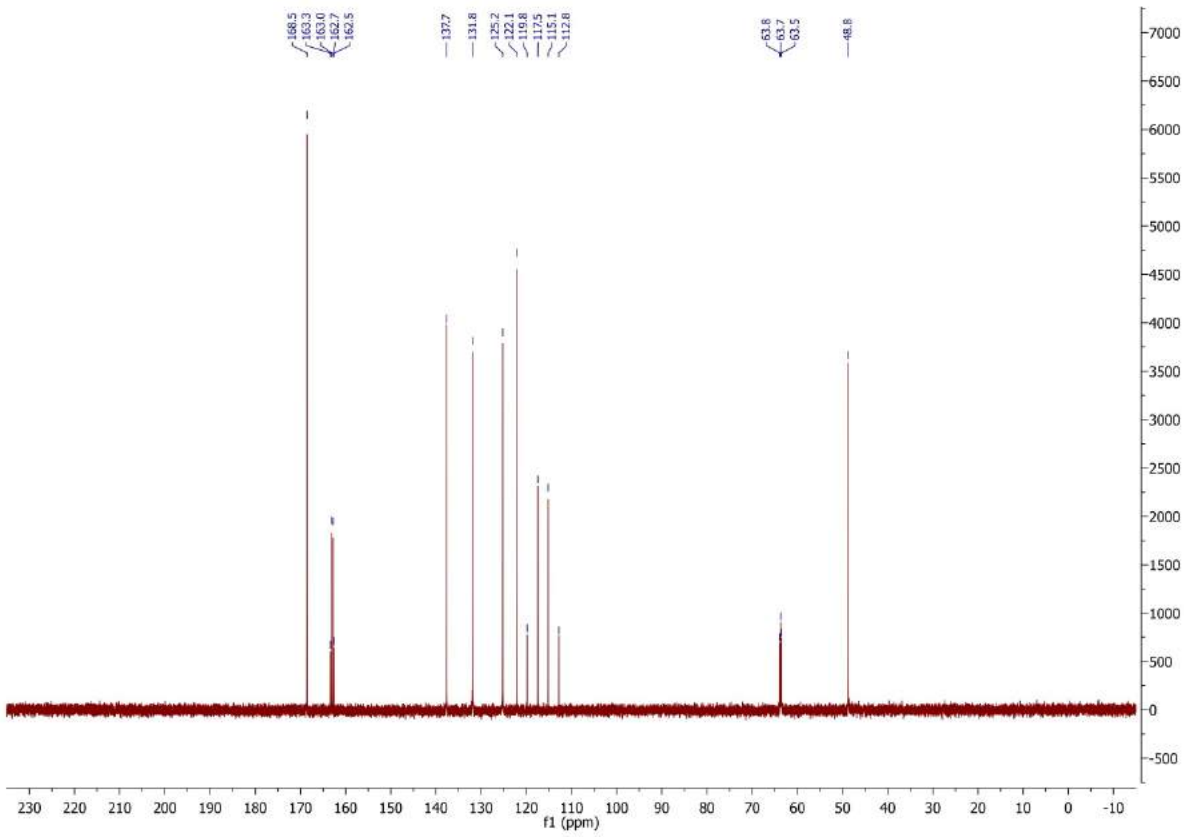

168.5
163.3
163.0
162.7
162.5
137.7
131.8
125.2
122.1
119.8
117.5
115.1
112.8
63.8
63.7
63.5
48.8
f1 (ppm)

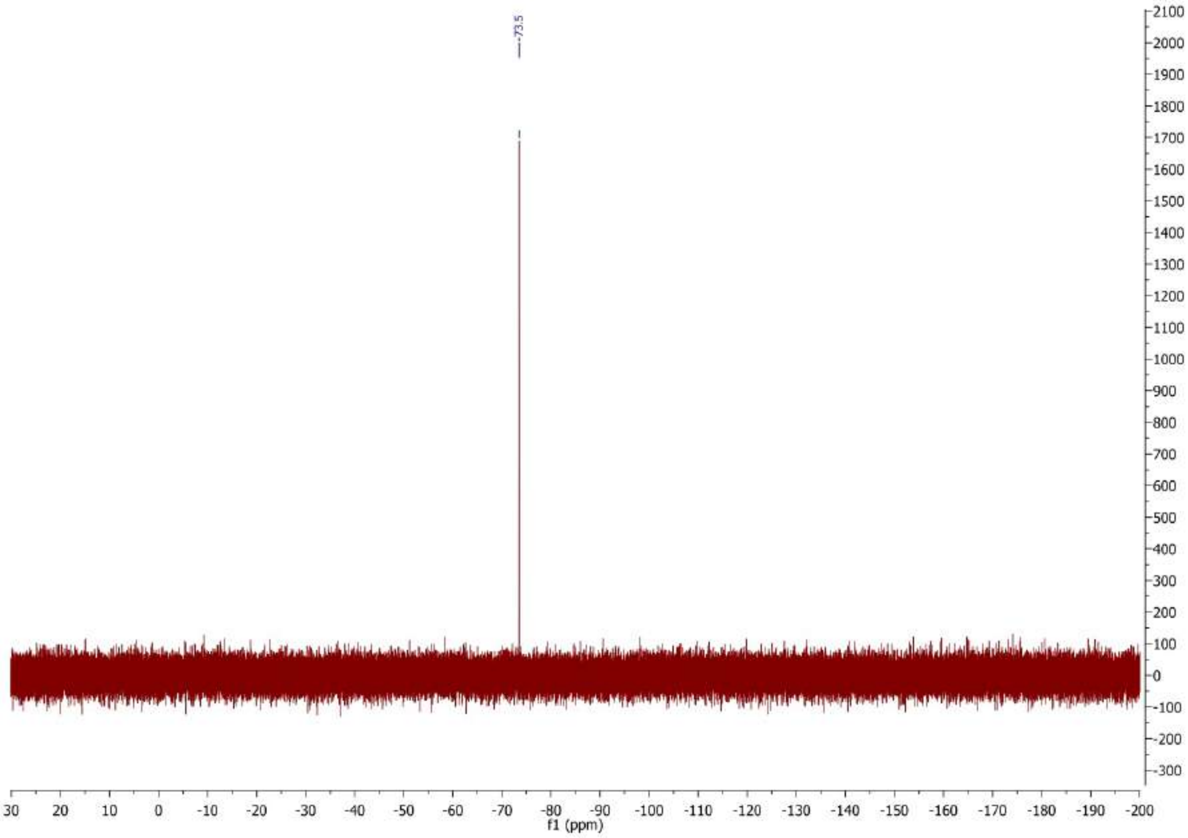

-73.5
f1 (ppm)

^{1}H- and ^{13}C-NMR-spectrum of ethyl (5*S*,6*S*)-6-((*tert*-butoxycarbonyl)amino)-5-methoxycyclohexa-1,3-diene-1-carboxylate (29)

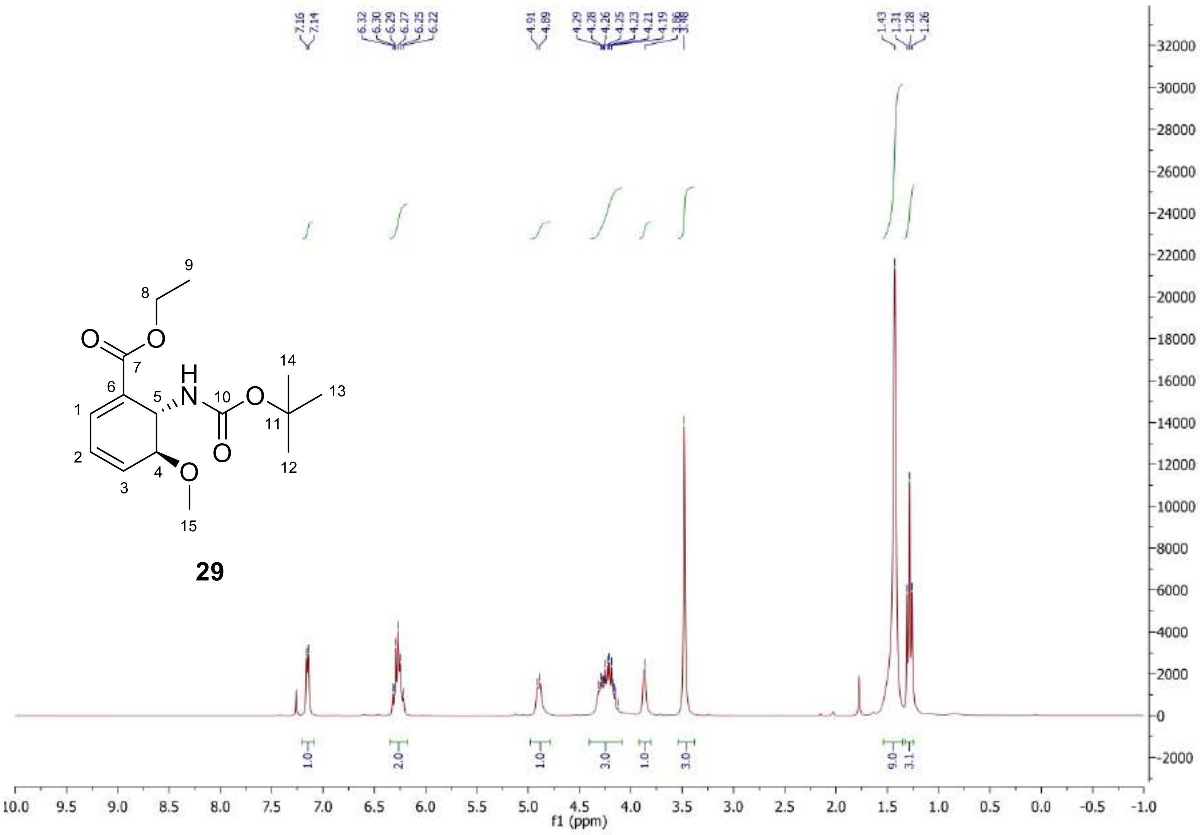

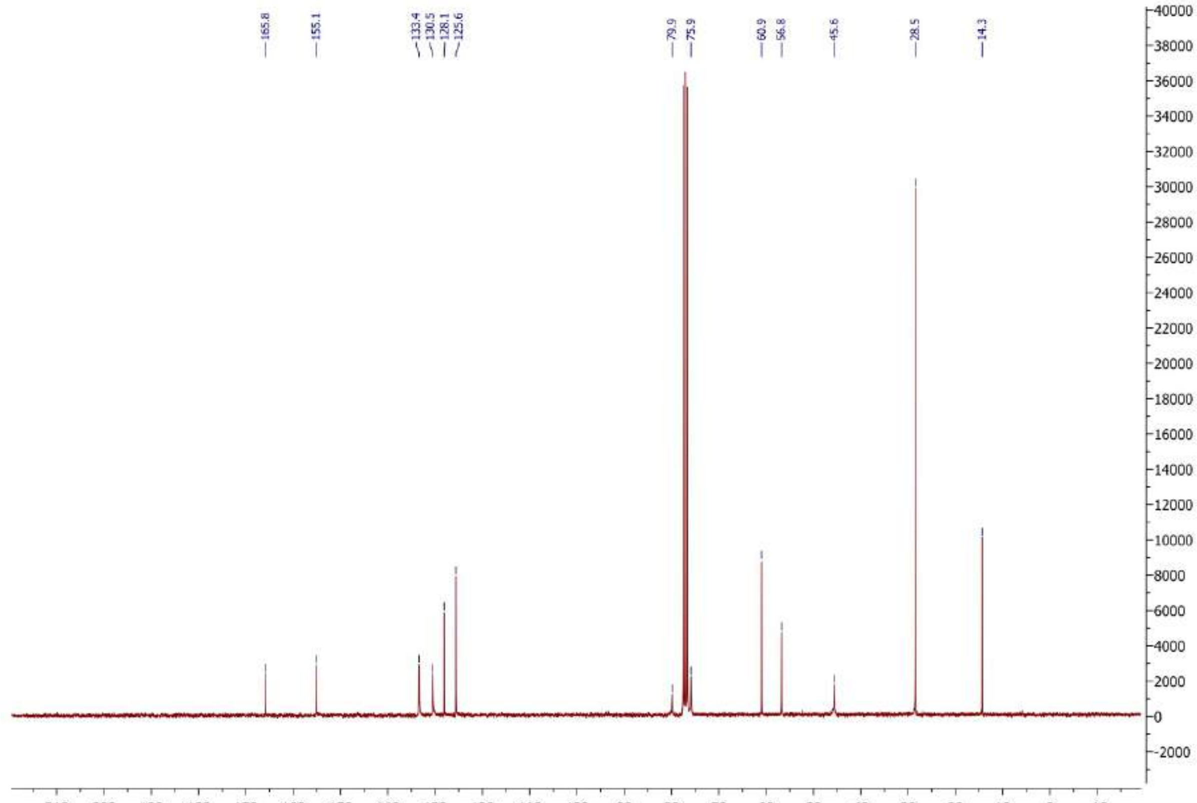

^{1}H- and ^{13}C-NMR-spectrum of (5*S*,6*S*)-6-((*tert*-butoxycarbonyl)amino)-5-methoxycyclohexa-1,3-diene-1-carboxylic acid (30)

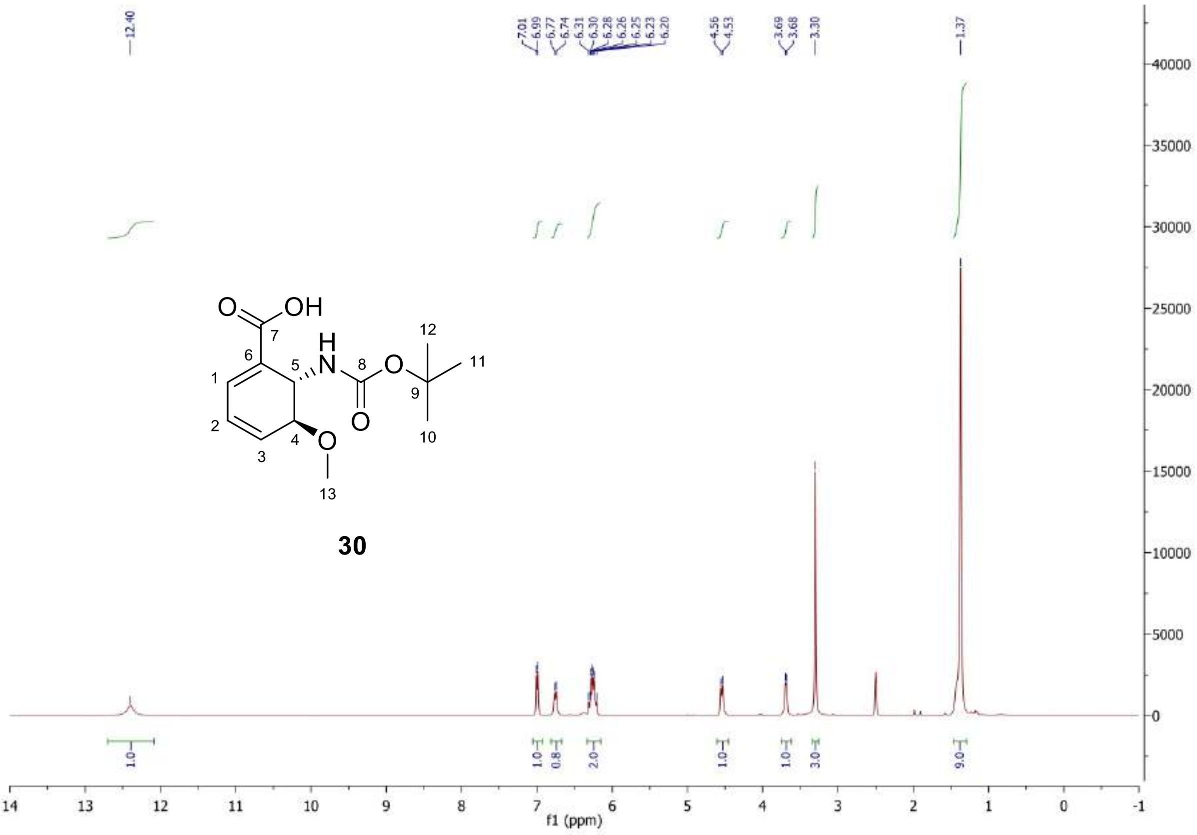

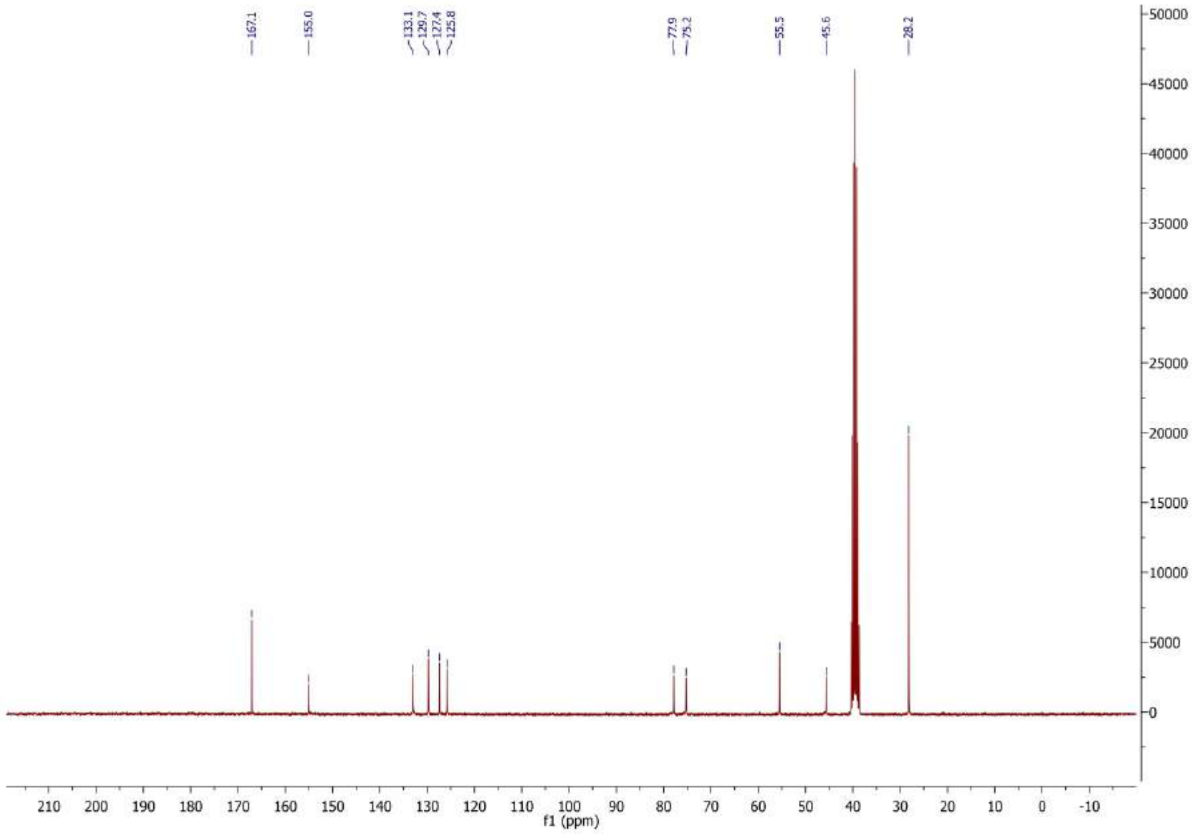

^{1}H-, ^{13}C- and ^{19}F-NMR-spectrum of (1*S*,6*S*)-2-carboxy-6-methoxycyclohexa-2,4-diene-1-ammonium 2,2,2-trifluoroacetate (26) (*O*-Me-DHHA)

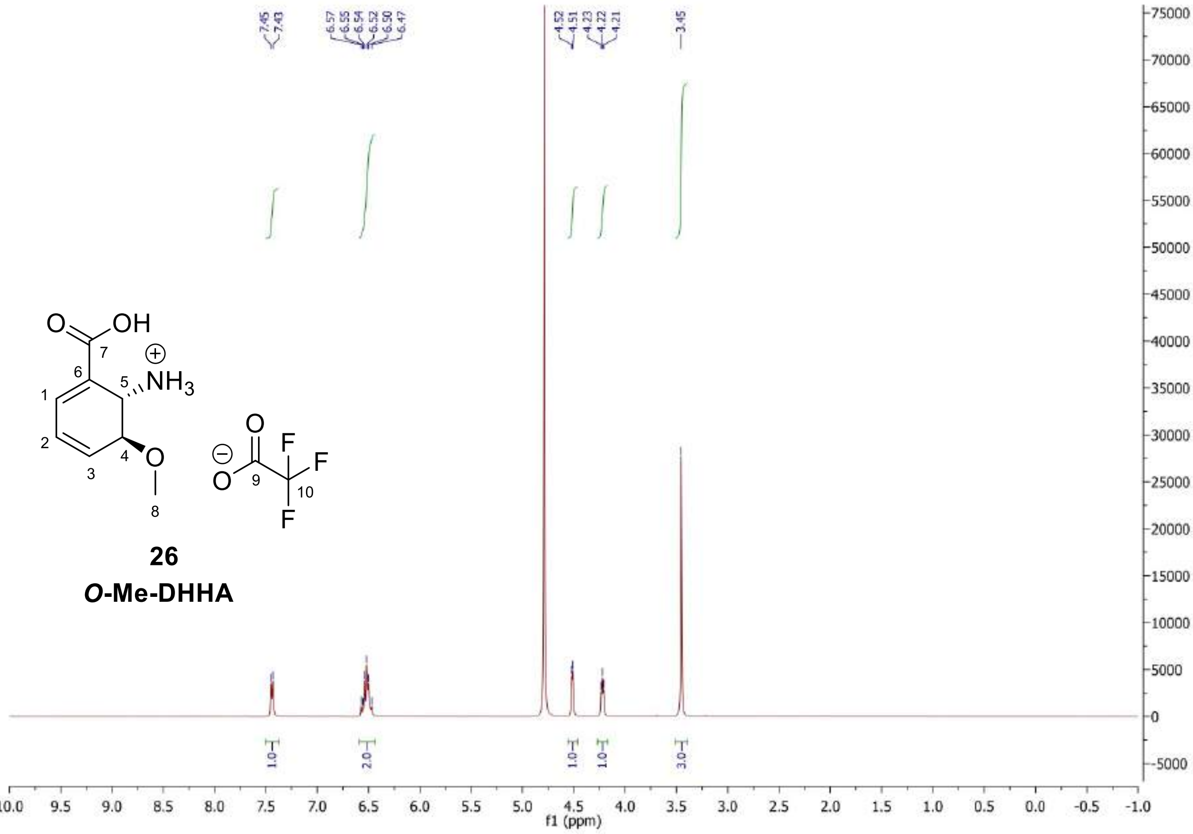

26
***O*-Me-DHHA**

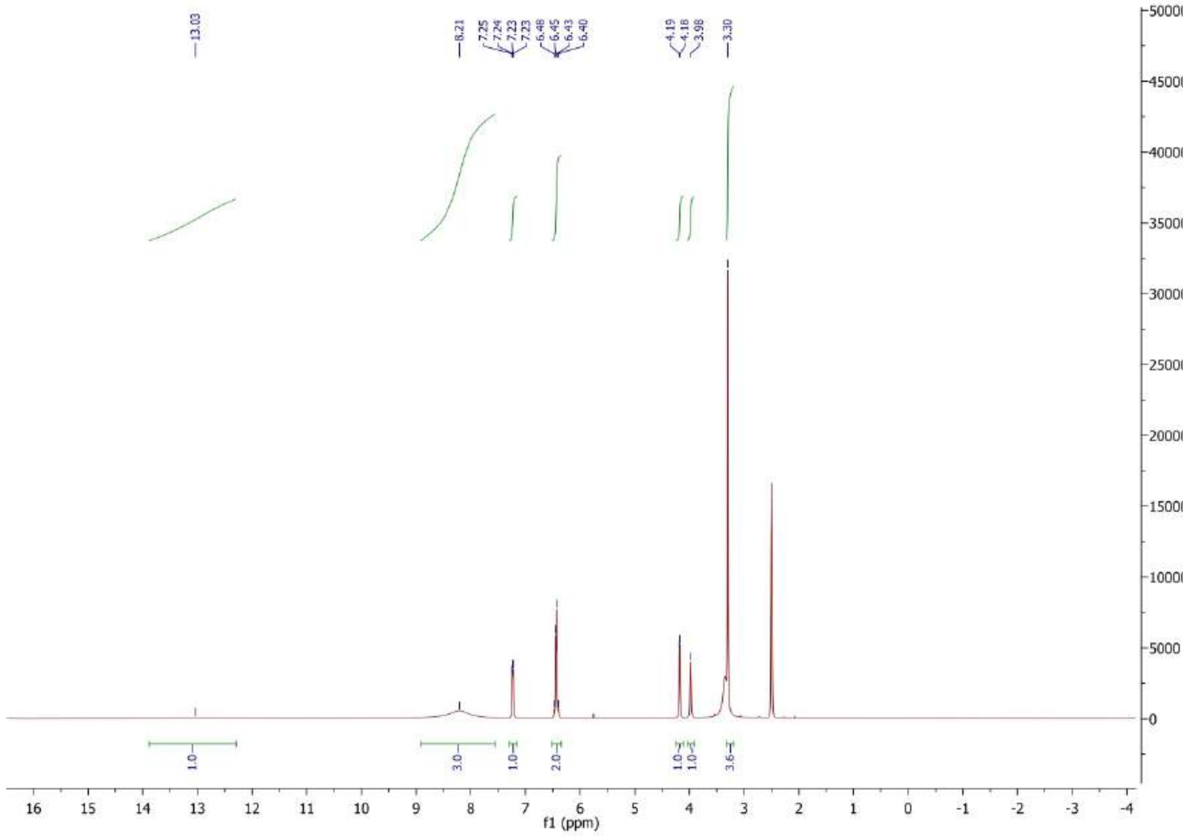

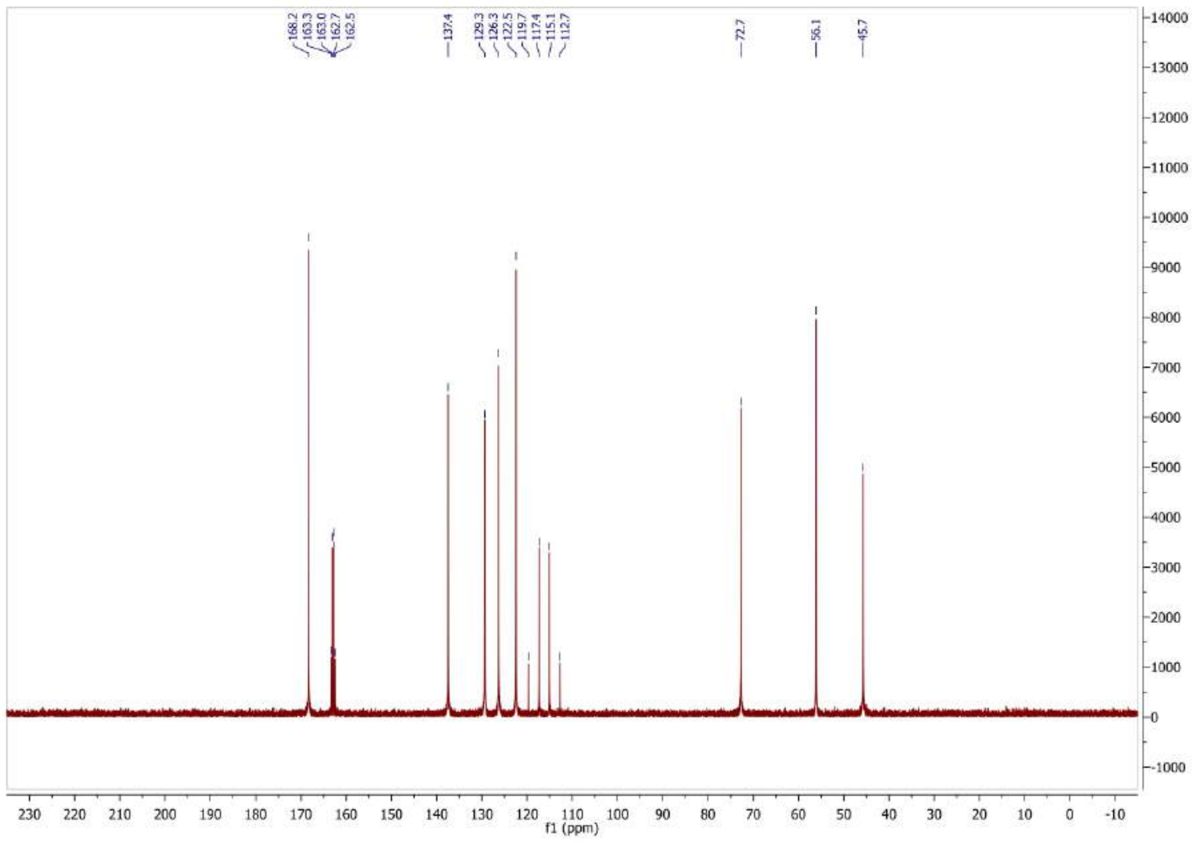
168.2
163.3
163.0
162.7
162.5
137.4
129.3
126.3
122.5
119.7
117.4
115.1
112.7
72.7
56.1
45.7
f1 (ppm)

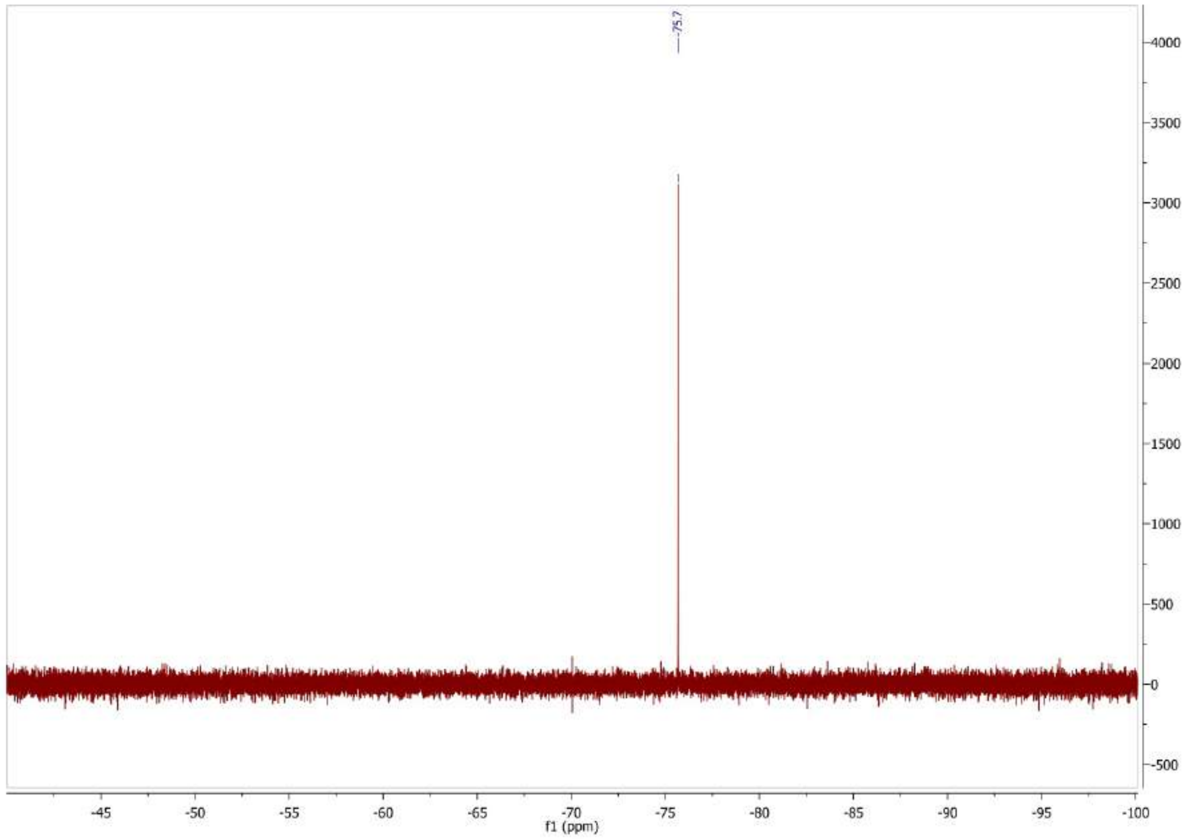
-75.7
f1 (ppm)

^{1}H- and ^{13}C-NMR-spectrum of ethyl (5*S*,6*S*)-6-((*tert*-butoxycarbonyl)amino)-5-ethoxycyclohexa-1,3-diene-1-carboxylate (*rac*-32)

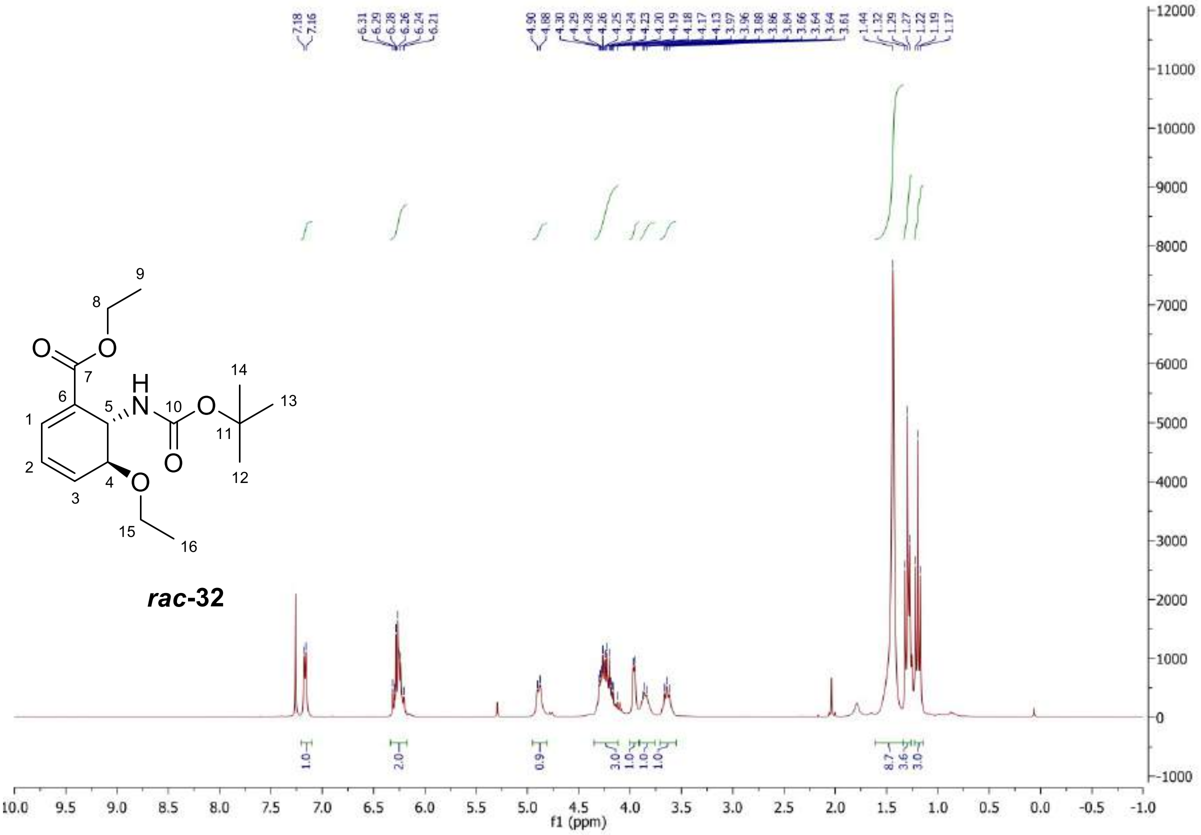

***rac*-32**

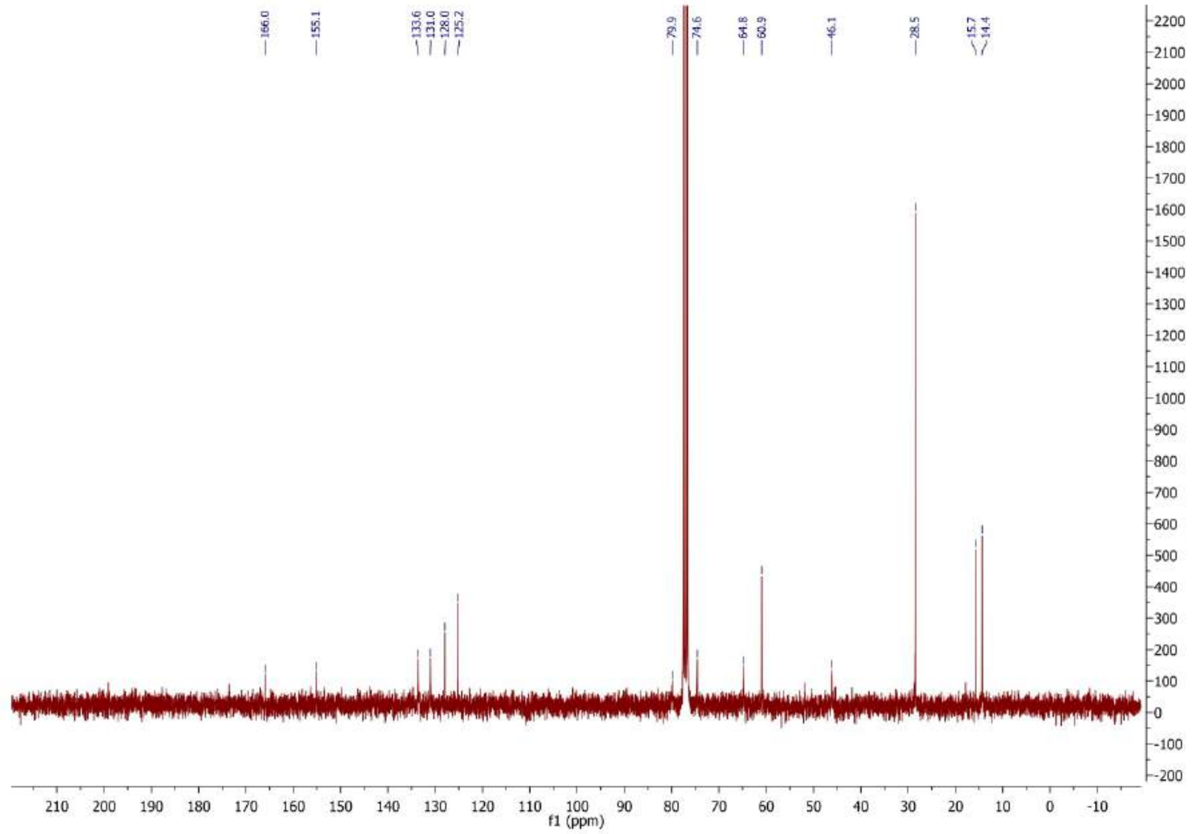

^{1}H- and ^{13}C-NMR-spectrum of (5*S*,6*S*)-6-((*tert*-butoxycarbonyl)amino)-5-ethoxycyclohexa-1,3-diene-1-carboxylic acid (*rac*-33)

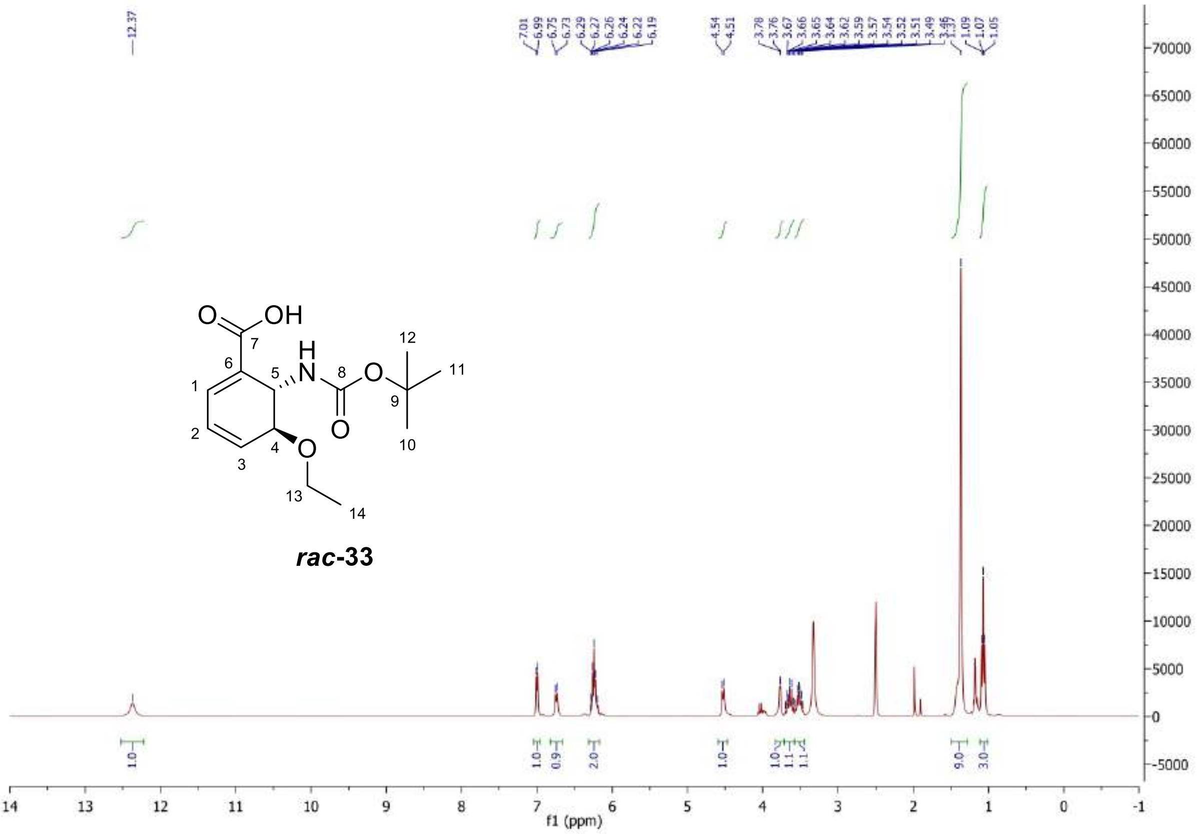

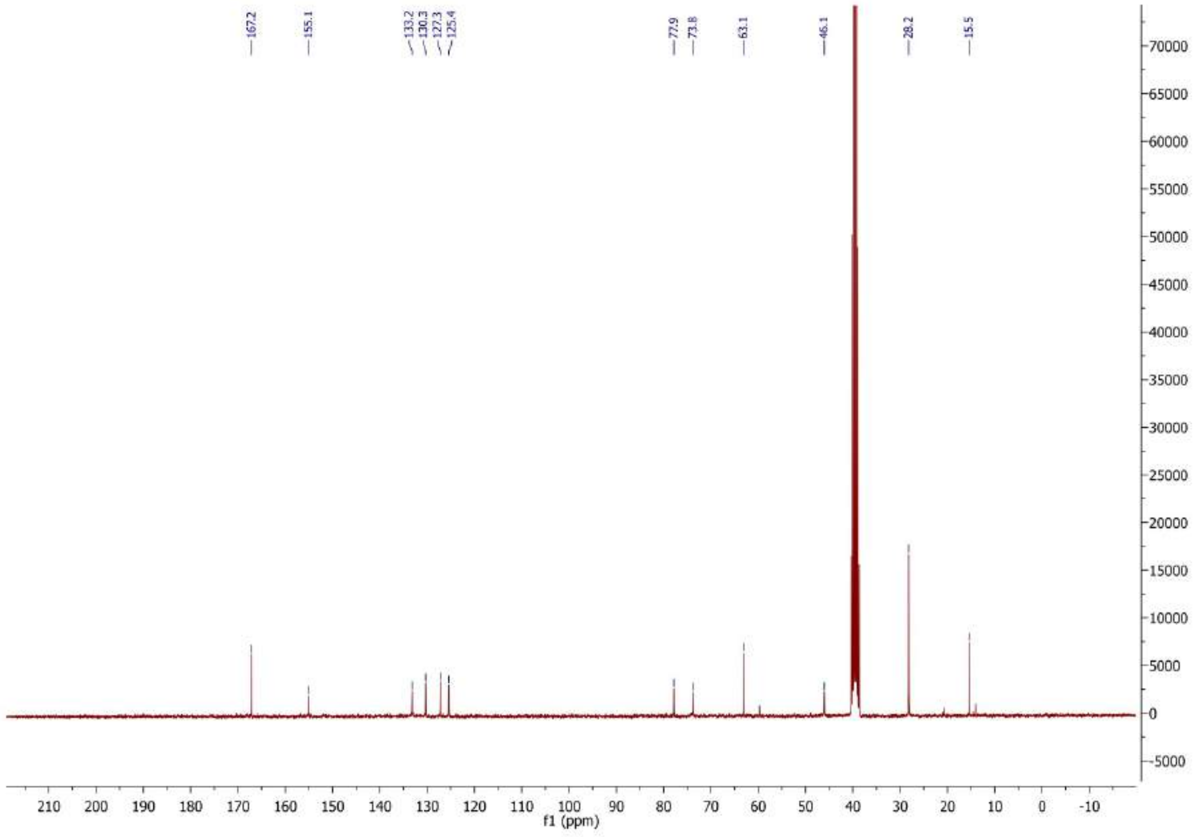

^{1}H-, ^{13}C- and ^{19}F-NMR-spectrum of(1*S*,6*S*)-2-Carboxy-6-ethoxycyclohexa-2,4-diene-1-ammonium 2,2,2-trifluoroacetate (*rac*-27) (*O*-Et-DHHA)

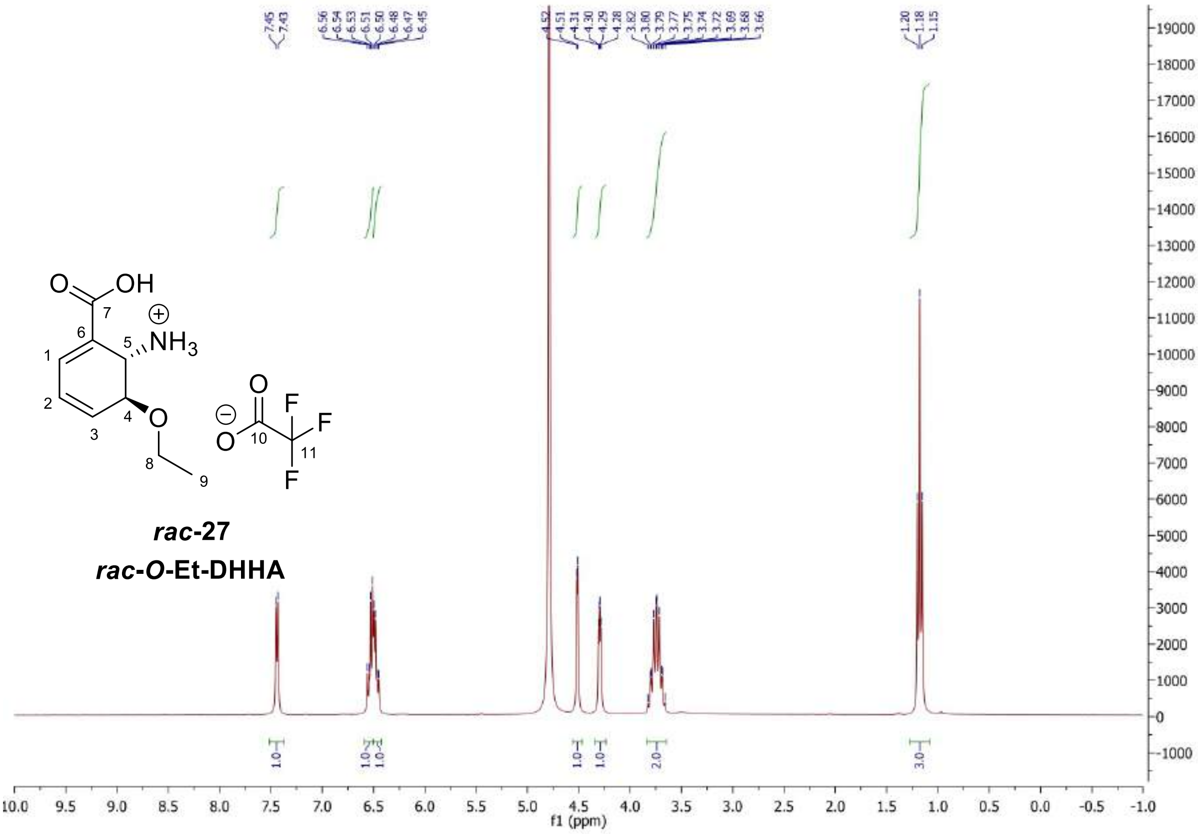

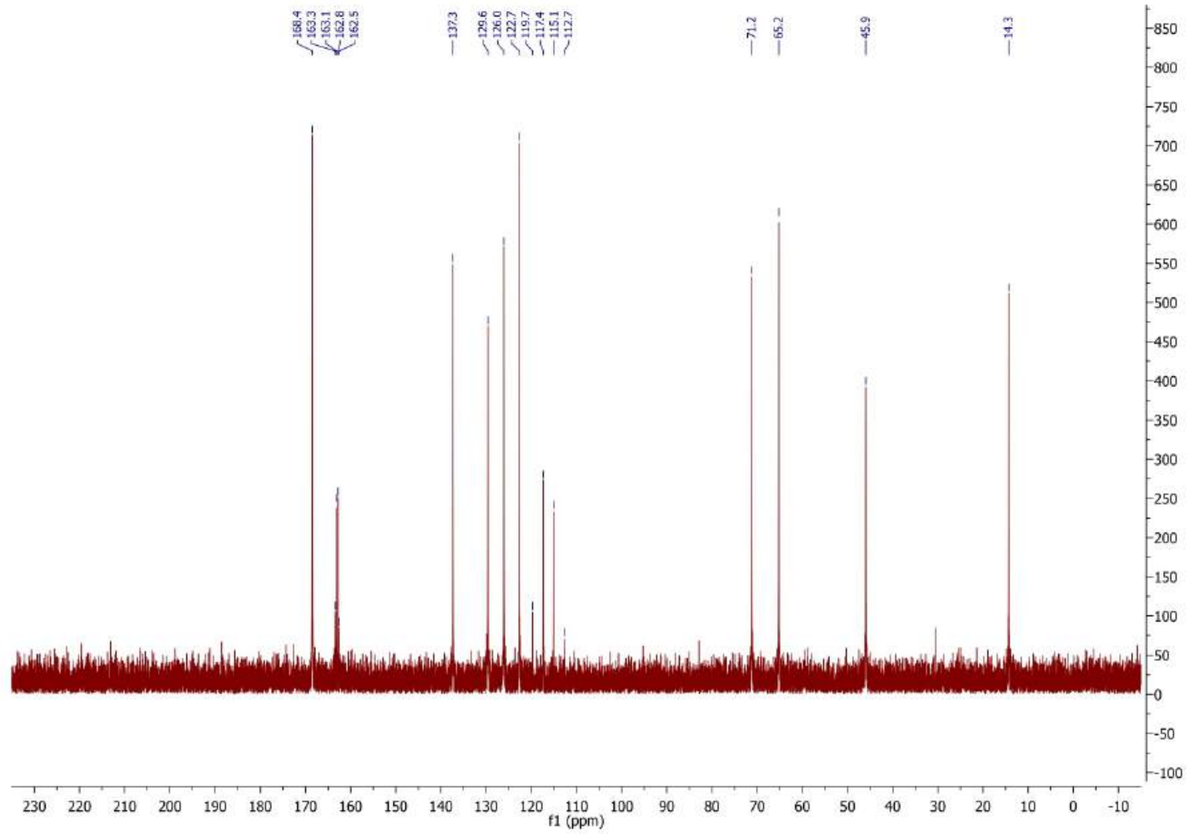

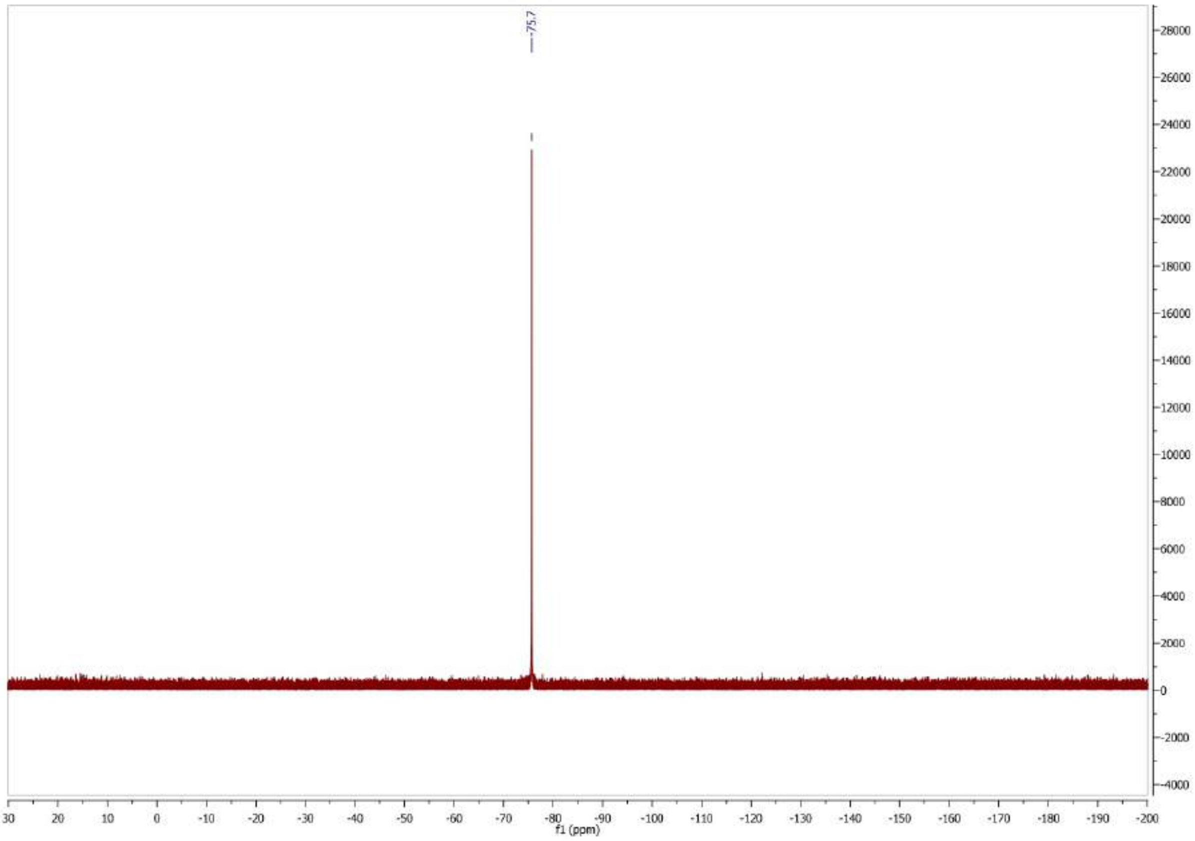

-75.7
28000
26000
24000
22000
20000
18000
16000
14000
12000
10000
8000
6000
4000
2000
0
-2000
-4000
30
20
10
0
-10
-20
-30
-40
-50
-60
-70
-80
-90
-100
-110
-120
-130
-140
-150
-160
-170
-180
-190
-200
f1 (ppm)

^{1}H- and ^{13}C-NMR-spectrum of ethyl (5*S*,6*S*)-6-((*tert*-butoxycarbonyl)amino)-5-propoxycyclohexa-1,3-diene-1-carboxylate (*rac*-35)

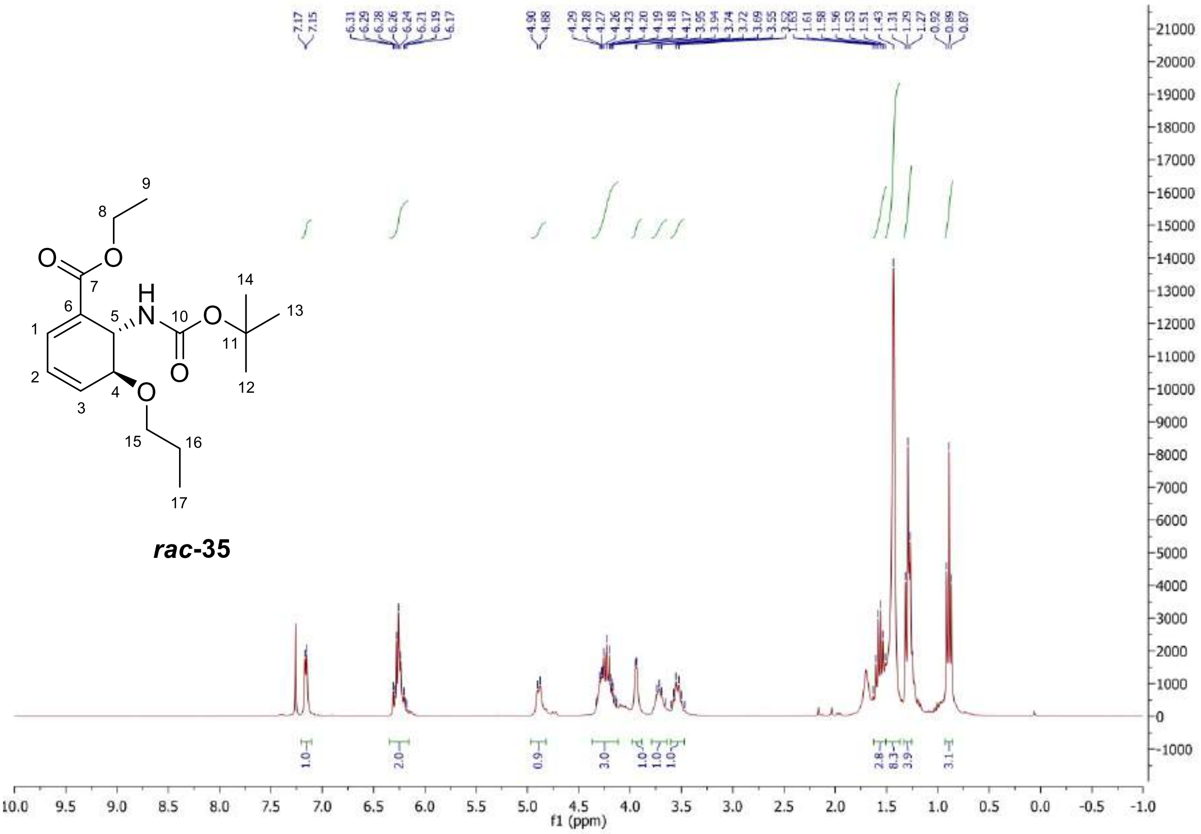

***rac*-35**

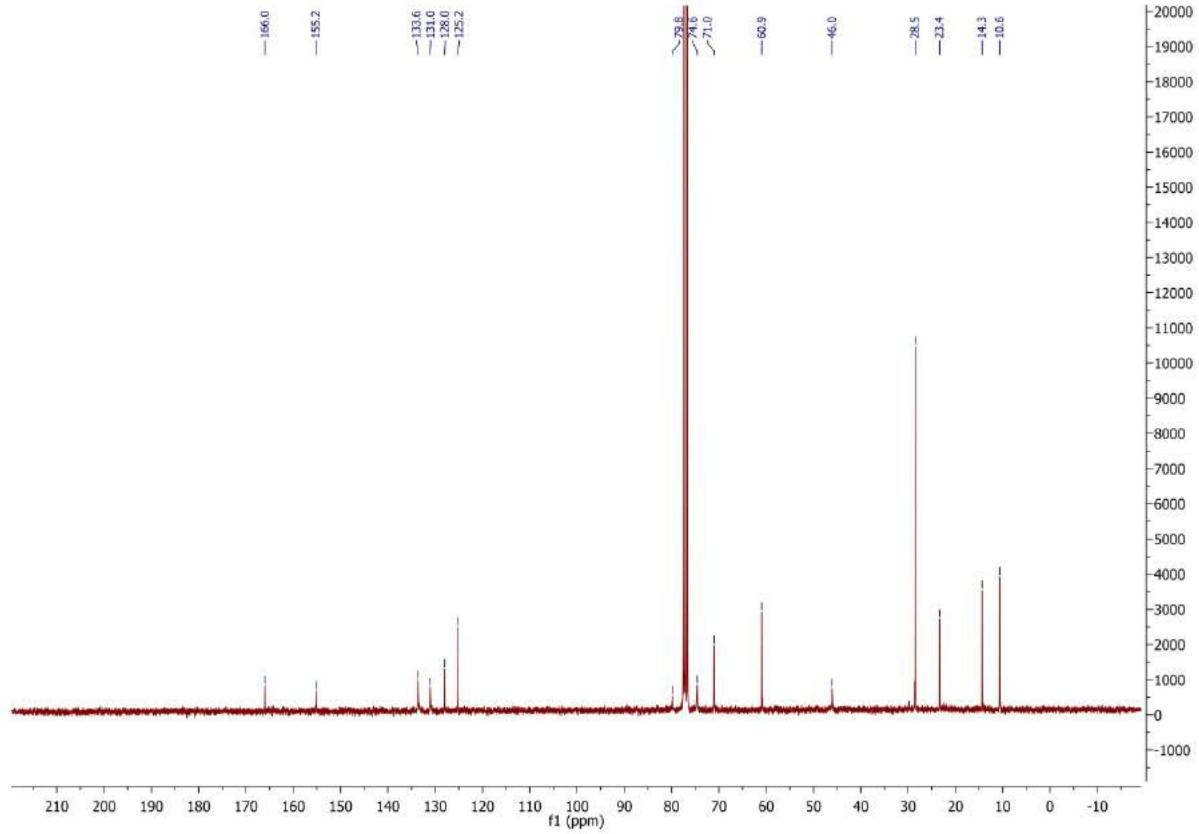

^{1}H- and ^{13}C-NMR-spectrum of (5*S*,6*S*)-6-((*tert*-butoxycarbonyl)amino)-5-propoxycyclohexa-1,3-diene-1-carboxylic acid (*rac*-36)

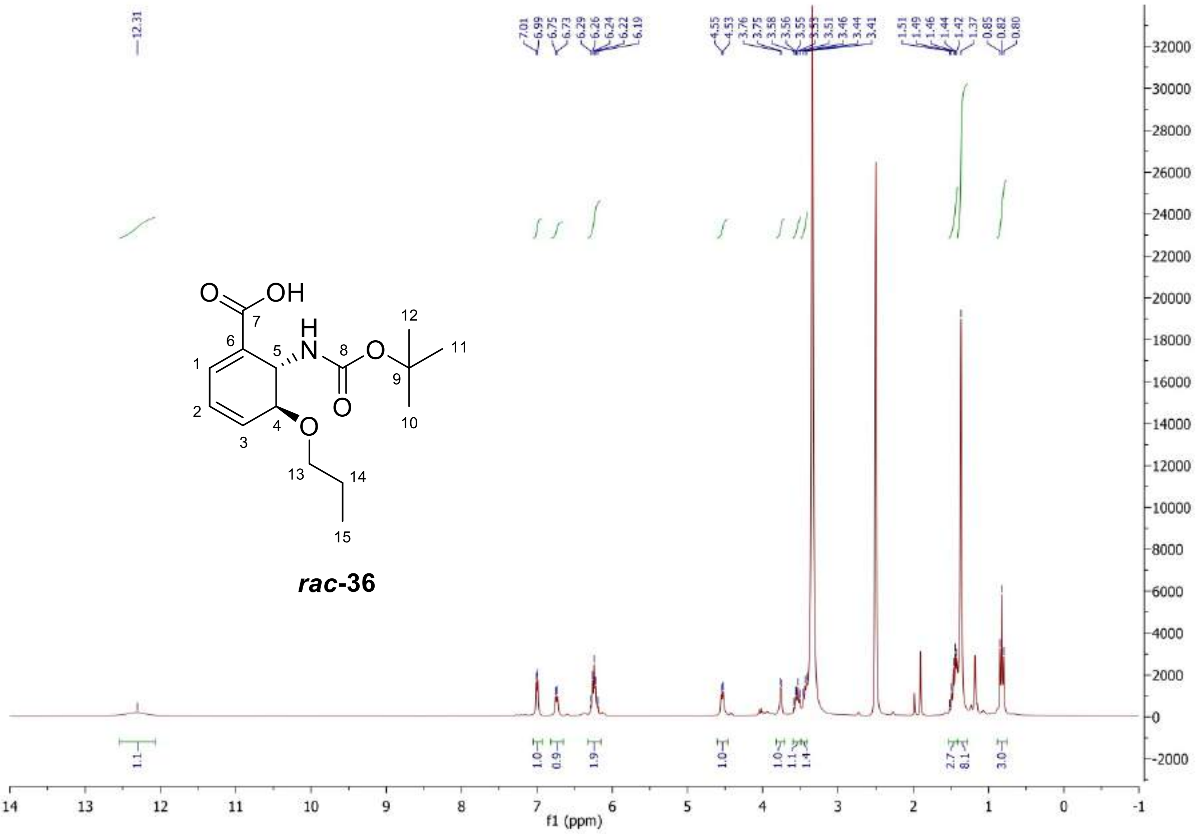

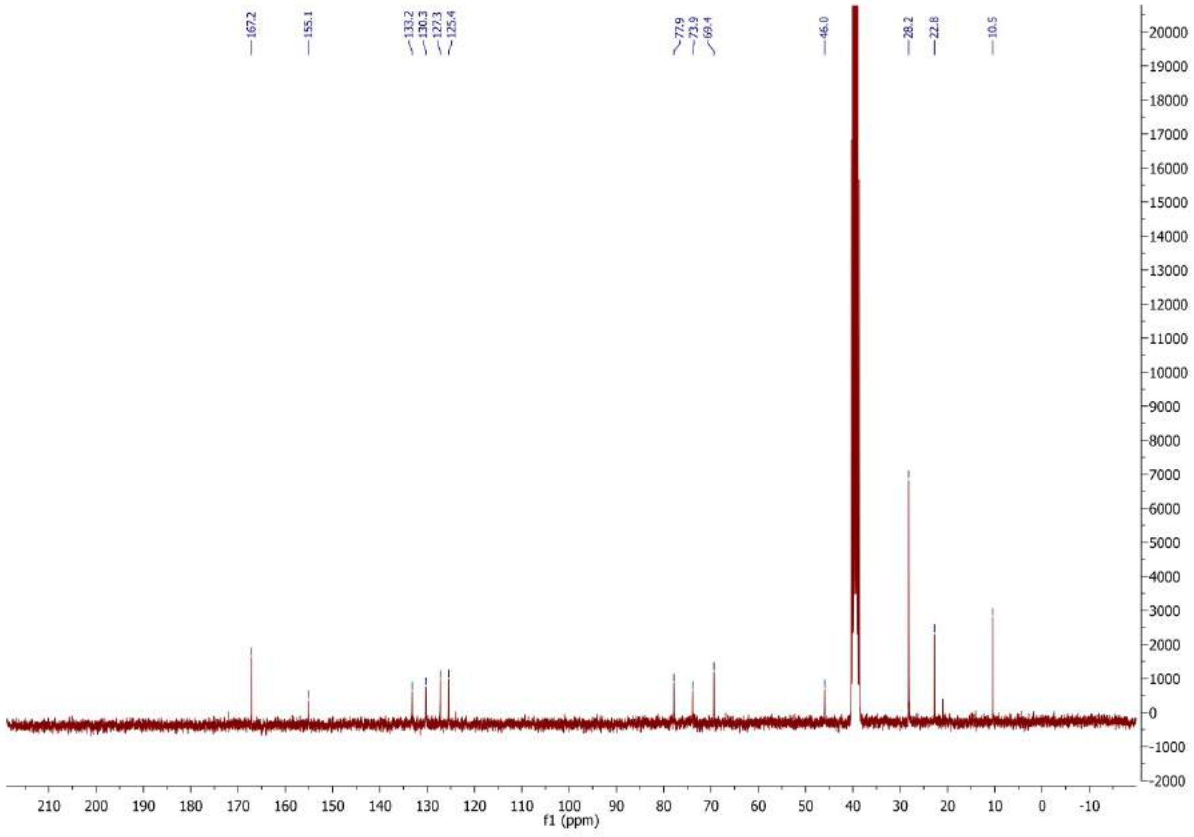

^{1}H-, ^{13}C- and ^{19}F-NMR-spectrum of (1*S*,6*S*)-2-carboxy-6-propoxycyclohexa-2,4-diene-1-ammonium 2,2,2-trifluoroacetate (*rac*-28) (*rac*-*O*-*n*Pr-DHHA)

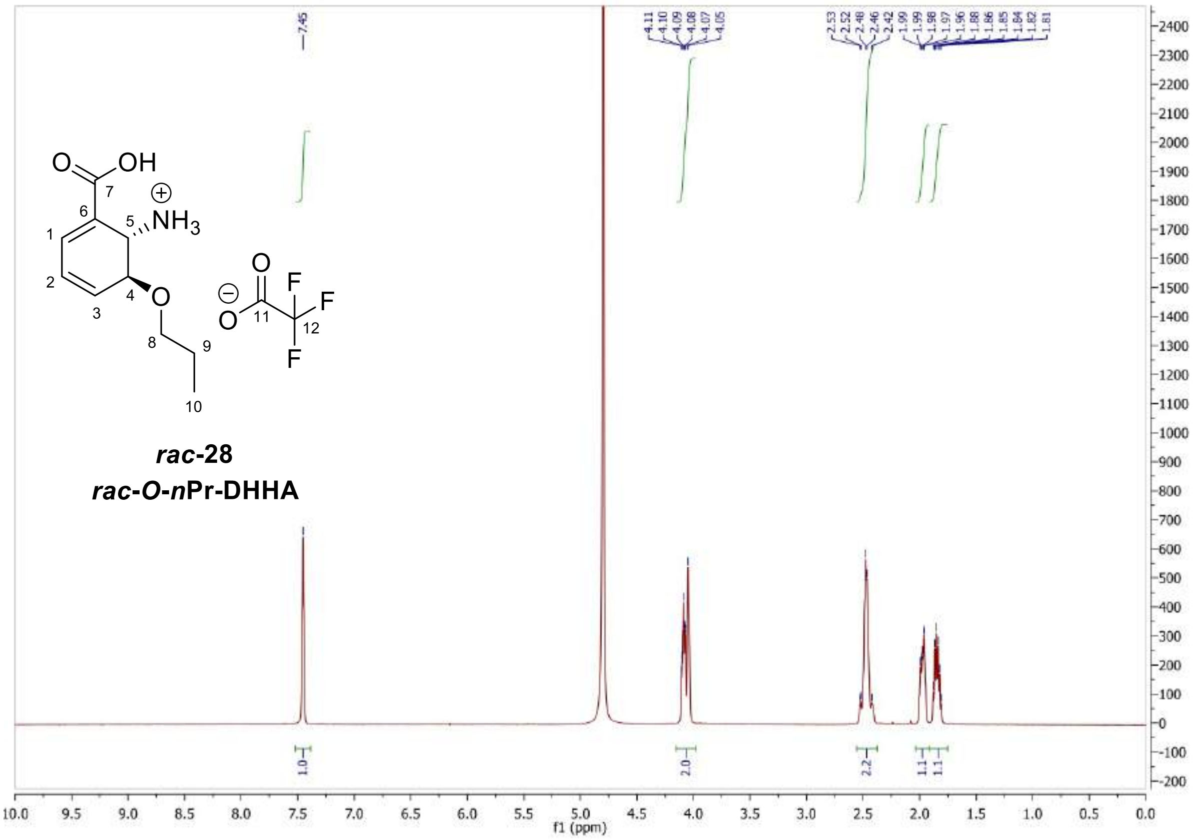

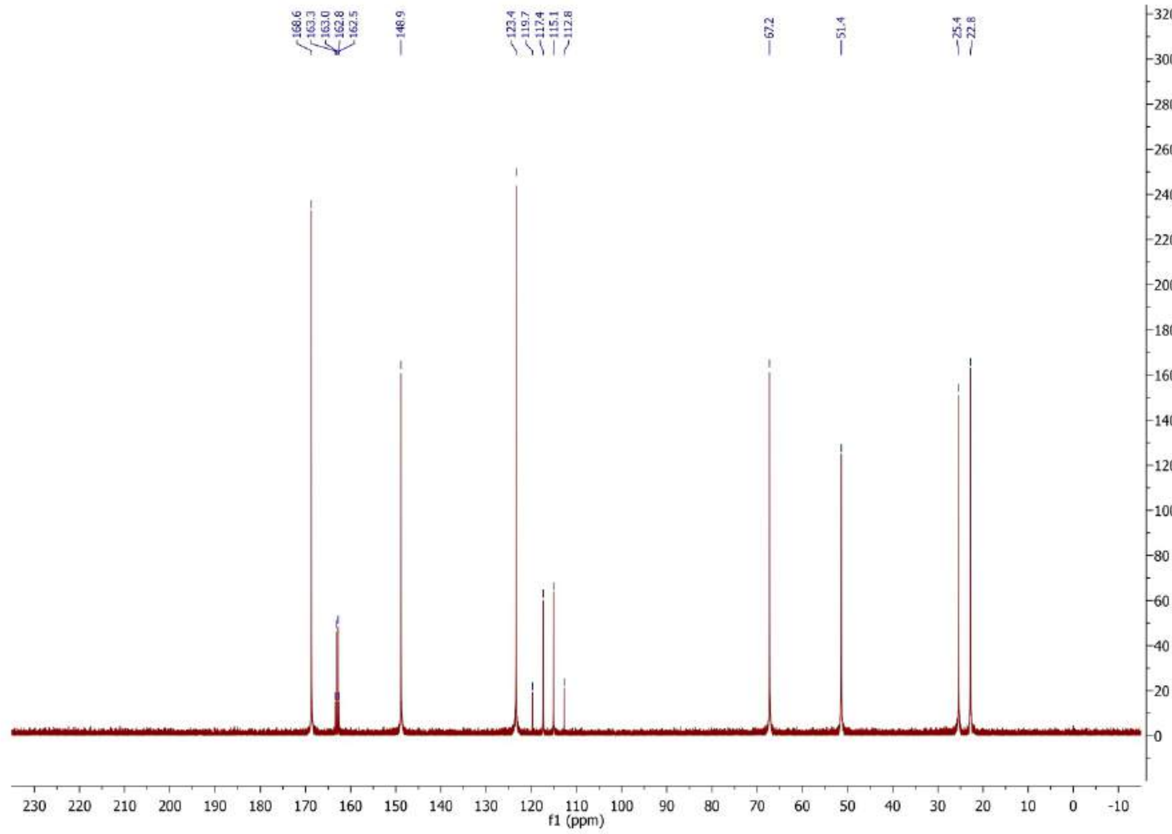

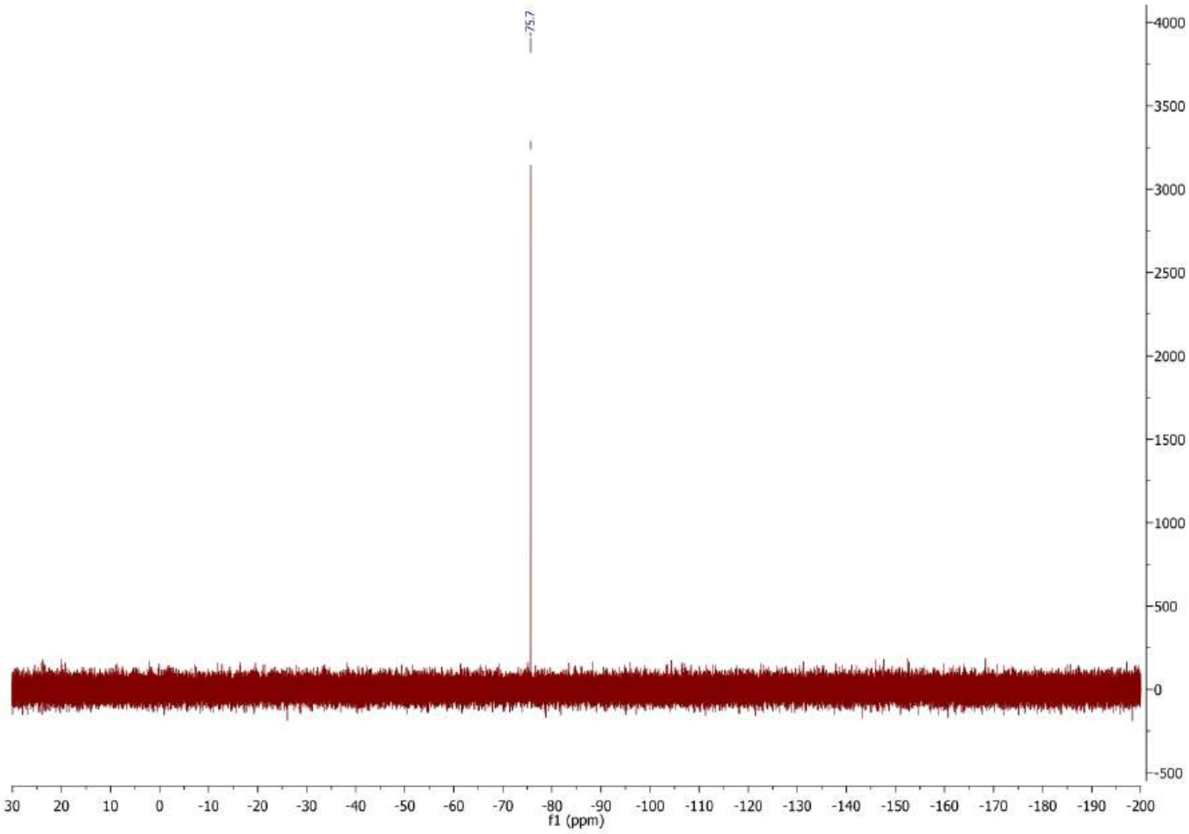
-75.7
f1 (ppm)

Supplemental References

1. Kabsch, W. XDS. *Acta Crystallogr. D Biol. Crystallogr.* **66,** 125–132 (2010).
2. Karplus, P. A. & Diederichs, K. Linking crystallographic model and data quality. *Science* **336,** 1030–1033 (2012).
3. Diederichs, K. & Karplus, P. A. Improved R-factors for diffraction data analysis in macromolecular crystallography. *Nat. Struct. Biol.* **4,** 269–275 (1997).
4. Weiss, M. S. Global indicators of X-ray data quality. *J. Appl. Crystallogr.* **34,** 130–135 (2001).
5. Chen, V. B. *et al.* MolProbity: all-atom structure validation for macromolecular crystallography. *Acta Crystallogr. D Biol. Crystallogr.* **66,** 12–21 (2010).
6. Blankenfeldt, W. *et al.* Structure and function of the phenazine biosynthetic protein PhzF from Pseudomonas fluorescens. *Proc. Natl. Acad. Sci. U.S.A.* **101,** 16431–16436 (2004).
7. Brooks, B. R. *et al.* CHARMM: the biomolecular simulation program. *J. Comput. Chem.* **30,** 1545–1614 (2009).
8. MacKerell, A. D. *et al.* All-atom empirical potential for molecular modeling and dynamics studies of proteins. *J. Phys. Chem. B* **102,** 3586–3616 (1998).
9. Neese, F. The ORCA program system. *Wiley Interdiscip. Rev. Comput. Mol. Sci.* **2,** 73–78 (2012).
10. Bashford, D. & Gerwert, K. Electrostatic calculations of the pKa values of ionizable groups in bacteriorhodopsin. *J. Mol. Biol.* **224,** 473–486 (1992).
11. Ullmann, R. T. & Ullmann, G. M. GMCT : a Monte Carlo simulation package for macromolecular receptors. *J. Comput. Chem.* **33,** 887–900 (2012).
12. Becke, A. D. Density-functional thermochemistry. III. The role of exact exchange. *J. Chem. Phys.* **98,** 5648–5652 (1993).
13. Weigend, F. & Ahlrichs, R. Balanced basis sets of split valence, triple zeta valence and quadruple zeta valence quality for H to Rn: Design and assessment of accuracy. *Phys. Chem. Chem. Phys. PCCP* **7,** 3297–3305 (2005).
14. Sinnecker, S., Rajendran, A., Klamt, A., Diedenhofen, M. & Neese, F. Calculation of Solvent Shifts on Electronic g-Tensors with the Conductor-Like Screening Model (COSMO) and Its Self-Consistent Generalization to Real Solvents (Direct COSMO-RS). *J. Phys. Chem. A* **110,** 2235–2245 (2006).
15. Field, M. J. The pDynamo Program for Molecular Simulations using Hybrid Quantum Chemical and Molecular Mechanical Potentials. *J. Chem. Theory Comput.* **4,** 1151–1161 (2008).
16. Becke, A. D. Density-functional exchange-energy approximation with correct asymptotic behavior. *Phys. Rev. A* **38,** 3098–3100 (1988).
17. Perdew, J. P. Density-functional approximation for the correlation energy of the inhomogeneous electron gas. *Phys. Rev. B Condens. Matter* **33,** 8822–8824 (1986).
18. Hehre, W. J., Ditchfield, R. & Pople, J. A. Self—Consistent Molecular Orbital Methods. XII. Further Extensions of Gaussian—Type Basis Sets for Use in Molecular Orbital Studies of Organic Molecules. *J. Chem. Phys.* **56,** 2257–2261 (1972).
19. Aleksandrov, A. & Field, M. A hybrid elastic band string algorithm for studies of enzymatic reactions. *Phys. Chem. Chem. Phys. PCCP* **14,** 12544–12553 (2012).
20. Fischer, S. & Karplus, M. Conjugate peak refinement: an algorithm for finding reaction paths and accurate transition states in systems with many degrees of freedom. *Chem. Phys. Lett.* **194,** 252–261 (1992).
21. Bigeleisen, J. The Relative Reaction Velocities of Isotopic Molecules. *J. Chem. Phys.* **17,** 675 (1949).
22. Mavrodi, D. V., Bleimling, N., Thomashow, L. S. & Blankenfeldt, W. The purification, crystallization and preliminary structural characterization of PhzF, a key enzyme in the phenazine-biosynthesis pathway from Pseudomonas fluorescens 2-79. *Acta Crystallogr. D Biol. Crystallogr.* **60,** 184–186 (2004).
23. Evans, P. R. & Murshudov, G. N. How good are my data and what is the resolution? *Acta Crystallogr. D Biol. Crystallogr.* **69,** 1204–1214 (2013).
24. Winn, M. D. *et al.* Overview of the CCP4 suite and current developments. *Acta Crystallogr. D Biol. Crystallogr.* **67,** 235–242 (2011).

25. Emsley, P., Lohkamp, B., Scott, W. G. & Cowtan, K. Features and development of Coot. *Acta Crystallogr. D Biol. Crystallogr.* **66,** 486–501 (2010).
26. Afonine, P. V. *et al.* Towards automated crystallographic structure refinement with phenix.refine. *Acta Crystallogr. D Biol. Crystallogr.* **68,** 352–367 (2012).
27. Adams, P. D. *et al.* PHENIX: a comprehensive Python-based system for macromolecular structure solution. *Acta Crystallogr. D Biol. Crystallogr.* **66,** 213–221 (2010).
28. Moriarty, N. W., Grosse-Kunstleve, R. W. & Adams, P. D. electronic Ligand Builder and Optimization Workbench (eLBOW): a tool for ligand coordinate and restraint generation. *Acta Crystallogr. D Biol. Crystallogr.* **65,** 1074–1080 (2009).
29. Schomaker, V. & Trueblood, K. N. On the rigid-body motion of molecules in crystals. *Acta Crystallogr. B* **24,** 63–76 (1968).
30. Berman, H. M. *et al.* The Protein Data Bank. *Nucleic Acids Res.* **28,** 235–242 (2000).
31. Bunnage, M. E., Ganesh, T., Masesane, I. B., Orton, D. & Steel, P. G. Asymmetric synthesis of the putative structure of (-)-oryzoxymycin. *Org. Lett.* **5,** 239–242 (2003).
32. Addo, J. K., Teesdale-Spittle, P. & Hoberg, J. O. Synthesis of 3-Nitropropanol Homologues. *Synthesis* **2005,** 1923–1925 (2005).
33. Masesane, I. B., Batsanov, A. S., Howard, J. A. K., Mondal, R. & Steel, P. G. The oxanorbornene approach to 3-hydroxy, 3,4-dihydroxy and 3,4,5-trihydroxy derivatives of 2-aminocyclohexanecarboxylic acid. *Beilstein J. Org. Chem.* **2,** 9 (2006).
34. Åhman, J. & Somfai, P. An Efficient Preparation of Potassium Bis (Trimethylsilyl) Amide (KHMDS). *Synth. Commun.* **25,** 2301–2303 (1995).
35. Ireland, R. E. & Meissner, R. S. Convenient method for the titration of amide base solutions. *J. Org. Chem.* **56,** 4566–4568 (1991).
36. Raheem, M.-A., Nagireddy, J., R., Durham, R. & Tam, W. Efficient procedure for the preparation of 2-bromofuran and its application in the synthesis of 2-arylfurans. *Synth Commun* **40,** 2138 (2010).
